# Genome-Wide Identification, Characterization, and Regulation of RWP-RK Gene Family in the Nitrogen-Fixing Clade

**DOI:** 10.3390/plants9091178

**Published:** 2020-09-11

**Authors:** Zhihua Wu, Hong Liu, Wen Huang, Lisha Yi, Erdai Qin, Tiange Yang, Jing Wang, Rui Qin

**Affiliations:** 1 Hubei Provincial Key Laboratory for Protection and Application of Special Plant Germplasm in Wuling Area of China & Key Laboratory of State Ethnic Affairs Commission for Biological Technology, College of Life Sciences, South-Central University for Nationalities, Wuhan 430074, China; zhwu@scuec.edu.cn (Z.W.); liuhong@mail.scuec.edu.cn (H.L.); 2017110270@mail.scuec.edu.cn (W.H.); 2018110278@mail.scuec.edu.cn (L.Y.); 13568822083@163.com (E.Q.); yangtge@163.com (T.Y.); 2 Institute of Food and Nutrition Development, Ministry of Agriculture, Chinese Academy of Agricultural Sciences, Beijing 100081, China; wangjing07@caas.cn

**Keywords:** RWP-RK, nitrogen-fixing clade, co-expression network, evolution, nodulation

## Abstract

RWP-RK is a plant-specific family of transcription factors, involved in nitrate response, gametogenesis, and nodulation. However, genome-wide characterization, phylogeny, and the regulation of RWP-RK genes in the nodulating and non-nodulating plant species of nitrogen-fixing clade (NFC) are widely unknown. Therefore, we identified a total of 292 RWP-RKs, including 278 RWP-RKs from 25 NFC species and 14 RWP-RKs from the outgroup, *Arabidopsis thaliana*. We classified the 292 RWP-RKs in two subfamilies: the NIN-like proteins (NLPs) and the RWP-RK domain proteins (RKDs). The transcriptome and phylogenetic analysis of RWP-RKs suggested that, compared to *RKD* genes, the *NLP* genes were just upregulated in nitrate response and nodulation. Moreover, nodule-specific *NLP* genes of some nodulating NFC species may have a common ancestor (OG0002084) with *AtNLP* genes in *A. thaliana*. Further, co-expression networks of *A.thaliana* under N-starvation and N-supplementation conditions revealed that there is a higher correlation between expression of *AtNLP* genes and symbiotic genes during N-starvation. In *P. vulgaris*, we confirmed that N-starvation stimulated nodulation by regulating expression of *PvNLP2*, closely related to *AtNLP6* and *AtNLP7* with another common origin (OG0004041). Taken together, we concluded that different origins of the *NLP* genes involved in both N-starvation response and specific expression of nodulation would contribute to the evolution of nodulation in NFC plant species. Our results shed light on the phylogenetic relationships of *NLP* genes and their differential regulation in nitrate response of *A. thaliana* and nodulation of NFC.

## 1. Introduction

Nitrogen (N) is an essential element for crop growth, productivity, and grain quality. The extensive use of synthetic fertilizers in developed countries is expensive and environmentally damaging. Much effort has been made to improve N use efficiency (NUE) in crop plants to allow high-yielding crops to be grown with low N input without significant yield losses [1]. NUE can be improved via the interaction of plants with microorganisms, such as rhizobia. When legumes interact with rhizobia, newly formed nitrogen-fixing root nodules (NFN) use nitrogen from the atmosphere via symbiotic nitrogen fixation [2]. Many studies have explored the mechanism of symbiotic nitrogen fixation, with the goal of engineering plants that can directly fix nitrogen for crop improvement [3]. RWP-RKs, which contain a conserved RWP-RK DNA binding motif, are a class of transcription factors (TFs) that control N uptake efficiency and N utilization by sensing nitrate signals [4]. These plant-specific TFs are grouped into two subfamilies: nodule inception (NIN)-like proteins (NLPs) and RWP-RK-domain proteins (RKDs) [5]. Compared to the subfamily of RKDs, NLPs contain an additional domain known as PB1 (Phox and Bem 1) at their C-termini, that allows interactions with additional proteins [4]. In non-nodulating *Arabidopsis thaliana*, RKDs are highly expressed in reproductive organs, highlighting their regulatory importance in female gametophyte development [6], while NLPs play a central role in nitrate signaling by binding to the nitrate-responsive *cis*-elements in their target genes [7]. NLPs also play important roles in nodulation, as well as the crosstalk of nitrate signaling pathway and symbiotic signaling pathway. The TF NIN (nodule inception) of NLPs specifically evolved in the NFC (nitrogen-fixing clade) originated 100-110 million years ago [8,9]. Nodule formation is dependent on the perception of limited N levels by the plant [10,11]. For example, NLP1 functions through PB1-mediated interactions with NIN, leading to the suppression of nodulation in *Medicago truncatula* [12].

The co-expression network is a valuable tool for revealing sets of genes that function in specific biological processes [13]. For example, comparisons of co-expression networks between human and chimpanzee brains identified key drivers of evolutionary change in brain development [14]. As a model dicot, non-nodulating *A. thaliana* has many advantages to study interactions between diazotrophic bacteria and dicots [15], and therefore, the knowledge of *A. thaliana* is helpful to translate biological knowledge from model organisms to crops. Since the recent release of multiple genomes of legumes and non-legumes of NFC are capable or incapable of undergoing nodulation [8,16], the distribution, evolutionary features, regulation of RWP-RKs in the NFC has not been subjected to detailed analysis. Therefore, a compared co-expression network analysis of both model and non-model plants will provide new insights into the evolutionary features of the RWP-RK family in NFC, and reveal possible relationships between the nitrate signaling pathway and symbiosis mediated by RWP-RK family members. Our understanding of RWP-RKs’ roles in nitrate signaling in *A. thaliana* could facilitate the improvement of NUE in crops via an evolutionary and comparative analysis [15,17].

In this study, we provided an insight into the evolution of RWP-RK genes in 25 nodulating and non-nodulating NFC plant species and *A. thaliana*, through genome-wide distribution, characterization, and regulation. First, we constructed a phylogenetic tree based on genome-wide gene duplication events. Next, we studied the physicochemical properties, genomic positions, gene structure and protein motifs. Then, we revealed the evolutionary relationship of RWP-RKs that putatively mediate nitrate and symbiosis responses in *A. thaliana*, *Phaseolus vulgaris* and *Glycine max*, through the analysis of differentially expressed genes and co-expression networks in these species. We then validated our hypothesis by using qPCR of *NLP* genes in *P. vulgaris* under different nitrogen regimes. 

## 2. Results

### 2.1. Phylogeny of NFC Species and Distribution of RWP-RKs

**Figure 1:**
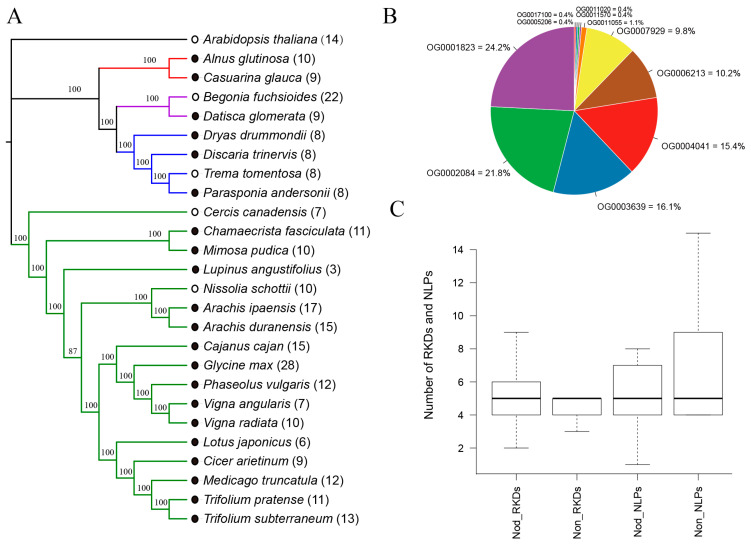
Origin and distribution of RWP-RKs in nitrogen-fixing clade (NFC). (**A**) Phylogeny of NFC species based on gene duplication events constructed using the maximum-likelihood method. The numbers on branches represent bootstrap values, and the numbers in round brackets represent the number of RWP-RK family members. The nodulating plants are indicated by filled circles, and the non-nodulating plants are indicated by empty circles. Each color line represents different orders of NFC, Fagales with red, Cucurbitales with purple, Rosales with blue, and Fabales with green. (**B**) Origins of RWP-RKs, as indicated by the distributions of different orthogroups. (**C**) Distribution of RWP-RK-domain proteins (RKDs) and nodule inception (NIN)-like proteins (NLPs) between nodulating plants (Nod) and non-nodulating plants (Non).

Gene duplication events have a major impact on the evolution of nodulation [18]. Large numbers of gene and genome duplication events have occurred in plants, and hence, no single-copy orthogroups are present in all plant species [19]. We studied 25 NFC species, of which 21 plants can form nodules, and the four remaining species lack the capacity for nodulation (Table S1). To learn about whether the genome-wide gene duplication events have an effect on the divergence of nodulating and non-nodulating plants in NFC, we inferred phylogenetic tree for 26 species using STRIDE (Species Tree Root Inference from Gene Duplication Events) [19], and found that the phylogeny of NFC species (Figure 1A) is consistent with species tree inferred from Angiosperm Phylogeny Group [20]. This indicated that genome-wide gene duplication events occurred in NFC did not cause direct divergence of nodulating and non-nodulating plants. A total of 292 RWP-RK domains and 150 PB1 domains has been identified in 26 species (Figure S1 and Table S1). Members of RWP-RK family with the only RWP-RK domain are defined as RKDs, while those containing both RWP-RK and PB1 domains are defined as NLPs. The number of RKDs appears to be randomly distributed in each order of NFC, regardless of nodulation status (Figure 1A and Table S1). For example, non-nodulating *Begonia fuchsioides* contains the same number (15) of RKDs as that of nodulating *G. max*. An orthogroup is defined as a set of genes that descended from a single gene in the last common ancestor of the analyzed species [21]. To explore the possible origins of all the identified RWP-RKs, we grouped the RWP-RKs into different orthogroups. Except for 7 members, the 285 RWP-RKs clustered into 11 orthogroups containing 1–69 RWP-RKs genes, suggesting that RWP-RKs have diverse origins (Figure 1B and Table S2). The presence of RWP-RKs with multiple origins might help these species adapt their growth and metabolism in response to fluctuations in nitrogen availability in different habitats, as legumes are exceptionally ecologically diverse [22,23]. The isoelectric points of the 292 RWP-RKs range from 4.73 to 10.6, and the molecular weights range from 7.14 kDa to 156.01 kDa. Most RWP-RKs (263 of 292) were predicted to localize to the nucleus, followed by chloroplast (22), extracellular space (5) and plasma membrane (2) (Table S3). Despite the similar median values for each subfamily (RKDs and NLPs) between nodulating and non-nodulating plants, the numbers of NLPs in non-nodulating are more than in nodulating plants (Figure 1C). Therefore, compared to RKDs, NLPs may play more diverse roles in non-nodulating plants than in nodulating plants. 

An alignment of the domains revealed that the 49th site (K, Lys) and 63th site (R, Arg) are conserved across all RWP-RKs (Figure S2). Except for *Arachis ipaensis* Araip.KR88K, *B. fuchsioides* Begfu255S10103, and *B. fuchsioides* Begfu91828S44750, which have lost the RWP-RK motif, all species contain the conserved RWP-RK signature, with some modifications, such as HWP-RK (HWPHRK of Araip.YWB61, Fabales), NWP-RK (NWPHRK of C.cajan_36762, Fabales), WWP-RK (WWPYRK of Datgl206S24145, Cucurbitales), HWP-RK (HWPSRK of Datgl229S25120, Cucurbitales), KWP-RQ (KWPHRQ of Glyma.04G054800.1.p, Fabales), and KWP-RK (KWPQRK of Glyma.06G054900.1.p) (Figure S2). Interestingly, almost all of these modifications occurred in the first amino acid of the conserved RWP-RK motif in nodulating Fabales and Cucurbitales plants. The 4th K, 13th R, 62th D, and 75th D sites of the PB1 domains of RWP-RKs are conserved across nodulating and non-nodulating plants (Figure S3). 

### 2.2. Phylogeny and Characteristics of the NFC RWP-RKs

**Figure 2:**
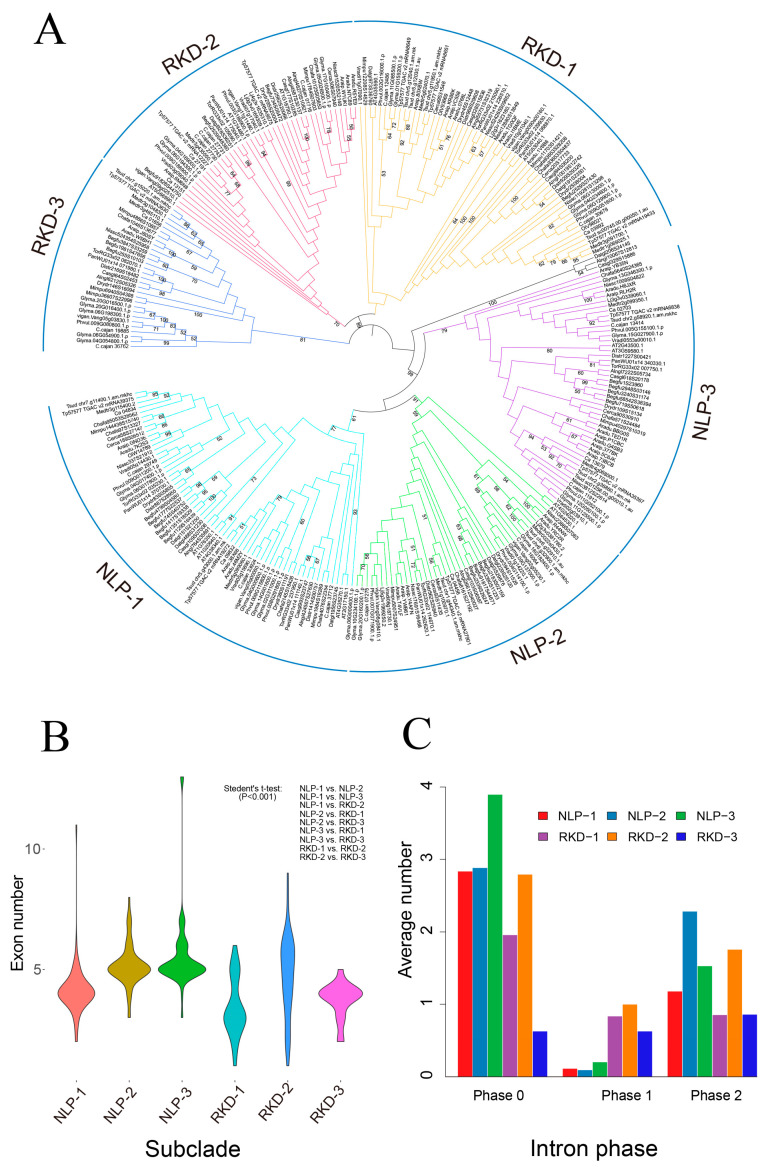
Phylogeny and exon features of RWP-RK proteins. (**A**) Gene tree constructed by the neighbor-joining method with 1000 bootstrap values. The bootstrap values ranging from 50 to 100 were shown on each branch. (**B**) Exon number for each subclade indicated by violin plots. Statistical tests with significance were shown on the top right. (**C**) Average number of exons for six subclades of RWP-RKs with phase 0, phase 1 and phase 2.

To investigate the phylogenetic relationships of RWP-RKs between NFC and *A. thaliana*, we constructed a phylogenetic tree of 292 RWP-RKs via the neighbor-joining method with 1000 bootstrap values. To limit issues related to high divergence between proteins, we selected the RWP-RK domains with 30 additional amino acids upstream and downstream of the domains for alignment and phylogenetic analysis. The tree formed six clades based on the relationships of the NLPs and RKDs in *A. thaliana*. The NLP subfamily clustered into a single clade with three subclades, NLP-1 (AtNLP1–AtNLP5), NLP-2 (AtNLP6, AtNLP7), and NLP-3 (AtNLP8, AtNLP9), containing all NLPs with PB1 domains. The RKD subfamily includes RKD-1 (AtRKD4, AtRKD5), RKD-2 (AtRKD1, AtRKD2, AtRKD3), and RKD-3, which was NFC-specific (Figure 2A). The NLP subfamily also includes several genes without PB1 domains Glyma.06G000400.1, vigan.Vang06g08410.1, vigan.Vang02g05230.1, Araip.YWB61, Araip.377BK, and Araip.5C6JK, which may have experienced the diverse evolution and widespread distribution of PB1 domains across all kingdoms of life in nature [24]. 

Gene structure analysis showed that the average number of exons in the NLPs and RKDs is 4.9 and 4.0, respectively. The numbers of exons in each subclade of NLPs are 4.1 for NLP-1, 5.3 for NLP-2, and 5.6 for NLP-3. The numbers of exons in each subclade of RKDs are 3.7 for RKD-1, 5.6 for RKD-2, and 3.5 for RKD-3 (Figure 2B and Table S4). Therefore, the average number of exons is higher in the NLPs than in the RKDs. Of the 50 consensus protein motifs, in addition to the three common motifs (including known RWP-RK and PB1 motifs) in both the RKDs and NLPs, the NLPs contain 39 enriched motifs, whereas the RKDs contain only eight enriched motifs. Each subclade contains some unique motifs, such as motif #24 in NLP-1, motif #18 in NLP-2, and motif #23 in NLP-3. In addition, members of NFC-specific RKD-3 contain several unique motifs, including motif #34, motif #37, and motif #41 (Figure S4, Figure S5 and Table S5). These unique motifs for each subclade may indicate their particular functions when interacting with other proteins [4].

Statistical analysis of intron phases revealed that, except for NFC-specific RKD-3, all other RWP-RKs contain the most phase 0 introns, which cause no disruption of a codon, followed by phase 2 and phase 1, which disrupt the codon between bases 2 and 3 or bases 1 and 2, respectively. However, RKDs contain more phase 1 introns than NLPs with phase 1 introns, and RKD-3 contains the most phase 2 introns compared to phase 0 and 1 (Figure 2C). Overall, the results of exon number and intron phase analyses were consistent with the phylogenetic tree, but there were some exceptions. For example, *Cerca58S27147* has more short additional exons than other members of the NLP-1 clade, but lacks additional protein motifs (Figure S5). Taken together, these results suggested that different subclades of RWP-RKs have different evolutionary features at the gene structure and protein level.

### 2.3. Comparison of RWP-RKs in Non-Nodulating (A. thaliana) vs. Nodulating Plants (G. max and P. vulgaris)

**Figure 3:**
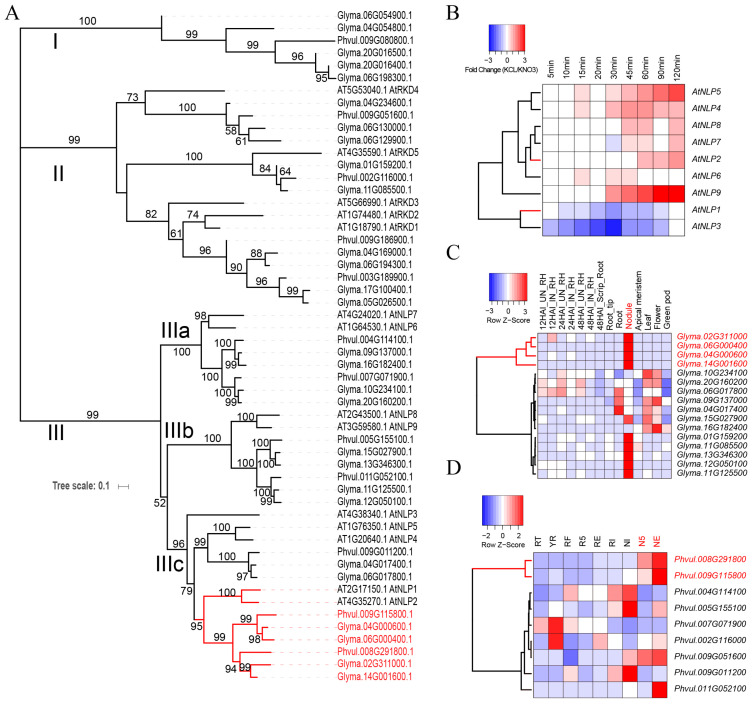
Phylogeny and comparison of RWP-RK expression patterns among *Arabidopsis thaliana*, *Phaseolus vulgaris*, and *Glycine max*. (**A**) Gene tree of RWP-RK proteins with bootstrap values ranging from 50 to 100 on each branch. (**B**) Fold changes of RWP-RKs in comparison of N-starvation roots (KCl treatment) to N-supplementation roots (KNO_3_ treatment) of *A*. *thaliana*. (**C**) Expression pattern of RWP-RKs in multiple tissues of *G*. *max.* 12HA1_IN_RH, 12HA1_UN_RH, 24HA1_IN_RH, 24HA1_UN_RH, 48HA1_IN_RH, 48HA1_UN_RH, 48HA1_Scrip_Root represent root hairs (RH), and stripped roots inoculated (IN) and mock-inoculated (UN) with *B. japonicum* at 12, 24, and 48 HAI (hours after inoculation); 18-day-old root tip, root and leaf, 18-day-old apical meristem, flower from R2 stage, green pod from R6 stage, nodule harvested 32 days after the inoculation. (**D**) Expression pattern of multiple tissues in *P. vulgaris*. RWP-RKs with high expression patterns in nodules were marked with red. In *P. vulgaris*, RT, root tips collected from fertilized plants; YR, whole roots, including root tips; RF, whole roots from fertilized plants; R5, whole roots separated from 5 day old pre-fixing nodules; RE, whole roots separated from effective nitrogen-fixing root nodules (NFN) collected 21 days after inoculation (DAI); RI, whole roots separated from ineffective NFN collected 21 DAI, NI, ineffective NFN collected 21 DAI; N5, effective pre-fixing nodules 5 DAI; NE, effective NFN 21 DAI.

We classified the RWP-RKs from *A. thaliana* (14), *G. max* (28), and *P. vulgaris* (12) into three clades: unique RKDs in *G. max* and *P. vulgaris* (Clade I), RKDs in all three species (Clade II, including AtRKD1–AtRKD5), and NLPs in all three species (Clade III, including AtNLP1–AtNLP9) (Figure 3A). We then integrated time-series transcriptome datasets from *A. thaliana*, including root samples treated with KCl (defined as N starvation) and KNO_3_ (defined as N supplementation) [25], as well as expression atlases from *G. max* and *P. vulgaris* [26,27]. In *A. thaliana*, all *AtRKD* genes are not expressed in roots, whereas *AtNLP* genes are differentially regulated depending on the concentrations of nitrogen. Specifically, *AtNLP1* and *AtNLP3* are downregulated, and other *AtNLP* genes are upregulated under N starvation, indicating that the different *AtNLP* genes have different responses to N starvation (Figure 3B). In *G. max* and *P. vulgaris*, *NLP* genes are expressed in different tissues, with some specifically expressed in the nodules (*Glyma.02G311000*, *Glyma.06G000400*, *Glyma.04G000600*, *Glyma.14G001600*, *Phvul.008G291800*, and *Phvul.009G115800*). These nodule-specific genes clustered together, and were closely related to *AtNLP*1 and *AtNLP*2 within subclade IIIc (Figure 3A–D). Phylogenetically, a close relationship of these genes implies that nodule *NLP*, *AtNLP1* and *AtNLP2* genes shared the recent common ancestor with the similar functions. Taking the phylogenetic relationships and expression clusters of *A. thaliana* and *G. max* and *P. vulgaris* together, we concluded that, compared to NLPs of IIIa and IIIb, nodule-specific *NLP* genes of NFC have an ancestry of origin (OG0002084) with *AtNLP1*, *AtNLP2*, *AtNLP4* and *AtNLP5* of non-NFC, which was also supported by our result of orthogroup analysis (Figure 1B). The shared ancestry of nodule-specific *NLP* and *AtNLP* genes indicates that the genetic improvement of these *NLP* genes would possibly contribute to nodulation in non-NFC.

### 2.4. The Involved Biological Processes of AtNLPs-Associated Modules under N starvation Condition

**Figure 4:**
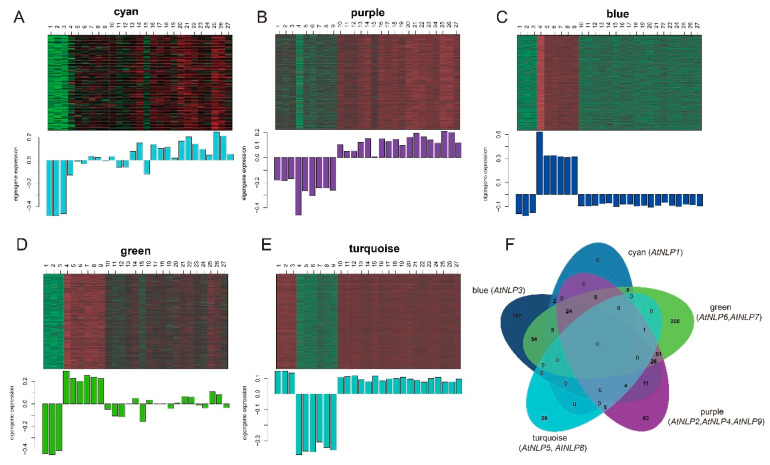
Modules containing *AtNLP* genes correspond to functional subdivisions of the N-starvation datasets. (**A**) Cyan module with *AtNLP*1. (**B**) Purple module with *AtNLP*2, *AtNLP*4, and *AtNLP*9. (**C**) Blue module with *AtNLP*3. (**D**) Green module with *AtNLP*6 and *AtNLP*7. (**E**) Turquoise module with *AtNLP*5 and *AtNLP*8. (**F**) Venn diagram of enriched gene ontology terms for each module at the biological process level. The sample accessions above the heatmap represent each time point with three replications seen in Varala, et al. [29], 5 min (1–3), 10 min (4–6), 15 min (7–9), 20 min (10–12), 30 min (13–15), 45 min (16–18), 60 min (19–21), 90 min (22–24), and 120 min (25–27).

Despite the important roles of nitrates in plant growth, N limitation, including N starvation or low N, is essential for nodulation in legumes [11]. To investigate the possible relationship between N starvation and nodulation, we explored differences in the modules containing different *AtNLPs* under N starvation vs. N supplementation. We used WGCNA (weighted correlation network analysis) to construct a weighted network from N-starvation datasets with *rlog* normalization (Figure S6), and identified a total of 12 modules of co-expressed genes in the network. We found that expression correlation (rho = 0.99) is more conserved than connectivity correlation (rho = 0.84) under N starvation (Figure S7). Gene connectivity is defined as the sum of connection strengths with the other genes in the network [28]. Less conserved connectivity, indicating the less conserved correlation among genes, suggested that changes of genes’ correlation under N starvation play more important roles than changes in expression levels, as shown in a comparison of human and chimpanzee brains [14]. *AtNLPs* were distributed in five of the 12 identified modules under N-starvation (Figure 4A–E). Different modules showed different expression patterns across a series of time points, with the earliest response of the turquoise module followed by blue and green modules, purple module. GO enrichment analysis of genes in the biological process category revealed no commonly enriched biological processes in the five modules (Figure 4F and Table S6). This indicated that these module genes suffered from different regulation and were involved in different biological processes under the sustained N starvation. For example, genes in the blue module (with *AtNLP3*) were relatively upregulated at the early stage (10–15 min of treatment). We discovered uniquely enriched GO terms in the blue module, such as “endocytosis” (GO:0006897, *P* = 2.6 × 10^−4^). The uniquely enriched GO terms in the green module (with *AtNLP6* and *AtNLP7*) included many important terms, such as “cell wall organization or biogenesis” (GO:0071554, *P* = 9.3 × 10^−27^), “cell wall organization” (GO:0071555, *P* = 8.5 × 10^−20^), “plant-type cell wall organization or biogenesis” (GO:0071669, *P* = 8.2 × 10^−15^), “cell wall modification” (GO:0042545, *P* = 4.1 × 10^−9^), “root hair elongation” (GO:0048767, *P* = 3.6 × 10^−6^), “root hair cell development” (GO:0080147, *P* = 3.4 × 10^−5^), and “auxin-activated signaling pathway” (GO:0009734, *P* = 5.4 × 10^−4^). Interestingly, the blue and green modules were both enriched in “symbiosis, encompassing mutualism through parasitism” (GO:0044403, *P* = 9.5 × 10^−4^), with 36 and 24 genes, respectively (Table S6). Although genes in both modules were highly expressed only at 10–15 min under the treatment of N starvation, the genes enriched for the GO term “symbiosis, encompassing mutualism through parasitism” did not overlap, highlighting the independent roles of genes regulated by *AtNLP3*, *AtNLP6*, and *AtNLP7* within each module. In the purple module (with *AtNLP2*, *AtNLP4* and *AtNLP9*), in which genes were continuously upregulated after 20 min under the treatment of N starvation, we detected a high proportion of unique GO terms associated with transport (20/54), such as “calcium ion transport” (GO:0006816, *P* = 2.8 × 10^−5^) (Table S6), which may be required for “calcium spiking”, a symbiotic signaling event. Taken together, the biological processes involved in the response to N starvation at early stage may explain the reason why nodulation only occurred under free or low nitrogen conditions and high nitrogen will inhibit nodulation.

**Figure 5:**
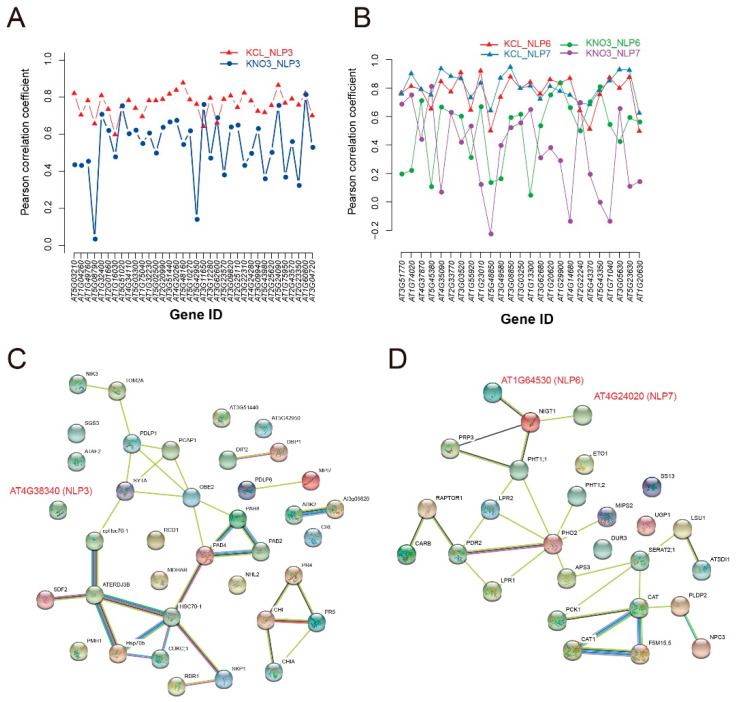
Relationships of *AtNLP3*, *AtNLP6*, and *AtNLP7* to genes involved in symbiosis (GO:0044403) and starvation (GO:0009267) under N starvation. (**A**) Correlation of *AtNLP*3 with genes from the enriched symbiosis-related GO term in the blue module. (**B**) Correlation of *AtNLP6* and *AtNLP*7 with genes from the symbiosis-related GO term in the green module. (**C**) Protein association network of AtNLP3 with proteins from the symbiosis-related GO:0044403 (symbiosis, encompassing mutualism through parasitism) constructed with STRING 11.0. (**D**) Protein association network of AtNLP6 and AtNLP7 with proteins from the symbiosis-related GO:0009267 (cell response to starvation) constructed with STRING 11.0.

Correlation analysis showed that *AtNLP*3 was more highly correlated with genes in GO categories GO:0044403 (symbiosis, encompassing mutualism through parasitism), while *AtNLP*6 and *AtNLP*7 were more highly correlated with genes in GO categories GO:0009267 (cell response to starvation) under N-starvation than under N-supplementation conditions (Figure 5A,B). In addition, *AtNLP3* was downregulated under N starvation compared to N supplementation (Figure 3B). The downregulation of *AtNLP3*, along with the downregulation of immune response genes, may be beneficial for rhizobial invasion via reducing the immune response. The expression levels of *AtNLP6* and *AtNLP7*, which have high sequence similarity, changed little (fold change < 2) between N-starvation and N-supplementation conditions. AtNLP7 is a major regulatory element among NLP proteins [30]. Despite their weak upregulation at the transcriptional level, *AtNLP6* and *AtNLP7* might be regulated primarily at the protein level rather than the transcriptional level, as previously reported [30]. Compared with co-expressed relationships, protein association networks predicted with STRING 11.0 showed that AtNLP3, AtNLP6 and AtNLP7 were little associated with genes of GO:0044403 (symbiosis, encompassing mutualism through parasitism) and GO:0009267 (cell response to starvation), respectively (Figure 5C,D). AtNLP6 and AtNLP7 were both associated with AT1G13300 (NIGT1/HRS1), which is regulated by AtNLP7 [31]. *NIGT1*/*HRS1* is induced by NO_3_^-^ [31], and the high expression correlation between *AtNLP6*/*AtNLP7* and *NIGT1*/*HRS1* in the green module (Table S7) further indicated that these genes were strongly co-expressed under N starvation conditions. Taking the network analysis of *AtNLP* genes, different origins of *NLP* genes showed the diverse regulation of biological processes under N starvation, which may be essential for nodulation under N limitation. 

### 2.5. Effects of High and Low Nitrogen on Nodulation via the Reglation of PvNLP Genes in P. vulgaris

**Figure 6:**
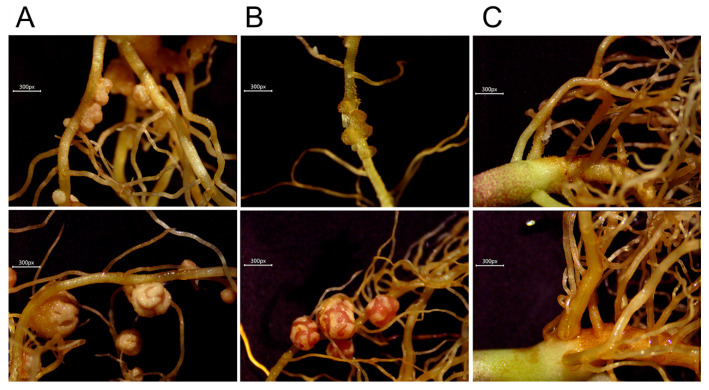
Phenotypes of early nitrogen-prefixing nodules and nitrogen-fixing root nodules (NFN) under different concentrations of nitrate with 0 mM (**A**), 5 mM (**B**) and 10 mM (**C**). Under each concentration, the top represents phenotype of early nitrogen-prefixing nodules and the bottom represent phenotype of NFN.

Not only are NLPs involved in the nitrate signaling pathway, but they also influence nodulation via the integration between nitrate and symbiotic signaling pathway [12]. How nitrate influences nodulation via the regulation of specific NLP members in legumes is unclear. Here, we investigated the expression pattern of *PvNLP* genes under nitrogen-free (0 mM), nitrogen-low (5 mM) and nitrogen-high (10 mM) conditions along with inoculation of rhizobia. We found that 10 mM nitrate inhibited nodulation in both young nitrogen-prefixing root nodules and NFN. NFN from plants treated with 5 mM nitrate appeared to be browner, and plant height was taller, than for plants treated with either 0 mM or 10 mM nitrate (Figure 6A–C, Figures S8 and S9). 

**Figure 7:**
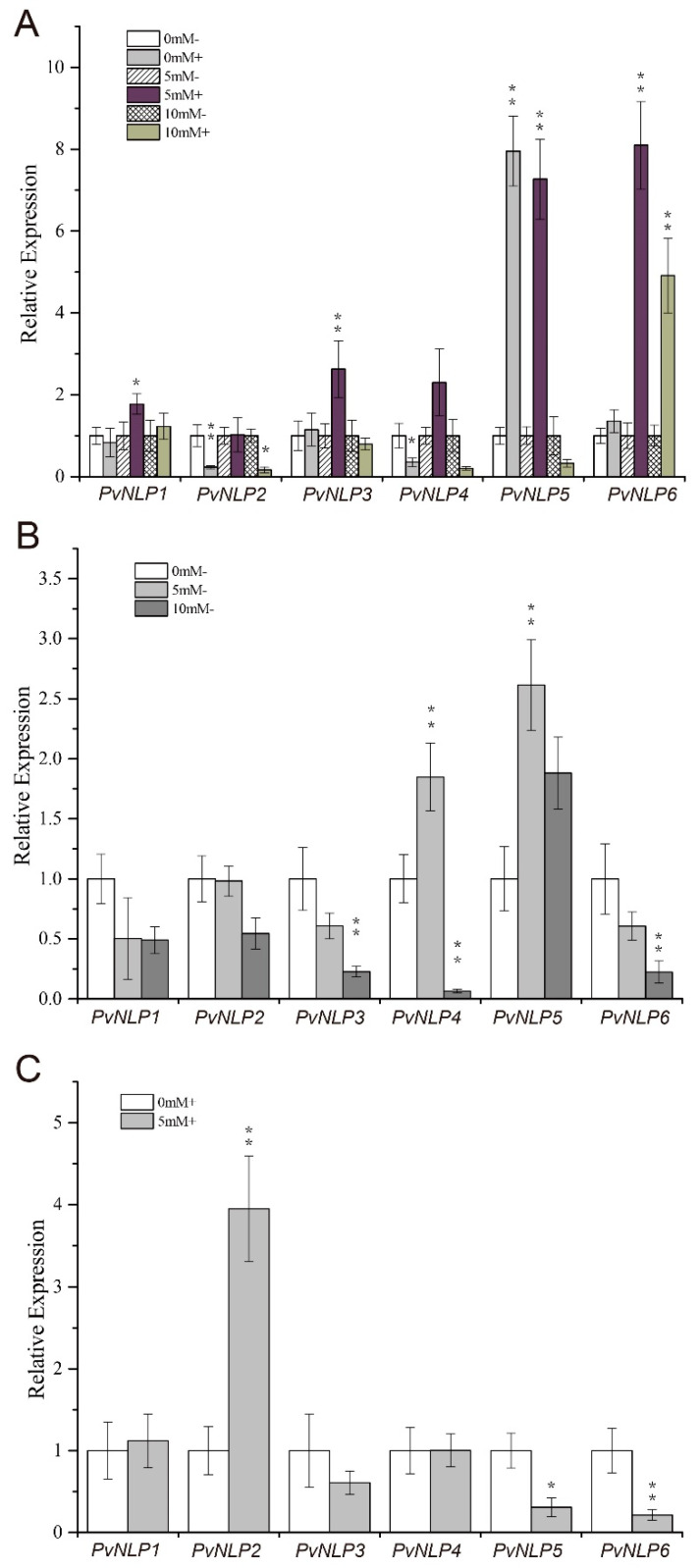
Expression analysis of selected *PvNLP* genes in early roots, early nodules, late roots, and nitrogen-fixing nodules (NFN) treated with different concentrations of nitrates. (**A**) Early roots and root-nodule mixtures treated with (+) or without (-) inoculation and 0 mM, 5 mM, or 10 mM KNO_3_. (**B**) Late roots treated without inoculation and 0 mM, 5 mM, or 10 mM KNO_3_. (**C**) NFN treated with inoculation and 0 mM or 5 mM. Student’s *t*-test was performed for two groups of inoculated and uninoculated samples under the same concentration of nitrogen (such as 0 mM- versus 0 mM+) in **A**; 0 mM- versus 5 mM-, 0 mM- versus 10 mM- in **B**; and 0 mM+ versus 5mM+ in **C**, * *p* < 0.05, ** *p* < 0.01, *** *p* < 0.001.

Expression analysis of six *PvNLP* genes in early roots and root-nodule mixtures under different concentrations of nitrates indicated that *PvNLP1*, *PvNLP3*, and *PvNLP6* showed higher expression at 5 mM nitrate (low-nitrogen conditions) with inoculation than they did under either 0 mM nitrate (nitrogen-free conditions) or 10 mM nitrate (high-nitrogen conditions), regardless of inoculation (Figure 7A and Table S8). When the roots were treated with a different concentration of nitrates without inoculation, *PvNLP4* and *PvNLP5* showed highest expression under low-nitrogen as compared to both nitrogen-free and high-nitrogen conditions, whereas the other genes showed gradual inhibition with increasing nitrogen concentration. Finally, *PvNLP3*, *PvNLP4*, and *PvNLP6* were significantly inhibited under high-nitrogen conditions (Figure 7B). In NFN, *PvNLP2* was significantly upregulated under low-nitrogen vs. nitrogen-free conditions, whereas other genes were downregulated or varied in expression pattern (Figure 7C). These results indicated the differential regulation of *PvNLP* genes in the development of nodules under different nitrogen conditions, and the *PvNLP2* (phylogenetically related to *AtNLP6* and *AtNLP7*) with common ancestor of OG0004041 is vital for nodulation via sensing nitrate signaling under low nitrogen.

## 3. Discussion

### 3.1. Distribution and Features of RWP-RKs in NFC

In contrast to animals, the success of angiosperms is partially attributed to innovations caused by gene or whole-genome duplications [32]. An accurate phylogenetic tree of species is required for evolutionary comparisons of RWP-RKs. It is impossible to deduce a phylogenetic tree from one-to-one corresponding orthologues, in that few of these orthogroups in 26 species contain just one orthologue from each species. Therefore, we used a newly developed method for phylogenetic tree construction based on the analysis of gene duplications for all genes [33]. Our phylogenetic tree suggested that the whole genome-wide duplication of genes is not the direct driver of the evolution of nodulation across the NFC, which is also supported by the finding that the evolutionary relationship between polyploidy and nodulation is not sufficient to make a species able to form nodules [34]. 

Variation of motifs of both the RWP-RK and PB1 domains caused by insertion/deletion may provide novel regulatory function for different RWP-RK members. For example, AtNLP8 with insertion in the RWP-RK domain functions as a master regulator of nitrate-promoted seed germination, and is activated by nitrate via a mechanism different from AtNLP7 without insertion [35]. Whether this insertion within RWP-RK of AtNLP8 is involved in this specific mechanism (as is the case for AtNLP6) remains to be explored. Variations in the domain lengths of RWP-RK and PB1 and specific amino acid sites (resulting in noncanonical domains) might mediate noncanonical interactions during various biological events [36]. 

Our phylogenetic analysis of NFC RWP-RKs supported the functional divergence of RKDs and NLPs [4]. Meanwhile, high numbers of exons increase the chances for alternative splicing of precursor mRNAs, which is important for gene regulation [37]. In addition, the excess of phase 0 introns of the six subclades in RWP-RKs is also found in other cases, supported by prediction of the exon theory of genes [38]. However, the RKDs have more exons with phase 1 introns than NLPs with phase 1 introns, which indicates different origins of intron-exon structures for RKDs and NLPs, respectively [39].

### 3.2. Effects of N Starvation on Nodulation Possibly Mediated by Regulation of NLP Genes

The most important nodule-bearing legume crops, such as *G. max* and *P. vulgaris*, have not been studied in as much detail as *A. thaliana*. RWP-RKs play important roles in both nitrate responses and nodule inception, and they interact with each other to coordinate nitrate signaling and nodulation [12]. Therefore, we felt that a comparison of RWP-RK expression and homology in *A. thaliana* vs. legume crops (*G. max* and *P. vulgaris*) would allow gene functional analyses in model organisms to be applied to nodulating crops. Such studies might also facilitate the transfer of the nitrogen-fixing trait into non-nodulating plants to improve NUE [3,40]. Interestingly, *AtRKD1*–*AtRKD4* are highly expressed in reproductive organs, and *AtRKD5* has pleiotropic effects on phytohormone pathways, highlighting the regulatory importance of *AtRKD* genes in female gametophyte development [6,41].

Modules including *AtNLP*-connected genes involved different biological processes, and may provide differential prerequisites for nodulation in NFC under N limitation, which supported the fact that high nitrogen inhibits nodulation. For example, GO term of “endocytosis” (GO:0006897) in the blue module may involve the formation of organelle-like structures known as “symbiosomes” during early rhizobial invasion; GO term of “auxin-activated signaling pathway”(GO:0009734) of *AtNLP6* and *AtNLP7* involved green module further indicated auxin signaling is required for rhizobial infection as reported in *Medicago truncatula*. The cell wall starts to weaken when the growing infection thread is close to the base of the root hair. Therefore, unique genes associated with the term “cell-wall modifications” may also be essential for nodule initiation. The GO term “cell cycle” (GO:0051726) in the purple module may be related to the rhizobial infection [42]. These unique GO terms involved in specific biological processes reflect the diverse regulations of *AtNLPs* under N-starvation conditions. Different originated *AtNLP* genes may have differential regulated roles in symbiosis under N starvation. In future, NFC *NLP* genes with the same origins as *AtNLP* genes would need to be further studied for their functions.

### 3.3. Simulation of Low Nitrogen on Nodulation Mediated by Differential Expression of PvNLP Genes in P. vulgaris

It is reported that high nitrate repressed nodulation, while nodulation only occurred under low nitrates or free nitrates [10,11,43]. In our experiment, we found that the effects of nitrate on nodulation were possibly mediated by regulation of *PvNLP* genes in *P. vulgaris*. The stimulation of nodulation by low nitrogen treatment has also been observed in *G. max* [44]. In *Lotus japonicus*, the NLP NITRATE UNRESPONSIVE SYMBIOSIS 1 (NRSYM1) is a key regulator of the nitrate-induced control of root nodule symbiosis [45]. Phvul.007G071900.1 (PvNLP2), closely related to NRSYM1 (Figure S10), showed the uniquely high expression pattern of *PvNLP2* in NFN under low-nitrogen conditions. This indicated that *PvNLP2* is involved in integrating nitrate signaling and nodule symbiosis in *P. vulgaris*. Moreover, we determined that PvNLP2 and NRSYM1 are closely related to AtNLP6 and AtNLP7. This finding points to the functional similarity of these proteins in integrating the nitrate-signaling pathway and nodulation. This notion is supported by the observation that transformation with *AtNLP6* or *AtNLP7* partially rescues the nodulation phenotype of the *L. japonicus nrsym1* mutant [45]. Combined with our network analysis of AtNLP6 or AtNLP7 under N starvation, we inferred that the NFC *NLP* genes closely to AtNLP6 or AtNLP7 might be involved in integrating symbiosis and nitrate signaling under N-starvation. The orthogroup analysis showed that these genes have the same ancestor (OG0004041). Compared to nitrogen-free conditions, we propose that the differential upregulation of genes under low-nitrogen conditions partially explains the stimulatory effects of low-nitrogen treatment on nodulation, which might occur via the upregulation of specific *PvNLP* genes in *P. vulgaris*. Meanwhile, the diverse response of other *PvNLP* members under different concentrations of nitrogen showed possibly different roles.

## 4. Materials and Methods

### 4.1. Inference of Species Tree Based on Gene Duplication Events 

The complete protein sequences of 25 NFC species and *A. thaliana* with available whole-genome sequences were compiled. To avoid false annotation as much as possible, only the proteins with complete coding sequences encoded by standard genetic codes were retained. All-against-all BLASTP (BLAST 2.7.1+) was conducted for complete proteins from 26 plant species. STRIDE [19] implemented in OrthoFinder-2.2.7 was used for phylogenetic analysis, with *A. thaliana* as the outgroup based on deduced gene duplication events. Orthogroups of the 25 NFC species and *A. thaliana* were deduced using OrthoFinder-2.2.7 [21].

### 4.2. Identification and Characterization of RWP-RK Genes

To identify the most complete set possible of the RWP-RK family in the 25 NFC species, 131 RWP-RKs from nine taxa covering the plant kingdom were retrieved from PlantTFDB 4.0 [46]. These were used to BLAST (BLAST 2.7.1+) [47] against all proteins of the 26 species with at least e-value of 1 × 10^−5^ and identity of 50%. The species (from nine taxa) contained *Micromonas* sp. RCC299 (Chlorophytae), *Klebsormidium flaccidum* (Charophyta), *Marchantia polymorpha* (Marchantiophyta), *Physcomitrella patens* (Bryophyta), *Selaginella moellendorffii* (Lycopodiophyta), *Picea abies* (Coniferophyta), *Amborella trichopoda* (Basal Magnoliophyta), *Oryza sativa* ssp. Indica (Monocots) and *A. thaliana* (Eudicots). The hidden Markov model (HMM) profile of the RWP-RK family (PF02042) was extracted from the Pfam 32.0 database [48] used for searching using HMMER 3.2 [49]. After integration of the results from BLASTP and HMMER and removal of redundancy, the results were further checked using InterProScan [50], and genes with conserved RWP-RK domain were retained for subsequent analysis. Basic bioinformatics analysis of various features of the proteins including the molecular weight (MW), isoelectric point (pI), and length were performed using ExPASy (http://www.expasy.ch/tools/pi_tool.html), and the subcellular localizations of the proteins were predicted using BUSCA [51]. Due to the lack of exon information in the annotated gff3 file of *Trifolium subterraneum*, gene structures were predicted for RWP-RKs from the remaining 25 species using GSDS 2.0 [52]. Additional conserved motifs were identified using the MEME 5.0.1 package [53], with the parameters: minimum motif width, 6; maximum motif width, 50; and maximum number of motifs, 50.

### 4.3. Phylogenetic Analysis of RWP-RKs

All identified RWP-RK proteins were aligned using ClustalW implemented in MEGA X. The best model for the aligned matrix was evaluated with ProteinModelSelection.pl (https://cme.h-its.org/exelixis/web/software/raxml/), and the Jones–Taylor–Thornton (JTT) model was selected for phylogenetic analysis. The phylogenetic tree was built with MEGA X using the neighbor-joining method with 1000 bootstrap values [54].

### 4.4. Co-Expression Network Analysis

The primitive transcriptome data of *A. thaliana* under N-starvation (treatment with 20 mM KCl) and N-supplement (treatment with 20 mM KNO_3_ + 20 mM NH_4_NO_3_) conditions were downloaded from the previous report (GSE97500) for subsequent normalization [25]. For transcriptome atlases of *P. vulgaris* (SRX695931) [27] and *G. max* (SRX017401 and SoyBase) [26,55], the primitive reads were downloaded and treated as follows: controlled for read quality with Trimmomatic 0.36 [56]; aligned with HISAT 2.1.0 [57]; converted to read counts with featureCounts 1.5.3 [58]; and normalized with *regularized logarithm* (*rlog*) using DESeq2 [59]. The *rlog* normalized expressed matrix was further transformed into an adjacency matrix based on the simulation of soft threshold for network construction [13]. Co-expression networks were constructed for *A. thaliana* using WGCNA_1.64-1 [28]. In brief, a signed, weighted correlation network was constructed using the dynamic tree cut method [60], and the resulting modules were merged based on the correlations of module eigengenes (Pearson correlation coefficient, PCC > 0.8). Then, the connectivity in each module was calculated using the intramodularConnectivity() function for adjacency matrixes implemented in the WGCNA _1.64-1 package. For each gene, the connectivity (also known as degree) is defined as the sum of connection strengths with the other network genes. In co-expression networks, the connectivity measures how correlated a gene is with all other network genes [28].

### 4.5. Plant Materials, Growth and Treatment Conditions

*P. vulgaris* (cultivar Tianmadidou) seeds were purchased from Shijiazhuang Xianfeng Seed Industry Co., Ltd, and sterilized with 95% ethanol for 1 minute (min), followed by 0.1% HgCl for 15 min, and transferred to 0.8% water agar for germination in the dark for 2 days (d), then for 2 d under a 16 h/8 h (light/dark) photoperiod at 23 ℃, 45% relative humidity in pots containing a 1:1 mixture of vermiculite and perlite. When the first true leaf emerged, 2 mL *Rhizobium tropici* CIAT899 (OD_600_ = 0.2) purchased from Culture Collection of China Agricultural University (CCBAU) was used to inoculate the *P. vulgaris* roots, and Fahraeus medium (FM) nutrient solution was added every 4–6 days. The plants were grown in nitrate-free FM supplemented with 0 mM (free nitrogen), 5 mM (low nitrogen), and 10 mM KNO_3_ (high nitrogen) under inoculated or non-inoculated conditions. Because early nodules are too small to sample, the early nitrogen-prefixing nodules were defined as root-nodule mixtures at 2 cm below the stem, which were obtained on the 7th day after inoculation (DAI), while NFN were sampled on the 21st DAI. Samples of the corresponding roots at the similar positions of nodulation were obtained from non-inoculated plants treated with the same concentrations of KNO_3_. Each treatment was performed with three independent replications.

### 4.6. Quantitative RT-PCR

The roots or nodules of *P. vulgaris* were immediately frozen in liquid nitrogen for RNA extraction. The qRT-PCR was performed to analyze the relative expression levels of genes in plants inoculated with and without rhizobia under different concentrations of nitrogen, using *actin* (*Phvul.008G011000*) gene for the control. The reaction volume was 10 μL, containing 0.2 μL of each gene-specific primer, 0.2 μL of cDNA, 5 μL of SYBR Green, and 4.4 μL of sterile distilled water. The qRT-PCR cycling conditions were as follows: 95 °C for 30 s, followed by 40 cycles at 95 °C for 10 s and 60 °C for 30 s; solubility curve of 95 °C for 15 s, 60 °C for 60 s, and 95 °C for 15 s. All reactions were performed in triplicate, and the relative expression level (2^-∆∆Ct^ value) was calculated based on normalization to the *actin* gene. The gene-specific primers and gene identifiers of *PvNLP* genes are listed in Table S8. The primers were designed with Primer 5.0 software and checked for specificity by BLASTN. A significance test of differential expression was carried out with Student’s *t*-test.

## 5. Conclusions

Our genome-wide analysis of RWP-RKs in NFC with many crops (such as legumes) uncovered their evolutionary features (phylogeny, exon structure and protein motif) in detail. By expression and phylogenetic analysis of two legume crops (soybean and common bean) with *A. thaliana*, we found that *NLPs* with different origins play diverse regulatory roles in nodulation. Co-expression network analysis of *A. thaliana* under both the conditions of N-starvation and N-supplementation revealed the differential response of *AtNLP* genes to nitrogen and involved multiple biological processes (such as symbiosis) under N-starvation, which may provide the foundation for nodulation until the emergence of *NIN* within the NFC. This helped translate biological knowledge from a model dicot (*A. thaliana*) to nodulating NFC [40]. A further qRT-PCR analysis of *NLP* genes in *P. vulgaris* suggested the differential response of *NLP* genes during nodulation programs under different concentrations of nitrates. These results will provide new insight into the features of RWP-RKs in NFC, as well as the relationship of RPW-RKs between nodulating NFC species (*P. vulgaris* and *G. max*) and non-nodulating *A. thaliana*, which will be helpful to improve NUE from soil by the genetic improvement of *NLP*s.

## Supplementary Materials

The following are available online at https://www.mdpi.com/2223-7747/9/9/1178/s1. Figure S1: Protein sequences of the 292 RWP-RKs identified in 26 species. For convenience, the sequence identifiers were named using the standard format “Species_sequence_identifier”, such as “Arabidopsis_thaliana_AT2G17150.1”. Figure S2: Sequence alignment based on the conserved domains of the 292 RWP-RKs. 1–231 are RWP-RKs of nodulating plants, and 232–292 are RWP-RKs of non-nodulating plants. Figure S3: Sequence alignment based on the 156 conserved PB1 domains. 1–113 are PB1s of nodulating plants, and 114–150 are PB1s of non-nodulating plants. Figure S4: Distribution of 50 consensus enriched motifs in the phylogenetic tree. Figure S5: Gene structures and top 25 enriched conserved motifs of the 292 RWP-RKs. Figure S6: Density distribution plots (A) and Q-Q plots (B) for data obtained using rlog normalization method for expression data under KCl and KNO3 treatment in Arabidopsis thaliana. Figure S7: Correlation of expression levels (A) and connectivity (B) of genes between KCl- and KNO3-treated samples in Arabidopsis thaliana. Figure S8: Plant heights at the early nitrogen-prefixing stage 7th day after inoculation (top) and corresponding stage 7th day after uninoculation (bottom) (A) and at nitrogen-fixing stage 21st day after inoculation (top) and corresponding stage 21st day after uninoculation (B) under 0 mM, 5 mM, and 10 mM KNO3 treatment in Phaseolus vulgaris. Figure S9: Statistics of plant heights at nitrogen-fixing stage 21st day after inoculation (DAI) and corresponding uninoculation in Phaseolus vulgaris. Student’s t-test: *, *P* < 0.05; **, *P* < 0.01; ***, *P* < 0.001. Figure S10: Phylogenetic tree of the NLPs from Arabidopsis thaliana and Phaseolus vulgaris, and NRSYM1 from Lotus japonicus, constructed by the neighbor-joining method with 1000 bootstrap values. Table S1: Information (nodulation status, distribution of RWP-RK and PB domains, RKDs and NLPs, taxonomy) about selected species from the nitrogen-fixing clade and Arabidopsis thaliana. Table S2: Distribution of orthogroups of the 292 RWP-RKs at the genome-wide level. Table S3: Genomic features, physicochemical properties, and subcellular localization of proteins of the 292 RWP-RKs. Table S4: Distribution and statistics of exon numbers and intron phases of the 279 RWP-RKs. Table S5: Gene distribution of 50 consensus enriched motifs among the 292 RWP-RKs. Table S6: Gene ontology analysis of AtNLP-containing modules in the biological process category. Table S7: Pearson correlation coefficients between AtNLP3, AtNLP6, and AtNLP7 vs. genes from the symbiosis-related GO term under N-starvation. Table S8: Gene-specific primers for PvNLP genes in Phaseolus vulgaris.
