# Supplementary material for: Genome-Wide Identification, Characterization, and Regulation of RWP-RK Gene Family in the Nitrogen-Fixing Clade
Source: Plants (Basel). 2020 Sep 11;9(9):1178. doi: 10.3390/plants9091178 (PMC7569760; doi:10.3390/plants9091178)
Supplement: Supplementary file 1 [file plants-09-01178-s001.zip › Supplementary_data/FigureS1.pdf]

>Alnus_glutinosa_Alngl764S35691
MEDGVLPPVTMLDTLLDTATDFDFMDELFSDGCWLETTDGSEFLNQIPSSSGALLDPLLLWPALETNGSLSASP
SQKGNQDETRRPSPDESQERSLVNATSLGQNTITLAGCRSVSENNVIEGSEPGTRLWIAPRGNPGSTSSVTERFI
KAIGYIREVTIDKDVLIQIWVPVYKGGRRILTTINQPFSIGSSCPSIARYREISESFVFSAEKDSKYLVGLPGRVYLDK
VPEWTPDVRFFRSDEYPRVNHAQQYDICGTLALPIFEQGSRTCLGVIEVVMTKRHIKYHPELESVCKALEAVDLR
STDALSTEHVKQASDKIYQAALPEILEVLRSACETHRLPLAQTWVPCIQQGKEGCRHSDDNYLDCVSTVDHACI
VADHNMQGFHEACSEHHLLRGQGLVGRAFGTNQPCFSTDIRSFVKTEYPLAHHARVFGLRAAVAIHLRSIHTG
TADFVLEFFLPVDCTDPEEQKKMLSSLSVIVQQVCQGLRVVTDNELKEDSNLPVNGKPSGGEILHEKSSEIWQH
QHDSNLKRNAEFGEECSAYDEGSSPSLDMGIAGEKKRTKAEKTITLEVLKQYFAGSLKDAARSIGVCPTTLKRICR
HHGIKRWPSRKIKKVGHSLLKLQGVIDSVQGASGTLQIKSFYTNFPELASQNLAGTSPFSASKLSDHPQPSSMQ
PEGGILNPQAAASKSPSSSCTQSSSSSGTLQHPSTWNVAGFDDPMVGENLGDGVLKRVGSEAELQASSQGPN
LLARSWSHKSLNEHPNAESLRPLRKNSGWLSQEGNTLKVKVTYGDEKVRFRMQNTWKHKDLLLEIARRFNID
DVSRFDLKYLDDDAEWILLTCDDDLEECIDVCRSLQNQTIKLCLQVSHYHLS
>Alnus_glutinosa_Alngl4587S30137
MASQTLNGWWSKHDLVYTDDDPFVLFPTEMPSLDLSGYSSLDWQYDLPIQESFLDAVPLTESFPTDPLSAPVDI
LQPTTSVYLPFSFRLLTEQSLHCIAGDIFCGYGVWNEIGGGFEAENQALVLCNNGKKGMKEARVEGKMKRSREE
RCSSTSSLLSKKAISQYFYMPITQAAKELNVGLTLLKKRSRELGIRRWPHRKLMSLQTLIRNVKELGREEGEESEAK
LRDALEILEREKKMLEEFPDLQLEDNTKRLRQACFKANYKRRRLMGMESLSCSSSNRGSVDDVMANDQRMDE
EEEEEEEEEKSLLLDSFSSSSSIML
>Alnus_glutinosa_Alngl6212S05326
MADPSAIVPYHDPYDGPLAENILTFMDDANPTLADVSYSYPTLPPSEFDPLQDPVTWDICNESNNEAGTSQAG
LSEVRAGTRNTEGPYFESQSARDWNMRPLSVWPVAPIPYNCSCCQVLREIIYTNGTITKKLEIHGRLGMICHAIL
QNLQNVNVNSSSNQYQMFDFCKKSIEDVKQFLVNYCVEQKLAGYVMVQDPLAIFYEALCVGLDWDEDPLPDD
FFQLSPTNSEGARQMVMDDPDAENERERTPRVSLAAQGHGCRARSTAARGRVGQKARCCALRVAARGSSDR
RFLDVSRILSGRSMQRRCKRDDRTTGSMGSSAAARECGYDEGVEETDSNFKSVRRLQRERAGKLTLNEVGRYF
HLPIEEAARRMKLCPTVLKKICRRYGMNRWPHRKVKSIQRQISSLTASLDSNNAEARANAQAEIDRLQQELTNV
CAGVVSRGSPEEHNYALRSARPD
>Alnus_glutinosa_Alngl7222S05734
MENSVSFDGTRNLNLEDNFSELMNYDNYAVWCRSPGATDQIFTSYGLSSDPSMPYGLSLDAFNFTEQNTGAF
PVNEIGGTFNPIGTSFSPGDKMMVQQMDTQDGFPMDFNIANELADAQNNGSLQQNNVLDMENSLIPRPLS
SPVEEKMLKALSLFKDSSGAGILAQVWVPRRLGDQYLLTTCDQPYLLDHILTGYREVSRSFTFPAEKRTGYYLGLP
GRVFTSKVPEWTSNVGYYKKVEYLRVEHAVNHEVRGSIALPVFDSPGMSCCAVLELVTTKEKMNFDAEMEIVC
NALEAVNLRTTPPPRLRTQYLSKNQKAVLAEIIDVLRAVCHAHRLPLALTWIPSCYTKGDGDEITGVRVRESNAN
SGEKYILCIEETACYVNDRTMKGFVRACLEHHLEEGQGVAGKALQSNHPFFFPDVKSYDISEYPLVHHARKFGLN
AAVSIRLRSTYTGDDDYILEFFLPVNMSGSSEQQLLLNNLPGTMQRMCKSLRTVSDAELCGAEGSKVGFEKGGI
QNFLPMAMSRRNSQMTLSDSDVNSTEKMPLNVSNTKNDGFEADSPHEQVFFLIATSGSGRQEKKRSTAEKNV
SLSVLQQYFSGSLKDAAKSIGVCPTTLKRICRQHGISRWPSRKINKVNRSLRKIQTVLDSVEGVDGGLKFDPATGE
FVATDSVIQESDSQKCLLFSNKNLCVKNVKPVNEHAVSVPSVPGIDGLSSLIKLDADDCFMGGNQVGLSSSKLIT
NACGVELEKSRVSLIDSSEDSTLVAINAPWPSPENASFGSSLANRGEKWGLNNDSWKLQNPDSHFVPRSSGSL
ATADEMDSRVGGDDGIGERTQRTSSSLTDSSNASRSTMPGSSSSSMSLEEEKHSKFKSSCADSGSKIIVKATYGE
DTIRFKFEPSVGCFQLYEEVAKRFKMQNGTFQLKYLDDEDEWVMLVSDSDLQECLEILDDAGARSVKFLVRDKP
CTISSSGSSNCFLPVGS
>Alnus_glutinosa_Alngl10019S12742
MDSNSTHLNIPKIENPHGFDWFPEEQSEKRTLFCLFSVLGSFCRLLELPPLDSFFDFDSTLPFLHNTKQFGVTEFQ

DFEDPDSDFFWAESKLPFLYEDVVVDQKPLNSTTEFDTIGHNYGNLGSDTSESSMVVLSEYASSSSRSEEERRSTS
GRKKSAALELDEIQRHFNVPITKAAKEMNVGLTVLKKRCRELNIMRWPHRKIKSLKSLINNVKELGLTNEVVMLE
EHKRLLEILPDMQLTQRTKKLRQACFKANYKKRRSLTIQA
>Alnus_glutinosa_Alngl10067S12813
MNPNTNTASPNHSLSFDHISNYFALPLNDAASNLGVCTSVLKKICRDNGLDRWPYRKFMSGKSIEEIKRNAARE
RYKELAEVSKIGRESGSQQPNSDMSKSQGATLAHNLQQQGSKNIQNVRPQNLLGTSLTKGITILDEFKYGYPAD
GLSTASNKWWGSSGPDGCEHVFGDGAETNENEKQQSEKMVADSSDMVTVDKEKHEGGKEGCKIASQGMG
GLASVRKRSMEEGREALKLGVFRGYGINTIGKRERTLLLRIFKSSLPKRWIYGSSY
>Alnus_glutinosa_Alngl26612S40027
MAESEEPKSTEEEEEEEEEEEGVEALIMDFDNLDDSWSWDQILLLSDNNIDNISHNPMSPLSDQLFSPPWAFA
DGNDDAVHATSAPPDSSPLLSCEFHVYTWQCFNQICNEGRQNALAEILGILTVVCETHKLPLAQTWVPCRHRS
VLAQGGGLKKSCTSFDGGCMGQVCLSTTDVALYVVDAHMCGFREACVEHHLQKDQGVAGRAFSSHSLCFCE
NITLFCKTEYPLVHYARMFGLASCFAICLRSTHTGDDDYIVEFFLPPSITDFYEQRILLGSLLATMKQHFQSLKVASG
IELEDEVSVEIIQVSTNERVDSRLESLQIPLSMKSPPRPDGLLNKGDVVQLESSKQQLVLHLNDINDKGTIANNVG
ESVDHFSSLENKEIRKTSERKRGKTEKSLSLEVLQQYFAGSLKDAAKSLGVCPTTMKRICRQHGISRWPSRKINKV
NRSLCKLKHVIESVQGAEGAFDLNSLTTSPLAVAVDSQPSSLNRSNPQKSPSFKLSEPQKKNESPTGITLKNNEQA
GMEVLLQGRRKLSPKNLIRDRSSSPPELVRGSNRSKTRSGSREESAGTPTSHGSCQGSPPDGSTRAKHLYISSINE
QCNKVGGCPDLAYQPPGAPNISATYLIPDALLMTEPQETFGGMLIEDAGSSKDLRNLCPSVADAMLDEQVPEF
CWTNPPCPSLAPNQSMATLAHTMPHVTAKKEMNSVTIKATFRKVIIRFRLSLSSGILELKEEVAKRLKLEIGTFGIK
YLDDDNEWVLIACDSDLQECIDISRSSASNVLRLLVQDVMANLGSSCESYGE
>Alnus_glutinosa_Alngl46270S10821
MASQTLNGWWSKHDLVYTDDDPFVLFPTEMPSLDLSGYSSLDWQYDLPIQESFLDAVPLTESFPTDPLSAPVDI
LQPTTSVIQGDIFCGYGVWNEIGGGFEAENQALVLCNNGKKGMKEARVEGKMKRSREERCSSTSSLLSKKAISQ
YFYMPITQAAKELNVGLTLLKKRSRELGIRRWPHRKLMSLQTLIRNVKELGREEGEESEAKLRDALEILEREKKML
EEFPDLQLEDNTKRLRQACFKANYKRRRLMGMESLSCSSSNRGSVDDVMANDQRMDEEEEEEEEEEKSLLLD
SFSSSSSIML
>Alnus_glutinosa_Alngl55731S32319
MIFQLGCKMDGNPTKETCQQSDDTYHQSTKSMPALHQDLNCLPYPVAMSELSEDQQKEHCSPGIAESKKKRA
ASEHIARIALPDLAKYFDLPIVEASRNLKVGLTVLKKKCREFGIARWPHRKIKSLDSLIQDLQEEAKRQQQENEAA
AMAVAKRQRMLESEKENIERKPFIELKTETRRFRQDVFKRRHRARAHRNHGPSV
>Alnus_glutinosa_Alngl424567S27630
MEIERKLMNMEYGTASFTAPNSATYGNFSPTDHTAAMEMDFMDELLLEGCWLETTRALNDPSPYYLPTLDSNI
NHHQQIYQEDQTVGVFPESETIIVEGTELGRRWWIGPRANPGPSSSVKERLMLAVGYLRECTKNMNVLLQLW
VPVRRGGSRYFLTSQDQPYYRNVCRAYQFAVEEDMEESGGLPGRVFMGKLPDQWTPPDVRFFKRDEYPRINY
AQQYDVRGSLALPVFERGSGTCLGVVEIVTTTQKINYRPELEHVCQALEAVDLRSSQYFSPPGIKACHDELYPAAL
AEIIEVLATVCKTHRLPLALTWAPCYQQGKGGCRQYSDENYAQCLSIVDTACFVADVDVLGFHEACSEHHLFRG
QGTVGTAFTTAKPCFASDITAFSKTEYPLSHHARMFGLRAAVAIPLRSIYTGSADFVLEFFLPKDCQDTEEQKQML
NSLSIVLQQACRSLHAVIDKELPDEDEIIYPVKEVAIEECHKSGSSTLKEGSPKDQSSWIAHMMEAQQKGKGVSIS
LEYQEEEQNEEFKVTTHWDNINTQGGSCNGQAFSDFGQVQQSSGSKGSVEGGGDSYSYGSRRSSGGRKSGEK
RRTKTEKTISLPVLRQYFAGSLKDAAKSIGVCPTTLKRICRQHGITRWPSRKIKKVGHSLRKLQLVIDSVQGAEGAI
QIGSFYSNFPELSSPATSSFSSLKMSENSKQSNPIPESSGLFTPSKSPPSSCSQTSGPSIFCATAANHHHTTTINALS
SGDTLMRENPIGILQMACSEADLHALNQQEANLLQNAMITCGLDTTLPVLPESSSHNSRDGGAFRVKATFGDE
KIRFSLHPNWTFGDLQMEIRRRFNLDDISRVDLKYLDDDREWVLLTCDADFQECIDIYRASQSHTIRLSLQHASN
PNLGSPFGSSGLS

>Arabidopsis_thaliana_AT2G17150.1
MEDDGGSDGGEGNGGFSPNSSFGAFADTAMDLDFMDELLFDGCWLETTDSKSLKQTEQSPSASTAMNDNS
PFLCFGENPSQDNFSNEETERMFPQAEKFLLEEAEVGKSWWIAPSASEGPSSSVKERLLQAISGLNEAVQDKDF
LVQIWVPIQQEGKSFLTTWAQPHLFNQEYSSLAEYRHVSETYNFPADEGMKDFVGLPGRVFLQKFPEWTPDVR
FFRRDEYPRIKEAQKCDVRGSLALPVFERGSGTCLGVVEIVTTTQKMNYRQELEKMCKALEAVDLRSSSNLNTPS
SEFLQVYSDFYCAALPEIKDFLATICRSYDFPLALSWAPCARQGKVGSRHSDENFSECVSTIDSACSVPDEQSKSF
WEACSEHHLLQGEGIVGKAFEATKLFFVPEVATFSKTNYPLAHHAKISGLHAALAVPLKSKSGLVEFVLEFFFPKA
CLDTEAQQEMLKSLCVTLQQDFRSSNLFIKDLELEVVLPVRETMLFSENLLCGAETVESLTEIQMQESSWIAHMI
KANEKGKDVSLSWEYQKEDPKELSSGRENSQLDPVPNNVPLEAEQLQQASTPGLRVDIGPSTESASTGGGNML
SSRRPGEKKRAKTEKTIGLEVLRQYFAGSLKDAAKSIGVCPTTLKRICRQHGIMRWPSRKIKKVGHSLKKLQLVM
DSVQGAQGSIQLDSFYTSFPELNSPNMSSNGPSLKSNEQPSHLNAQTDNGIMAEENPRSPSSSCSKSSGSSNN
NENTGNILVAEDADAVLKRAHSEAQLHNVNQEETKCLARTQSHKTFKEPLVLDNSSPLTGSSNTSLRARGAIKVK
ATFGEARIRFTLLPSWGFAELKQEIARRFNIDDISWFDLKYLDDDKEWVLLTCEADLVECIDIYRLTQTHTIKISLNE
ASQVKLSGSFGNTGLS
>Arabidopsis_thaliana_AT2G43500.1
MENPFASREKGFGNYSDFPTEQMDGLSSNFGSGVRNLISDDMFNPSSELMNFDSLAAWCNSPSATDILFAQY
GLSNSQPMPFGAFTSFHVADPKATSLTRSFYDLESSYYGEERSSAQEMNSQFHRSSDSDELSGKRRKVVNQKIG
FPNVLNCTIPRSLSHSLDEKMLKALSLFMESSGSGEGILAQVWTPIKTGDQYLLSTCDQAYLLDPRFSQYREVSRR
FTFAAEANQCSFPGLPGRVFISGVPEWTSNVMYYKTDEYLRMKHAIDNEVRGSIAIPILEASGTSCCAVMELVTS
KEKPNFDMEMDSVCRALQAVNLRTAAIPRPQYLSSSQRDALAEIQDVLRTVCHAHKLPLALAWIPCRKDQSIRV
SGQKSGENCILCIEETACYVNDMEMEGFVHACLEHCLREKEGIVGKAFISNQPFFSSDVKAYDISEYPIVQHARK
YGLNAAVAIKLRSTYTGEDDYILELFLPVSMKGSLEQQLLLDSLSGTMQRICRTLRTVSEVGSTKKEGTKPGFRSSD
MSNFPQTTSSENFQTISLDSEFNSTRSMFSGMSSDKENSITVSQGTLEQDVSKARTPEKKKSTTEKNVSLSALQQ
HFSGSLKDAAKSLGGETSAYFQAWVYFFCPTTLKRICRQHGIMRWPSRKINKVNRSLRKIQTVLDSVQGVEGGL
KFDSATGEFIAVRPFIQEIDTQKGLSSLDNDAHARRSQEDMPDDTSFKLQEAKSVDNAIKLEEDTTMNQARPGS
FMEVNASGQPWAWMAKESGLNGSEGIKSVCNLSSVEISDGMDPTIRCSGSIVEPNQSMSCSISDSSNGSGAV
LRGSSSTSMEDWNQMRTHNSNSSESGSTTLIVKASYREDTVRFKFEPSVGCPQLYKEVGKRFKLQDGSFQLKYL
DDEEEWVMLVTDSDLQECLEILHGMGKHSVKFLVRDLSAPLGSSGGSNGYLGTGL
>Arabidopsis_thaliana_AT4G24020.1
MCEPDDNSARNGVTTQPSRSRELLMDVDDLDLDGSWPLDQIPYLSSSNRMISPIFVSSSSEQPCSPLWAFSDG
GGNGFHHATSGGDDEKISSVSGVPSFRLAEYPLFLPYSSPSAAENTTEKHNSFQFPSPLMSLVPPENTDNYCVIK
ERMTQALRYFKESTEQHVLAQVWAPVRKNGRDLLTTLGQPFVLNPNGNGLNQYRMISLTYMFSVDSESDVEL
GLPGRVFRQKLPEWTPNVQYYSSKEFSRLDHALHYNVRGTLALPVFNPSGQSCIGVVELIMTSEKIHYAPEVDK
VCKALEAVNLKSSEILDHQTTQICNESRQNALAEILEVLTVVCETHNLPLAQTWVPCQHGSVLANGGGLKKNCT
SFDGSCMGQICMSTTDMACYVVDAHVWGFRDACLEHHLQKGQGVAGRAFLNGGSCFCRDITKFCKTQYPLV
HYALMFKLTTCFAISLQSSYTGDDSYILEFFLPSSITDDQEQDLLLGSILVTMKEHFQSLRVASGVDFGEDDDKLSF
EIIQALPDKKVHSKIESIRVPFSGFKSNATETMLIPQPVVQSSDPVNEKINVATVNGVVKEKKKTEKKRGKTEKTIS
LDVLQQYFTGSLKDAAKSLGVCPTTMKRICRQHGISRWPSRKIKKVNRSITKLKRVIESVQGTDGGLDLTSMAVS
SIPWTHGQTSAQPLNSPNGSKPPELPNTNNSPNHWSSDHSPNEPNGSPELPPSNGHKRSRTVDESAGTPTSH
GSCDGNQLDEPKVPNQDPLFTVGGSPGLLFPPYSRDHDVSAASFAMPNRLLGSIDHFRGMLIEDAGSSKDLRN
LCPTAAFDDKFQDTNWMNNDNNSNNNLYAPPKEEAIANVACEPSGSEMRTVTIKASYKDDIIRFRISSGSGIME
LKDEVAKRLKVDAGTFDIKYLDDDNEWVLIACDADLQECLEIPRSSRTKIVRLLVHDVTTNLGSSCESTGEL
>Arabidopsis_thaliana_AT4G35270.1
MEGGRGGGDGNFLPNSNFGVFSDSAMDMDFMDELLFDGCWLETTDGKSLKQTMGQQVSDSTTMNDNN

NNSYLYGYQYAENLSQDHISNEETGRKFPPIPPGFLKIEDLSNQVPFDQSAVMSSAQAEKFLLEESEGGRRYWIA
PRTSQGPSSSVKERLVQAIEGLNEEVQDKDFLIQIWLPIQQEGKNFLTTSEQPHFFNPKYSSLKRYRDVSVAYNFL
ADEDSKESVGLPGRVFLKKLPEWTPDVRFFRSEEYPRIKEAEQCDVRGSLALPVFERGSGTCLGVVEIVTTTQKM
NYRPELDNICKALESVNLRSSRSLNPPSREFLQVYNEFYYAALPEVSEFLTLVCRVYDLPLALTWAPCARQGKVGS
RHSDENFSECVSTVDDACIVPDHQSRHFLEACSEHHLLQGEGIVGKAFNATKLFFVPEVTTFSKTNYPLAHHAKI
SGLHAALAVPLKNKFNSSVEFVLEFFFPKACLDTEAQQDMLKSLSATLQQDFRSLNLFIDKELELEVVFPVREEVV
FAENPLINAGTGEDMKPLPLEEISQEDSSWISHMIKANEKGKGVSLSWEYQKEEPKEEFMLTSGWDNNQIGSG
HNNFLSEAEQFQKVTNSGLRIDMDPSFESASFGVGQTLLGSRRPGEKRRTKTEKTIGLEVLRQYFAGSLKDAAKS
IGVCPTTLKRICRQHGITRWPSRKIKKVGHSLKKLQLVIDSVQGVQGSIQLDSFYTSFPELSSPHMSGTGTSFKNP
NAQTENGVSAQGTAAAPKSPPSSSCSHSSGSSTCCSTGANQSTNTGTTSNTVTTLMAENASAILKRARSEVRLH
TMNQDETKSLSRTLSHKTFSEHPLFENPPRLPENSSRKLKAGGASKVKATFGEAKVRFTLLPTWGFRELQHEIAR
RFNIDNIAPFDLKYLDDDKEWVLLTCEADLEECIDIYRSSQSRTIKISVHEASQVKLGGSFGSIGLGPSL
>Arabidopsis_thaliana_AT4G38340.1
MVGPFKKILHAHNQRFPPSSSSSLDPVVDDSSRKQTRIILSYLLHLSLSLHITYSLCLLHFFFGSSNPFSPMEESNNS
AVVDFPDNFMDQLLWEECWEEEATQHDQALSSPSGLKERVACAMGHLQEVMGERELLIQLWVPVETRSGRV
LSTEEQPYSINTFSQSQSLALYRDASAGYSFAAEVGSEQLVGLPGRVFLRRMPEWTPDVRFFRKEEYPRIGYARRY
QVRATLALPLFQGTSGNCVAVMEMVTTHRNLEYASQLSTICHALEAFDLRTSQTSIVPASLKVTSSSSSSSRTEVA
SILQGICSSHGLPLAVTWGHQDSSSCLSALISASYAADHGSRCFLAACSEHHLLGGEGIAGRAFATKKQCFATDVA
IFSKWSYPLSHYAKMFDLHAALAVPILTRGNRTVQFVLELFFPRDCLDIQTHSLTLASQLKLRFQSSPHLMVDDN
QIAEEVRDAATPPLTQEDPKGKQVSFSFSSASSLENRKRKTKAEKDITLDTLRQHFAGSLKDAAKNIGVCPTTLKRI
CRQNGISRWPSRKIKKVGHSLRKLQVVMDSVEGVQGSLHLASFYSSFPQLQSSSSSSFPFINPTQTVHVPPKSPP
SSSGSQSSSGSSTCCSSEEQQLGGFQKPALSHPQLLTLSSMHEDQRPVRVTSSLPPLPSATTPRKAKDGMKVKA
MFGDSMLRMSLLPHSRLTDLRREIAKRFGMDDVLRSNFSLKYLDDDQEWVLLTCDADLEECIQVYKSSSLKETIR
ILVHHPLSRPSFGS
>Arabidopsis_thaliana_AT4G35590.1
MVDQGFFTLKKEKKKNILIKLFYVKILGFCFCDSWSSSDMAHSLTSLAVFQSVIRKEMVRSLHVYESVEIEREFWF
KSKSCYVEKKAKPLFRSEDFRRPEISEGSVFGTWRCIFVFRFNHSLPRFPTLLCLSRNPKLEDIPNLANELKFISELK
PSKIYEEEQCSSSTEGYYNSDLPKPRKLVLKQDLNCLPDSETESEESVNEKTEHSEFENDKTEQSESDAKTEILKKK
KRTPSRHVAELSLEELSKYFDLTIVEASRNLKVGLTVLKKKCREFGIPRWPHRKIKSLDCLIHDLQREAEKQQEKNE
AAAMAVAKKQEKLETEKRNIVKRPFMEIGIETKKFRQENFKKRHRASRAKKNQESLVTSSST
>Arabidopsis_thaliana_AT1G74480.1
MADHTTKEQKSFSFLAHSPSFDHSSLSYPLFDWEEDLLALQENSGSQAFPFTTTSLPLPDLEPLSEDVLNSYSSAS
WNETEQNRGDGASSEKKRENGTVKETTKKRKINERHREHSVRIISDITTYTTSSAPTTLSKETVSRYFYMPITQAA
IALNVGLTLLKRRCRELGIRRWPHRKLMSLNTLISNVKELQKMEGEENAEKLQDALEMLEKEKRTIEDLPDLEFK
DKTKRLRQACFKANHKRKKKRSLKSDQSQVPSCSSSGSVPSDESVDEAGMESDEEMKYLLCGFSSEFTSGL
>Arabidopsis_thaliana_AT1G64530.1
MELDDLDLSGSWPLDQITFASNFKSPVIFSSSEQPFSPLWSFSETSGDVGGELYSAAVAPTRFTDYSVLLASSESET
TTKENNQVPSPSWGIMPLENPDSYCAIKAKMTQALRYFKESTGQQHVLAQVWAPVKNRGRYVLTTSGQPFVL
GPNSNGLNQYRMVSLTYMFSLDGERDGELGLPGRVFRKKLPEWTPNVQYYSSKEFSRLGHALHYNVQGTLAL
PVFEPSRQLCVGVVELIMTSPKINYAPEVEKVCKALEAVNLKTSEILNHETTQICNEGRQNALAEILEILTVVCETYK
LPLAQTWVPCRHRSVLAFGGGFKKSCSSFDGSCMGKVCMSTSDLAVYVVDAHVWGFRDACAEHHLQKGQG
VAGRAFQSGNLCFCRDVTRFCKTDYPLVHYARMFKLTSCFAVCLKSTYTGDDEYVLEFFLPPAITDKSEQDCLLGS
LLQTMKQHYSSLKVVSETELCENNMSLEVVEASEDGMVYSKLEPIRIHHPAQISKDYLELNAPEQKVSLNSDFM
ENNEVDDGVERFQTLDPIPEAKTVKKSERKRGKTEKTISLEVLQQYFAGSLKDAAKSLGVCPTTMKRICRQHGIS

RWPSRKINKVNRSLTRLKHVIDSVQGADGSLNLTSLSPRPWPHQIPPIDIQLAKNCPPTSTSPLSNLQDVKIENR
DAEDSAGSSTSRASCKVNPICETRFRLPTHNQEPSRQVALDDSDSSSKNMTNFWAHLTCQDTASPTILQHKLVSI
KATYREDIIRFKISPESVSITELKQQVAKRLKLETAAFELKYLDDDREWVSVSCDADLSECLDTSAAKANTLRLSVH
DVTFNFGSSCESSEETMMCL
>Arabidopsis_thaliana_AT1G18790.1
MKSFCKLEYDQVFGKENNSFSFLNHSSLYSHQSELANPFFELEDEMLPSATSSNCFTSASSFLALPDLEPISIVSHE
ADILSVYGSASWTAEETMFVSDFAKKSETTTTKKRRCREECFSSCSVSKTLSKETISLYFYMPITQAARELNIGLTLL
KKRCRELGIKRWPHRKLMSLQKLISNVKELEKMEGEENEDKLRNALEKLEKEKKTIEKLPDLKFEDKTKRLRQACF
KANHKRKRRSGMSTPITSSSSSASASSSSYSSVSGFER
>Arabidopsis_thaliana_AT1G76350.1
MENNSLPMDPAMDSSFMDGLLLEGCWLETTDASEFLNFSPSTSVAPFDPSSFMWSPTQDTSNSLSQMYGQD
CPERSSLEDQNQGRDLSTFNRRWWIGPSGHHGFSVMERLVQAVTHIKDFTSERGSLIQLWVPVDRGGKRVLT
TKEQPFSHDPMCQRLAHYREISENYQFSTEQEDSDSSSRDLVGLPGRVFLGKVPEWTPDVRFFKNEEYPRVQH
AQDCDVRGTLAIPVFEQGSQICLGVIEVVMTTQMVKLSPDLESICRALQAVDLRSTEIPIPPSLKGPDFSYQAALP
EIRNLLRCACETHKLPLAQTWVSCLKQSKTGCRHNDENYIHCVSTIDDACYVGDPTVREFHEACSEHHLLKGQG
VVGEAFLTNGPCFSSDVSSYKKSEYPLSHHATMFGLHGTVAIRLRCIHTGSVDFVLEFFLPKNCRDIEEQRKMLN
ALSTIMAHVPRSLRTVTQKELEEEGDSMVSEVIEKGVTLPKIENTTEVHQSISTPQNVGLVFDGGTTEMGELGSE
YGKGVSVNENNTFSSASGFNRVTEKKRTKAEKNITLDVLRQYFAGSLKDAAKSIGVCPTTLKRICRQHGIQRWPS
RKIKKVGHSLQKIQRVIDSVEGVSGHHLPIGSFYASFPNLAASPEASSLQQQSKITTFLSYSHSPPAKSPGSSCSHS
SSCSSETQVIKEDPTDKTRLVSRSFKETQTTHLSPSSQEDDFLRVKVSYEEEKIRFKMRNSHRLKDLLWEIAKRFSIE
DVSRYDLKYLDEDNEWVLLRCDDDVEECVDVCRSFPGQTIKLLLQLSSSYLPERSSVSGCLS
>Arabidopsis_thaliana_AT1G20640.1
MEDSFLQSENVVMDADFMDGLLLDGCWLETTDGSEFLNIAPSTSSVSPFDPTSFMWSPTQDTSALCTSGVVS
QMYGQDCVERSSLDEFQWNKRWWIGPGGGGSSVTERLVQAVEHIKDYTTARGSLIQLWVPVNRGGKRVLTT
KEQPFSHDPLCQRLANYREISVNYHFSAEQDDSKALAGLPGRVFLGKLPEWTPDVRFFKSEEYPRVHHAQDCD
VRGTLAIPVFEQGSKICLGVIEVVMTTEMVKLRPELESICRALQAVDLRSTELPIPPSLKGCDLSYKAALPEIRNLLR
CACETHKLPLAQTWVSCQQQNKSGCRHNDENYIHCVSTIDDACYVGDPTVREFHEACSEHHLLKGQGVAGQA
FLTNGPCFSSDVSNYKKSEYPLSHHANMYGLHGAVAIRLRCIHTGSADFVLEFFLPKDCDDLEEQRKMLNALSTI
MAHVPRSLRTVTDKELEEESEVIEREEIVTPKIENASELHGNSPWNASLEEIQRSNNTSNPQNLGLVFDGGDKP
NDGFGLKRGFDYTMDSNVNESSTFSSGGFSMMAEKKRTKADKTITLDVLRQYFAGSLKDAAKNIGVCPTTLKRI
CRQHGIQRWPSRKIKKVGHSLQKIQRVIDSVQGVSGPLPIGSFYANFPNLVSQSQEPSQQAKTTPPPPPPVQLA
KSPVSSYSHSSNSSQCCSSETQLNSGATTDPPSTDVGGALKKTSSEIELQSSSLDETILTLSSLENIPQGTNLLSSQD
DDFLRIKVSYGEEKIRLRMRNSRRLRDLLWEIGKRFSIEDMSRYDLKYLDEDNEWVLLTCDEDVEECVDVCRTTP
SHTIKLLLQASSHHFPERSSATEYSLWH
>Arabidopsis_thaliana_AT3G59580.1
MENPSASRDNKGFCFPDIPVEEMDGWVKNLISEEDMFSSSSTSELMNFESFASWCNSPSAADILFTQYGLSTS
QSIIPFGGLEGSYACEKRPLDCTSVPRSLSHSLDEKMLKALSLFMEFSGEGILAQFWTPIKTGDQYMLSTCDQAYL
LDSRLSGYREASRRFTFSAEANQCSYPGLPGRVFISGVPEWTSNVMYYKTAEYLRMKHALDNEVRGSIAIPVLEA
SGSSCCAVLELVTCREKPNFDVEMNSVCRALQAVNLQTSTIPRRQYLSSNQKEALAEIRDVLRAVCYAHRLPLAL
AWIPCSYSKGANDELVKVYGKNSKECSLLCIEETSCYVNDMEMEGFVNACLEHYLREGQGIVGKALISNKPSFSS
DVKTFDICEYPLVQHARKFGLNAAVATKLRSTFTGDNDYILEFFLPVSMKGSSEQQLLLDSLSGTMQRLCRTLKTV
SDAESIDGTEFGSRSVEMTNLPQATVSVGSFHTTFLDTDVNSTRSTFSNISSNKRNEMAGSQGTLQQEISGARR
LEKKKSSTEKNVSLNVLQQYFSGSLKDAAKSLGVCPTTLKRICRQHGIMRWPSRKINKVNRSLRKIQTVLDSVQG
VEGGLKFDSVTGEFVAVGPFIQEFGTQKSLSSHDEDALARSQGDMDEDVSVEPLEVKSHDGGGVKLEEDVETN

HQAGPGSLKKPWTWISKQSGLIYSDDTDIGKRSEEVNKDKEDLCVRRCLSSVALAGDGMNTRIERGNGTVEPN
HSISSSMSDSSNSSGAVLLGSSSASLEQNWNQIRTHNNSGESGSSSTLTVKATYREDTVRFKLDPYVVGCSQLYR
EVAKRFKLQEGAFQLKYLDDEEEWVMLVTDSDLHECFEILNGMRKHTVKFLVRDIPNTAMGSSAGSNGYLGTG
T
>Arabidopsis_thaliana_AT5G53040.1
MSSSKHSSVFNYSALFLSLFLQQMDQNSLHHLDSPKIENEYEPDSLYDMLDKLPPLDSLLDMEDLKPNAGLHFQ
FHYNSFEDFFENIEVDNTIPSDIHLLTQEPYFSSDSSSSSPLAIQNDGLISNVKVEKVTVKKKRNLKKKRQDKLEMS
EIKQFFDRPIMKAAKELNVGLTVLKKRCRELGIYRWPHRKLKSLNSLIKNLKNVGMEEEVKNLEEHRFLIEQEPDA
ELSDGTKKLRQACFKANYKRRKSLGDDYY
>Arabidopsis_thaliana_AT5G66990.1
MADQRPLMTWLEANNYESFLQEDIFSFLDQSLFVDPHSSFIDPFKDFQTQNWFSLQDSIVNHISTTFAADHTFL
ASLDLEAISSTFSLDISSGWWNENNGNYNNQVEPNLDEISRTNTMGDPNMEQILHEDVNTMKEKTSQKRIIM
KRRYREDGVINNMSREMMKQYFYMPITKAAKELNIGVTLLKKRCRELGIPRWPHRKLTSLNALIANLKDLLGNT
KGRTPKSKLRNALELLEMEKKMIEEVPDLEFGDKTKRLRQACFKAKYKRRRLFSSSS
>Arachis_duranensis_Aradu.N7C89
MASCNYFFNPQQIDEYPFPLPIQCSLYDYDYYYYSSENTNEYPPLGCWAHEFSIQDMECYPSLPFYETLSIEPISTI
KDYDFYDIKEGFSVWNEVDAGFDCSEKVSSSSSLSNNNNNEENKSIRRVINKRVCREERSSNSTKMLSRKMISD
YFYMPITQAAKELDVGLTLLKKRCRELGIRRWPHRKLMSLQTLINNVQELGKEEGRESEEKLRSAIEILEKEKKLLE
EMPDLQLEDNTKRLRQACFKANYKKRKLIMGSSSSNSSMESSSSYNIIGHNATRITSSKGDTMDSVGFISFEDEE
DQERDIKYALSLPPLSHNIQMV
>Arachis_duranensis_Aradu.R5YE9
MECYPSLPFYETLSIEPISTIKDYDFYDIKKGFSVWNEVDAGFDCSEKVSSSSLLSNKNNEEDKSIRRVMNKRVCR
EERSSNSTKMLSRKMISEYFYMPITQAAKELDVGLTLLKKRCRELGIRRWPHRKLMSLQTLINNVQELGKEEGRE
SEEKLRNAIEILEKEKKLLEEMPDLQLEDNTKRLRQACFKANYKKRKLMMGCTSSNSSMESSSSSSYHIIGHNAL
RTSQNNTMGGVEFISFDDDEYQERDIKSVLSLPSLSHNIQMCSSYMFNHPSDASFSSAIR
>Arachis_duranensis_Aradu.TG0QF
MKMNIGLTLFKRRCKELSINHWPHRKIKSLHLLVQNLKETALDNKSWSVGARNKDVGESSRNGTHIRDKETKAS
FLENWMPLKPSLTPSSSPISPTERSILTARKGVEGNREGISGGGKGVATTPRCRTTTPHRQTYPTVATNVDEKRW
LVSEKVAGLEPTSWRRGIGERQNSHLVAAVHGASSCITVTRRREKMLAGGAASSRHCCTTDRHRSIIALNETHK
EEPDVREEEGERSEADMVSATAVYLRHCRTAPLRCLVGASVTVAGDLWLLENTAIVATEGGRSIAAVLTARHSLVV
TRTTAGDATTWLSHFLLVVPPPLFHKHTRSPVKEKKERRARRGERDPEGGRGSPPRLAAGGRARSEAELRSNHR
VRSLSRYQHRRDLQPVCSVVGKLVAVGVSPSLSLL
>Arachis_duranensis_Aradu.I1BME
MLVFYFLIPSLTSLKRRCRELSINRWPHRKIKSLHLLLQNLILIIIGLINDRVKTDYFN
>Arachis_duranensis_Aradu.G4SB3
MEDQFFSEGEGIGYWTPPGAQFDGPSMIDNGIKNLVSEEMLDNLPDLMNFDNLAGWDCGNYNATETSLGF
DEKAVLQQLETQLGLSEDANDMNNINGSLRELSAADMGNCLVPRPFAWSLDERMLRAMDLFKESVGDGILTQ
VWAPMKHGNDFILSTSEQPYLLDHMLAGYRDVSRQFTFSAEDKPGSFPGLPGRVFNSQIPEWTSNVGYYNKS
EYLRVEHAINHEVRGSIAVPIIDTLAEPPCCAILELVTTKEKTDFDRELEIVSSALQLVNLRTTTPPRLMSQCLSNNK
RAALAEIIDVLRAVCHAHRLPLALTWIPCYYSEGIGDEASRIRIKDGGRIPGEKTVLCIEESACYINDIVVGGFVHAC
VEHYLEEGQGAAGKALQSNRPFFCPDVKAYNISEYPLVHHARKYKLNAAVAVRLRSIHTNDDDYIIEFFLPVNMN
GSEEQQLLLDNLSDTVQRMCQSLRIVSDAELHGIEGSQVQFSKEEGSHMAPDGDHDLVQPMSMTNNETEAA
HNQAMDGSRKQTEKKKSSVEKNFSSSVLQQYFSGSLKDAAKSLGVCPTTLKRICRQHGISRWPSRKINKVNRSL
KKIQTVLDSVKGVEGGLEFDPYKGGFMPGGSSNQEINAHKSSLFGKKCSVKYPDPASGSKDSKPIAMDDGGLR

QEPFPVSILESSWCDIAYHSPNTLVADEMADKLGEHHNPTTSSMSDSSNGSGSILRGSRKFEKRKHLKAKSPCV
DNGSKLVVKAAYGEDRIPGCLQLYEEVATRFKLQIGSFQLKYLDDDEEWVMIVTNSDLRECIEIFDDLGTRAVKFL
VSETCLVERTLRHDYASSKYLFSEDWLFSVRCLSNNKRAALTEIIDVLRAVCRAHSLPLALTWIPCYYSEGIGDEASR
IRTKEGRKLPGEKTVLCIEESACYMNDIVVGGFVRACVEHYLEEGQGAAGKALQSNRPFFCPDVKAYNISEYPLV
HHARKYKLNAAVAIRLRSTHTNDDDYIIEFFLPLNMNRCQEQQVLLENLSRTMQRMCQTLRKVSDAELYGIEGS
QVQFSKEEGSHMAPDGNHDSVQPMSMTNNETEAAHNQAMEGSRKQIEKKRSPVKKNVSLSVLQQYFSCSLK
DAAKSLGVCPTTLKRICRQHGIARWPSRKINKVNRSLKKIRTVLDSVQGVEGGLKFDPYKGGFMSVGSSNQEIN
AHKSSLFRKKCSVKYPDPASGSKDSKPIAMGDGGLRQEPFPVSILESFWCDIAYHSPNSLVADEMETYLNGADKL
GEHYNPTTSSMTDSSNGSCSILRSSSSASGKFENRKHLKAKSPCVDSGSKIIVKAAYGEDLIRFKFDPAAGCLQLYE
EVAARFKLQIGSFQLKYLDDEEEWVRMANDSDLEDCIEMLDDLDTRAVKLLVHDMPSGLSSLGSNG
>Arachis_duranensis_Aradu.BBG0S
MEDQFFSEGEGIGYWTPPGAQFDGPSKIDNGIKNLVSEEMLDNLPDLMNFDNLAGWGNIPSVTDHNLDNGI
SSLASMQYSPPDAFSLVEQDNDPYFMTKDCGNYNATETSLGFDEKAVLQQLETQLGLSEDANDINNINGSLQEL
SAADMGNCLVPRPFAWSLDERMLRAMDLFKESVGDGILTQVWAPMKHGNDFILSTSEQPYLLDHMLAGYRD
VSRQFTFSAEDKPGSFLGLPGRVFISQIPEWTSNVGYYNKSEYLRVEHAINHEVRGSIAVPIIDTLAEPPCCAVLEL
VTTKEKTDFDKELEIVSSALQLVNLRTTTPPRLMSQCLSNNKRAALTEIIDVLRAVCHAHRLPLALTWIPCYYSEGV
GDEASRIRIKDGGKIPGEKTVLCIEESACYVNDIVVGGFVHACVEHYLEEGQGVAGKALQSNRPFFCPDVKAYNI
SDYPLVHHARKYKLNAAVAVRLRSIHTNDDDYIIEFFLPVNMNGSEEQQLLLDNLSDTVQRMCQSLRSVSDAEL
YGVEGSQVQFSKEEGSHMAPDGDHDSVQPMSMTNNETEAAHNQAMDGSRKQTEKKKSSVEKNFSLSVLQQ
YFSGSLKDAAKSLGVCPTTLKRICRQHGISRWPSRKINKVNRSLKKIQTVLDSVKGVEGGLEFDPYKGGFMPGGS
SNQEINAHKSSLFGKKCSVKYPDPASGSKDSKPIAMDDGGLQQEPFPVSILESSWCDIAYHSPNTLVADEMADK
LGEHHNPTTSSMSDSSNGSGSILRGSRKFEKRKHLKAKSPCVGSGSKITVKAVYKEDMIRFKFDPAAGCLQLYEE
VATRFKLQIGSFQLKYLDDDEEWVMMVTDSDLKECIEILDDLGTRAVKFLVSETCLVV
>Arachis_duranensis_Aradu.0768L
MDSSLSILLVFNNTIQPELFRSVHVYCRELLDGDEREEEEVVVEREFVLSESGFYVEMKCTPILTSLKLCVDDVCEG
YKNGVWLCVFAFHADHTPQFTHIPSILLFARNSKLKAIPNLLNDLQIIYKLELKAEVKDDLLDSSKEESQGNSDGH
QSPQKVLPVFNQDLNCLPLDEDTTELIDKKTNVESLPGFGAKKRRAQSDHIAKIALPDLVKYFDVPIVEASRNLNI
GLTVLKRKCREFGIPRWPHRKIKSLDNLIHDLEEEAKHQEPKDKVSAMAMVRRQRMLECEKENMEKKPFMDI
QTETKRFRQDVFKRRHRARAVEKQSSAV
>Arachis_duranensis_Aradu.YRC2R
MPDSCEEKSELPSKSKPQEEHGFPMDFDIYLESSSWPMDHTPSASNPMSPFIITTSSEQPFSPLWAFSDVEDDH
RHVRVVAGDNTNTNTAIETENPVENDDNKKIVSPPFVPLPPIKIPDGYCLIKERMTQALRHFKQLTEQNFLAQV
WAPVRNGNRYALTTSGQPFVLDPHSNGLHQYRTVSLMYMFPVDGENDEILGLPGRVFQQKLPEWTPNVQYY
TSREYSRRNHAQHYNVRGTLALPVFESPGQSCVGVLELIMTSEKVNYAPEVDKVCKALEAVNLRSSEILEHPFAQ
ICNEGRQNALAEILEILTVVCETHNLPLAQTWVPCRHRSVLANGGGLKKSCSSFDGHCMGRVCMSASDVAFYV
IDAHTWGFHDACAEHHLQQGQGVAGRAFLSHNMSFCGNITQFCKTDYPLVHYALMFGLTSCFAICLQSSHTGS
DDYVLEFFLPPSITNFYEQKDLLGSILATMKQNFQSLKVAAGVELEEGCTIEVVEPINERIHLSLESVPVAQSAKSPP
PTLNASLNKDDGVPQGPLEQQMPAWLEDINDGGNLGDNAGGSMNPMTSLDAKIKKKPSERKRGKAEKMISL
EVLQRYFSGSLKDAAKSLGVCPTTMKRICRQHGISRWPSRKINKVNRSLSKLKCVIESVRGAEGAFALNSVNKDP
LPIAAGSFTEPCTSKMFNRNASLSIQPSKTRMNENDLDTSRVSETNRQVRMQDQLLEGEQSPEKVIHEEGWST
QEVGTKDPEKFRNLSGSSEDSANPHSHNSCHGSPPNEISPANIFRPFNKEKSVPLGVSTESTMQPTNALNYANA
YTALNVERTEPQEPFGRMLLEGVGSSKDLRNLCPLEDQALEACGVNPPCHDLAPKQCMMDTTLNSNNTMIPF
APKKEMKSVTIKATYKEDIIRFRVFLNCGIVELQEEISKRLKLEIGAFDIKYLDDDNEWVLISCDADLQECMDVLTS
SGSNMIRLVVHDTVSILGSSCESSGN

>Arachis_duranensis_Aradu.46M2Y
MEYGGIVHNENGAFGSLSESAVEAEVDLVDELLVEGCWVETTSAGGCYNWMHNPSDAVVAPQPQYLEMLQL
QLQEESSSSSSDQESLVGKRWWIGPTRPGPSSSSSSSSSVKERLVVAVGYLKDYTKNNNLLIQIWVPPRRSPLPLP
LPLPLDVLTFPNPSPNPNPNLCVRFLRSHEYSHHYQAQQHYDLRGSIALPVFERGTATCLGVLDILITNHLNLTNN
YRPQLDNVCNALEAVDFRSSHQNLNANFMPPAIKVYEELYQAALNEIVEVLTSVCKTQNLPLGLTWAPCIQQGK
CGCGHSSAMCVSTVDTACYVGDLEMLGFQEACSEYHLFKGQGIVGTAFTSAKPCFAIDITAFSRAEYPLSHLANI
FGLHAAVAIPLRSLYTGSADFVLEFFLPKDCHDTEQQKHMLNSLSMLVQQACRSLHVATEDDFTPPMLTHHQA
QAQIMEEACSKDQSWIAHMMEAQVQQKGKGVCVSLEYQMEEPKEEFKVTTNWDTANDQAHHQLFSSSDF
GHIDVLEHSRSRASTAVDGGEESYALGGRRSSSSGSGGRKSGDKRRTKAEKTISLPVLRQYFAGSLKDAAKSIGVC
PTTLKRICRQHGITRWPSRKIKKVGHSLKKLQLVIDSVQGAEGAIQIGSFYASFPELSSTDLSSRSESSPSKKTHSTTI
PHQNHNNNTFLKSPPSSACSQTLDVVMTESSEAALLLKRAHSEAAELLQHASLQSQEDTKHNRSLEMARRFNL
NDISISSSNIDLKYLDEDGEWVLLACDADLEECKDIHRSSQSRTIRLSLFQASPLNHLATNTFGNGTTSPS
>Arachis_duranensis_Aradu.7K2S3
MRDGGVLSSGTMLEPTPPPPPPPLPPPPAPPPTTTTSISVDYDYINELFVDGCWLAASSSAAAAADGSADFYVP
SPSFSNPIFDPFSWPCLDTEQIESQAREREQEHEKQEQPPPFSHDLVVAANSCSRNQQQQYHHYENQSVENN
NPNPNNPSEFFRRWWIAPSSNPGPGSYVVEKLLKALMCIKDVNRNKDMLIQIWIPVMNRGGTQILRTNGLPF
SLESRSVNLAKYREISEVYQFSAEEDSKELVPGLPGRVYKEKVPEWTPDVRFFRSYEYPRVDHAQVYDVRGSLAL
PIFEQGSKNCLGVVEVVMTQQKINYRPELESVCQALEAVNLTSSKLPTIQNVKVKMEKKKKKNHLRHTSCEKSYE
TALPEIQEVLRSACEIHKLPLAQTWIPCIKQGKEGCRHSEDNYPHCISPVEHACYVGDPSIQVFHEACSEHHLLKG
QGVAGGAFMTNQPCFAPDITLLSKTDYPLSHHARMFGLRAAVAIRLRSIYNSSDDFVLEFFLPLECIDNDEQKK
MLTSLSLIIQRVCHSLRVISDKELEEETDFSVEEVIAHEDSGTFASAAAWPEPLQSQIVASLGAQEKSSETMGTSFS
DQRQQQQESSVLKGNLDSNGECSTYNVGNLSSKTGDKKKSKVDKTITLQVLRQHFAGSLKDAAKNIGVCTTTL
KRICRQHGIKRWPSRKIKKVGHSLQKLQLVIDSVQGASGAFQIDSFYSKFPDLAASPNLSGTSLFSNLKQCDNNP
NSLSIQPDPGSLSPEGASKSPSSSCSQSSISSHPCSSMAEQQNHHLTNNNFDSSKDQMVLLVGENSSGDGLLKR
IRSEAELKSLNEDRVKIVMPRSQSQETLGQHNVQNGHHGSLSRTKSKGTQKEDAAYRVKVTYGDEKARFKMPK
NWGYEDLVQEVGRRFCISDMSKFDVKYLDDDYEWVLLTCDDDLEECIEVCQSSESTTIKLCLQLSNTNHSMRNP
LEFR
>Arachis_duranensis_Aradu.1V6B6
MEFFNNELDSFSNDIDKIMGISPMDDFDDWAPLLTYKSTQKLFLTDLPEVDNDFDDLPPLDDFKKESEEEDQEH
KQLLQLVPSSSTIKNKKSELEYDEIKKHFHLPITEAASKMNIGLTLLKRRCRELSIKRWPHRKIKSLHLLIQTLKETGL
DNEVEMLEEEINMLKQVPETEITQETKKLRQAYFKANYKRRKLLASNSS
>Arachis_duranensis_Aradu.H6JXR
MEPYPYSPKRSGASYWGSCRVQLEGSTSLDGDMSNSISDDMACSFSGLMNFDPYAGLYNSPSITDQVFNNGL
PSSYPSTPGFNLEENNSSQFFRTEMNGYFNTKQGSPGYGEKIAFQQMDTLLGFFDGTDDTHSLSSMQKINGSS
QHLDAIGSYIMSKPASLSLDERMLKALSVFKESAGGGILAQVWVPIKDGDQFFLTTSEQPYLLDQILAGYREVSR
TYTFSTEGKPGSLPGLPGRVFMSKTPEWTSNIGYYNKNEYLRMDDAINHEVRGSIGLPISDLHSELPCCAVLELV
TTNEKPDFEKEMDIVCHALQNVDLTANMPPRLLPQCLSNSKRAALTAIVDVLRSVCHAHRLPIALTWIPCCVAED
AEGKDTKIHIKEGHSSPREKNILCVEESACYVTDKAMEGFVHACIEHHLQEGKGIAGRAHQSNHPFFCPDVKAY
NISEYPLVHHARKYNLNAAVAIRLRSTYTNDDDYILEFFLPVNMKGSSEQQLLLDNLSGTMQKVCRNLRTVSDEE
LSRMKGSHVGFKNKNVPRIFPMSRRNSQIPLTNSNHDSVRKMSIKASNQGNNGIESSHNQERNGSRRMVETK
RPAEKKVSLSVLQQHFSGTLKDAARSIGVCPTTLKRICRKQGISRWPSRKIKKVNRSLKKIQTVLDTVEGLEGGLKF
DPCLGALVSREGESIIQEIDAHDTLLHPENVTAKEKWVLNKGGSREDKSKHSSILVNRDDDGAVEHNHPSSSGFT
NSSNDSGSNQRQNLKGKSTFADKTGLKLQAKATYGEDTVRFKFDPSWGCLKLYEEVATRFKLQNGSFQLKYLDN
EQELVRLLDDSDLQECMEILDEVGKSCVRFVVRDIPCNLSSPSCSSCYLGCDS

>Arachis_duranensis_Aradu.T4VLF
MSESEEDKTDFASLKSKEDQQQSPPQLPPPSAMDFDLDLETSWPLDHLSFVSNNPMSPFLFPISSEQPSSPLWL
FSDAEDERHNNTLASAPAFSDFHKIFSCDSNSVTEKPVENANDEDKKLLPPIVAMPPLEILDRYCVIKERMTQAL
RYYKELTEQNVLAQVWAPVRNGNRFVLTTSGQPFVLDPHSNGLHQYRTVSLMYVFSADGEKEESLGLPGRVYQ
QKVPEWTPDVQYYSTKEYPRRDHAQHYNVRGTLALPVFEPSMQSCVGVLELIMTSQKINYAPEVDKICRALEA
VNLKSSEILGHQYTQICNEGRQNALAEILEILTVVCETHNLPLAQTWVPCRHRSVLAHGGGLKKSCSSFDGSCM
GKVCMSTTDIAFYIIDAHLWGFREACVEHHLQQGQGVAGRAFSSHSMSFCRNITRFCKIDYPLVHYALMFGLTS
SFSICLRSSHTGDDDYVLEFFLPPRITDFNEQKALLGSILTIMKQHFQSLKIASGVELEQNALVETIEATIEGVHLRFE
SIPVRQDASPNVREELAQDPSLQKIMMGCNDGGSIGDQIPSLETKNTNKPSERKRGKTEKSISLEVLQRYFAGSL
KDAAKSLGVCPTTMKRICRQHGISRWPSRKINKVNRSLSKLKRVIESVQGAEGAFALNPLSTSPLPFPEHSTPNK
FSQQASPTEPQIRENELDASKVLETTRIARAQCLEKMVNDKSGSIREVGKETKGPRAKSCSSADSTNPTSHGSCH
GSPPIESSPVKDIFITSNNDQCVGLRSPEATMQPPNNTLSYPTTCTMPDMVATELQEPFGGMLVEDAGSSKDLR
NLCPSVAEAIVEDMAPEPCRTNPPFSGLAPKQCMDPLKETVTPFAARIEMKTVTIKATYREDIIRFRVSLNCGIVE
LKEEVAKRLKLEVGTFDIKYLDDDHEWVLIACDADLQECIDVSRSSASNIIRVLVHEITSNLGSSCESSG
>Arachis_duranensis_Aradu.TED1R
MEDQFFSEGEGIGYWTPPGAQFDGPSMIDNGIKNLVSEEMLDNLPDLMNFDNLAGWVEQDNDPYFMTKD
CGNYNATETSLGFDEKAVLQQLETQLGLSEDANDINNINGSLQELSAADMGNCLVPRPFAWSLDERMLRAMD
LFKESVGDGILTQLVNLRTTTPPRLMSQCLSNNKRAALTEIIDVLRALCHAYSLPLALTWIPCCYTEGVGDEASRIRI
KDGGRIPGEKTVLCIEESACYINDIVVGGFVHACVEHYLEEGQGVAGKALQSNRPFFYSDVKAYNISAYPLVHHA
RKYKLNAAVAVRLRSIHTNDDDYIIEFFLPVNMNGSEEQQLLLNNLSDTMQRMCQSLRTVSDAELYGIEGSQVQ
FSKEEGSHMAPDGDHDSVMPMSLTNNETEAAHNQAMDGSRKQIEKKKSPAEKNISLSVLQQYFSGSLKDAAK
SLGVCPTTLKKICRQHGISRWPSRKINKVNRSLKKIQTVLDSVKGVEGGLKFDPYKGGFMSGGSSNQEINAHKSS
LFRKKCSVKYPDPAAGSKDSKPIAMDDGGLRQEPFPVSILESSWCDIAYHSPNTLVADEMADKLGEHHNPTTSS
MSDSSNGFGSILRGSRKFENRKHLKAKSPCVDNGSKLVVKAAYGEDRIRFKFDPAAGCLQLYEEVATRFKLQIGSF
QLKYLDDDEEWVRMVNDSDLKECMEILDDHGTRAVKFLVLDIPSGLSSRGSNT
>Arachis_duranensis_Aradu.W5BH1
MDNPYFVVPYHDPYDTPPLSPSFFNISNPTLLDLTNVSSGTESRHSQQKSNGIQEANLQPTFDPMIQDNNSVG
GTGGNNFESIPLLQVHSEEGPSHDFNNDVNTITLSHWPLPPVPFSCSCCSILREIVHTNGFNFTKVEIHGRPGLIS
HAIQHPTVNGDFVSTGNLQYQMIDFCRRSIDDVKNFLVEYCRVQSAAGYFMLQDPLSAYYEALCTGLEWVEEF
SDDTDDLNASNSDDENEQVADNGVGASGKAPRRLSLSEQRDRAGKMKLDDFAPYFHFPIEEASRQMNICPTV
VKKICRKEGLSRWPYRKIKSTVRQISQLKRTMESKDERSKARIQAEIYWLHLKLREICAGRYPSGIVIP
>Arachis_ipaensis_Araip.W1UKI
MFNLYFSGNSNEYPPLECWAHEFSIQDMECYPSLPFYETLSVEPISTIKDYDFYDIKKGFSVWNEVDAGFDCSEK
VVSSSSSLSNNNNEEDKRIRRVMNKRVCREVRSSNSTKMLSRKMISEYFYMPITQAAKELDVGLTLLKKRCRELG
IRRWPHRKLMSLQTLINNVQELGKDEGTESEEKLRNAIEILEKEKKLLEEMPDLQLEDNTKRLRQACFKANYKKR
KLIMGSSSSNSSMESSSSYNIIGHNATRITSCKGGTMDSIGFISFEDEEDQERDIKSVLSLPPLSHNN
>Arachis_ipaensis_Araip.377BK
MDLKTSYSDWLFSVQCLSNNKRAALTEIIDVLRAVCHAHSLPLALTWIPCYYSEGIGDEASRIRIKDGRKIPGEKTV
LCIEESASYINDIVVGGFVHACVKHYLEEGQGVAGKALQSNRPFFYPDVKAYNLYTMQFFLPVNMNRSQEQQL
LLDNLSGTMQRKCQTLRKVSDAELYGIEGSSFSCMSLTNNETETAHNQDMEGSRKQIEKKRSPVKKNVSLSVLQ
QYFSGSLKDAAKSLVCPTTRKRICRQHGIARWPSRKINKVNRLKKIQTVLNSVQGVEGGLKFDPYTGGLMSGGS
INQEINARKSSLFQKKCSVKHPDPAAKDAVTISPAFGSKGKNLAVKFEDDNDNDVCLLGNQLLHSDMLISNSKG
ELKKNKAFSVGVDSKPIAKDDGGLCQEPFPVSILESSRCDVAYHSPNSLVADEMETYLNGADKLGEHHNPTTSS
MTDSSNGNLRIGNI

>Arachis_ipaensis_Araip.KR88K
MLDSSKEESQENSDGHQSSQEVLPVFNQDLNCRPLDENTTELIDNKTNVESLPGFGAKKRRAQSDHIAKIALPD
LVEYFDVPIVEALRNLNIGLTVLKKKCR
>Arachis_ipaensis_Araip.6M6N8
MDVVERCDEESDNAENEYVDNAWKEMSKDKGKGKKGLKRKSISECFYMPIAEASRHLKVGLIHLKRRWRDLGI
QRWPHRKLMSLQTLINNLQQQQQQQKGVDEDESSRIRSAIHILEREKSLLEEIPHLQLETTPKG
>Arachis_ipaensis_Araip.5C6JK
MEDQFFSEGEGIGCWTPPGVQFDGPSMIDNGIKNLVSEEMLDNLPDLMNFDNLAGWGNIPSVTDHNLDNG
ISSLASMPYSPHDAFSLVEQANDPYFMTKDCGNYNAIETSLGFDEKVVLQQLETQLGLSEDANDMNNINGSLQ
ELSAADMGNCLVPRPFAWSLDEKILRAMNLFKESVGDGILTQVWAPMKHGNDFILSTSEQPYLLDHMLAGYR
DVSREFTFSAEDKPGSFPGLPGRVFMSQIPEWTSNVGYYNKSEYMRVEHAINHEVRGSIAVPIIDTLAEPPCCAV
LELVTTKEKPDFDKELEIVSSALQLVNLRTTTPPRLMSQCLSNNKRAALTGIIDVLRAVCHAHSLPLALTWIPCYHS
EGIGDEASRIRIKDGVKIPGEKTVLCIEESASYINDIVVGGFVHACVEHYLEEGQGVAGKALQSNRPFFYSDVKAY
NISEYPLVHHARKYKLNAAVAVRLRSTHTNDDDYIIEFFLPVNMNGSQEQQLLLNNLSDTMQRMCQSLRTVSD
AELYGIEGSQVQFSKEEGSSFCSMSRGNSHMAPDGDHDSVQPMSLTNNETEAAHNQAMDGSRKQIEKKKSP
VEKNVSLSVLQQYFSGSLKDAAKSLGGKTTTALSICPTTLKRICRQHGIARWPSRKINKVNRSLKKIQTVLDSVQG
VEGGLKFDPYKGGFMPGGSSNQEINAHKSSLFRKKCSGKYPDPASKDAVTVSPASGGKDSKPIGMDDGGLRQE
PFLVSILESSRCDIAYHSPNTLVADEMENYLNGADKLGEHHNPTTSSMSDSSNGSGSRKFENRKHLKAKSPCVD
KGSKFIVKAAYKEDMIRFKFDPAAGCLQLYEEVATRFSTMKRNG
>Arachis_ipaensis_Araip.P1CBC
MSQCLSNNKRAALTEIIDVSRAVCRAHSLPLALTWIPCYYSEGIGDEASRIRIKDGCKIPGEKTVLCIEESACYINDI
VVGGFVRACVEHYLEEGQGVAGKALQSNRPFFYSDVKAYNISEYPLVHHARKYKLNAAVAVRLRSIHTNDDDYII
EFFLPVIMNGSQEQQLLLDNLSDTMQRMCQSLRIVSDDELYGIEGSQVQFSKEEGSSFFSMSRGNSHMAPEG
DHDSVQPMSLTNNETEAAHNQAMDGSRKQTEKKKSTVEKNVSLSVLQQYFSGSLKDAAKSLGVCPTTLKRICR
QHGIARWPSRKINKVNRSLKKIQTVLDSVQGVEGGLKFDPYKGGFIPGGSSNQEINAHKSSLFRKKCSGKYPDP
ASKDAVTVSPASGSKDSKPIGMDDGGLRQEPFPVSILESSRCDIVYHSPNTLVADEMENYLNGADKLGEHHNPT
TSSMSDSSNGSGSRKFENRKHLKAKSPCVDKGSKIIVKAAYKEDMIRFKFDPAAGCLQLYEQVATRFKLQIGSFQL
KYLDDEEEWVRMVNDSDLEECIEILDDLGTRAVKFLVSDMPSGLSSSGSNS
>Arachis_ipaensis_Araip.YB35N
MEDQFFSEGEGIGYWTPPGAQFDGPSMIDNGIKNLKIPGEKTVLCIEESACYINDIVVGGFVRACVKHYLEEGQ
GVAGKALQSNRPFFYSDVKAYNISEYPIVHHARKYKLNAAVAVRLRSIHTNDDDYIIEFFLPVIMNGSQEQQLLLD
NLSDTMQRMCQSLRIVSDDELYGIEGSQVQFSKEEGSSFFSMYRGNSHMAPEGDHDSVQPMSLTNNETEAA
HNQAMDGSRKQTEKKKSTVEKNVSLSVLQQYFSGSLKDAAKSLGVCPTTLKRICRQHGIARWPSRKINKVNHS
KPIGMDDGGLRQEPFPVSILESSRCDIAYHSPNTLVANEMENYLNGADKLGEHHNPATSSMSDSEGIGDEASRI
RTKDGRKIPGEKTVLCIEESACYTNDIVVGGFVRACVEHYLEEGQGVAGKALQSNRPFFYPDVKAYNISEYPLVH
HARKYKLNAAVAIRLRSIHTNDDDYIIEFFLPVNMNGSEEQQLLLDNLAGTMQRKCQTLRKVSDAELHGIEGSSF
CSMSRGNSHMAPDGDHDSVQPMSVTNNETEAAHNQAMEGSRKQIEKKRSPVKKNFSLSVLQQYFSGNLKD
AAKSLGVCPTTLKRICRQHGIARWPSRKINKVNRSLKKIQTVLDSGVEGGLKFDPYKGGFMSGGSSNQEINAHK
SSLFRKKCSVKYPDPAAKDAVTISPASGSKGENLAVNQLLHSDILISNSKGELKKNKAFSVDVDSKPIAMGDGGLR
QEPFPVSILESSRCDVAYHSPNSLVADEMETYLNGADKLGEHHNPTTSSMTDSSSGSCSILRPSSSGSPKFENRK
HLKAKSPCVRRGSKIIVKAAYGENIIRFKFDPAAGCLQLYEEVATRFKLQIGSFQLEYLDDEDEWVRMVTDTDLEE
CIEMFDDLGTAVKFLVREPCSNS
>Arachis_ipaensis_Araip.38X68
MEYGGIVHNENGGFGSLSESAVEAEVDLVDELLVEGCWVETTSAGGCYNWMHNPSDAVVAPQPQYLEMLQL

QLQEESSSSSSDQESLVGKRWWIGPTRPGPSSSSSSSSSSSVKERLVVAVGYLKDYTKNNNLLIQIWVPPRRSPLP
LPLPLPLDVLAFPNPSPNPNPNLCVRFLRSHEYSHHYQAQQHYDLRGSIALPVFERGTATCLGVLDILITNHLNLT
NNYRPQLDNVCNALEAVDFRSSHQNLNANFIPPAIKVYEELYQAALNEIVEVLTSVCKTQNLPLGLTWAPCIQQG
KCGCGHSSAMCVSTVDTACYVGDLEMLGFQEACSEYHLFKGQGIVGTAFTSAKPCFAIDITAFSRAEYPLSHLAN
IFGLHAAVAIPLRSLYTGSADFVLEFFLPKDCHDTEQQKHMLNSLSMLVQQACRSLHVATDDDFTPPMLTHHQA
QAQIMEEACSKDQSWIAHMMEAQVQQKGKGVCVSLEYQMEEPKEEFKVTTNWDTTNDQAHHQLFSSSDF
GHIDVLEHSRSRASTAVDGGEESYALGGRRSSSSGSGGRKSGDKRRTKAEKTISLPVLRQYFAGSLKDAAKSIGVC
PTTLKRICRQHGITRWPSRKIKKVGHSLKKLQLVIDSVQGAEGAIQIGSFYASFPELSSTDLSSRSESSPSKKTHTTN
IPHHQNPNNNTFLKSPPSSACSQTLDVVMTESSEAALLLNRAHSEAAELLQHASLQSQEDTKHNSFISSRSNNK
SHHHHSQDRDKERGAFRVKATFGDEKIRFSLQPNWGFRDLQLEMARRFNLNDISISSSNIDLKYLDEDGEWVL
LACDADLEECKDIHRSSQSRTIRLSLFQASPLNHLATNTFGNGTTSPS
>Arachis_ipaensis_Araip.VC558
MDSSLSILLVFNNTIQPELFRSVHVYCRELLDGDEREEEEVVVEREFVLSESGFYVEMKCTPILTSLKLCVDDVCEG
YKNGVWLCVFAFHADHTPQFTHIPSILLFARNSKLKAIPNLLNDLQIIYKLELKAEVKDDLPDSSKEESQGNSDGH
QSPQKVLPVFNQDLNCLPLDEDTTELIDKKTNVESLPGFGAKKRRAQSDHIAKIALPDLVKYFDVPIVEASRNLNI
GLTVLKRKCREFGIPRWPHRKIKSLDNLIHDLEEEAKHQEPKDKVAAMAMVRRQRMLECEKENMEKKPFMDI
QTETKRFRQDVFKRRHRARAVEKQSSAV
>Arachis_ipaensis_Araip.R44NW
MPDSCEEKSELPSKSKPQEEHGFPMDFDIYLESSSWPMDHTPSASNPMSPFIITTSSEQPFSPLWAFSDVEDDH
RHVRVVAGDNTNTAIETENPVENDDNKKIVSPPFVPLPPIKIPDGYCLIKERMTQALRHFKQLTEQNFLAQVWA
PVRNGNRYALTTSGQPFVLDPHSNGLHQYRTVSLMYMFPVDGENDEILGLPGRVFQQKLPEWTPNVQYYTSR
EYSRRNHAQHYNVRGTLALPVFESPGQSCVGVLELIMTSEKVNYAPEVDKVCKALEAVNLRSSEILEHPFAQICN
EGRQNALAEILEILTVVCETHNLPLAQTWVPCRHRSVLANGGGLKKSCSSFDGHCMGRVCMSASDVAFYVIDA
HTWGFHDACAEHHLQQGQGVAGRAFLSHNMSFCGNITQFCKTDYPLVHYALMFGLTSCFAICLQSSHTGSDD
YVLEFFLPPSITNFYEQKDLLGSILATMKQNFQSLKVAAGVELEEGCTIEVVEPINERIHLSLESVPVAQSAKSPQPT
LNASLNKDDGVPQGPLEQQMPAWLDDINDGGNLGDNAGGSTNLMTSLEAKIKKKPSERKRGKAEKMISLEVL
QRYFSGSLKDAAKSLGVCPTTMKRICRQHGISRWPSRKINKVNRSLSKLKCVIESVQGAEGAFALNSVNKDPLPI
AAGSFTEPCTSKMFNRNASLSIQPSKTQINENDLDTSRVSETNRQVRMQDQFLEGEQSPEKVIHEEGWSTQEV
GTKDPEKFRNLSGSSEDSANPHSHDSCHGSPPNEISPANIFRPFNKEKSVPLRVSAESTMQPTNALNYANAYTAL
NVERTEPQEPFGRMLLEGVGSSKDLRNLCPLEDQALEACGVNPPCHDLAPKQCMMDITLNSNNTMIPFATKK
EMKSVTIKATYKEDIIRFRVSLNCGIVELQEEISKRLKLEIGAFDIKYLDDDNEWVLISCDADLQECMDVLTSSGSN
MIRLVVHDTVSILGSSCESSGN
>Arachis_ipaensis_Araip.GND3E
MDFDYINELFVDGCWLAASSSSAAAAADGSADFYVSSPSFSNPIFDPFSWPSLDTEQIESQEREQEHEQQQEQ
PPFSHDLVVAANNCSQNQQQQYHHYENQSVENNNPNPNNPSEFFRRWWIAPSSNPGPGSYVVEKLLKALM
CIKDVNRNKDMLIQIWIPVINRGGTQILRTNGLPFSLESSSVNLAKYREISEVYQFSAEEDSKELVPGLPGRVYKEK
VPEWTPDVRFFRSYEYPRVDHAQVYDVRGSLALPIFEQGSKNCLGVVEVVMTQQKINYRPELESVCQALEAVN
LTSSKLPTIQNVKRTSCEKSYETALPEIQEVLRSACEIHKLPLAQTWIPCIKQGKEGCRHSEDNYPHCISPVEHACY
VGDPSIQVFHEACSEHHLLKGQGVAGGAFMTNQPCFAPDITLLSKTDYPLSHHARMFGLRAAVAIRLRSIYNSS
DDFVLEFFLPLECIDNDEQKKMLTSLSLIIQRVCHSLRVISDKEVEEETDFSAEVIAHEDSGTFASAAAWPEPLQSQ
IVASLGAQEKSSETMGTSFSDQRQQQQESSVLKGNLDSNGECSTYNVGNLSSKTGDKKKSKVDKTITLQVLRQH
FAGSLKDAAKNIGVCTTTLKRICRQHGIKRWPSRKIKKVGHSLQKLQLVIDSVQGASGAFQIDSFYSKFPDLAASP
NLSGTSLFSNLKQCDNNPNSLSIQPDPGSLSPEGASKSPSSSCSQSSISSHPCSSMAEQQNHHHTNNNFDSSKD
QMVLLVGENSSGDGLLKRIRSEAELKSLNEDRAKVVMPRSQSQETLGQHNLKNGHHGSLSRTKSKGTQKEDAP

YRVKVTYGDEKARFKMPKNWGYEDLVQEVGRRFCISDMNKFDLKYLDDDYEWVLLTCDDDLEECIEVCQSSES
TTIKLCLQFSNTNHSMRNPLEFR
>Arachis_ipaensis_Araip.RLH2R
MEPYPYSPKRSGASYWGSCRVQLEGSTSLDGDMSNSISDDMACSFSGLMNIDPYAGLYNSPSITDQVFSNGLP
SSYPSTPGFNLEENNSSQFFMTEMNGYFNTKQGSPGYGEKIAFQQMDPLLGFFDGTDDTHSLSSMQKINGSS
QHLDAIGSYIMSKPASLSLDERMLKALSVFKESAGGGILAQVWVPIKDGDQFFLTTSEQPYLLDQILAGYREVSR
TYTFSTEGKPGSLPGLPGRVFMSKTPEWTSNIGYYNKNEYLRMDDAINHEVRGSIGLPISDLHSELPCCAVLELV
TTNEKPDFEKEMDIVCHALQNVDLMANMPPRLLPQCLSNSKRAALTAIVDVLRSVCHAHRLPMALTWIPCCV
AEDAEGKDTKIHIKEGHSSPREKNILCVEESACYVTDKAMEGFVHACIEHHLQEGKGIAGRAHQSNHPFFCPDV
KAYNISEYPLVHHARKYNLNAAVAIRLRSTYTNDDDYILEFFLPVNMKGSSEQQLLLDNLSGTMQKVCRNLRTVS
DEELSRMKSSQVGFKNKNVPRIFPMSRRNSQIPLTNSNHDSVRKMSIKASNQGNNGIESSHNQERNRSRRMV
ETKRPAEKKVSLSVLQQHFSGTLKDAARSIGVCPTTLKRICRKQGISRWPSRKIKKVNRSLKKIQTVLDTVEGLEG
GLKFDPCIGALVSREGESIIQEIDAHDTLLHPENVTAKEKWILNKGGSREDKSKHSSILVNRDDDGAVEHNHPSSS
GFTNSSNDSGSNQRQNLKGKSTFADKTGLKLQAKATYGEDTVRFKFDPSWGCLKLYEEVATRFKLQNGSFQLKY
LDDEQELVRLLDDSDLQECIEILDEAGKSCVRFVVRDIPCNLSSSSCSSCYLGCDS
>Arachis_ipaensis_Araip.YWB61
MKQHFQSLKIASGVELEQNALVETIEATIEGVHLRFESIPVRQDASPNVREELAQDPSLQKIMMEGVLSKSCRLR
WCNQLSTLVEHRAFTLEEDDTIIRAHARFGNKWATIAPLLSARTDNAIKNRWNSTLKRKCASMGPIDDPHFAQP
LKRFVRTSVAVPVSTVVVRYSLLTVMAKTEEEGQMDYESTRRDFAGKRECGGKESEEGSVVVDDEGKREKEKEE
GVGRVDGLWRRRRAQKMEGEREDGLVVVVAMFAPTTMKRICRQHGISHWPSRKINKVNLSLSKLKRIIESVQ
GVEGAFALNPLSTKQSVSRKWLMIKEVGKETKGPRAKSCSSADSTNPTSHGSPPIESSPVKDIFITSNNDQCVGL
RSPEATMQLPNNTLSYPTTCAIPDMVATKLQEPFGGMLVKDAGSSKEFCS
>Arachis_ipaensis_Araip.Y4AFN
MSESEEDKTDFASPKSKEEQQQQSPPQLPPPSAMDFDLDLETSWPLDHLSFVSNNPMSPFLFPISSEQPSSPL
WLFSDAEDERHNNTLASAPAFSDFHKIFSCDSNSVTEKPVENANDEDKKLLPPIVAMPPLEILDRYCVIKERMTQ
ALRYFKELTEQNVLAQVWAPVRNGNRFVLTTSGQPFVLDPHSNGLHQYRTVSLMYVFSADGEKEESLGLPGRV
YQQKVPEWTPDVQYYSTKEYPRRDHAQHYNVRGTLALPVFEPSMQSCVGVLELIMTSQKINYAPEVDKICRAL
EAVNLKSSEILGHQYTQICNEGRQNALAEILEILTVVCETHNLPLAQTWVPCRHRSVLAHGGGLKKSCSSFDGSC
MGKVCMSTTDIAFYIIDAHLWGFREACVEHHLQQGQGVAGRAFSSHSMSFCRNITRFCKIDYPLVHYALMFGL
TSSFSICLRSSHTGDDDYVLEFFLPPRITDFNEQKALLGSILTIMKQHFQSLKIASGVELEQNALVETIEATIEGVHLR
FESIPVRQDASPNVREELAQDPSLQKIMMGCNDGGSIGDQIPSLETKNTNKPSERKRGKTEKSISLEVLQRYFAG
SLKDAAKSLGVCPTTMKRICRQHGISRWPSRKINKVNRSLSKLKRVIESVQGAEGAFTLNPLSTSPLPFPEHSTPN
KFSQQASPTEPQIRENELDASKVLETTRIARAQCLEKMVNDKSGSIREVGKETKGPRAKSCSSADSTNPTSHGSC
HGSPPIESSPVKDIFITSNNDQCVGLRSPEATMQPPNNTLSYPTTCAMPDMVATELQEPFGGMLVEDAGSSKD
LRNLCPSVAEAIVEDMAPEPCRTNPPFSGLAPKQCMDPLKETVTPFASRKEMKTVTIKATYREDIIRFRVSLNCGI
VELKEEVAKRLKLEVGTFDIKYLDDDHEWVLIACDADLQECIDVSRSSASNIIRVLVHEITSHLGSSCESSGE
>Arachis_ipaensis_Araip.80XBW
MTRRSCTCKQTPKPRLLSRCFSLLSPQRHCSCFVPNSDGSIFNLAMKNTLLTTLLVFKNTIREELIRSVHVYQIKDG
KVREVEREFVFSESGSYGEMRSTPILRLQKKLCVAEVSEGYQNGVWLCIFSFHRDHKPQFSSIPNLLLLSRNLKLR
TIPTLLRDLRVIYKLEKDKDVLSDSTGEERQGNNKDSQPLRKVVPVLTQDLNFLPYEDDMSESPDNKLDVQALP
DADSAEKKKRASSDRVAKITLSELVKYFDIPIVEASRRLNVGLTVLKRKCREFGIPRWPHRKIKSLDSLIHEIQEEAN
NQESDDKAAALAAIEKRRMLESEKENIERKPFMDIQSETKKLRQDIFKRRHRARAQVKLNSTVSSSSSSNKTTIET
>Arachis_ipaensis_Araip.J80SY
MDNPCFVVPYHDPYDTPPLSPSFFNISNPTLLDLTNVASGNESSHSQQKNNIIQDGNLQPTFDPMIQDSNFVG

GTGGNNFESIPLLQVHSEEGLSHDFNNNVNTITLSHWPLPPVPFSCSCCSVLREIVHTNGFNFKKVEIHGRPGLI
SHAIQHPSVNGDYTSTSNLQYQMIDFCRRGIDDVKNFLVEYCRVQSAAGYFMLQDPLSAYYEALCTGLEWVEEF
SDDTDDLNASNSDDENEQVADNGVGASGKAPRRLSLSEQRDRAGKMKLDDFAPYFHFPIEEASRQMNICPTV
VKKICRKEGLSRWPYRKIKSTVRQISQLKRTMESKDVRSKARIQAEIYRLHLKLREICAGRYPSGLVIP
>Arachis_ipaensis_Araip.73BCB
MEDQFFSEGEGIGYWTPPGAQFDGPSMIDNGIKNLVSEEKLDNLPDLMNFDNLAGWGNIPSVTDHNLDNGI
SSLASMPYSPPDAYSLVEQANDPYFMTKDCGNYNAIETSLGFDEKAVLQQLETQLGLSEDANDMNNINGSLQE
LSAADMGNCLVPRPFAWSLDEKMLRAMNLFKESVGDGILTQVWAPMKHGNDFILSTSEQPYLLDHMLAGYR
DVSREFTFSAEDKPGSFPGLPGRVFMSQIPEWTSNVGYYNKSEYLRVEHAINHEVRGSIAVPIIDTLAEPPCCAVL
ELVTTKEKPDFDKELEIVSSALQLVNLRTTTSPRLMSQCLSNNKRAALTEIIDVLRAVCHAHSLPLALTWIPCYYSE
GIGDEASRIRIKDGGKIPGEKTVLCIEESACYINDIVVGGFVHACVEHYLEEGQGVAGKALQLNRPFFYSDVKAYN
ISEYPLVHHARKYKLNAAVAVRLRSIHTNDDDYMIEFFLPVNMNGSQEQQLLLDNLSGTMQRMCQSLRTVSD
AKLYGIEGSQVQFSKEEGSSFCSMSRGNSHMAPDGDHDSVQPMSVTNNETEAAHNQAMDGSRKQIEKKKSP
VEKNVSLSVLQQYFSGSLKDAAKSLGVCPTTLKRICRQHGIARWPSRKINKVNHSLKKIQTVLDSVQGVEGGLKF
DPYKGGFMPGGSSNQEINAHKSSLFWKKCSGKFPDPASKDAVTVSPASGSKDSKPIAMDDGGLRQEPFRVSIL
ESSRCDIAYHSPNTLGEHHNPTTSSMSDSSNGSGSRKFENRKHLKAKSPCLDKGSKISVKAAYKEDMIRFKFDPA
AGCLQLYEEVATRFKLRIGSFQLKYLDDEDEWMMMVNDSDVEECIEILDDLGTRAVKFLVKHGSKIIVKAAYKED
MIRFKFDPAAGCLQLYEQVATRFKLQIGSFQLKYLDDEEKWVMMVNDSDLEEFIEISDDLRTRAVKFLVSDLVV
>Begonia_fuchsioides_Begfu1S23960
MSHWETSSGDLENLTPLDGGTGSLSFEDFYGNFSSLMSFDTDSGWCYGGTTDHMLCSSGFSSDLSMPSTSLD
PLNFVEDNREPFAMTESSSITSSLGTTYNGGVNSMFQHLDSVFRFEIDSKGVNDSESKQKDVVDHGSYIIPRPFG
WSLDERMLKALSLFKESSGGGILAQVWVPMKQANQYVLTTSKQPYLLDQMLMGFREVSRMFTFSIEGKPGSG
LGLPGRVFASRIPEWTSNVSYYKKGEYLRVEHAIGHEVRGSIALPVHDCHSEIPCCAVVELVTNKEKRNFDVEME
VVCQALQAVNLRTSLPPRLLTQCLTNDQKSALAEITDVLRAVCYAHRLPLALTWIPYCSNTEADNGFTRVHVNDS
IIGPKEKRILCIKETASYVNDRTMQDFVSACANHNLQEGQGVVGNALQSIHPVFNPDVKTYDMNEYPLVHHVR
KFGLNAAVAIRLRSTYTGDDDYILELFLPVNMTGSSEQQLLLNNLSGTMQRICKSLRTVSNVNLVCSEHPKVGIQ
AETVQKLAPSFISKSPSRLSDSLKTIEKQKLSNSANDGVETKLSSMKTSKSSKHTEKKRSTGEKNVSLSVLQQHFS
GSLKDAAKSIGVCPTTLKRMCRQHGILRWPSRKIKKVNRSLRKIQTVLDSVHGVDGGLKFDPSTGGFIATATAME
EFNDQSCFLLPNKKLPTDEVPAFKDSMSVPQEVSVKVEEDECFLARKSMILSLRIPNNESNAMPIYISDDSKCSSS
LAGDVGEADDEVYNNPRSSATNSSSGSQSVVHGSALCESHSFDGRNQPKDRISCVESGSKITVKASYLEDTVRF
KFDTTLKYFQLCEDIAKRFKLFGGTFEIKFLDDEKEWVMLASDSDLQECLEIMDDFGSQTVKLLVRDRAFGGSSS
AGSSCFLSGG
>Begonia_fuchsioides_Begfu255S10103
MADPGDEAPYNDPYDELNADDVLVFMSDQNPSLADLANVAEPSSSYPPMLEDGRGSGQSNNQFHDPRRWE
WVHHQSIAGGSSGAEPSNADPGPLNVGVGPSNFGGDNITPVPENVEPQSPADDVSWQVLVHPISPAPFFCRV
NIRKLEIHGRLGMISHAIYEDRPILSADSSNTQSQMFDFCKKSLGEIKKFFIQYNVNNGIDRFFALPEALAFFYDTL
CVGMEWKQKRSNRNAEPPPPPPPPATLPTFPLSAPSTTTSSVAAASTSHAGKSARRTASRATFVSLEIVNRTFDV
IGGAVQRQRTGKMTLGDMRAYFHLPISEASRRLRLCPTVVKKICRQSGLLRTLTPPLPPLSHLTVADQEMTTRLR
KNRKKRGHVSAGHGRIGKHRKHPGGRGNAGGMHHHRILFDKYHPGYFGKVGMRYFHKLRNKFHCPIVNIEK
LWSMVPQEVKDKASKDNVPLIDVTQFGYFKVLGKGVLPNDQPIVVKAKLVSKIAEKKIKENGGAVVLTA
>Begonia_fuchsioides_Begfu745S40752
MADGIFSAVTVLETSSGSVMEFDYMDELQLLECCWFETADGSEFLPITPSISSSLFEPYSFGWSVPVINGDSSSIP
PRNNDQNHSVSTMNQKTVDDSGSLNDKCLIENSELQKRRSLSPRSFPGQSPVMEKLIKAVNYIQEFVSEKDVLI
QVWVPVNTGGRLVLTTTDLPFSVNAACSRLAKYRDISMRYQFSAEKDSEDVVGLPSRVFLRKVPEWTPDVTFF

RRDEYPRVTFAQQCDVRGTLALPVFERGNKSCLAVIEVVRTSRQIKYVQELESVCKALEAVDLRSSDALGAPNSK
LSDCFSYGEVELFVLAEIHSVLRFACETHRLPLAQTWAPCVQQRRQGCRHSDENYVRCVSTVDRACFVADPSLG
PFHEACSEYHLLKGQGVVGEAFTTHKPCFSSDVKSYGNTEYPLSHHARMFNLWAAIAIRLRSFHAGKADFVLEF
FLPVTCTDHEEQRIMVSSLLIIIQHSIRSLRLVTDKELENEARRPFAIPEIPQVQPGDDDFTVVGDDSRQKYMGEN
SFSLKRNKQDSSLKEAVDGSECFNFGEGSLLAVGGGGKPGEKKRIKAEKAITLQVLRQHFAGSLKDAAKSIGVCP
TTLKRICRQHGIKRWPSRKIKKVGHSLQKIQRVIDSVEGASGTFRIGSLYSNFPEIASPALSEKSSVLASKIIDCPKTS
TFQGAASKSGSSSCSHGSSSSLSCSSRSLQLPPSCQYADRSSRENSGDGELKRDKSEAELHVPSQEEMIFTRSQS
QKTLGEHLGPENSLRRVKDSGRASEEDDSIRAKVTYGAEKIRLKMRKNQGFRELLDEICRRFQLSDTSKFDLKYL
DDDSEWVLLTCDADLEECIDVYKQSESLNQTIKLSLTLSRTRNSLGSYDLSQL
>Begonia_fuchsioides_Begfu719S50618
MEHPSSPNEKGMRCLETSRGQLETFMPLDVNMGNLGFEDDYGNFSNLSNLNSSAEWCYSPTPDPVSHYGLSL
VSSLPCSSLNPSNLVEDNNEAFSMTDISGVTYSMGSSFHQLESVFRFSIDSSDVNNSESKQNNVMDTSNYVISR
PFGWSLDERMLRALTLFKESSNEGTLAQVWVPMKHGNQYVLTASEQLYLLDQMLMGFREVSRMFTFSTEEKP
GAVLGLPGRVFASKIPEWTSNVSYYKKEEYLRVEHALGHDVRGSIALPVFDFQSEMFCCAVLELVTNKEKPNFDL
EMELVCQALRAVNLTTATPPRLLPHSLTNNQKSALAEITNVLRAMCHAHRLPLALTWIPCCYARGLDDASTRVH
VRDSTIGPHEKLIICIEETACYVNDKTMQDFLTVCTNHYLEEGQGIVGKAVQSNQPFFNHDVQAYNINEYPLVHH
VRKFGLKAAVAIRLRSTYTGIDDYILELFLPVDITGRNEQKLLLNSLSRTMQRVCRSLRTVSNVDLVCSERSKVGFP
NETVQKLTPISFRNLESSTSDSLSAKEKLKLSKSVNEDIETICSSDPTSGSQRQLGKKRSTAEKNVSLSILQQYFSGSL
KDAAKNIGVCPTTLKRICRQHGISRWPSRKINKVNRSLRKIQTVLDSVQGVEGGLKFDPSTGGFIATPAPMEEFN
SQSNFLLPKKKPHCGEEPALNNLLSVPRVCFNDHNSSIKLEDNEFLMAGDDILPSTSISPRKFGVPRVDLSGDSK
NSIKIFGGLFRNNYSNSHFESQSSSSLAADEMDTIGEADDGVVEHNHPLSSSTGDSSSGSRTLLHGSSSGSQSFE
GQKYPKYKICCVDSGSKITVKASYGEDTIRFKFDPSWGYFQLNEEVAKRFKLPSGSLELKYLDDEKEWVMLGCDS
DLHECLELMEDLGARTVKFLVRDRPFAVSSSAGSCCLLSGD
>Begonia_fuchsioides_Begfu734S05472
MKKEGPSSVSSRSRNEGYSFAVPMLVPQLDFSFPAVDWPVQENHVDAAALGNICQSDLYSSINIDLVGIPNDFS
YDCGDVGGVWSVIDEELGFQEKLIFGEDGGMEMKGREEKKGIKISRDEKNSCSKMLSRSTISQYFYMPITQAAK
ELNVGLTLLKKRCRELGIRRWPHRKLMSLQTLIKNVEDLSKDDGVETEAKLRDAVELLKRERLMMEETPDLQLE
DNTKRLRQACFKANYKKRKIMGMVESQASSPSTTYLDSIEVVEFEEDEELKTLLSQRSFPSSSGFVF
>Begonia_fuchsioides_Begfu1129S16439
MEDAIFSPAPMTGALVEYDMDFSYIDELLFGGCWFETTDGSEFLSSSNSNSFFEPSFRDPAIDTNCGPNTNIDLA
KTSVFGEYSENRAFPGSELGRRWWIGPRANPGLGSSVMDRLIKAVEYINHLERDKDVLIQVWIPENRGGRRVL
RTSELAFSMTSSSERLAKYRDISENYQFSADENSKEGLGLPSRVFLGKIPEWSPDVQFFRSDEYARVDFAQQCGV
RGTLALPVFDRGSRTCVGVIEVVMTTQQIKYSSELEIVCKALKAVDLRSSNAPETPNSTVFKASYEALFQEIRQVL
GFACETHKLPLAQTWISCSEQGNVGCRHSDENYIDCVSTVEEACYVADPFFTAFHEACAEHHLLKGQGIAGEAF
ETNGPCFSSDVKSFGNTKYPLSHHARMFGLQAAVAIRLRSIQSAKTDFVLEFFLPVDCFDVVEQRNLLSSLSLLM
QERFRSLRVITNKELDEEADLRQQRGTDNHSNGETCRQEVFKEQKGVFTENSSGFSECTRDGSNLIEDSFSNAS
MGRRAEKRRTKGEKTISLQTLQKYFAGSLKDAAKSLGVCPTTLKRICRQHGIKRWPSRKIKKVGHSLQKLQVVID
SVKDTSGSFQIDSLYSNFSELGSPKTGGIISPPSVKVNDNPQTTTSKSLSSSCSRSSGSSNCYSSRTKPRHLRLNSST
SKDPMVGNNSGGYQMKRARSEANLSTPNEGRTILPRSKSHRNLVGCGEDLLCPSKNSKKLTEDKDSRRIKVTYG
NEKIRFRMHKEWRWKELVDQIERRFRIQDITGFDVKYLDDDFEWVLLTCDADFEECLYVYRLAKRSTIKLSIQLSR
HCSK
>Begonia_fuchsioides_Begfu1351S18529
MEEAIFSPATMAGALVEYDMDFGYIDELLLGGCWLETTDGSDFLGSSNSSSLFEPSFRWAAMDTNGGLNTNA
PRKDCVSSPLFEAPERHFAEASDHGEQSENCTFQGSELGRRWWIGPRANPGAGSSVMDRLVKAVGYINDRER

DKDVLIQVWLPENRGGRRVLRTSELAFSMNSCSERLAKYRDISVSYQFSAEENSEEGVGLPSRVFLGKAPEWSP
DVQFFRSDEYARVDFAQQCGVRGTLALPVFDRGSGTCVGVIEVVMTTQQIKYSSELEIVCRALEAVDLRSSNVL
GTTKAKVYKASYEALFPEIREVLGLACQAHKLPLAQTWISCSEQGNMGGCHSEESYIDCVSTVEEACYVSDPFFK
AFHEACSEHHLLKGQGVAGEAFVTNGPRFSSDIRSFGNTKYPLSHHARMFGLQAAVAIRLRSIQSAKTDFVLEFF
LPLDCIDVADQRNLLTSLSLLMQEHFQSLRVITEKELREEVNSQRSNDGACRQEGLNGQKGSHPDQTAYQHPS
PTSYFTNGGHGSLFSPEKPGEVFTENSSGHDTNLEGFSECTLDGSTLVEDSLSNAGMGRRVEKRRVKGEKTISLQ
TLQKYFAGSLKDAAKSLGVCPTTLKRICRQHGIKRWPSRKIKKVGHSLQKLQVVIDSVKDTSGSFQIDSFYTNFSE
LGSPNMGGIISPAPTKGNDNPQTTTSASLSSSCSHSSSSSHCYSSRTKQQRLKWNNSISSNPRAGDDSGGFQM
KRVRSEVHLQTILPRSKSQRNHANTTEDRLQLAEDYQRVKVTYGSNKIRFRMRKEWRWKELIGEIGRRFNIPDIS
TFDIKYLDDDFEWVLLTCDADLEECLYVYRLAQRATIKLSIQLARRSSRWQ
>Begonia_fuchsioides_Begfu1417S19338
MADGIFSAVTVLDSFSDSAMEFDYMDELLLECCWFETADGSEFLPVNPSISSSLFEPYSIEWSIPEINENSSSIPLS
NNDQSYRSSTMNQKTVDDGESLNDKCSIENSEQQKRWSLSPRSFPGQSSVMEKLNKAVKYIKEFVKDKDVLIQ
VWVPIYTDGRLVLTTNNLPFSVNDGCSRLAKYRDISMRYQFSAEKDSKDVVGLPSRVFLRQVPEWTPDVTFFR
QDEYPRVSFAQQCDVRGTIALPVFERGNKACLAVIEVVRTCRQIKYGQELDSICKALEAVNLRSSDALGAPISKLS
DSFSYGEVEFFVLAEIHSVLRFACETHRLPLAQTWAPCVQQRRQGCRHSDENYVFCVSTVDRACFVADPNVGP
FHEACSEYHLLKGQGVVGEAFTTNKPCFSGDVKLYGNTEYPLSHHARMFNLSAAIAVRLRSFHAGKSDFVLEFFL
PVTCVDHEEQRSMMSSLLIVIQHSIRSLRLVTDKEFENEIPQVQPADDRFAVDGDDSRRKSMAANSLSSKHNKQ
DSILKEAVDGNDCFNFGEGSSLAPVGGGGKTGEKKRTKAEKAITLQVLRQHFAGSLKDAAKSIGVCPTTLKRICR
QHGIKRWPSRKIKKVGHSLQKLQRVIDSVEGASGTFQIGSLYSNFPEIASPTLASKIIDCPKAATVQGGSSKSGSSS
CSHGSSSSLSCSSRSLQFPSSWQNAGNENPNGGENSGDGEPKRDKSEAELNVSSQEETIFTRSQSQKTHSELH
GPENSLRGVQDKASEEDNSFRAKITYGDEKIRLKLRKNQGFRELLDEIGRRFHLSDTSKFDLKYLDDDSEWVLLTC
DADLEECIDVYKQSESPNQTIKLSLQLSRSRNSLGRYDLSQQ
>Begonia_fuchsioides_Begfu1777S22537
MDDGILSPAAMQGASDSAMDLDYMDELLLAGCWLEATDGSEFLNQTPPYSSTPLDPSLSGWSALDVNGDLST
VPPPNSDQNLGNEKGKLSALTANQKNTDDFDDELRRRWWIAPGPASSVRERLMKAVGYIKDFVRDKDVLIQV
WVPVNTEGRTVLTTTGLPYSVNTSCSRLSSYGDISMKYQFSAEKDSKDMVGLPSRVFIRKVPEWTPDVTFFRSD
EYPRVSFAQQCDVRGTLALPVFEQGNKSCLGVIEVVMTSRQIKYGEELQSVCKALEAVDLRSYDALGSPNAKVF
YRSNQDLLLEIQGISRFACESHSLPLAQTWASCAQQGREGCRHSDENYILCVSTVDQACFVADSSVKPFHEACSE
HHLLKGQGVVGEAFTTNKPCFMSDIRSYGNTVYPLSHHARMFDLQAAVAIRLRSIHARKADFVLEFFLPTSCTD
PEEQKKILLSLSIIIQHSCRNLRVVTDKEFKEEGYHPFNAGIASDNKTGIHEIPSVQIYDNACSIFSQDNEIKTVDEK
SSGRRQNKQDSSFKEAVEGNEECINFGEGSSSTFGIGKTGEKRRTKAEKAITLQVLRQYFAGSLKDAAKSLGVCPT
TLKRICRQHGIKRWPSRKIKKVGHSLEKLKRVIDSVEGGSGAFQLGSLYSNFPELASPNVSRTSPLLASKMNDCPK
TSPVQLEGSIVNLQAPASKSPSSSCSQGSSSSLCCSSKSLQFPIPCNEGGSEDPNAGENVGIGELKRVKSEAELHV
SSRGGLDIPRSKSFRGLGEHWGVENFQREAKDIRITSEEDDFLRLKVTYGDEKIRLRMHKNQGYRELLNEIGSRF
QIIDTSKFDLKYLDDDSEWVLLTCDADLEECMDVYRQSSSRSSTRGRTIKLSLQLARVRNSLGSHDLSQL
>Begonia_fuchsioides_Begfu1981S47656
MADPGDEVPYNDPYDRLNADDILVFMSDQNPSLADLTNVAEPSSSYPPVLEDGRGRGESDDKFHDPRRWEW
VHRQSIAGGSSGAGPSNADPGPSNAGAGPSNFGGDTSTPLLENVEPQSPVDNVSWQVLVHPISPAPFFCSCCL
VLRQIIHTNGVNIRKLEIHGRLGMISHAIYEDRPILSADSSNTLSQMFDFCEKSLDEIKKFFIQYNVSNGIDRFFALP
EALAYFYDTLCVGMEWEEKKSNRNAQPPPPPPPPPPPPPPPPYPPPPFQPLLQLPPPSQPPPPPPPGNPPGERA
SERRSSRSRSLTGPLTSLAAQRQRTGRMTLDDMRAYFHLPISEASRRLRLCPTVVKKICRKSGLRRWPHRKLVSY
HRKVSILQPNLNSSDPEVKERAIVEIERLRDEMTLIYAGVNLN
>Begonia_fuchsioides_Begfu2183S02159

MVSPWALDQISPILITSSDQPCSPLWAFSDGEEVGGGKLHAHPRLVHGRIRDNHLGITNLNLATEKPTKNIETSLL
QLPPLCGLKSTENPDGFSSIKEKMTQALRYLKELTDQYVLAQVWAPVKSGDRYVLTTLEQPFVLDAQSNGLHQY
RMVSLMYMFSVDGETNGVLGLPGRVFRQKSPEWTPNVQFYSSKEYPRKSHALHYNVQGTLALPVFESSGQSC
IGVLELIMNSPKINYAPEVDKVCKALEAVNLKSSEILDYPNAQICNEGRQNALAEILEVLTIVCEIHKLPLAQTWVP
CKHGSVLANGGGLKKSCSSFDGSCRGQVCMSATDIASYIVDAHAWGFRDACLEHHLLKGQGVAGRAFLSQSS
CFSVDITKFCKTEYPLVHYARMFELKSCFAVCLRSGFTGDDDYILEFFLPFVVNEYYEQLSLLGAILATMKQHFCTL
KVASGIKFEEGEIFVEIIEASTDDGLKSRFEYIRIPQSDPAPNGGEMFGFRLTELRLPADCDGDENKAGPESKTGNP
SPPKEARKRSERKRGKTEKSISLEVLQQYFTGSLKNAAKSLGVCPTTMKRICRQHGISRWPSRKINKVNRSLSKLK
RVIESVQVAEGAFGLTNSPPISPHGTNHLISPIDKPSDNQGENKELSSWMGDGFVGGTLRFDEYIHDKSGPEPK
LCKGSNESRTKSGSREESAGGSPTSHGSCQGSPANHNIPAKDPLVFTNLEQPIAGVESTGKLTPAALYFVPDLLPA
TQVEEPFGGMLIEDAGSSKDLRNLCPSAADAALDERIPESYWPHCAGSASAQFPDISGMRRETKRMTIKAMYK
EDIIRFRVVMSSGLVELKEEVGKRLKLEVGTFDIKYIDDDQEWVLIACDADLQECVEISKSSSGSNMIRLLVHDLTG
NLGSSCESTGE
>Begonia_fuchsioides_Begfu2529S27430
MESGGRFNAQKVEHPNDFDWIYNGEQPLFRNEFEFPPLETCYDLEFFPMPLHLNSLPQFEFQDFSDFIGGNFF
SPEIDCCISSEEKPVEAMSRSFNERCKDVNEGLRQVSGEERWGFSKTTKSWGDEGRRKSESLGLEEIQKQFHLPI
TMAAKKLKVGVTLLKKKCRELRIMRWPHRKLKSLDSLINNVKEMGMAREMMMLEEHRKMVEEEPDMQLSE
RTKRLRQASFKAKYKKRRSQFLCNQV
>Begonia_fuchsioides_Begfu2529S48380
MGSHWVRSWSENQAIASNEYCSFAFPCLFPQFDFSSHAYDGTYDRPFQDIYSEAATLASDFQSDNLYSSLNIEPI
PSKQNDREFSYNYGELCGFWSAINEDLGFRENLEFCENGGMEMREREEKKGIKRNKEGKSGSSKTLSRATICQY
FYMPITQAAKELNVGSTLLKKRCRELGIRRWPHRKLMSLETLIRNVQDLSKKDGDESESKLRDAVEILKKERTLM
EEMPDLQLEDETKRLRQACFKANYKKRKIMRMVESSSSPSNANLGSIEVEKFEEDEEMKSLLSQCSFSSSSGFIF
>Begonia_fuchsioides_Begfu2339S26247
MPEPELDSPAVFPLKPTAVDDRDPLLMDFDVDMGSPWPLDQMSPILISSSDQPFSPLWAFSDGENDGDGRLH
LHSRSASGMSNLNLVGQKPTHNIETNVLQLPPLWGLASMENPDGYCLIKEKMTRALRYLKELTDQNVLAQVW
APVKSGGRYMLTTLEQPFVIDAQSNGLHQYRMVSLMYMFSVDGETDEVLGLPGRVFKQKLPEWTPNVQFYSS
KEYSRRNHALHYNVQGTLALPIFEPSGQCCIGVLELIMNSPKINYAPEVDKVCKALEAVNLKSSEILDHPNTQICN
EGRRNALAKILEVLTVVCESHKLPLAQTWVPCRHGSVLANGGGLRKSCSSFDGSCKGRVCMSASDLASYVVDA
RVWGFREACREHHLQKGQGVAGRAFLSQSSCFSSDITQFCKTEYPLGHYARLFELKSCFAVCLRSNFTGDDNYIL
EFFLPSAITEYYEQLALLGSLLATMKQHFQTLKVASGINFEEGEVFVEIIQASVDEGPSSRFEYVQIPQSVEFTPMS
NGASNGRETCGFNSIKQKLAVVCDNEENNCGAENKTHNSIPAPKKDRKKTSERKRGKTEKSISLDVLQQYFTGS
LKDAAKSLGVCPTTMKRICRQHGISRWPSRKINKVNRSLSKLKRVIESVQVVEGAFGMTPLTTCTNSPPFAFDGP
MQRSSPGEHHSNDKESSLVSKMPRNEEYIHEKSGFSPTIRKESNKSRTKSGSREESGGTPTSHGSCQGSPDDESL
LAKDSHGLPYHEHPSKQADSPKKLDPIAPLPIHDPLTEAEEEPFGAMLIEDAGSSKDLRNLCPSAADAVLDEHNP
ESTPTQFVDIIGSRQEAKRMTIKATYKEDIIRFRIPFSSGVVELREEVAKRLKLEVGTFDIKYIDDDQEWVLIACDAD
LQECVDIPKSSGSNIIRLLIHDLSTNLGSSCESTGEGY
>Begonia_fuchsioides_Begfu2948S03146
MSHWETSSGDLENLTPLNGGTGSLSFEDVYSNFSSLMSFETDAGWCYGGTTDHMLCSSGFSDPSMLSTSLDPL
NFVEDNREPFAMTESSSTTSSLETSFNCGVNSMFQHLDTVFRFEIDSSGANDSKSKQKDAIDHGSYIISRSFGWS
LDERMLKALSLFKESSGGGILAQVWVPMKQANQYVLTTSKQPYLLDQMLMGFREVSRMFTFSIEGKPGAGLG
LPGRVFTSRIPEWTSNISYYKKGEYLRVEHAIGHEIRGSIALPIHDCQSEIPCCAVVELVTNKEKPNFDAEMEVVC
QALQYLLVKFDDYLSHDISHLAFAGQSSSEFSACDTNVLGAPMSLFFLVKGVIHPPLIANPSSKITAASTKVRGFLP
LIVRMMPLFPIHTYLFLELNYLQAVNLRTSLPPRLLPRCLTYDQKSALAEINDVLRAVCYAHRLPLALTWIPYCYNT

GASNGFTRLHLRDCILGPKEKHILCMEETASYVNDKTMQDFVSACANHNLQEGQGVVGNALQTIRPIFNPDVK
TYDINEYPLVHHVRKFGLNAAVAIRLRSTYTGDDDYILELFLPVNMTGSLEQKLLLNNLSGTLQRICKSLRTVSNV
NLVCSEHPKAGIQAEAVQKLAPSFISNSPSRLSDSLNTVEKQKLSNSANDGVETKYSSMKTSKSSKHFEKKQSTG
EKNVSLSVLQQHFSGSLKDAAKSIGVCPTTLKRMCRQHGILRWPSRKIKKVNRSLRKIQTVLDSVQGVDGGLKF
DPSTGGFIATTSAMQEFNNQSCFLLPNKKSPTDEVPAFEDSISVPLEVSVKLEEDECFLAGESMISLLSIPNNNSN
VLPIDINDDSKCSSPLAGDEEETIGEGDDEVIGYNNPLSSSTPNSSSGSQSFVHGSASGGSHSFDGRKQPKDRISC
VDRGSKITFKASYGEDTIRFKFDTSSKYFQLCEDVAKRFKLTSGSFEIKYLDDEKEWVMLASDLDLQECLEIMDDF
GSQTVKLLVRDRVFGGSSSAGNSPPPKPWDRAGASSGPAPFKPPSAGSTSDVVEASGTANPGEIVSSSDRNATT
NINALGRPVPTRPWEQNYGTGNYVGGYGSTSNYNSGYGSGMYGSYGGVGGYSSGMYGTSMYNRGGYGGLG
GSSMYGGGMYNGGYGSSMGAYGSSMGQYGSSMGGYGMGMGGPYGAQDPNNPYGPPSSPPGFWMSFLR
VMQGVVNFFGRISILIDQNTQAFHMFMTALLQLFDRSGMLYGELARFVLRILGIKTKSRQAPPLGPDGLPLPGF
HKAPTNQNLIEGPKAGPDGSWDNVWVNDSDK
>Begonia_fuchsioides_Begfu3240S31174
MEQPSSSKEWGMSCWENSRGQWESLMPLDVGMGSHSFEDVYGNFSNLSTLDTTDGWCYSPDPIFPSGSSL
VPSIPCTSLDPSNFVEDNSKAFSMTEISGVTDSMGSSINCGDTDLFHQLESVFRFSIDSSDVHEPETNQNNVTNT
GSCIISRPVGWSLDERMLRALALFKESSREGILAQVWVPMKHGNQYVLTTSEQPYLLDQMLTGFREISRMFTFS
TEEKPGAVLGLPGRVFTSKIPEWTSNVSYYKKGEYLRVEHALGHDVRGSIAFPVFNFQSAMSCCAVLELVTNKEK
PNFDMEMEVVCQALQAVDLRTTPTPRLLPQNLTNNQKSALAEITDVLRAVCHAHRLPLALTWIPCFYNTRADG
SMTVNVRDTSIGPHEKCIICIEETACYVNDKTMQDFLSVCTNHYLEEGQGIVGKALQSNQPFFNPDVQAYHINE
YPLVHHVRKFGLNAAVAIRLRSTNTGVDDYILELFLPVDMTGRTEQQLLLNSLSGTMQRICRSLRTVSNVDLVCS
EHPKAGFPAEAVQKFTPISFEISKSRTLDSWSANEKLKLLMPLNDDVETNCSSDLTSGSHRLLEKKRSIAEKNVSLS
VIQQYFSGSLKDAAKNIGVCPTTLKRICRQHGISRWPSRKINKVNRSLRKIQTVLDSVQGVEGGLKFDPSTGSFIA
AGAPIKEFNGQSSFLLPEKKLCHRESPGINNSVSVPRPCFNGHSSSIKLEDGEFLMARDGILPSISIPKRTSDILQGE
LSSDSKSSSSLAADEMDTGGEADDGVVEGNHLSSSTGDSSSGSRTLHGSSSGSQSFDDQKDPKDKISCVDSGSK
ITVKVSYGEDMVRFKFDPSWGYFQLTEEVAKRFKLPSGSFELKYLDDEKEWVMLACDSDLQECIELMEDFGGRT
VKFMVRDRPFAVISSTGSCCFLSGD
>Begonia_fuchsioides_Begfu3390S11233
MPEPEFDGAAHFPVKTTADYRDSLLDFDVDVGSPWTLDQISPILITSSDQPCSPLWAFSDGEEVGDEKLHAHPR
SVHGITNLNLATEKPTQNIETRLLQLPPIWGLKSAENPDGYCLIKEKMTQALRYLKELTDQHVLAQVWAPVKNG
DRYVLTTLEQPFVLDAQSNGLHQYRMVSLMYMFSVDGETDGVLGLPGRVFRQKLPEWTPNVQFYSSKEYSRR
NHALHYNVQGTLALPVFEDQSCIGVLELIMNSPKINYAPEVDKVCKALEAVNLKSSEILDHPNTQICNEGRQNAL
AEILEVLTFVCEIHKLPLAQTWVPCKHGSVLANGGGFKKSCSSFDGSCRGQVCMSATDIASYIVDAHAWGFRDA
CLEHHLQKGQGVAGRAFLSQSSCFSMDITQFCKAEYPLVHYARMFELKSCFAVCLRSSFTGDDNYILEFFLPSVEK
EYYEQVSLLGAILATMKQHFHTLKVASGIKFEEGEIFVEIIEASTDDGLKSRFEYIRIPQSFGLPHESDPAPNGGELF
GFNSMKPKLPTGVDDESNTSVEGKRRVPSPSMDVKKRMERKRGKTEKSISLDVLQQYFTGSLKNAAKSLGVCP
TTMKRICRQHGISRWPSRKINKVNRSLSKLKRVIESVQVAEGAFGLTPLTTTANSPPFSPDGTNQRGSQIHKPSD
NQGENKELSQGKDLASWMDGGFVGGTLRFDDYIPAKSGIEPKFSKRSNKSGTKSGSREESAGGSPTSHGSCQG
SQANDSIPAKDLVVFTHHEQPITEAELPGKINPAVSYFAPDLLPATAVEEPFGAMLIEDAGSSKDLRNLCPSAADA
VLDEHIPEPYWPRFPGSSSAQFADRTRQETKRMTIKATYKEDIIRFRIAMSSGLMELKEEVAKRLKLEVGTFDIKYI
DDDQEWVLIACDADLQECVEISKSSGSNIIRLLVHDLSSNLGSSCESTGE
>Begonia_fuchsioides_Begfu3847S33259
MADPRDNFPYSDPYDDLNADDILNFMADRNPCLDDLKNITRNSSHSGPNLEARVGVEVPGEWERLNPQSIA
GGSSGAGSSYVGSDRNNQPPEIIQRKIPFDYNVKQILVSPISPSPFFCSFCQVLRQIVHSNGISVRKLEIHGRFGMI
AHAVLEDRPIISANSSNTHYQMTDLCKKSLDEIKQYLIDYSVSNVVDQYRPLPESLLVFYDTLCAGMEWEMNKN

GKENRPPPRPSNPPPPPPPPSQPPPPPPPQRTGRMTVRDMCEYFHLPIAEASRRLHLCPTVVKKICRKSGLQR
WPYRKIKSYQRKLSMLRSNLNSNEQETTSRTIVEVERIQEELIQIFDVVNLN
>Begonia_fuchsioides_Begfu4799S04580
MDDGILSPAAMLGASDSAMDLDYMDELLLAGCWLEATDGSEFLNQTPPFSSTPFDPSSSGWSALDVNGDLST
VPPPNSDQTPGNENGKVAVQTANQKNTDDFDDELRRRWWIAPGPASSVRERLMKAVGYIKDFVRNKDVLIQ
VWVPVNTEGRMVLTTTGLPYSVNTSCSRLSSYGDISMKYQFSAEKDSKDMVGLPSRVFIRKVPEWTPDVTFFR
SDEYPRVSFAQQCDVRGTLALPVFEQGNKSCLGVIEVVMTSRQIKYGDELESVCKALEAVDLRSYDALGSPNVK
VFYRSNQDLLLEIQGILRFACESHALPLAQTWASCAQQGREGCRHSDENYILCVSTVDQACFVADPTVEAFHEA
CSEHHLLKGQGVVGEAFTTNKPCFVSDIRSYGNTEYPLSHHARMFNLQAAIAIRLRSIHSRKADFVLEFLLPTSCT
DPKEQKKILLSLSIIIQHSSRNLRFVTDKERKEERCRPFNAGVASDSKDRIHEIPPVQIYDNAGSVFSRDNEKSSGH
RHNQQDSSLKEAVEGNEECINFGEGSSSTLGLGKTIEKRRTKAEKAITLQVLRQYFAGSLKDAAKSIGVCPTTLKRI
CRQHGIKRWPSRKIKKVGHSLEKLKRVIDSVEGGSGAFQLASLISNFPELASPNVTRTSPFLASKTSDCSKTSPVH
LEGSIANLQAAASKSPSSSCSQGSSSSLCCSSKSLQFPVPSNEAGSEDPNAEENVGNGELKRVKSEAELHVSSRG
GIYIPRSQSFRGLTEHWGKENLQTQAKDIQRTSEEDNLLRLKVTYGDEKIRLRMHKNQGYRELINEISSRFQIIDTS
KFDLKYLDDDSEWVLLTCDADLEECMDVYRQSSSRGRTIKLSLQLARISQKKGSKYMKTSRSCVGKWEAVGEED
TGTESNLDSGPAKLNVQTVAKCLPMSRDWWKQFDPA
>Begonia_fuchsioides_Begfu58542S38394
MEHPSSSKERGTSCWEPSMGQWESFMPLDGGMGNLSFEDFYGNFSNLSTPDATAEWCYSPDSIFPSGLSLVP
SIPCTSLDPPNFVEDSSKAFSMNEINGGTDSMGSSFNCGDKGLFHQLESAFMFAIDSSDVNESETKQNNVMNT
GSCIISRPVGWSLDERMLRALTYFKESSGEGILAQVWVPMKHGNQYVLTTNDQPYLLDQMLSGFREVSRMFT
FSTEEKPGAVLGLPGRVFTSKIPEWTSNVSYYKKGEYLRVEHALGHDVRGSIALPVFDFQSVMTCCAVLELVTKK
EKPNFDMEMEVVCQALQAVNLRTSLPPRPLLQNLTNNQKSALAEITDVLRSVCHTHSLPLALTWIPCCYTTRAD
AGSTTVHVRDITTGPHEKCIICIEETACYVNDKTMQDFLSVCTNHYLEEGQGIVGKALQSNQPFFNPDVQAYHIS
EYPLVHHVRKFGLNAAVAIRLRSTYTGFDDFILELFLPVDMKGSMEQQLLLNSLSETMQRICRSLRTVSNVDLVC
SEHPKAGFPAPISFGNSKSRTSDSLRVNEKLKLSKSVNNDVETNCSSDLTTSGSPRLLEKKRSIAEKNVSYSVLQQY
FSGSLKDAAKHIGVCPTTLKRICRKHGISRWPSRKINKVNRSLRKIQTVLDSVQGVKGGLKFDPSTGGFIATGTSIE
EFNGQSSFVLPEKKLCCRESPGFNNPVSVPRPCFNDQSSSIKLEEDEFLMARDDIIPTISIPKRTSGILRGELSSDSK
RLSSLAADEMDFGGEADDGVVDHNHLSSSTSDSSSGSRTFLHCSSSGSQSFDDQKHAKDKISSVDSGSKITVKA
SYGEDMVRFKFDPSWGYFQLAEEVAKRFKLPSGSFELKYLDDEKEWLMLACDSDLQECIELMEEFGGRIVKLLIR
DRPFGVGSSAGSCCFLSGD
>Begonia_fuchsioides_Begfu91817S44671
MPEPELNSPAVFPLKTTAVDDRDPLIMDFDVDMGSPWPLDQMSPILISSSDQPFSPLWPFSDEEDDGDIKLHL
HSRSARGMSNLNLVCQKPTHNIETKVLQLPPLWGLTSMENPDVYCLIKEKMTRALRYLKELTDQSVLAQVWAP
VNRGGRYMLTTLEQPFVLDAQSNGLHQYRMVSLMYMFSVDGEADGVLGLPGRVFKQKLPEWTPNVQFYSSR
EYSRRTHALRYNVQGTLALPIFEPSGQCCIGVLELIMNSPKINYAPAVDKVCKALEAVNLKSSEILDHPKTQICNEG
RQNALAKILEVLTVVCERHKLPLAQTWVPCRHGRVLANGGGLRKSCSSFDGSCKGRVCMSASDLASYIVDARV
WGFREACREHHLQKGQGVAGRAFLSQSSCFSPDVTQFYKTEYPLGHYARLFELKSCFAVCLRSSFTGDDDYVLEF
FLPSAITEYYEQLALLGSLLATMKQHFQTLKVASGINFGDGEVFVEIIQASADEGLSSRFKYVRIPQSVELTPVSNN
GATNGGETCGFNSMKQELVAVCDDEENNRAAEKETRNSVPSPQKNRKKTSGRKRGKTEKSISLEILQQYFTGSL
KDAAKSLGVCPTTMKRICRQHEISRWPSRKINKVNRSLSKIKRVIESVQVVEGAFGLTPLTTSTGSPPFALDGPM
QGSSPGDHHSEEKESSLGSKMPRNELSSFMEDRHIIGALRLDEYIHGKAAFFPKIRKESNKSRTTSGSREESAGTP
PHGSCQGSPPDESLLAKDPHDHEHAIKRLVSPEKLNPIAPSPIRDTPTEAEESFRAMLIEDGGSSKDLGNLCPYAA
DAVMDEQNTESSWPNRPGSTPSQLVDITGTSTTIKATYKEDIIRFRIRLSSGVVELREEVAKRLKLEVGTFYIKYID
DDQEWVLIACDADLRECVDISKSSGCNIIRLLIHDLSTNLGSSCESTGEGY

>Begonia_fuchsioides_Begfu91828S44750
MGSHWAGSWSKNDAFGRNEACSFAVPCLLPQFDFSFHAFDWTNDRPFQDSFSEAETLADNFQSDYLCSPLNI
DSISSKQNADECSYDYGETGGFWNGLNEELGFQENFVFCENGGEKIKEMESKNGNKRNREEKNGSSKTLSRTTI
SQYFYMPITQAAKELNVGLTLLKKRCRELGVFSLSLSLSLSLSLSLSLSLSLLHLCHKQNLSKEDGDESEGKLRDAVE
MLKKERNMMEEMPDLKLEDNTKRLRQACFKANYKKRKLMSMVESPSSPPSSNFESIQEVKFEEDEEMKSLLS
HCSFSSSSFIF
>Cajanus_cajan_C.cajan_11912
MEDHFPPKGKEIGYCTSPGVQLEESTSVDGGTKNSVSDDMFSYFSELMNFDTYAGWSNSPSLTDQSLANVFSS
FSSPPDGLNLVEHSNGPFFMTEVGEIHNDMESSPSCEERVIFHQMDIQLGFLDDANNLDSKQNHNGTSQKINT
SDMCNYIISRSSGRSLNDRMLRALSSFMESADAGMLAQVWLPIKHGDEFILSTSEQPYLLDPKLAGYREVSRAF
TFSAEGKPGSCPGLPARVFISHVPEWTSNVGYYNKTEYLRLEHAMNHEIRGSIALPISDENSQVPCAVLELVTTKE
KPNFDRELEIVTHALQFLKKYVIYIQQSDQHRTNDKCPCDDFMQLVNLRTTTPPRLLPQCLSSNKRSALTEIIDVL
RAVCHAHRLPLALTWIPCCYREGTGDETERIRIKEGRTSSNEKCVLCIEESACYVNDGAVRGFVHACVEHHLVEG
QGIAGKALQSNHPFFYTDVKTYDIVEYPLVHHARKYNLNAAVAIRLRSTYTNDDDYILEFFLPVNMTGSSEQQLL
LDNLSGTMRRICRTLRTVSDAELTGIEGSQGGFPREKVSGIFPLSRRNSQITFINDDHDSVQKMSLKVSNLKNNG
IEAAHSQEMNGSRKQIEKKRCTVEKNVSLSVLQQYFSGSLKDAAKSIGVCPTTLKRICRQHGISRWPSRKINKVN
RSLKKIQTVLDSVQGVEGGLKFDPYTGGFIAGGSIMQETESHKYLLFPEKSSVKDPKPATQKKISVVSAPGSTSEN
SAIKLDGNDVGLVGNHLVHSRSVLISSTNRVEGADEHNHPTSSRTTDSSNGSGSIMHGSSSCSQSFENHKHSKV
KSTCVDSGSKIIVKAGYRGDTIRFKFDPSAGCFKLYEEVATRFKLQNGSFQLKYLDDEEEWVMLVNDSDLQECTEI
MDDIGTRCVKFLVRDVPCVLSSRDSNSCFLSDSS
>Cajanus_cajan_C.cajan_12486
MDTSLLTHLLLLNNTLQPELMRSVHVYRQGEGEEREVEREFVFSESGSYGEMQATPIFRLKKSCVSEVCEGYRN
GVWLCIFAFHVDHTPQFSGIPPLLLVTRNPKLQMIPNLLNDLHKIYKLDQKEEDKDIPQDSTGEEWQGNNNISE
PSQKVFRVLEQDLNCLPYEEEESELLDNDTDVESSPGLIEKKKRAPSDLVAKISLSDLVKYFGMPIVEASRNLNVGL
TVLKRKCREFGIPRWPHRKIKSLDSLIHDLQEEAKHQELEDMEAAMAVTKRQKMLENEKEDIEKRPFMDIQSET
KRFRQDVFKRRHRARAIEKHNSTVSTT
>Cajanus_cajan_C.cajan_13414
MEYPFSPKGGGIGDWQSSRAQLEGSTSLDGGMRNSISEDMPNSFSELMNFDTYAGLCDSPSITDQILANELPS
FASLPYPLPDGFNLVQQNSGQCYMSRVGKNNNDMESSPMYGEKVGCQQMDTLLGFFSNSNDGNNLNSKLK
TNGSSQHSNAFDTGNYIMSRPPGLSLDERMLRALSFFKESAGGGILAQVWVPIKHGDQFILSTSEQPYLLDQM
LAGYREVSRTFTFSAEGKSGCFLGLPGRVFTSKVPEWTSDVGYYSMSEYLRFEHAINHKVRGSIAFPIFDMHSEL
PCCAVLELVTTKEKADFDKELEIVCRALQLVNLRTTKPLRLLPECLSNNKKATLTEIVDVLRSVCHAHRLPLALTWIP
CYTEGSRGEATRIQIKEGHSTSSEKGILCIEESACYITDRAMTGFVRACMEHHLEEGKGVAGKALQSNHPFFYPD
VKTYDISEYPLVHHARKYNLNAAVAIRLRSTYTNDDDYILEFFLPANMRGSSEQQLLLDNLSGTMQRICRSLRTVS
DVELSGIGSSQVGFGKKNVPSFFPLSSRNSQIPLINGDRDSSQKMSLKATNLGNNEIEPSPSQERNGSKRQVQK
NRSTLEKNVSLSVLQQYFSGSLKDAAKHIGVCPTTLKRICRQHGISRWPSRKINKVNRSLKKIQTVLNSVQGVEG
GLKFDPSVGAFVAGGSIIQEIDAHKSLMFPEKNTIKDCAPDTLEAVSVPTAPCSEDENFSIKQQAFAPRCKIVLNK
LVWVSRYLIGDEMDIGVDGDDGVVEPNHPTSSSLTDSSNGSGSMMHSNSSGSESFQNQNQSKVKSTIVDSGS
ILIVKATYGEDTIRFKFDPSAGCFRLYEEVATRFKLQNGSFQLKYLDDEEEWVMLVNDADLQECIEILDDIGTRSVR
FLVRDMPCVLGSSGSSNGYLGGSL
>Cajanus_cajan_C.cajan_16064
MDFDIGLESPWPLDHVSLVSNPMSPFLLSTISEQPFSPVWAFSDVEDDRQIRIAASGNTNTTNENPVENDANK
KTVSPLVALPPSENPDGYCLIKERMTQALRHFKELTEQNVLAQVWAPVRNGNRYALTTSGQPFVLDPHSNGLH
QYRTVSLMYMFSVDGENDEIQGLPGRVFQHKIPEWTPNVQYYSSKEYQRLNHAQHYNVRGTLALPVFEPAGQ

SCVAVLELIMTSQKINYAPEVDKICKALEAVNLRSSEILEHPYNQICNEDRQYALAEILEILTVVCETHSLPLAQTWV
PCKHRSVLAHGGGVKKSCSSFDGCCMGQVCMSITEVAFYVIDARTWGFHEACVEHHLQQGQGVAGRAFLSH
KMCFCGNITQFCKTEYPLVHYAIMFGLTSCFAVCLQSSHTGNDEYVLEFFLPPMITDFNEQKSLLGSILATMKPHF
QSFKIAAGVELEENGSVEIVEAINERVHLRFESIPITQSSKSPPRHALPNMGEALPPEPSEQQIMAYCDDINDGGS
LGDNAGGHIDQNTLLETKTKKKPSERKRGKAEKSISLDILQHYFTGSLKDAAKSLGVCPTTMKRICRQHGISRWP
SRKIKKVNRSLSKLKCVIESVHGAEGAFGLNSLSTSSLPIAAGSFSEPSTSNKQIEMGEQFLGARTQSPEKLINDKV
GTIQEIGTKGSNKLRTGSGSSEGSANPTPHGSCQGSPPNEISPTKDIFIAGNNEQCLVLRGSLESTLLSTRTSTPNR
PTAYPMPNIVDTTEPQVPFVGQLLEGAGSSKDLRNLCPSADAVLEDQVPEACRMNPQRVKSVTIKATYKEDIIRF
KVSLDCGIVELKEEIAKRLKLEVVTFDIKYLDDDHEWVLIACDADLLECMDISSSSGSNIIRLVVHDILSILGSSCESS
GDWKGCI
>Cajanus_cajan_C.cajan_19885
MSLPHWPPSPKPFFCSCCQVLRQIIHTNGSRFDKLEIHGTIGMISHAIIRNTTPGDPSSNYQMVDFCNRNKEEIK
SFLKQYCEQQNIFGYIIMKDPLSAYYDALSTGMDWAQDISDEDDDLISENDVEQDQEPEPEPENEMASNARRL
QVIVFVFLGSVAERVAKMTLGDLSDYFHIPIKETAKLLEVSTSVVKKVCRKAELYRWPQRKVKSNMRKITVLRRGL
ANPGTREKTRVEIQRLQQEMVEYCGGLAPTGIEMLQV
>Cajanus_cajan_C.cajan_36762
MDNNNFGFVSYDDEDPFLCPLIPNLLVEVDPSLVLPKAQPHQQNDHPFGLQHGNNVPFTIQDQFMQDNNN
NGEVANFDVESRGQQTMHVDGSNNISNEGGVGNFEIEGRGQTMHGDGSNNIFNEGRVVTSVSYWPLIPRPF
FCTCCQVLRQIIHTNGVQFEKLEIHGTIGVMGHAIIQNRNITQGDHPSSDPHQIIDFHYRSTEQIKSFMVEYCTQ
MSKLGYIIVEDVLSTYYEILCTGVDWAKDTSDEDIDLIPSNDMGQDFEPEPQPQHEPEPEAVNLKRKRRRPDAE
QRKRIKAMKLEDCSDYFHLTIGAAAQKLDVCISALKSLCRRNNLENWPHRKVPML
>Cajanus_cajan_C.cajan_30678
MEVSPLEGFYEHDWTHHFPYSSKQLCFNELPDIENFNFDLSLPLLGEEMEEVEQKPLKVVVPQKGHGDRDKGF
GGALVVNEKGFATRVKKEEEENVDKKPLPVPRSNMKKKKKNNNNKKSCVLEFEQIKKYFDVPINEAAKKMDVG
LTLLKRRCRELNIMRWPHRKLKSLQTVIDNVKELGLANEIAMLEKHKRMLEKLPGLELSEETKKLRQACFKANYK
RRRCLALKA
>Cajanus_cajan_C.cajan_27730
MLSRKTVSEYFYMPISQAARELNVGLTHLKKRCRELGIQRWPQRKLMSLQTLIKKIQDQIEGYENDEKIRAAIEVL
EREKRMVEEMPDLQLDDNTRRLRQACLKANYKKRRLMGMHEVHETTISP
>Cajanus_cajan_C.cajan_27731
MLSRKTVSEYFYMPISQAARELNVGLTHLKKRCRELGIQRWPHRKLMSLQTLIKKIQEQGEGNENDEKIRAAIEV
LEREKRMVEEMPDLQLDDNTRKLRQACFKAKYKKRKLTGMHEVHETTISP
>Cajanus_cajan_C.cajan_27743
MLSRKTVSEYFYMPISQAARELNVGLTHLKKRCRELGIQRWPHRKLMSLQTLIKNIQEQGEGYENDEKIRAAIEV
LEREKRMVEEMPDLQLGDNTRRLRQACFKANYKKRKLMGMHEVHETTISP
>Cajanus_cajan_C.cajan_29749
MMEAPPPDGTTSMDFEYMGELFLDGCWLEASADGSEFLLQSPSFSNSLFDPSFSWPALETNHNESHDAAFGT
QQESHNSMVNIVSGGGGGSGMQFQFDAHSVEGASEGVKRWWFAPTPSPGPGPSITEKLLRALTWFKDYNR
NKDMLIQIWVPVHRGGRPILAANDLLFSLESRSVNLAKYREISVGYEFSTDEGESKELVPGLPGRVFRNKVPEWT
PDVRFFKSDEYPRVDHAQEYDVSGTLAVPIFEQGSKMCLGVIEVVMTTQQINYGPELESVCKALEAVDLRSSKH
SNIQIVKACNRSYDAALPEIQEVLRSACEMHNLPLAQTWIPCVQQGKEGCRHSDDNYLLCISPVEQACYVRDPS
IRPFHEACTEHHLLKGEGVAGGAFMTNQPCFSTDITSLSKKDYPLSHHARLLGLRAAVAIRLRSIYNSTDDFVLEF
FLPVDCNDSEEQRKMLTSLSIIIQRVCCSLRVISDKELEEANLSVDEVIGLADSGVSRTAICSGLQQKEKSSETMCR
KFSDLRQQENPILKGNLDCVRECSTSVEGNFSGAGISRTVERRRAKAEKTITLQVLRQYFAGSLKDAAKNIGVCTT

TLKRICRQHGIKRWPSRKIKKVGHSLQKLQLVIDSVQGASGAFQIDSFYSNFPDLASPNLSGTSLFSTLNQADNP
NSVSTQPEPGALSPEGASKSPSSSCSQKLQHHTNNKDPAVGEDSADVVLKRIRSEAELKSLSRDGTKLLPRSQSQ
ETLGEHPITQYHRPLLKTSSKVDAHRVKVTYGDEKTRFRMLKSWGYEDLLQEIARRFNVSDMRKFDVKYLDDDC
EWVLLTCDADLEECIDVCQSSESSTIKLCLHASSHSMRSSLEFR
>Cajanus_cajan_C.cajan_33924
MEYEGLVQNGAYGSLSEFFAPETDLIDELFVEGCWVETRVNSKSEWSMEAQIEESEVGKRWWIGPRANPGPS
TSVQERLVVAVGYLKEYAMNSIQVWVPARTTRAQPPYALGLDYGDATRGFEFEDEWSPNPNIRFFRSDEQYDV
RAGSLALPVFERGTAICLGFLQILMPNNPNLHNVCHALQGVDFINQNLNVPPGVTVKGFDELYEGALNEIVQVL
TCVCKAHKLPLGLTWAPCIQQGKSGCGHLKELLGFQEACSQCHLLGQGQGQGVVGTAFTTTKPCFANDMSKA
LPLAHHANMFGLHAAVAIPLRSVSADFVLEFFLPRDCQDYKHILNSLSMLVQQACRSLHVVTVEEEFLLPVAHE
MESSSWIAHMMEAQTQQNGKGVSVSLEYLQEPKQEFKVTTNGNCNENEESYGGRRGGRKSGDKRRTKAEKT
ISLPLLRQYFAGSLKDAAKSIGVCPTTLKRICRQHGITRWPSRKIKKVGHTLRKLQVVIDSVQGAQGAIQIGSFYTS
FPELSSTTATASAVSQSSKNVNNNNDTSENNQGQLGATTFKSPTSSCNASIQLQAPHPIQCLEVPPLPQTSVWN
TTTFRVKATFGDEKIRFSLQPNWVFRDLQMEIARRFNLNENINNIQLKYLDDAREWVLLTCDADLEECKDINRSS
HNRTIRLFLFQASPLNHANAFGSGSPSYS
>Cajanus_cajan_C.cajan_01716
MPFISGKGEVYPALDWAHDFPIQDYYFDAVPLMDYYPSDPLYETLSIEPTPTVQDYDFYDIKKGFSVWNEVDAV
LDSEKVFLFCNNEESGSGKEMMEDGKVNREREERIGSSSTRLLSRKILSQYFYMPITQAARELNVGLTLLKKRCR
VLGIRRWPHRKLMSLQTLINNIQELGKEEGKESEEKLRSAIEILEREKKLLEEMPDIELEDNTKRLRQACFKANYKK
RRLEGRRETQSSSFSGHARTIDTTGEYSYENEEERDIKYPLSSVNVMI
>Cajanus_cajan_C.cajan_07275
MSESEEENPDCVPRSKAEEEGGCNMDFDLELESSWPLDHMSFVSNPMSPFLFSNTSDQPYSPLWAFSDGEDE
RHPRFPASAFSDCHKIFSCDSNSVAEKPVENDDHKKLLPPLVPMPPVDTLDGYCVIKERMTQALRYFKELTELNV
LAQVWAPVRNGNRFVLTTSGQPFVLDPHSNGLNQYRTVSLMYMFSVDGENDGSLGLPGRVFQQKLPEWTP
NVLYYSSKEYPRRDYAQHYNVRGTLALPVFEPSMQSCLGVLELIMTSPKINYAPEVDKICKALETVNLRSSEILDQ
PYNITLAMLLNQICNEGRQNALSEILEILTVVCETHNLPLAQTWIPCRHRSVLAQGGGLKKSCSSFDGSCMGKV
CMSTTDIAFYIIDAHLWGFRDACVEHHLQQGQGVAGRAFLSHNMCFCSNITQFCKTDYPLVHYALMFGLTSCF
AICLRSSHTGNDDYVLEFFLPPRVTDFHEQKTLLGSILASVKQHFQSLKIASGVELEDCSIEIVEATIERVHTRFESIPI
APSIKSPTRHDTSPNMGRNIEHIPSLEAKNIKKPSERKRGKTEKSISLEVLQRYFAGSLKDAAKSLGVCPTTMKRIC
RQHGISRWPSRKINKVNRSLSKLKRVIESVQGAEGAFGLNSLSPSPLPIAVGSFPEPFPYPMPDFASTELQEPFGG
MLIEDAGLDLSPKQSMDTPNKAVTPFAARKEMKTVTIKATYREDIIRFRVPLTCGIVELKEEVAKRLKLEVGTFDIK
YLDDDHEWVLIACDADLQECMDVSRSSGSNIIRVLVHDITSNLGSSCESSGE
>Cajanus_cajan_C.cajan_37712
MQIWVPERGGGGGGGGVGPGPDESVWGGYPMNMNMNMNMNMNMNMNMNMNMNMNMNMMNRSI
RVGFLRWEEGVPYGYEEGPGWMGLPVLERGSGKCLGVLHIEYEYEGGVEGAVNEVVEVLTCVCKAQNLPLALT
WAPCTQAKCGYGHSHSNYVSTVDRACFVGDAHLLPFHEVCSDHHLLRGQGIVGTAFTTAKPCFATDITAFTNDE
YPLAHHASVLGLHAAVAIPLRSVSTDFVVELFLPKDCLLREHQNHLLNSLSLLLQQACRSLHVVTEDELSFPFPQV
VEEQSWIANMMEAQAQKGKGVCVSLEYLELEEPMEEFKVTSNCNWDKTPSYVEEQQVFSDFIAQDSQTHTH
TFSARRGRKSGEKRRTKAEKTISLPVLRQYFAGSLKDAAKSIGVCPTTLKRICRQHGITRWPSRKIKKVGHSLKKLQ
LVIDSVQGAEGSIQIGSFYSSFPELSSAAPCESKNKNINNSLHGHAESPVGVAVALRVKATFADEKIRFSLQQVWG
FAELQVEIARRFNLNEMRSVVLKYLDDEGEWVVLACDADLEECKDLHTASHNRTIRLALFQASPPNTFPYPSPST
>Casuarina_glauca_Casgl329S15886
MEMSSQHNPNANAASADQSLSFDDISNYFSLPLNDAATNLGVCTSVLKKICRDNGLDRWPYRKFLCGKSIEEIK
RYAARERHKELAQISKIGRESGSQQANSDMSKLQGGTFPHNLQQQGAKNIQNLRPQNLLSTSLTKGITTLDEFK

CGFPSGGLFTVSNKWWGNSSPDCCEGIFGDEAGINENEKHESQEMAAESSNMVTVDKEKCEGGKEASEIALQ
GMGLLAAVRKRAIEGREALKLGVVRGYGINKIGKKEKSLLLRIFKSSLPKQWLYGS
>Casuarina_glauca_Casgl94S06200
MDSSRLNVSQIENPQDFDWNAVEYADKLLELPPLDKFFDFDPLPFLQNSKQLALTEFQDFEDLNPHFFGADYKL
PFPYEDVVVDQKPLNSTTDYVDHYDNLVSDLSASSIVVQGEYASSSQSEEEKRSSGRKKSVVLELDEIRKHFSLPII
KAAKEMNVGLTVLKRRCRELNITRWPHRKIKSLKSLINNVKELGLTKDVVMLEEHKRRLEKVPDMELTEKTKKLR
QACFKAKYKKRRSLTIQT
>Casuarina_glauca_Casgl109S07006
MEKVVLSPGDMLGALPDTAMDLDFMDEIFLDGCWLETTDGSQFLHQIPATSGALLDPSLVWSALEANGNGSE
SLSQEYNQVEAQRQSPDESQERSLVNAPSRGQNPIFSLAGYSMSEKNVTEGSELGTRWWIAPRGNPGSTSSVT
ERLMKAIGYIREFTRDRDVLIQLWLPVNREGRRILTTSNQPFLLGSNCPRMARYRDISGNFVFSAEKDSKELVGLP
GRVFLDKVPEWTPDVRFFRSDEYPRVNHAQEYAICGTLALPIFEKGSSTCLGVIEVVMTKRHIKYRSEVESVRKAL
EAVDLRSTDALSTDHVKAFNKAYQTALPEILEVLKSACKTHRLPLAQTWVPCIQQGKEGCRHSDENYLDCVSTV
DHACIVADPNMQGFHEACSEHHLLKGQGIVGRAFRTNQPCFSEDITSSGKTDYPLSHHAKVFGLRAAVAIHLRS
IHTGTADFVLEFFLPVDCTDHQEQKKMLSLLSVIIQQVCQSLRVVTDEELKEEAKFAVSGKPSSEETSNEKSSEFW
HHSRERNLRNAEFGEGCSAYKEGSFPINRAGEKRRTKAEKTITLEVLKQYFAGSLKDAAKSIGVCPTTLKRICRQH
GIKRWPSRKIKKVGHSLRKLQVVIDSVQGASGTFQIDSVYTNFQELASPNLQGSSPFSASKFNDHPQLSSMQLE
GRILSPQAAASKSRSSSCSQGSSSSQCCSSGTHQHPSTFNVAGCDDPVIGENIGDGVLQRITSEAELQASNEGPK
LMARSPSHKSLREHPNAENLLPLKKDSGRLSQEGDALRVKITYGDEKVRFRLQNNCKYTDLLLEIARRFDIDDINR
FDLKYLDDDAEWILLTCDADLEECIDVGRSSQGQTIKLCLQVSHYQRGMS
>Casuarina_glauca_Casgl993S23747
MEIERKLNMDYGAFASNSTTYGNLTDTAMEMDFMDELLFEGCWLETTSGSNHLPSGPLTSRALNDPSHYLPLL
DSNSSGHLNISHHQQIFQEETEGTFPESEGILVEGTELGRRLWIAPRANPSPSTSVKERLMLAIGYLRECTKNMN
VLIQIWVPIRRGGSYFLTTQDQPYYFGANCKNLANYRNVSKAYQFAVEEDMEESAGLPGRVFLGKLPEWTPDV
RFFKKDEYPRINYAQQYDVRGSLALPVFERGSGTCLGVVEIVTNTQKINYRPELENVCQALESVDLRSSQLLSPPG
VKACDELYQAALAEIIEVLATVCKAHRLPLALTWAPCYQQGKGGCRHSDENYALCVSTVDAACFVADLDVLGFH
EACSEYHLFRGQGTVGTAFTTSKPCFATDITAFSKTEYPLSHHARMFGLRAAVAIPLRSIYTGSSEFVLEFFLPKDC
QDPEEQRQMLNSLSIVLQQACRSLHAVMDKEPEEQEVIYPVKEIAIASDVRINKEEPQKSGSPPMREASTKESS
WIAHMMEAQQKGKGVSISLEYQEEEPKEEFKVTTHWDNTLGGSCHGQAFSDFGQLQQSSGSKGSVEGGGDS
YSYGSRRSSGGRRAGEKRRTKTEKTISLPVLRQYFAGSLKDAAKSIGVCPTTLKRICRQHGITRWPSRKIKKVGHSL
RKLQLVIDSVQGAEGAIQIGSFYSNFPELSSSGNSSFSSLKMNENSKQSNAIPETSGLFIQGSSTLSKSPPSSCSQN
SGPSIFCPSGAKQQNTTVNTLSTGETLMRENPVGVLQMMGCTEVNLHAMSQQDLSLLQGVESFKSFGSHPGL
ETLPILPESSSHNSQYGGALRVKATFGDEKIRFSWQQNWTFGDLQLEIARRFNLDDINRVDLKFMDDDGEWVL
LTCDADFQECIDIHRASESHTVRLCVQHASNPCLGSPFGNTSLS
>Casuarina_glauca_Casgl103S06587
MPGPKDPKSTGLTAEEQPQEEEEEEEEEEEEEVEEEEDRLERLVMDFDDLDDSWPLDQILLLSNGNNDKSPSS
MSPFYLSCSDQPVSPLLAFVDGGDDAVHVASASPDSSLLLSRSPNFITEMPMENVNNRRFPSPFMGLKPLDDL
DGYCVIKERMTQALRYFKDLTEQNVLAQVWAPTKYGGRDVLTTSGQPFVLGPHSNGLHQFRMISLGFMFPVD
EEKDGVPGLPGRVFRQRLPEWTPNVQYYSNKEYPRRDHAEHYNVQGSLALPVFDPSGQSCVGVLELIMTSPKI
HYAPEVDKVCKALEAVNLKSSEILDKPHIQICNEGRQNALAEILGILTEVCETHKLPLAQTWVPCMHRSVLAHGG
GLRKNCTSFDGVCMEQVCMSTTDVALYVVDARMCGFREACVEHHLQKGQGVAGRAFSSHGLCFCGNITRFC
KTEYPLVHYARMFGLASCFAICLSSTFTGDDDYILELFLPPSITDFYEQQILLASLLATMKGHFQSLRVASGIELDKE
VSVEIIQVSADERVDSRLGSLRVPVSMKSPTRPEGLLNNGDTVQLKSSQQQLMLHSNGIHDKGATAKNGVGSI
NHFSSLENKEMKKTSERKRGKTEKSISLEVLQQYFAGSLKDAAKSLGVCPTTMKRICRQHGISRWPSRKINKVNR

SLSKLKRVIESVQGAEGAFGLDSITPSPGPVPVAVDSQPSRLNRSSPQTSPTSKLSEPQKNNELLAGRPLKNDEEV
GNQEEDQLHRQRLLSPKNDIRDHSRSTSEPGKGSNRSKTRSGSCEESAGTPTSHGSCQGTPLNGRTMPKNQYV
SSVNEQCIEVARSPGLAYEPPGEPCISASYLIPTTLLMPETQETFGGMLIEDAGSSKDLRNLCPAVADAMLEEQLP
ELCRTNPPYPSTAPNQPMAPPVHTTPHVTAMNEMKSVTIKATYREDIIRFRISLSSGIMELKEEVAKRLKLEIGTF
DIKYLDDDQEWVLIACDADMQECMDLSRSSASNIIRLLVQDAMVNLGSSCESSG
>Casuarina_glauca_Casgl177S10285
MAGQTLNGWWPKHELVYKDEEPFFFPSQMPSVDVRKKDYISHLCIFCGSGYSALDWQYDLPIEESFLDAVPLM
EGFPTDPLYEQVDLESTTSLIQEDAFRGYETRVGVWNDMANRFEHKNQALPLCDSGNKGKEPMVEGKTKRCR
EERCSSSSTLSRTVISQYFYMPITQAAKELNVGLTLLKKRCRELGIRRWPHRKLMSLQTLIQNVKELVKEEGEESEA
KLRDALEILEREKKMLEEIPDLQLEDNTKRLRQACFKANYKKRKLTGMNSLSCSSSNRSSADDVMADDRMNED
EDEYLFLDSFSSSSIMF
>Casuarina_glauca_Casgl618S20178
MENSVSFDGTGNLNSEDNFSELINFDNYPGWCSSPGATDQFFSYNVSPSVPAVPCGLALDALDLTEQNTGSFP
VNRVCGTFNPVGNSFGPGDKMLFQHMDAQYGFPVDASVGNDSAVAQNNGSLQQNNGSLQQNNELDMEN
CLIPRPLSTSVEEKMLRALSFLKDSSAGGILAQVWVPMRVGDQYVLTTCEQPYLLDHMLAGYREVSRLFTFPAER
RSGCVLGLPGRVFLSKVPEWTSNVSYYKKGEYLRVDHAANHEVRGSVALPVFEDPETTCCGVLELVTTKEKSNF
DAEMDTVCNALQAVNLTTTLPPRLQPQCLSKNQRAVLAEIFDVLRAVCHAHRLPLALTWIPCCYIEGVGYEIGRL
QIRGARTNSGEKHILCIEETACYVNDRKMEGFVHACLEHHLEEGQGIAGKALQSNHPFFSTDVKSYDISEYPLVH
HARKFDLNAAVSIRLRSTYTGDDDYILEFFLPVNMSGSSEQQHLLNNLSGTMQRMCKSLRTVSDAELLGAGGS
EVEFPKGAAQHFPPMAIPRRNSQIILSDSDVNSIEKIPLNVSDTINDGFEADSPPEQAISGSRRQEKKRSTAEKNV
SLNVLQQYFSGSLKDAAKSIGVCPTTLKRICRQHGISRWPSRKINKVNRSLRKIQNVLDSVQGVDGGLKFDPNT
GEFVATGPVVPESDSPKKLLFPDKNLCAKDGKCSFNNENAVSIIKLESDDCSVGDQVEFSSGKHITNVSQVEPMK
YNVSLVDCSEDPKLVAIEAPRALPEKASLGSSLANRSKEWGLHNGSLKFENPDSHFVARSSDSLAADEMDTGVG
GDDDGMVEPTQGSSSSMTDSSNASASTMHGSSSSSMSFEDQKHSKVKSSCADSGSKIIVKATYKNDTIRFKFE
ASAGCFQLYEEVARRFKLQNGTFQLKYLDDEEEWVMLVNDADLQECVEILEDIGSRSVKFLVRDTPCNISSSGSS
NCFLVGGS
>Casuarina_glauca_Casgl328S15836
MKVAEHPPGSKRRTPTSKPIFQPFCADFLFSSCELIRSLHVYRSEDGKEYEVGREFLVSAGGSYTEMEAHPALILL
NFRVSEVFEGLLNGFWLCIFAFHADCPPCSTCIPSPLTMSRYPPNSSSDETHHKPGKSMRALYQDLNCLPYPIAM
PRLSEDKQKEHSPPGIVQSKKKRAASESVARIALSDLVKHFDLPIVEASRNLKVGLTVLKKKCREYGITRWPHRKLK
SLDNLIQDLQEEAKRQEREDEAAAMAVAKRQRMLESEKENIERKPFIELKSETKRFRQDVFKRKHRARALGTLG
PPGSSDNGRE
>Casuarina_glauca_Casgl64S02453
MTDPRAVVPYHDPYDGQLPEDILTFMDDANPTLADVSYSIPTMPPPDFDPFGNSVLWDMCNVSNNEARNSQ
AGPSEVHAGTSNTEGAYVESQRPSGGNMRPLPIWPTEPIPFSCSCCQVLREIIHTNGTCTKKLEIHGRFGMICHA
ILSSLQNVIVNSSSNQYQMFDFCKKSIEDVKHFLVNYCIEQNLAGFVMVQDPLAIFYEALCVGLDWDEDLLADD
FFQSSPSDSEQRERAGKLTLQDVACYFHLPIEEAARRMKLCPTVLKKICRRDGMNRWPHRKVRSIQRQISSLTAG
LDSNNPEVKANAEAEMDRLQQELTNVCAGVVSNSLYEECNLPPRSRID
>Cercis_canadensis_Cerca9S31548
MDYSRPTLTTLIVFMNTIREELIRSVHVYRLQDGKQSEVEREFYFSGSGSYAEMRASPLLRLQKFRASEVLEGYQN
GVWLCIFSFHADHKPPFSGIPSLLLVTRNPKLRTIPTLFNDLYLINKLDCRSEGKVIQQILPEERFQANGNDDHPSK
IILPVLDQDLNCLPYSVDMSELPDNGLVDESLTGNIEKKRRAATAHIKKIDLPELVKYFDMPIVEASRNLNVGLTVL
KRKCREFGIPRWPHRKIKSLDNLINDLQEEAKQQEPENEAAAVAVTTRQRMLESEKENIERKPFMDIQSETRKFR
QDVFKRRHKARAKQSSSVSST

>Cercis_canadensis_Cerca20S17733
MDYSTEFDFPKIENSQGFDSFYPVEQQESMHLYRVMEFSPFEGFFDLDSVPLPHQREQLCFNEFPNLEDFSSNF
ASLEYNLPPLYEDLAIDQKPLSIISPSPPPAHCESVANNFNELSMIVASGSSNSCKNEEREEGTRSNGRRKRTCSLE
LDEIRNHFDVPITKAAKQMNVGLTLLKRRCRELNIMRWPHRKIKSLKSLINNVKELGLTEEVAMLERHKMLVEKL
PDMELTEETKRLRQACFKANYKRRRYLAIQA
>Cercis_canadensis_Cerca58S27147
MGDGAVSPGTLLEAPPESAMDFDYMDELFLDGCWLETTTEGSDFLLQTPPTCNPLFDPSFSWPVLETNGVLSL
SPSHNGNQEERQSPLFNESQETPFNAQPVSQDISNIEGSCSQSEFHSVEGSEVVTRWWIQPTPTSNPGPASSV
MERLIRALVYIKDFSRDKDVLVQIWVPVNRGGRQILTTNDLPFSLGSTCPNLSKYREISEKYQFSAEDESKGLVAGL
PGRVFRGKVPEWTPDVRFFRSDEYPRVDHAQEYDVRGTLALPVFEQGSRSCLGVIEVVMTTQQIKYRSQLESV
CKALEAVDLRSSEISSIQNVKEACNESYEAALPEILEVLRCACEMHKLPLAQTWVPCIQQSKEGCRHSNDNYLHC
ISPVDHACYVGDPSVQAFHEACSEHHLLKGQGVAGGAFMTNQPCFSADITSMSKTEYPLSHHARMFRLRAAV
AIRLRSIHSSRDDFVLEFFLPLDCIDGEEHKKMLTSLSMIIQRVCHSLRVISNNELEEETRLLVDEVIAPADGVSART
ATWMDLQQSGTVASLRTAEKSSETIGKFSDLSQQQQDSTFDEGSLSSVGISKTGEKRRTKAEKTITLQVLRQYFA
GSLKDAAKNIGVCTTTLKRICRQHGIKRWPSRKIKKVGHSLRKLQLVIDSVHGGSAAFQIDSFYSNFAELASPNLS
GTSLLSAKQNDNPNPSTTEPEPGMLSPSKSPCSSCSQSSFSSHSCSSISEQHHLTLTQNVASRKDPMVGEDCSDC
VLKRVRSEAELKGSSLDGAKVLPRSQSQEALGQHPKNEFLRPRTHQVAQKEDAYRVKVTYGDEKTRFRMPKNC
GYQDLLGEISRRFNINDMRKFDIKYLDDDSEWVLLTCAADLEECIDVCHSSQTRTIKLSLHVSNHKYRNRSLFVVG
ENYAGHYVPQLAELMLQFNKKDKLFNLKGIAIQRTECSHLFVTTHHVTLDVCISSVFSQTKILNPQMYNRLLPSR
LVGVRRWSAHSNVLDYELLDLEIPTITVVGKLVRAGIPVLVYSGDQDSVVPLTGTRTVIHQLAKELGLKTTLPYRGL
VGGWTQVYGNILSFATIRGASCEAPFSQPQRSLVLFKSFLEGRPLPEAF
>Cercis_canadensis_Cerca93S11435
MSESEEENPELAPKSKTPEDPECCMEFDLDLESSWPFDQISFVSNPTSPFLIPPSSEQPSSPLWAFSDVDDDRHA
RVSASSFSDCNRLFSCNSNSTPEAPTENDDGKKNLSPLVALPLLENPDGCCLIKEKMTQALRSFKELTGQNVLAQ
VWAPVRNGNRYVLTTSGQPFVLDPHSDGLNQYRTVSLMYLFSVDGENDGILGLPGRVFQQKLPEWTPNVQY
YSSREYPRRNHAQHFNVRGTLALPVFEPSGQSCIGVLELIMTSQKINYAPEVDRVCKALEAVNLRSSEILDHPNT
QICNEGRQNALAEILEILTVVCETHKLPLAQIWVPCRHRSVLAHGGGLKKSCSSFDGSCMGQVCMSTSDVAFYII
DAHIWGFREACVEHHLQHGQGVAGRAYLSHDMCFCGNVTHFCKTEYPLVHYALMFGITSCFAICLRSSHTEND
DYILEFFLPPKITDFYEQKTLLGSILAIMKQHFQSLKIASGVELEEYSSIEIIEATNESVYSMFESIPISQSTKSPPGPDIS
PKVGEMELLDSSEQQTMMYVDDIKGEGTLVDNTGGSIDQVSSLETKNIKKPSERKRGKTEKSISLEVLQRHFAGS
LKDAAKSLGVCPTTMKRICRQHGISRWPSRKINKVNRSLSKLKQVIESVEGAEGAFDLNSLSTSPLPIAVGSFPEP
SNKFNQQSSLSTGPSETQMKENELDVFQVPEMNRQVGVEDQLLGTRIGSSEKVIHDKVGSPQEFSKGPNRSAT
RSGSSEDSANPTSHGSCQGSPPNENSPAKDTFIAANNEQCVVEGGSPELNMQPTNKLNSPTAYSIPDLVATEPQ
EPFGGMLIEDAGSSKDLRNLCPSVADAIVEDQVPEASGTNPLCSDLAPKLCINTLGNTMTPSAAMREMKSVTIK
ATYKQDIIRFRISVTCGILELKEEVAKRLKLELGTFDIKYLDDDREWVLIACYADLQECMDISKASSSKIIRLLVHDIAS
NLACSYDSSGE
>Cercis_canadensis_Cerca90S30910
MEYPFSPKGKGIGYWTSPRAQLEGSTSLDGGIRNSISEDVLNNFSELMSFDTYAGWCNSPSVNEQILANGFSSF
APYASSDGFNLVEQSSGAFFMTEVSGNYNTMGSSPNIGEKVEFQQVDTQLGFSDDANDANNLNSKQNNGSF
QQLNTLDLGNYIIPRSPGWSLDERMLRALSFFKESAGGGILAQVWVPIKQGDQFILSTSEQPYLLDQMLAGYRE
VSRAFTFSAEEKPGSLPGLPGRVFISKVPEWTSNVVYYNKTEYLRVEHAIDHEVRGSIAVPVFDRHSEMPCCAVL
ELVTTKEQPDFDRELEIVSQALQVVNLRTTMPPRLLPQYVSNNKRAALTEIIDVLRAVCHAHRLPLALTWIPCCYI
EGVGDEITGIRIREDDSNSSEKGVLCIEESACYINDRLMEGFVHACVEHHLEEGQGIAGKALQSNHPFFYPDVKT
YDISEYPLVHHARKYNLNAAVAIRLRSTFTKDDDYILEFFLPVNMRGSSEQQLLLDNLSGTMQRICRSLRTVSDAE

LSGREGSQEGFQNEKAPCFLSMSRRNSQIALIDGDHDSIGKMPLKASNLRNNGIEAAHNQVMSGPRRQVEKK
RSTAEKNVSLSVLQQYFSGSLKDAAKSIGVCPTTLKRICRQHGISRWPSRKINKVNRSLKKIQIVLDSVQGVEGGL
KFDPSTGGFVAGGSIIQEFDAHRSLLFSEKSMPVKDPELVTQDAVAVPLVPCTEHDNLVIKMEEDDVYLTSGNQF
VHSRSVLTHNSCEGELKKVNASSVDCSEDSKSMAIGDGSCHTARLGTKDQDCPDQACLGSVLAKKGDKWGQN
RGGLGLENANCDIVSQSSSSLVAGGIENGVDGDDGIVDHSHPASSSMTDSSNGSGSMMHDSSSGSQSFENQK
HSKVKSTCVDSGSKIIVKATYGEDTIRFKFDPSAGCFQLYEEVATRFKLQNGSFQLKYLDDEEEWVMLVNDSDLQ
ECLDILDDIGTHSVKFLVRDLPFVLSSSGSSSCF
>Cercis_canadensis_Cerca189S06512
MGDGAAPPGTLLEAPPESSMDFDYMDELFLDGCWLETTTEGSDFLLQTPSTCNPLFDPSFSWPVLETNGVLSL
SPSHNGNQEERQSPLFNESQETPFNAQPVSQDISNTEGSCSQSEFHSFDGSEVVRRWWIQPTPTSNPGPASSV
MERLIRALVYIKDFSRDKDVLVQIWMPVTRGGRQILTTNDLPFSLGSTCPNLTKYREISEKYQFSAEGESKVLVAGL
PGRVFRGKVPEWTPDVRFFRRDEYPRVDHAQEYDVRGTLALPVFEQGSRTCLGVIEVVMTTQQIKYRSQLESV
CKVLEFNDWRKVINLQAVDLRSSEISSIQNVKEACYESYEAALPEIQEVLRCACEMHKLPLAQTWVPCIQQSKEG
CRHSNDNYLHCISPVDHACYVGDPSVQAFHEACSEHHLLKGQGVAGGAFMTNQPCFSADITSMGKTEYPLSH
HARLFRLRAAVAIRLRSIHSSRDDFVLEFFLPLDCNDGEEHKKMLTSLSMIIQRVCHSLRVISNNELEKETRLLVDE
VIAPADSVSGRTATWMDLRQSGTVASLRTAEKSSETIRKFSELSQQQQDSTFDEGSSSSVGISKTGEKRRTKAEKT
ITLQVLRQYFAGSLKDAAKNIGVCTTTLKRICRQHGIKRWPSRKIKKVGHSLQKLQLVIDSVQGGSAAFQIDSFYS
NFAELASPNLSGTSLLSAAKQNDNPNPSTTEPEPGMLSPSKSPCSSCSQSSFSSHSCSSISEQHHLTLTQNVASSK
DPMVGEDCSDGVLKRVRSEAELKGSSLDGVKVLPRSQSQEALGQHPKNQFLRPRTHRVAQKEDAYRVKVTYG
DEKTRFRMPKNFGYQDLSGEISRRFNINDMSKFDIKYLDDDSEWVLLTCAADLEECIDVCHSSQTRTIKLSLRVS
NHCMRRRW
>Cercis_canadensis_Cerca306S07940
MLFSGNIGNEYFAVDWAYEFFTQDNISDAFPLMGSFPDPYETLAIEPTSSLKVADDDFYAIKNGLAVWNENDVV
FDSDKQTLLLCKNGENGKEMQEDRKVKRCREERMSSTRMLSRKTISEYFYMPITQAAKELNVGLTLLKKRCREL
GIRRWPHRKLMSLQTLIKNVQEIGKEEGQESEEKLRNAIEILEKEKKLLEEKPDLQLEDYTKRLRQACFKANYKKR
KLMGMEMGSMDLLQCSSLCGHERRDEDVYINDEKEQDINSLVPDAHSSYNIMF
>Chamaecrista_fasciculata_Chafa97S13327
MSIDGVVSSGTLLERPSDSSMDFTYMDELFLDGCWLETTTDASGFLLQSPSSCNPHLDHSFSWPALEVEERQS
PLFNEFQETQERINNNNTGEVCNQSENHSDENSEVVTTRWWIGPMANLNPQLSVLERLIKALVYIRDFNKDKD
MLIQIWVPINNKAGRKILTTSDLPFSLETRCPNLAKYREISEKYQFSAEEDCKVLVAGLPGRVFIDKVPEWTPDVR
FFRSDEYPRVDHAQEYDVRGTLALPVFERGSRTCLGVIEVVMTTQKIKYFSELESVCKALEAVDLRSSKLSSMQN
LKACNNSYEAALPEIQQILRSACEMHKLPLAQTWVPCIQQGKEGCRHSDDNYLHCISPVEHACYVGDPCVRGF
HEACCEHHLLKGQGVAGGAFMTNQPCFSTDITSLSKTDYPLSHHARILGLGAAVAIRLRSIHSSVDDFVLEFFLPV
NCTDSEEQKKMLTSLSMIIQRVCRSLRVITDKELEEETSISMDEVIASADSRSAKMELQQSRTDEKASETGNDRQ
LSDLGQQEESISKGNLDYVAECSTIGGEGSLPIVGVSKMGDKRRTKAEKTITLQVLRQYFAGSLKDAAKNIGVCTT
TLKRICRQHGIKRWPSRKIKKVGHSLQKLQLVIDSVHQGASSAFQIDSFYSKFPELASPKLPVSSSLFSTWKQIDN
NNNPNPSSTCLQSPEAPSSSCSQSSSSSHSCSNTPEQHHQHQHYNTQSVDGIKDHHHASVGEDCENGVLKRIT
SEVELKIFSQDKSELNLRRCQSHETLGQLPKTALKGSSGTHKTPDQAQSQRVKVTFGEEKARFRMPGTWGYQH
LLREIATRFNIDDMSKYDLKYLDDDSEWVLLTCDADLEECIDVCQSSQSSTIKLCLQVSRTLGLHDCKS
>Chamaecrista_fasciculata_Chafa5640S24365
MECPSPPMGKPIGYWTSSRALLEGPTPFDCVMSNSYSEDTRDSFSEPINPDTYAACWSNSPSMIDQILANEFSS
FYSIPYISPGEFDLVDQSNGSFYMADVGANCNALRSSSSPGEKAVIQQTDAQLGFLDDANYLSSKQQMNGSFQ
QLNTSDTDNYTMYRPPVCSLDERMLRALSFFRESSGGGILAQVWVPIKLGDEFILSTSEQPYLLDQMLAGFREV
SMTFKFSAEGKPGSFPGLPGRVFISKVPEWTSNVGYYNKTEYLRVEHAINHKVRGSIAVPVFDHHSEMPCCAVL

ELVTMKEKLDFDRELGIVCDALQVANLRTIMPPRLLPQCLSNDKRAILTEIMDVLRAVCHAHRLPLALTWIPCCYI
DGHRDETMRIKIKEGYFNSGDKCVLCIEESACYIKDKVMDGFVHACVEHYLEEGQGIAGKALRSNQPFFYPDVK
AYDISEYPLVHHARKYNLNAAVAVRLRSTNTNDDYILEFFLPVNMRGSSEQQLLLDNLSCTMQRICKSLRTVSDA
ELSGIVGSPVRFQRKQVSGVVPKSRKNSKIASTDGDPDSVKKMPLKTSNLRKLDYEAAHNQEMSGSRRQVKQK
RSKTKKNISFDVLQQHFSGRLRDAAKNIGVCSTTLKRICRQHGISRWPSRKISKVNRSIKNIQTMLDSVQGVKGG
IKFDPSQGSFVAGGSMIEEFDARSNLQFCEKVTPVEDPRQDGFVRRHSSSLVAGEMENGGVPEEHNHITSSTM
TDSSSDSSSSEVSASSWSGSDNIENQKHSKAKSMMGLDGGVYKIIMKATYREDTIRFKFDPSVGYLQLYEEVATR
FKLQSGSFQLKYLDDEEEWMMLVDDSDLQEFTEMVDDARRRSVKLLVCDMPCVSETSLQATKRDTL
>Chamaecrista_fasciculata_Chafa8161S27192
MSESEEENNYVLLPPKSKPATEEPGSVMDFDLDLESSWPLDQLSFISNPFSPFLVAASSEQPSSPLWVFSDVDDD
KPARLAASAFSDCHRLLPCNSNTVAEKPAESEKQKLPSPSVALPPLENLDGYCLIKERMTQALRYFKELTEQNVW
APVKIGNQFVLTTSGQPFVLDPHSNGLYQYRTVSLMYLFPVDGENDGIQGLPGRVFQHKLPEWTPNVQYYSPR
EYPRLNHAQNYNVRGTLALPVFEPSEQSCVGVLELIMTSQKINYAPEVDKICKALEAVNLKSSEILEHPYTQICNE
SRQNALAEILEILTVVCETHKLPLAQTWVPCRHRSVLVQGGGLKKICSSFDGSCMGQVCMSTTDVAFYVVDGH
MWGFHEACAEHHLQQGQGVGGRAFLSGNMCFCGNITQFSKIDYPLVHYALMFGLTSCFSICLQSAHTGNDNY
ILEFFLPSKITDFTEQKTLLESILATMKQHFQSLKIAAGFELEEEASIEIIEATNEKVYSRLDCIPLTQPSKLPPGPDALP
NMERMPPLDCSEQQIMYYDDINGGANLGDNAGGNFDQISSLETKTVKKPSERKRGKTEKSISLEVLQRYFAGS
LKDAAKSLGVCPTTMKRICRQHGISRWPSRKIKKVNRSLSKLKRVIESVQGAEGAFGLNSIGTSPLPMPVGSNAE
PSTPNKLSPQQTKENELDATKVSDTNRQARVEHGLPGGGRVHSPEAAIHDKSGSSQEFGKRRKGSRTKSGSSE
DSAHPASHGSCHSSPQNESSPSKDIFIATNGEQCVAVGGSPELKLQPISKLNSPPAYPIPDLVVTELQKPFGGMLI
EDAGSSKDLRNLCPSADANLEDQVPEACGTNPPCSDLGPKQCPDTLSHTVLPFAAMREMKSVIIKATYREDIIRF
RLSLASGIVELREEVAKRLKLEVGTFDIKYLDDDQEWVLIACDADLQECMDVSRSSGGSIIRLLVHDIASNHGISD
TNRQARVEHGLPGGGRVHSPEAAIHEKSGSSQELGKRRKGSRTKSGSSEDSAHPASHGSCHSSPQNESSPSKDI
FIATNGEQCVAVGGLPELKLQPISKLNSPPAYPIPDLVVTELQKPFGGMLIEDAGSSKDLRNLLLGIVGA
>Chamaecrista_fasciculata_Chafa93653S04837
MEISPWEDFGEMSLVPFSYPSNPLCFNQFPELENFTSDFKTLDNYNLPPFPESIVFEEKPKPLAQGYFLPANSQT
HIHLPSLMGETSASSIMNNGFGSCVKYVKEEQNDENQRMNSMTTLRKNKTTSLEFNEIRKHFDVPITKAAKRM
NVGLTLLKRRCRELNITRWPHRKIKSLKTLIYNVKEMGLTSEVEMLEKHKRLVEGRPDLELDEKTKKLRQACFKAN
YKRRRLLAFQS
>Chamaecrista_fasciculata_Chafa93653S28208
MEISPWEDFGEMSLVPFSYPSNPLCFNQFPELENFTSDFKTLDNYNLPPFPESIVFEEKPKPLAQGYFLPANSQT
HIHLPSLMGETSASSIMNNGFGSCVKYVKEEQNDENQRMNSMTTLRKNKTTSLEFNEIRKHFDVPITKAAKRM
NVGLTLLKRRCRELNITRWPHRKIKSLKTLIYNVKEMGLTSEVEMLEKHKRLVEGRPDLELDEKTKKLRQACFKAN
YKRRRLLAFQS
>Chamaecrista_fasciculata_Chafa1046S13677
MADPRSIVPYHDPYDFPLPRSIYDIGNDQNPTLADLSSSSLPQPLHAIYQQGSDNAPNPLIQDTLHNFSHHQAG
PSQVHEETHTGQDYSGSQNFAEVVQLSFWPEPPVPFICSCCQVLREIVHTNGLMFTKLEIHGRLGMICHAIQQ
KSNITNAASSTDHQIHISIEEIKNFLTKYWMEQNAAGYFVVQDPFSAYYEALCVGMDWTEDIKDELVDMNVDN
NGGTSDDAEDPEKNPKATCLATQRERAGKMTLSDFSDYFHLPIEEAAKEVNLCPTVVKKICRKAKLARWPHRKI
KSIVKKINILRGTLNAEDAMAKARNEAEIARLRQEMIECCGGVVPTALNLD
>Chamaecrista_fasciculata_Chafa2145S18426
MHHRTPKPNTNSLEQHKDWEMQHQQEEYMYGGGEVVENCGYGRLCEAIPEAALIDELLVQGCWVEASGVIS
HHVNQQQYMSHQNNSQKKWWIGPIPSSSVKERLLVAVAYLRHYYYYTNISNIQIWVPPSPTNLHPHYAYDDHE
ANTEDVTSVFLPKLLQQYYWITPINNVRFFRTHHCYPHSINYKDDGGAASLPVFQRGSGACLGVLQILMDHQS

NNINYPPQPQLLPPCNHAVELKSGHQNLIPPAATNNKVTNWEVERGAIIEEIIEILRSVWKTHNLALVLTWGPCI
QQGKSGCSSEECVSTVDCACLVGDVDIVGFQEACSEWHLLGRQGIVGTALTTTKPCFATDISGFSRAEYPLALHA
TMFGLHAALAIPLYFRTNTATADFVLEFFLPKDCRDSEQQREIVKSLCMVVHQAACRSNLHVEVVGFEQDHDD
HELMKEKQTESAVVSSIWQLEEAKQEFKLTTSSRKSGDKRRTKAEKTITLPLLRQYFAGSLKDAAKSIGVCPTTLKR
ICRQHGITRWPSRKIKKVGHSLKKLQLVMDSVEGVEGAIHIGSFYSNFPQLTTTSSASWNHHNNSQSSNSNNIIT
NTNTINYNYGNEGSTSLTKSKSSPSSCSGSANYNNAEDAGLNRSHHMYTESFSFRVKASFGDENIRFSFPPNWG
LRDLQMEIARRFNLNLNDVLTNVDLKYLDDDGEWVLLTCDADFEECKDLHLHTTPSSSHSHTTLTIRLSLFQPPA
LPSNLPNSLHQLYQYHF
>Chamaecrista_fasciculata_Chafa10125S05853
MAWTSKYELDEVVFPSYDLWGNSNGYFGLDYWPYGSALPWMEPDILYETLAIEPTLPSMQDNEFYDIGKGLA
VWNEIDAGFDSEKQPVFLLCKNEESGMMEMKEENGNEMKRSCREERGCSSIKLLSRKTISQYFYMPITQAARE
LNVGLTLLKKRCREVGIRRWPHRKLMSLQTLINNVQMLGKEGGEGSEEKLRNAIELLEKEQKMMEEEPDSQLE
ENTRRLRQACFKANYKKRKLMGSIGYHTSTSGDQYCTTNECYYNEQIQDMNNVLACFN
>Chamaecrista_fasciculata_Chafa4076S22354
MEEYDGAFESLSESAASVSSEVSLVMDFIDDLLVHGCWLETSSYINNNFNNSNNNHLLPFTNCIIPSSSSSSSFTT
TSHYYPNNNYLQHYCFWTSSDNSSSSSTTSSEIYHHQHHQQQQLESFVLENITTSNTNTNNDELLVGKRWWIG
PKANPGPSSSVKERLVLAVGCLKELYYTTTNNNNNNNNTANKNNFMIQIWVPPHHHEDYYRKLHHHNDYYGG
GGVVRFFRSHEYPYANYLVNGSQQYYDNVRGGPCLALPMFERGSGTCLGVVEILLTHHHANHHNNNNINNIN
SNLSHNLQAVDFRSCQNFIPPPMKVFDEWYQAALNEIVDVLSTVCKTHNLPLALTWAPCVQQNKTGCPHNTN
NNNNNNTSSSDDQIHQNNNNYMWCVSTVDSACYIGDLDLLGFQEACSEYHLFRGQGIVGTAFSTAKPCFATDI
TAFTKPEYPLSHHATMFGLHAAVAIPLRSSVYTGSSDFVLEFFLPKDCHDIEQQKQMLNSLSIVVQQACFRSLHV
VSSQVVDNQEAEFMFNNNNVREILKGSSGIHKEETLDFGSQSSEEHPSSKESSWIAHMMEVQNQQKGKGVS
VSLEYLEEPKQEFKVTTNWDISGSHHGGLCHNGQVFSSDFGQVHQQQQSSASRATVEGGGESYSYGGRRSSG
GRKSGEKRRTKAEKTISLPVLRQYFAGSLKDAAKSIGVCPTTLKRICRQHGITRWPSRKIKKVGHSLRKLQLVIDSV
QGAEGAIQIGSFYSSFPELSTTDLSSGVPHSSQSPSNNISDHSKKTHHHHHHQHHHTEITNGLLKSPPSCCSQTS
TIINNNAINNNGDIIMTENPEALINSRVHTQNHALIEAELLEDAKHNHNHHHLEALPPLPAEITTHHGGAFRVKA
TFGDEKIRFSLQPNWGFRELQMEITRRFNLNNNINHDVMMNNFDLKYLDDDGEWVLLTCDADLEECKDIYRS
SQSRTMRFSLFQASPMNLASDTFGNTSSSPS
>Chamaecrista_fasciculata_Chafa571S24484
MEYPFSHKEKDTEHSTSQAQLEGSTSLDGGERNLISEDMLNTFSELMNFDTYAGWCNNSSATDPVSDNGLSSF
TSVPYASPDRFNLMEQSSGPFFMNDVNRNYNGIRSLPNSGEKLVCQQMDTQLGFSDDANDADNFNSKQHIN
HSYKQPDNLEVGNNMIPSPPGWSLDERMLKALSLFKESAGGGILAQVWVPIKHGDQFILTTSEQPYLLDQMLA
GYREVSRTYTFSAEGRPGSFPGLPGRVFISKVPEWTSNVGYYSKTEYLRVEHAINHEVRGSIALPVFDPHSEMPC
CAVLELVTTKEKPDFDRELEIVSQALQVVNLRTTMPLRLLPQCLSNNKRVALMEIIDVLRAVCHAHSLPLALTWIP
CCYTEGTSNGTTRIQIREGHLSFHEKFILCIEESACYVNDRPMEGFVHACAEHHLEEGQGIAGKAIQSNHPFFYP
DVKAYDIVEYPLVQHARKYNLNAAVAIRLRSTFTNDDDYILEFFLPINMRGSSEQQLLLDNLSGTMQRICRSLRTV
TDVELSGISGSQVDFQNEKVHGFLSTSRRNSPIELTDGEHDLVSGISLKAPNLRNDGIEASQSQAVSGSRKHVEK
KRSTAEKNVSLSVLQQYFSGSLKDAAKSIGVCPTTLKRICRQHGISRWPSRKINKVNRSLKKIQTVLDSVQGVEGG
LKFDPSTGGFVAGGSIIQEFDVHSNMLFPEKSMHMQDPESVAKDGVSVPTLPCDEGENSSIKLEDNDVYMEG
NRLMNLRDVLIPSCDERELKKANVFSVDFNDDSKLMTLDDAQYCPEKACLGSVPANEGKRKGLNKDGLGVKIN
SRDVVSWSSGSLIADEMENVVDEDNVEHNHVTSSSMTDSSNGSGSMNHGSSSCSQSFENQKHSKVKSTVVD
RGSKIIVKATYKEDTIRFKFDPSAGCFQLYEEVATRFKLQNGTFQLKYLDDEEEWVMLVNDSDLQECLEILDDVGA
RCVKFLVRDMPCFLSSSGSSNCFLGDS
>Chamaecrista_fasciculata_Chafa95053S28562

MEDGVTKPAVTTTPEPPPEPSSMDFDDVNELFFDGCWLETATTGSDFLLNTLSSSNPLFDSSSSWPAMDSNGG
FSRQKEYSKEDIMKQSQSENNSVEGGEELEEVRRWWVGPRASPGPASSVMERLIRALVYIKEQNKHKEDALIQI
WVPVNKEGRRILRTTDLPFSLETKSPNLAKYREISEKYEFSGEQGMASGLPGRVYVGKVPEWTPDVRFFTSDEY
PRVQYAQNYDVRGSLALPVFEQGSTTCLGVIELVMTNHHIKYRPQLETLCKALQAVDLRSSNISSMQNVKLQGL
GCNKSYEGVIPEIQEVLRSACDMHKLPLAQAWVPCLQQGKEGCRHSDDNYLHCISPVEHACCVGDSSVQGFH
EACCEHHLFKGEGIAGQAFLTNQPCLSTDVTSFGKTEYPLSHHARMFKLRAAVAIRLRSIHTTKDDFVLEFFLPLS
CTDSDEQKNLLTSLSSIIQRVCHSLRVISDEELEEEANILVHEVIAPTDSFTSAAMMEINQSGMVALSGTQEKSSQ
TMGRKLSEPMQKQEVSMLGGNLDGEFSTHGDNSLSSVSISKMGEKRRAKAEKTITLQVLRQYFAGSLKDAAKN
IGVCTTTLKRICRHHGIKRWPSRKIKKVGHSLQKLQLVIDSVQGVSGAFEMDSLPKLASPILSGTSLFSTYKLSDQ
QNPSSTQCVPDLLSTEVAASKSPSCSCSQSSISNHTCTSVSEKINVGPGNDGVLKRVRSEAELKCLSEERAKFLPR
SQSQETLGIDPKAECYLPFLKTGPEATQKKDAHRVKITYGDEKARFRMPKNWGYEDILQEIAKRFNINDIGQFDI
KYLDDDSEWVLLTCDADLEECIDVAQSCGSSTIKLRIQLSNHCMRRSRAPS
>Cicer_arietinum_Ca_02703
MEYPFYHTGKGIGYWQSPGTQLEGSTSLDGGISNLVSEDMPSSFSELMNFDNYAGLCSGPSMTDQIMANELP
ALASVLYQSSDGLNIVEQNSGQFYMTEVGGNSNNPQSSPIYGEKIVCQQMDTLLGFLDNNDANNLSSKQKING
SLQHVNTFDTGNCVIPKPPALSLDERMLKALSFFKESAGGGILAQVWVPIKHGGQVFLSTSEQPYLLDQMLAGY
REVSRTFTFSAEGKPGCLPGLPGRVFISKVPEWTSNVGYYNPSEYLRVEHARNHEVRGSIAFPIFDMHSGLPCCA
VLELVTTKEKPNFDKELEIICRSLQLVNLRTNVPFRLLPECLSSNKRAALTEIVDVLRSVCHAHRLPLALTWIPCFYT
KGTRDETTRIQIKEGNSSSREKNILCIEESACYITDRVMEGFVHACVEHHLEEGKGVAGKALQSNHPFFYPDVKA
YDISEYPLVHHARKYSLNASVAIRLRSTYTYNDDYILEFFLPINMKGSSEQQLLLDSLSGTMQRICTSLRTVSEAELS
GIKSLQVGFEKKNDPRFPPLSTQNSQIPSIKENNGSVQKLSLKASNQRKNGNEPSCNQETNGPRRRVEKNKSTS
EKNVSLSVLQQYFSGSLKDAAKSIGVCPTTLKRICRHHGILRWPSRKINKVNRSLKKIQTVLDSVQGVEGGLKFDP
SMGAFVAGGSTIQEIDEHESLFFPEKSTAQDPQNLENKLEGKLKETNSSSVDCSEDSKSMAMDDCHEQACFGS
VLGKSDKLVLNKGGLRIEKCKHNNTSSFFVDEMDTCVDGDDEVVEHNNPTSSSLTDSSNGSGSMIHDISSGYED
FENQKHCKGKSTIVDGGSKIVVKATYGEDTIRFKFDPSTGCFRLYEEVAARFKLQNGTFRLKYLDDEEEWVMLVN
DSDLQECLEILNDMGTRNARFLVRDVPCVLSSSGSSNCYLGGSS
>Cicer_arietinum_Ca_09832
MEYGGGLVEDCGGFGTMVGGETDMIEELLVEGCWVEASENNLMMMHHDDHMSSSTTPQPHYIPMGDND
EESGNFVVGKRWWIGPQANPGPSTSVKERLVLAVGYLKEYTKNSNSNSTSNSNSNSNSNSNVLIQIWVPLRRR
SGLFHNQYHNPNPVHESLPISVPNSNVQVRFFRSHEYPRVQAHHGSLLALPVFERGTATCLGVIEFLIANQNLIN
YRPQLDHLSNALEAVDFTSNQNMIPPAVKVFEELYEAAVNEIVQVLASVCKTHNLPLALTWAPCIQQEGGGRGK
GGCSNSNGNSNSNSNGSNNNMMMSCVSTVDSACYIGDVDVLGFQEACSEYHLYNGQGIVGTAFTTTKPCFAI
DITAFSKAEYPLAHHANMFGLHAAVAIPLRSVYTGSAADFVLEFFLPKDCLDTHQQKHMLNSLSLVVQQACRSL
HLHVVMEDDHHNHNHHHLHHQHQHQHQHEEDQFTFPTTNIYMPSVDAAAAAPVVVLSQVEADASASASA
CSTKETTSSSCSWIAHMMEAQHKGKGVSVSLEYLQEPKEEFKVTTCNWEREREGNSVFSEFGQVLQQQQHEH
QSMSNSRASTVVSVEGGEESGGGGGSGGGGRRSSCSSNGRKSGDKRRTKAEKTISLPVLRQYFAGSLKDAAKSI
GVCPTTLKRICRQHGITRWPSRKIKKVGHSLKKLQLVIDSVQGAEGAIQIGSFYASFPELSSATGHSDQSSMKLM
HNHGVSDHHNSFYGDGGVTTSNLKSPSSACSQTVGNQTNSTLINNNVVLMTESNALASTDHAHAHAHAAAL
MQIRRTHSEAELLHHHTSNIDIQDYQLHDQDTKQLLLHFNKSQTLPPRPSTTTTTNGYNNSLERGAFRVKATFA
DEKIRFSLQGMWCFRDLQVEIARRFNLNDMNNLVVKYLDDEGEWVVLACDGDLEECKDLHTSSHTRTIRLSLF
QASPLNLHPNTFRNTSSSPPSSS
>Cicer_arietinum_Ca_04456
MAESEEENQEFLPKTKTSSLEEHGCCAMDFDLDLETSWPLDHMSFVSNPMSPFQFSTISDQPSSPLWAFTDGE
DDKHVKLAASALSDCHKIFSSDSNSITEKPVENDENKKHLPPLVPPIENLNGYCAIKEKMTQALRYFKEWTELNV

LAQVWAPVRNGNRYMLTTSGQPFVLDDPHSNGLCQYRTVSLMYMFSVDGENDGTLGLPGRVFQQKLPEWS
PNVQYYTSKEYPRRYYAQHYNVRGTLALPVFETSLQSCIGVLELIMTSPKINYAPEVEKICKALEAVNLRSSEILDYP
YTQICNEGRQNALSEILEILTVVCETHNLPLAQTWVPCRHRSILAHGGGLKKSCSSFDGSCMGQVCMSTTEVAL
YIIDPHLWGFREACVEHHLQQGQGVAGRAFLSQNMSFCTNITQFCKTDYPLVHYALMFGLTSCFSICLRSSHTG
NDYYVLEFFLPPNITEFHEQKTLLGSILAIMKQHFQSLKIAAGVELGEDGSIEIIEAMDESVHIRIESIPIVQSIKSPPR
PDASPNMEEDKVPQDPSDIQGENIGGNIDQVSFLGNKNVKKPSERKRGKTEKSISLEVLQRYFAGSLKDAAKSL
GVCPTTMKRICRQHGISRWPSRKINKVNRSLSKLKRVIESVQGGEGAFDLNSLNTNQLPIVSSFPEPSTPNTSNQ
QASLSIRPSEPQIKENGIDASKVSETNIEVVMEDQLLGGRKRSLGKEVIINDKGVPVQKVRARSGSSGDSTNPISH
DSCHGSPPIESSTIKEIFIPSNNEQCVALKGSPESRVQPTNGFNSASAYRMPDNVIAELEEPFGGMLIEDAGSSKD
LRNLCPSVAEAILEDMAPEPCGTNLPGSSYLAPKQCMDTINTSTTPFAARKEMKTVTIKATYREDIIRFRVTLTCGI
VELKEEIAKRLKLEVGTFDVKYLDDDHEWVLIACDADLQECIDVTRSSGGSNIIRVLVHDITSNLGSAYESSGE
>Cicer_arietinum_Ca_01656
MVEDQNVNNNFTFGGNNFEVGSTSQIHDNQNQPANDIPVLLNHAEVLALDQWPPAPLPYFCSCCHVLREIIH
ANGIQFEKLEIHGRLGLITHAIHHQTPVNGDAPIYQMVDFCRRSLEEIKIFLAQYCVERNRAGYFILQDPMSAYYE
TLCTGLDWIEDLNMEDPVDANNNNNQNNSDEIVEPQEQEGENGTPTKKNLSVQRKKAGKLTLNDLCDLFHLP
IEEASECVKLCPTVVKKTCRKAGLARWPYRKSSEDMAASDQTLVCVKQVKQELSNEWDESMPLPGDIIEGFSTH
NSDADESFLPVKTSSEFSSQLGKINPRLESIWIKVRRGDSLLKLQTCIVQQKGYVLRRKYTIQATTDHRHIADLGDL
TLEQCTELQVMSSRVVQTQGKGLHIDRIKYDWKMKVGTYLPHQSSSVVSSILFTPLAGEHCINTTTARCMAWF
SASISSGVPLVFVNIQTELIPPTIEKTNLSNMQQKHYTAQIIHGIRLWFLPGLEEIPIELIPEPNEVRFGMEINRTEEG
FVCIYSVTGGTSADRGGLKELHEEATANGFLLVISRLEGKSKMPTSVCSDGLIHCCDHAEMKDLLVSAIDQYEIIHL
HVMAWPNQTRSSPTQVVGFAALLPPQRSFSTHHYD
>Cicer_arietinum_Ca_03992
MDFFTEHQITQGSNFFYNAQPQQIDTPEDKIMDISPLDGFVDFNWETEFQRNELYISDEFKDLENFNFDFNLP
HLEQDFEVDQKPLNAVLPQLEHCNDEGVSGFIENGFVSCVKKEEEEEEKEKPLSLALLSGCSSKKKRSSLLEFDDI
KKHFNVPITLAAKRLNVGLTLLKRRCRELKITRWPHRKLKSLVLLIDNLKKMGLADEVVKLEKHKKMLEKLPGIEL
NEEIKRLRQACFKANYKRRRLIGSRP
>Cicer_arietinum_Ca_04834
MEDGALTSSTTMMEAPQPDENSMDFDYVSELLLDGCWLEASADGSDFLLQSSPFSNPLFDPSFTWPSLDNID
PPDNVDRHKSQDASLGTEQESRSIVNVDGGSNQKEYRFETYSGEGFFSRWWIGPTPNSGPGGSFIVEKLIRALK
WIKDFDRNKDMLIQIWVPVNRGDDRPFLTANDLPFSLETRSLNLARYREISVRYQFSAEEEDSKDLVAGLPGRVY
RDKVPEWTPDVRFFRSDEYPRVDHAQECDIHGTLAVPVFEQGSRTCLGVIEVVMTTQQSNYSAELETVCKALE
VVDLRSSRHSSLQNVKARDRSYETALPEIQEVLRSACEMHKLPLAQTWIPCIQQGKDGCRHSDDNYVHCISPVE
YACYIGDSSVRFFHEACMEHHLLKGQGVAGGAFMTNQPCFSADITSLSKIDYPLSHHARLFGLRAAVAIRLRSIYS
TSDDYVLEFFLPVDCNDSGEQKKMLTSLSMIIQRVCRNLRVITNKELEKNNLSANEVMDLADSGFASGATWSEL
QHRRMAASLDGDFFFNETMGGKFSEQTQQQENLILKGDTESGRECSLSVEGNLLSSVGIREKRRPKADKTITLQ
VLRQYFAGSLKDAAKNIGVCTTTLKRICRQHGIKRWPSRKIKKVGHSLQKLQLVIDSVQGASSTFQIDSFYSKLSD
LASPNLSGTSLISTLNQIDNQVSLNTQPDPDSLSPEDASKSPSTSCSQSSFSSHSCSSMSEQQHHTNNFASNKDP
LVGEDSVDVVLKRIRSEAELKSLIQDNNKANVMPRSQSHETLGEHPITEYHQSLLKTDRKASQKEDAHRVKVTY
GDEKSRFRMPKTWCYEDVVQEIGRRFNVSDMSKFDIKYLDDDCEWVLLTCDADLEECIDVCQSSEISTIKLCLQP
SSNFIRSSLEFR
>Cicer_arietinum_Ca_13679
MKSSTTSEDMFNNISELMNFDTYAGWCNGSSSVNDHDHSLVHEFSSFASIPYSPHDGLNLVEHINGPFFMTEI
GGGNYNDMDNSTSYGGNYQLEFLDTNNSNETNNSDSQQGQNGSFQLLNSNNSSDMCNYLIPKSPSWSLDE
RMMSALSFFKESAGEGILAQVWVPVKYGDEFILTTSDQPYLLDQKLAGYREVSRLFTFSAEMKMGSCPGLPGR

VFISHVPEWTSNVGYYHKSEYLRLEHAISHDVRGSIALPISDMLSEVSCSAVLELVTTKEKLNFDKELEFVSHALQR
VNLRTIMPPRLLPQCVSINKRAALTEIIDVLRAVCHAHRLPLALTWIPCCYSEGKGEESERIRIKEGHTSSDEKCVLC
IEESACYINDKMVGGFVHACADHHLEEGQGISGKALQSNHPFFYTDVKAYDVSEYPLVHHARKYSLNAAVAIRL
RSTYTNDDDYVLEFFLPINMTGSSEQQLLLDNLSDTMRRICKSLRTVSEAELRGIQGSQDGFQKENVSGFFSLSR
GNSQIAFTTGDQDLFQMSLNATNLKNIQGHCQATNAPRKQIEKKRSAVEKNVSLSVLQQYFSGSLKDAAKRIGV
CPTTLKRICRQHGISRWPSRKINKVNRSLKKIQTVLDSVQGVEGVLKFDPHTGGFVAGGSIIQQIGSHKSLMFPE
NFFAQDLAPAPITTREPISVTPPPCENSAIKLEDGLKKENASSVDCIQDSTSITMHDEPCQTSWIWTKTQDCPKQ
TSIGSVFEKQDDQCGLNKTSSFSLIEHELGGDENNQPTSSSMTDSSNASGSMVRRSSSGSQQSFENRKRSKMK
SVCVDSESKFAVKANYKGDTIRFKFDPSVGCFRLYEEVTTRFKLQNGSFTLKYLDDEEEWVMLVNDSDLKECVEIL
SDVGTHCVKLLVRDIHGSIVTSNS
>Cicer_arietinum_Ca_13151
METMPLIGWNSYTDENPFSFTTYQCFPYSGNSNEYFPVTFPKEEFPNDSYLDSFSVEYCNYIPDPYQSLCIEPMS
SSLQDYDFYDVEKGFFGWSEIDAGFESEKVYLNFTDEKIVNEVKEELVDVKSGGEERKCSNAKNLSRKIISQYFYM
PITQAARELNVGLTFLKKRCRELGIRRWPHRKLMSLQTLINNFQDQMKEGGLESAEKLRYAIDALEREKKVVEE
MPDMQLEDGTKRLRQACFKANYKKRKLMEDPQMF
>Cicer_arietinum_Ca_27191
MEYFCPEFLYETFPTESPTPISVQDYDFGDAKNVFMWNELDAVFYSDKHRESSAREISKGKRCKEENSTSARML
SRKKISEYFYMPISQAARQLNVGLTHLKKRCRDLGIQRWPHRKLMSLQTLIKNVQEQGNENDEKIRNAVEILEKE
MKMVEEKPDLQLEENTKRLRQACFKANYKKRRLMVMRLMDQ
>Datisca_glomerata_Datgl488S31230
MDDGFLSPGTMLDGPSDSAMDVDYMDELLLEGCWLETDGSEFLPQTPFNSNSLFDPFFGWPPVNTGDLSTN
PAPNTDQHEQERQVLNDTTPFLSQNAIDVAGCSGQTENQIIEGSEVGRRWWIGHPGPGCSVTERLIRAVGYIK
DSSRDKDVLVQVWVPINSGGKRVLTTNDLPFCLNTSCTRLARYRDISMSYQFSAEENSKDVMGLPGRVFLGKV
PEWTPDVRYFRSDEYPRVRYAQQCDVRGTLALPVFDQGSRTCLGVIEVVMTTQQIKYGAELENVCKALEAVHL
RSSDALSSPHVKVFYRSYGAILLEIRKVLRFACKTHRLPLAQTWASCIQQGREGCRHSDENYNRCVSTVDQACYV
REPGIQNFHEACSEHHLLKGQGVAGEAFLTNKPCFSSDIKSFGNTEYPLSHHARMFELHAAVAIRLRSIHARKAD
FVLEFFLPVGCTDPEEQRKLLSSLSIIIQQSCHSFRIVTDEELREERDLPFAQVVVPLNGTPSKAEGQVGDNPISLFP
DEKPAEVLKAAECSEECLTFGEHSSSNAGMEKTGEKRRNKAEKTITLQVLQQYFAGSLKDAAKSIGVCPTTLKRV
CRQHGIQRWPSRKIKKVGHSLQKLQHVINSVQGTSGAFEIGSLYNNFPDLASPNLSGTSPFLTLNTGTAAGDGII
NPKVAPSKSASSSCSQSSGSSSLCFSSRTHADAVIGENPGEGELKKVKSEAELNVSIQEGIEIPRRSQSHKSLNELR
PGMKNNRQMSEEEDAQRVKVTYGDDKIRLRLHRGWGYEDLRAEIGRRFNINDMSKYDLKYLDDDSEWVLLT
CDADMEECIDVYRASQSQTIKLSLQVSRFRSSIGSSFLS
>Datisca_glomerata_Datgl229S25120
MSDAEEDNPAAFPFKPKDDQYVRTAEDQGEPIMDFDLFLDNSWPLDQITPFISQPLSPILISVSDQSYSPLWAFS
DGDGKLARYACSVLGASNLPTEESANNHENEVPNPSALWGLKALENPDSYCLLKGRMTQALRYFKESTELHVLA
QIWAPMKDGDRCVLTTSGQPFVLDTQSSGLHQYRMVSLTYMFSLDGKTDGVLGLPGRVFLQKLPEWTPNVQ
YYSSREYPRLSHALHYNVQGTLALPVFEISGQSCLGVLELIMTSPKINYAPEVDKVCKALEAVNLKSSEILDLPSTQI
CNEGRQNALAEILEVLTVVCETHNLPLAQTWVPCRHRSVLTHGGGLKRSCTSFDGSCMGQVCMSATDLAFYV
VDAHKWGFREACLEHHLQKGQGVPGKAFLSHSSCFCTNITQFCKTVYPLVHYARMFGLKSSFSICLRSTFTGDD
EYILEFFLPCHQELNLLGSLFATMKQHFRTLKVASGRIDLNKDEVFVEIIEASMNEGLVSRVEYVRISKPTELHPMP
QVVINEGELNLPKEQLLVDFDYQNYGSNAVHENGSLTPVNFNEIRGKRKTSKRNRGKAEKSISLEVLRQYFSGSL
KVAAKSLGVCPTTMKRICRQLGISHWPSRKINKVNRSLSKLKNVIESAQGGEGAFGLTSLVTSPLPVTFGSSSHPL
QGSNQQNSSSYKPCEPLNKNLISKTHGNKAQPGKAVVNRSKLGSGSGNENSGTQTSHSSCQVSPTKPNLSASF
LDPDALVLLQSNEPFGGMLIEDAGSSKDLRDLICPVAADAGRPEMKTMTIKATHKEDIIRFRVPLSVGTGKLKEE

VAKRLKLEVGTFDMKYIDDDQEWVLIACDADMEECLDIFKSSGSNVIRLLIHDLTVNVGRSYESNGGL
>Datisca_glomerata_Datgl973S36760
MILNWCLLTNQKKGKPNFRMEELCKLNNSKYHQPRKFPALDQDLNCLPGSVFTADRSDDRGIEFGAPGDAGI
MEKKKKRAASEHIAKIALADLVKYFDLPIVEASKNLKVGLTVLKRKCREFGIPRWPHRKIKSLNGLIRDLQEEAKQ
WEQDNKAAMAVAKRQMILESEKESIERTPNMEIQSETKKFRQDVFKRRHRDRALKHLDL
>Datisca_glomerata_Datgl151S21250
MGDGILSPATTGNPFDFFMDFDYIDELLLEGCWLGTTDGSEFPRQIPSNTSALFDPSFGWAATAMETNGNM
NMNPTQNDDHEEGGRPFLNEPHVNNTEKALSLNEYMDDVAGCSSQSEKYTVEGFSDMGRRWWIGPRAYP
GPASSVIDRLIWAIGSVKDSERDKDMLVQVWVPENRGVRRVLATSDLPFSVNTSCQRLARYRDISVNYQFSAEE
DCKEAVGLPSRVFLGKVPEWTPDVQFFRSDEYARVDYAQEYGVRGTLALPVFERGSRTCLGVIEVVLTTQQIKYN
SELKIMCKALEAVDLSSSTILSSPNVKALNRSYQAVLPEILEILRFACETHKLPLAQTWVSCLQQGREGCRHSEENY
INCISTVDQACYVADPHFQSFHEVCSEHHLLKGQGVAGEAFMANGPIFLNDITSFGNTEYPLSHHARMFELHA
AVAIRLRSIHTSKADFVLEFFLPVDCIDPEKQKKMLQSLSIIIQESCKSLRVITDGELREETSLLLDRRELEANVNDAP
LDKNPETASVMHIQEHGGGAVPFSPDKKPEVLTEDSPGFQLQQQNTNFKGFVESIEEECSTFCEGSLSNASMG
KTGEKKRVKAEKTITLQVLRQHFAGSLKDAAKSLGVCPTTLKRICRQHGITRWPSRKIKKVGHSLQKLQLVIDSVQ
GSSASFQIGSLYTNFSDLASPNLSRTSPFSTSVKGDHQTASNIQQDGSMINARTAASKSLSSSGSQSSSSSQCCSS
RAQQNHPHWNDIEDRVVGENSGVGQLKRARSEAEQNTTNQGLENRLLRCQSHKALCERPSPENLVQISKSSG
RLSEEEDVQRVKVIYEDEKIRFRMANSWGFKDLLMEIARRFSIQDIGKFDIKYLDDDSEWVLLTCDADLEECIDVY
RSSLSQTIKLSIQLARHHARSL
>Datisca_glomerata_Datgl3S29876
MPEPEVDNSAAFPLKSKDYLVVRGAVDERDTLMDFDLDMDSSWPLEQMSSFISNSMSPILISTTSDQPCSPLW
AFSDGDDDGDDKLPAYACSMPSTLNSNLVTQKPTENHPTRNLPIPSPLWGLTPLENSDGYCLIKERMTQALRYL
KESTDQHVLAQVWAPVKSGGRYVLTTSGQPFVLDAQSNGLHQYRMVSLMYYFSVDGGADGVLGLPGRVFQ
QKLPEWTPNVQFYSSKEYPRLNHALHYNVQGTLALPVFEPSGQSCVGVLEVIMTSQKINYAPEVDKVCKALEAV
NLKSSEILDHPNTQICNEGRHNALAEILEVLTVVCETHNLPLAQTWVPCRHRSVLANGGGFKKSCTSFDGSCMG
QVCMSATDVAFYIVDAHIWGFREACLEHHLQKGQGVPGRAFLSHSSCFCRDITQFCKTEYPLVHYARMFGLKSC
FAICLRSTFTGDDEYVLEFFLPPSITEYSEQQDLLGSLLVTMKQHFHSLKVASGIKFEEGEGLVEIIQASTNGEFEPKL
EYIQIPHSEKLSPRSKEAPIAGQLGALNLSKQPSGVVGYDVVNYGSNGHDLGSQNPASFPQNTDSKKTSERKRG
KTEKSISLEVLQQYFAGSLKDAAKSLGVCPTTMKRICRQHGISRWPSRKINKVNRSLTKLKRVIESVQGAEGAFGL
TSLATAALPVAATSNSPPLAPDGSNNQNSPAYKLYDTTAEIKESSLNSKSPGNDVQAEMEDQLLRGRSIMEELILQ
QNGFLPKFTKGSNRSKTGSGSREESIGTPTSHGSCQGSPANESVLAKDPFVSPHHDSPEFTFRPATCPIPGIVVVT
TEPEESFGGMLIEDAGSSKDLRNLCPSVADAVADDQVPYWPNPPDMAPIQTIDAAAAHAMPLATSRQETKR
MTIKATYKEDIIRFRIAVSCSIVELREEVAKRLKLEVGTFDIKYMDDDQEWVLIACDADLQECMDISKSSGCSIIRLL
IHDLTANLGSSCESTRD
>Datisca_glomerata_Datgl206S24145
MSSSQQQNLDKGTSINPSSQPHRPLSFDHISEYFSLSLSDAASKLGVCTSVLKKICRDNGLDWWPYRKFLSGKSI
EDIKRYAARERYKVIARARQSKIGGRSENTQIQGVMSQNLQQQQGSKKPEIGRPQNLPCPNMMKGVLTLDEF
KYGFPSDGLSTASNKWWGSSCPDGYEGIDREGAESDAEDDKKHQFEEAANDGSKSLSVIADNETNESMKDEC
KMDQIDPQGTVLLRAVRERSVEEGRKALKGGFFCGYDVNQLGKRERTLVLQIFRSSIRNEWINSS
>Datisca_glomerata_Datgl1955S23745
MEYGALTVNSTYGNYTETTMELDFMDELLQGRWMDSSNGTNLLQSVSSVRALNNDSSNYVTPHEINTNHLM
INPNHHVYEEDQANHETFLVEEVDQSSSRKWWISPRVYPEPSSCVKERLTLALRYLKDCLTNRDLLIQMWMPV
RRGDKLVLTTHDQPYLLEPSCVSLANYRKVLSGYEFAAEEEGEESVGLAGRAYVGKLPEWTPDVRYFKRHEYPLI
RYAEEYNVRGSLALPVFERGSGACLGVVEIVTTTQRVNYRPELDFITQALESVDLRSFQNFIPPIVKTCDELYQAAL

TEILEVVTSVCKTHRLPLALTWAPCSQQGKGGCRHSDQTYTRCVSTVDSACFVSDLETTGFHEACAEHHLFRGQ
GIVGTAFTTKKPCFAVDVTAFSKTEYPLSHHARMFGLVAAVAIPLRSVYTGPAEFVLEFFLPKECRNNDEQKRML
NSLSIVIQQSCRSLHVVTDNELEEEITEKAKPGPSTLEGDHHCTKDQSSWIAHMMEAQEKGKGVSLSLQYQTDE
AEEEREGFRVTSYWDATQGGGLTNGEVITEFGELQQSSGLKSGDEGGGDSYSFGSRRGGRKAGEKRRTKAEKTI
SLSVLRQYFAGSLKDAAKSIGVCPTTLKRICRQHGITRWPSRKIKKVSHSLRKLQLVIDSVQGAEGSIQISSFYKSFP
ELGSTNVDVNDQFSSLKINYQNQHQAESGLFSHGAPAKSPASSSSQNSIPTRPNHHHMIANVNAFSGGDFLITE
DPVGVMNRGSSEEPFGELMTNNNGCFKVKANFVDKKIRFILQPSWGFRDLQLEIGRRFNLEEMRGVEIKYLDD
DHELVLMTCDADLEECKEIYSSSQNNTIVLSLSWASHQNPAKSYGKSSPS
>Datisca_glomerata_Datgl928S36251
MTSGSCFPALEWHYQLPVQDTPFDFIPLVGSFPFDTALYSSLDIEPVIKTSTTDDIFYGYGDAMGVWQNGMNN
ANGLLQENPVLLCNGIVEEKDDQEKKNNNNSKSLSRKVISQYFYMPITQAAKQLNVGLTLLKKRCRELGIRRWP
HRKLMSLQTLIKNVHDMSKDEGEENQLKLKDAINILEKEKKLIEEVPDLQLFDNTKRLRQACFKANYKKRKLMGI
MMDSQSSSTSNNASVEYTITYGIANGEDDDEEEDEELKSLLSQYSFSSSSIML
>Datisca_glomerata_Datgl191S23501
MEYSTNQFNPNVRKFEFPYNEFDWLPAHEDPSHFSKAWELPPVVDRFYDFNSLPITNRHYGYLDSLPQFESVL
DFPDFSDEKFHLPEDEIESIFRDILMDEKPLNTLFTDSYNENNCGNNNLNNQFGTEVSRMMMMNIQTEYSINS
SKKNNEELGNMRLSCGRKRSAVLELDDIQKHFDVPITKAAKKLNVGLTLLKKRCRELNIMRWPHRKLKSLKSLIT
NVKEMGMTNEIIMLEEHKKLLEKLPDMELTERTKRLRQACFKANYKKRRCLAITNS
>Discaria_trinervis_Distr562S25840
MSESEEEASPFPPKSEDVDEDGDGDREALMDFDLESPWPLDQIAFVSNPMSPLVFSSTDQPYSPLWAFCDGD
SEEKLARHVNSTIADCPRVTSCDSNSVTERPAENEGKRMWTSPLLGLVPFENPDANCLIKEKMSQALRYLKDST
DQHILAQVWAPVKNGGQYVLTTSGQPFVLDSHNNGLHQYRIVSLMYMFSMDGDSEGVLGLPGRVFRQKLPE
WTPNVQYYTIREYPRLGHAQHYNVQGTLALPVFEPSGQACVGVLELIMTSPKINYAPEVDKVCKALEAVNLKSS
DILDHTGTQICNEGRQNALTEILKILTVVCETHKLLMAQTWVPCMHRNILAYGGGLKKSCTSFDGSCMGQVCM
SITDVAFYIIDSTMWRFREACLEHHLQKGQGVAGRAFLTHNLCFCADITQFCKIEYSLVHYARMFGLTSSFAICLRS
THTGDDDYILEFFLPPSISDSRQQQIFLGSLLATIKKHFQSLRVASGTVFEEEVFVEIIQASSEGLDSRIECIQIFQSPE
SPTVPTILPNVGDMVQPDSTPHQLIEDFCAANDGSTAGQGCGSNSHVSFQENKDTKKISERKRGKTEKSISLEV
LQQYFAGSLKDAAKSLGVCPTTMKRICRQHGISRWPSRKINKVNRSLSKLKRVIESVRGAEGAFGLTPLSTSPLPV
AVGSVSQPFTLNRTSSPSSKTCDSFMKKKKSQTSFSPGREGLAGMEEPLLGGVLRPEELIHESIRFSPEIGTGLKQS
KSGSSSREASVGTPTSHGSCQGSPANGSAAKKDYLGSCQGSPANGTTIIKDPVSSIHEQCVKVDGSPESALQPTD
ELLKFPVLCSIPDALVTTESQEPFGGMLIEDAGSSKDLRNLCPAADAILDDQIPEYYWVNPQCSDLAPKQPMGTL
TPTMSHLKPRQEMKNVTIKATYREDIIRFRISLSSGIVELKEEVAKRLKLEVGTFDIKYMDDDHEWVLIACAADLQ
ECMDICISSGSNVIRLLVHDIMPNLGSSCESTE
>Discaria_trinervis_Distr887S28955
MDDIVLSPATVLGAPSDTAMDLDFMDELFLEGCWLETTDGSGFLNQSPPGSNAVFDPLFVWPPLETNSELSTN
PSQRSNQDERPRTPHNDPLERGPLATQSPPQNRIDVEGCSGGSEDQTIQVCEPGRRWWIGPKASPGPASSVT
ERLVRALAHIKDFIEDNNALVQIWIPVHREGRRVLTTNDLPCAIDPSCPNLARYRDVSVNYQFSVEEGSKELVMG
LPGRVFSGKVPEWTPDVRFFRNEEYPRVSHAQRYDVCGTLALPIFEQASKTCLGVVEVVLTTQKIKYCSELESVCK
ALEAVDLRSSEILNTQNVKVSNMYYQAALPEIHEVLRSACETHRLPLAQTWVPCIQQAKEGSRHSDDNYTQCVS
TVDHACHVADPHIRAFHEACSEHHLLKGQGVAGEAFMTNQPCFSADVASFPKTKYPLSHHARMFGLRAAVAI
RLRSIHAGVADFVLELFLPVDCTDPEEQKKMLTSLSLIIEQFCQSLRVITDRELEEESDFSVSEVTVPSNSRLGKTAC
LTEVQQSGTAVSLFPKVKPKEDSGEKLSDSRQLQKDSNDKTVEYAGDCSIIGEGSFSSLGVGKTTEKRRTKAEKTI
TLQVLQQYFSGSLKDAAKSIGVCSTTLKRICRQHGIKRWPSRKIKKVGHSLQKLQLVIDSVHGASGAFQIDSFYTN
FPELASPNLSGGGPFSTSKPSDHRKPSNMHPEGDIFSSQATASKSSSSCSQSSSSSQCCSSRAQQQPPMSNVAG

NEDLMVRENSGDGMLKRVRSEAELHASTLDVKKPLQRSRSHKLLSDHHQKENIPSLPTNNSRGGSQVVGDFQ
RVKVTYGDEKTRFRMHNNWGVKELVQEIARRFNIEDVDQFDIKYLDDDSEWVLLTCDADLEECIEICRSSSESNT
IKLSLQVSRHYLRSCLGGNPSRPS
>Discaria_trinervis_Distr929S29326
MEASLQLYVPKLENQLDFDWLYHEEPSENYFPELPPLPDTFIDFNFSLPIPYHYNYNHIHNVPCIEFYDFEDVISSG
FFLDNVAYSSPCEDFMVDEKPQTTMKNSFTHLENLTHEERGLPSVSNDREEERSSSNGGDQRKKSAGLELNDI
QKHFGVPITKAAKELNVGLTVLKKRCRELNIMRWPHRKIKSLKSLIDTVKEMGLTNEIMMLEKHKSLLEVLPDM
ELTEKTKKLRQTCFKANYKKRRSLQLAYA
>Discaria_trinervis_Distr968S29608
MDSSSTHFLHSLLIFSNTANRELIRSLHVYRLDNGKETEIERLFVFSENGPYVEMAAVPLFKLLKIQVSEVYEGCVN
GLWVCIFAFHAGHRPQTTCIPSILSIFRNPKLKSIPSLANDLQSIFQLSRIVDERQTNNENYHQSKRSLPVLDQDLN
CLPYSVTMSESAEEEQIDQSSPGTVAMKKKRAASDHIARIALSDLAKYFDLPIIEASRSLKIGLTVLKRKCREFGIPR
WPHRKIKSIDSLIRDLQEEKCQQQEDMAAAIAVAKRQKMLESEKESIERKPFLEIKSETKRFRQDVFKRRHRARA
LMSQDLTISDSENPYGLH
>Discaria_trinervis_Distr1227S00421
MENPFSSKEKGFENWESSRAQVENMASFDGGTRNSLAEDMLNNFSELMNFDNYAGWCNSSPAVDQMPAS
FGLPSYPSMSYAPIDALNSTERSIGLLPVAGGGGDFNLERSPLNYGDKTVFQQVDNQFGFSSNSNDANESVTKQ
SGGSFQQNNIMDIMNSIISRPLALSLDEKMLKALSLFKESSGGGILAQVWVPMKHGDQYFLSTFEQPYLLDHM
LAGYREVSRLYIFSAERKEGSFPGLPGRVFISKIPEWTSDVGYYSKTEYLRADHARNHQVRGSIALPVFDSGPDVS
CCAVLELVTTTEKPNFDSEMELVCRSLQAVNLRTSVPPRLLPQCLSRNQKDALAEIIDILRAVCHAHSLPLALTWIP
CCYTEGASEENMTLRIRKGNKSSNEKSILCIEETACYVNDRAMQDFVRSCAEHHLEEGQGLAGKALQSNLPFFF
PDVKTYDICEYPLVHHARKFGLNAAVAIRLRSTCTGDDDYILEFFLPVNMKGASEQQLLLNNLSGTMQRICRSLR
TVSETELTGAGCSNIEFQTGSIQNFPFMSRGNSQLTLSDSDVNFMEGVTLNIPDQKNEGIEADIPLEQALGGPRK
KMEKKRNTAEKNVSLSVLQQYFSGSLKDAAKSIGVCPTTLKRICRQHGISRWPSRKINKVNRSLRKIQTVLDSVK
GAEGGLKFDPTTGGFVATGSIIQDFDTQKNLLFPEDTQSTQNPELIIQHAVSANTVPFIEGEDFVIKLEEDEYCMG
GNIPIRNSCEMESKKLNIPTLNSNGSSKPISIDTGSCQPASFVTRLQNRPEIASLVSYTVKEGKKPSEKNYNLKLEN
SDCHFASRSSSSMAAANEMDAAEDGDDGIVEHSQPTSSSMTDSSNGSGSMVNDCSSGSQSFDERKQPKGKR
NCFDSGFKIRVKATYREDTIRFKFEPSAGYFQLYEEVARRFKLQNGTFQLKYLDDEEEWVMLVSESDLQECLEILD
DVGTHTVKFQVRDMPYGLSSSGSSNCFLTGGS
>Discaria_trinervis_Distr1414S00757
MEYSGAMVPNSTYGNFPNSESDFMEELLFEGCWLESSSGFNFMQSTSNPRSLLLDPSHYMPSFDSSALSQRR
QIYNEDDDDNDEQQESSKRVWIGPRAYPNPSSSVKERLMMAVGYLKDCTRDRDVLIQIWVPIRRGGRQMLTT
CNQPYSLNPDCKSLANYRAVSSGYHLLTEEDSGESVGLPGRTFLGKLPDWTPDVRFFRSYEYPRVSYAQQYDVR
GSLALPIFERGSGSCLGVVEIVMTTQKVNYRPELEYVCQALEAVDLRSSQNFIPPNIKACDELYQAALSEIVEVLTS
VCKTHRLPLALTWAPCVQQGKGGCRHSDENYARCVSTVDSACLVIDLDIMGFHEACSEHHLFRGQGIVGTAFS
TNKPCFATDITAFSKTEYPLSHHARMFGLHAAVAIPLRSVYTGSSDFVLEFFLPKDCHDPEEQKQMLNSLSIVIQQ
ACRSLYVVVDKEPEEAMLPIRETIIPSDVRLGKEESQKLGSPSEETSQKESSWIAHMMEAQQQGKCLSVSLDYQ
MEEPKEEFRVTTHWDSNQGGLPNGQVFSDFGQLQQSSGPKVSVEGGGDSYSFGGRRSSGGRKAGEKRRTKS
EKTISLQVLRQYFAGSLKDAAKSIGGKKISTTISNIILFIKSIVCPTTLKRICRQHGITRWPSRKIKKVGHSLRKLQLVID
SVQGAEGAIQIGSFYSSFPELSSPKFPGSDPFSSMKSSDQSKQLNSQTESGLFSQGGTMKSPSSSCSQTSGPSVC
AAGVQQHITNINGMSSADALMAEDPVGMLKRACSDAELLACNLEETKIMPRSLSYKALSENRTLESLPPLPGSS
GWNFRDGGALRVKANFGDEKIRFSLQSNCSFGDLQLEIARRFNLDDISNIDIKYLDDDREWVLLTCNADLEECM
EIYKSSQSRTLRLSLQRAPYPNLGSSFGSSSPS
>Discaria_trinervis_Distr2169S18482

MADQAGLSVPYHDPYDGPYNDDILNLMDDNIPFLADLAPLFDGPSFHPHSVRLFNDFGVQEAEHPSQWDSR
NNGPGGGVNSEAGPSGIGCEMGNPPGANLETPNACDDYACTKPLSVWPVPPLPFNCSCCQVLREIIHINGDKI
MKLEIHGRLGMICHAVFQNQIVDVSSSGNNNRSEMFDFCKKSIEDVKQFLMQYCLQRKLEGYVMQQDPLAFF
YEALCVGLEWDDIDITNSDDLILTSPVNSGSARMQQPEEAGQPAVDIEAERNRSRSLAEQRQRTGRLTLFDLREY
LHLPIEEAARRMNVCPTVVKKICRRNGLTRWPHRKIKSIRKQISNLLPSLDSDDARIRINAQTEINRLQQEVTNIC
AGVSIAML
>Discaria_trinervis_Distr2562S20066
MVAKDIENPFPFPTTNHFTTPFDLSSILGGDGFFPMDCQFELPLPDNNNNNILLDAVPLMECFPTDPLYTTLDFK
PTGPNLFQDDLFYGDHHGNSLGVYKDVFVGLESSQQQQQQLLTCNKDGLNNKKVLREERKIIKRVRSSSEEKS
SSCSKALSRQIISQYFYMPITQAAKELNVGLTLLKKRCRELGIRRWPHRKLMSLQTLIKNVQELGKEEGEESELKLR
DALELLEREKRLMEELPDMQLEDNTKRLRQACFKANYKKRRLMGMMGNSQAPSLICNNSPAANSMSITYEN
RNDDEDDSQEELISLLSDSFSSTDVSQRQTRSKPEYPFAPNPRKLLISLIKSSTHKTHLLPIHAHIVRTSLIQDHSVSL
QFLSRAALSPVRDVVYSRLFFSQIWEPNVLQCNTMIRAYSMSDSPEEGVYMYREIRRRGLCPDPVSSSFALRSCI
RVSSLEGGIQVHGRVLRDGHQLDSLLMTTLMDLYSCCERFREARKLFDEIPTKDAVAWNVLISCFLRNKRTRDA
MSLFDIMQSETYACEPDAVTCLLMLQACANLNALEFGERVHEYIEEHGYSGALNLSNTLISMYSRCGCLDKAYG
VFKGMQEKNVISWSAMISGFAINGYGREAIDAFEEMRRVGVQPDAQTFTGVLSACSHCGLVDEGMMFFDCM
SKDFRIEPNIHHYGCVVDLLGRAGKLDRAYQLILSMNFRPDSTIWRTLLGACKIHGNVNLGECVVGHLIELKAAE
AGDYVLLLNIYSSVGNWEKVKEVRKFMKDKAIQTTPGCSTIELKGVVHEFVSDDVSHQRKDEIYKMLDEINSQL
KIAGYEVEISSELHNLGSEEKGYVLSYHSEKLAIAFGILATPPGTTIRVAKNLRICIDCHNFAKVLSWSYNREVIIRDR
TRFHHFQYGKCSCNDYW
>Dryas_drummondii_Drydr2S08004
MASLSYGQHTPLCQMDYSTVSTQLIVPKIEYPQYDFDWIDYYQDPFHNTSTLLEQVPQQDNFFDHDQFSSQPL
PYNQYNQLLPHMGYQDFEDLSSWDYTLDNTATFASSSHCDYMVVDEKPLNTLVNSFEQYYSNNMSTSDFDEV
SRMIEWGLNNWGKHEEESGRSTRSSGNKKIKKTAALELDEIQKYFDLPISHAAKELKVGLTVLKKRCRELNIMR
WPHRKIKSLKTLIENVKGMGMTNEIMMLEEHKRLLQQLPDMELTERTKKLRQACFKANYKKKRSLTITAGRA
>Dryas_drummondii_Drydr394S11526
MPEPEDETRTPAIPPKSKNVAVVDEREAAVLMDFDLDLDASWPLDQIHFVSNPTSPLTFSSTDQPCSPLWAFFD
GDIDDKLAGHVTQALSDSPQFVACISDPVTEKPRENEYNRMLPSPFLGLEPVENPDVYCLFKERITQALRYLKEST
DQHVLAQVWAPVKKGSRYVLTTSEQPFVLDPHSNGLHQYRMASVMYMFSVDGESDGALGLPGRVFQQKLPE
WTPNVQYYSIKEYPRLDHAQHYNVQGTLALPVFEPSGQSCVGVLELIMTSPKINYGPEVDKVCKALEAVSLKSTE
ILEHTNKQIQICNEGRQKALTEILEILTVVCETHKLPLAQTWVPCMHRNVLAHGGGLKKSCTSFDGSCMEQVCM
STTDVACYIVDAHMWGFREACVEHHLQKGQGVTGRAFLSRNACFCRDITQFCKTEYPLVHYARMFGLTSCFAIC
LQSIHTGNDDYILEFFLPPSADSYVQQTLLGSLLAIIKNHFQTLKVASGFVLEEEGIVEVVEVSTNEGLDSRLECIWI
PHSTETPPEPELPNREETVHLDSSNPQLMVDFDAVNNGNNAVNECEKNNSSLSENKDIKRASERKRGKAEKSIS
LEVLQKYFAGSLKDAAKSLQVCPTTMKRICRQHGISRWPSRKINKVNRSLSKLQRVIESVQGAEGAFGLSPLTAS
PLPVAAGPISQPSILNGSNKQSSPRSKPCELHGEKKDSPTATTPGSLGQAGIEDQFIDGGILSQEELLQEHSRFLLD
IGKGSNMSKTGSGSREASDGTPPTSNSSCQGSPANGGSMVNGPVVSSIHEQGIEVDGSPESAFRPPVELNLAV
ACSIPDALLMTEAEEPCRGMLIEDSGSSKDLRNLCPSAADAVVDEQVREARWTNHPCSDSAPKQSIGTLPHTM
PHIVDRKEMKSVTIKATYKEDIIRFRLSMSSGIVELKEGVEKRLKLEVGTFDIKYMDDDLEWVLIACDADLQECM
EICISSGSNMFRVSVHDIMPNLGSSYESTDE
>Dryas_drummondii_Drydr83S05805
MEDGLLSSATMLGAQPDFAMDLDFMDEIFLEGCWLETTDGPEFPYQISSGSDALLDHSIFWPALETNGNLRM
NPSQNSNQQEMQRSLFTDLQEEGPINVQSPGQNMIDAKGCSGQLADPTIEGHELSGSWWIGPTENPALSVM
ERLMRALVYIGDVMRNKDVLVQIWVPVDRGGRRVLTTNDLPFSLDSGCPRLAKYRGISVNYQFSTGEDSEELVK

GLPGRVFSGKVPEWTPDVRFFRSDEYPRVDHAQLYDVRGTLALPIFERGSRNCLGVLEVVTTTQKIKYQTELESV
CKALEAVDLQSSKNLSTHNVKECDKPYQAALPEIQEVLRSACETHKLPLAQTWALCTQQGKDGCRHSDDNYVH
CVSTVDHACHVTDRHIQGFHEACSEHHLLKGQGIVGSAFITNQPCFSNDITSSVKTEYPLSHHARMFGLHAAVA
IRLRSILTGSTDFVLEFFLPVECRDPEEQKKMLTSLSLIIQQTCRSLRVVTDKELEEETDFPVSEAVAPSDPSPSSFAC
LTEVQQSGNDVSIYPKEKQREILGVKSSKLKQRQQDSNQKVGTEFGGECSTFGEGSFSSVGVSKTREKRRTKAEK
AITLQVLRKYFSGSLKDAAKSIGVCSTTLKRICRQHGIQRWPSRKIKKVGHSLQKLQLVIDSVQGASGAFQIDSFYT
NFQELASPNLSGTNPFSTSKLSDQPKPTNMPPEGGVFISQATASKSPSSSCSQSSSSSHCCSSRTQQQPPTWNV
AGNADPIVGSNFSDGMLKRVRSEAELHAFGQDGSELLPRSQSGKNLREQKKVKSIPPAVKNNGRLIQEGDAQR
IKVTYVDEKTRFRIQSDWGYEDLVQEIARRFSIEDMSKFDIKYLDDDSEWVLLTCDADLEECIDVSQSSQGHTIKL
SLQLSRRHLKMLLGSSGHS
>Dryas_drummondii_Drydr109S15134
MEYNFSSKEKGSDRWVSSRAQVENLASFEVGTRNSISEDMFNNISELMNFDTFAGWCSSPAAMDQISASLGV
SSCPSATYAPLDALNFAEQNSGALPVTEGGGTFNVAGSPFNCGNKIVFQPMDTPQCGVSIDSHDANDSAAKPN
NRSFQQNNVMDMSNYMISRPPGWSLSEKMLKALSLFKDSSGGGILAQLWVPMKHGDQYLLSTCEQPYLLDH
ILTGYREISRMFTFSAEEKQGSILGLPGRVFVSKVPEWTSNVSYYNKAEYLRVEHAANHQVRGSIALPVFDLDPE
MSCCAVLELVSTKEKPNFDTEMEIVCDALQAVNLRTSVPLRLLPQCLSMNQRAALTEITDVLRAVCHAHILPLAL
TWIPCYYHEGTGDEIIRVRVKEGVTSSDEKCILCIEETACYVNDRTMQGFVRACAEHHLEVGDGIAGKALQSNH
PFFINDIKVYDIYEYPLVHHARKFGLNAAVAIRLRSTYTGDDDYILEFFLPVNMKGSPEQQLLLNNLSSTMQRICKS
LRTVLDAELVGVEDSNAGFQRGAIPNKPPISRRNSQTTSTDGELKSMEKIPSNVFNRRNGGNEADNPPEQAPS
GSKRQVEKKRTTAEKNVSLSVLQQYFSGSLKDAAKSIGVCPTTLKRICRQHGISRWPSRKINKVNRSLKKIQTVLD
SVQGVEGGLKYDPTTGGFVATGSIIQEFDAPKNILFPEKNMPLQNAEPVSQDPVSVPSMSCNNDENLVIKLEEN
GCVGGSMLIPTSHESEVKKHNTPLIKCSMDSKPTSIDLMSCRPTNHDTMPPSCPENASRRCLKLENSDCHFVSQ
SSSSLAATDEMDTGVDGDDGIVEYNQHTSSSMTDSSIGSGSSMHGCSSSSQSFEERKHPQVNTSSVENESKIIV
KATYKEDTIRFKFQPSVGCLQLYEEVAKRLNLQNGTFQLKYLDDEEEWVMLVSDADLLECLEILDDIGTRSVKFLV
RDIPFGVSSSGSSNCFFAGGL
>Dryas_drummondii_Drydr295S20004
MLSRETISKYFYMPITQAAKELNVGLTLLKKRCRELGIRRWPHRKLTSLQTLIKNIQELGKEGVESEEKLRSAIELLE
RERKVMEEVPDMQLEDNTKRLRQACFKANYKKRKVMTGMLECSSHSSNLMITNHASMAAASNSFDQTREE
EDEEIKSLLSDSFSSTSMNML
>Dryas_drummondii_Drydr146S16094
MADPCSVLPYHDPYDLGQYNEIMNLLYDDSDPILENVPPPLEGLSSPNHDYHPIHHQIEALDGGDHHHPMPLF
DGVQDFDHLLSDPNPTMGDLGNLSIDNNVGGSDSQAGPSQIFGELSNFDPPRGDEFDRNPRFADVRPLTVW
PEPPVPFNCSCCQVLREIIHFNSNDIMKLEIHGRLGMICHAILEIRRNIFNVNSSDDNQYQMFDFCKKSIEEVKQF
LMQYCLERKLEGYSMQQDPLAIFYEALCVGMDWDDILNTDDFIPASSPNPGAEGEMEQPANPPEAAENEIERT
RRFSLAEQRERTANMTLDDLREYFHLTIEEAAKRLLVCPTVVKRICRKYGLSRWPSRKIKSIERQKTNLRPSLCSND
AEIRARAQAEIQRLETEVASLVP
>Dryas_drummondii_Drydr45S12448
MDSSEPHCLRSLLVFKNTVNQELIRSLHVYEIEDGKENGVEREFVFSANGSYEEVTAEPLLRLMKFRASQVLEGH
VNGSWVCILAFHANRSPNFTCIPDILSVSRNPKLQSIPTLANDLQNIFKLSCITDDKEPSQRSQEETCEGDNDNH
HQLRRSLPVLDLDLNCLPYPVTMSESSEDHESGRNSPGTEKKKKRAASDHIANISLQDLAKHFDLPIVEASRNLN
VGLTVLKRKCREFGIPRWPHRKIKSLDGLIRDLQEETEIQQKENEAAALAVAKRQKMLESEKESIEKTPFLEMKNE
TKRFRQDVFKRRHRARALKSQDPSNSGD
>Dryas_drummondii_Drydr380S11191
MDYGGLMPNPSYGTLSDTTMEVDFMDELLFQGCWLESNPGLNLLQQQHQQPAGPPVSRALTDHPSHYFLPY

ESNPTAAHLSITPHQHQQIYQEDTDGSYPESHSSFVVEGNTEAGRRLWIGPRANPGPSSSVKERLMLAIGYLKE
CTKGRDVLIQIWVPIKRGGRQYLTTHDQPFSLDPNCKSLAGYRNVSKDYQFITDEDSAESVGLPGRAFLEKLPEW
TPDVRFFRSYEYPRIEYAQQYDVRGSLALPIFERGSGTCLGVVEIVMTTQKVNYRPELEYVCQALEAVDLRSSQN
FCPPCVKTCDELYQAALAEIVEVLTSVCKTHRLPLAQTWAPCVQQGKGGCRHSDENYAHCVSTVDVACFVADL
DILGFHDACSEHHLFRGQGIVGTAFTTNKPCFATDIKAFSKTEYPLSHHARMFGLHAAVAIPFRSVYTGPADFVLE
FFLPKDCQDHEEQKQMLNSLCIVIQQACRSLHVDVDKELEEEVMFPVRESVVASDMGLHKEETQKLISSPTEEP
SAKESSWIAHMIEAQQKGKGVSVSLDYQTEEPKEEFKVTTHWDNTQGSLHNGQVFSEFGQLHQSCGPNGNV
EGGADSYSFGGRRTSGGRKAGEKRRTKTEKTISLPVLRQYFAGSLKDAAKSIGVCPTTLKRICRQHGITRWPSRKI
KKVGHSLKKLQLVIDSVQGAEGAIHIGSFYSSFPELNSPKFPGSGQYSSLNMSDHSKQVNPQHENGLFSHGTTT
KSPSSSCSQTSGPSVCVPGAHQHPVTINALSSGDAFMTEDPVGVLKRACGDASLHASFQEETKLIHRSQSHKSF
SDNLTYENLPPLAGSSSRNSRDGGLYRVKATFRDEKIRFSMLPNSSFRDLQLEIARRFNLDDVSRIEIKYLDDDRE
WVLLQCDADLEECMEIYSSSPGRTVRLCLQQAFYPNLASSYGNSSPS
>Glycine_max_Glyma.10G234100.1.p
MSEPEEENNQDYVPRSKPAEEGGGGCTTMDFDLDLETSWPLDHMAFGSNPMSPFLFSTSSDQPYSPLWAFS
DGEDPKLPASAFSDCHKIFSCDSNSIAEKPVENDDNKKNLPPLVPMPPVENLDGYCVIKERMTQALRYFKELTEL
NVLAQVWAPVRNGNRYVLTTSGQPFVLDPHSNGLHQYRTVSLMYMFSVDGENDGSLGLPGRVFQQKLPEW
TPNVQYYSSKEYPRRDHAQHYNVRGTLALPVFEPSIQSCVGVLELIMTSPKINYAPEVDKICKALETVNLRSSEILD
HPYTQICNEGRQNALSEILEILTVVCETLNLPLAQTWIPCKHRSVLAQGGGVKKSCSSFDGSCMGKVCMSTTDI
AFYIIDAHLWGFREACVEHHLQQGQGVAGRAFLSHSMCFCSNITQFCKTDYPLVHYALMFGLTSCFTICLRSSHT
GNDDYVLEFFLPPRITDFHEQKTLLGSILAIMKQHFQSLKIASGVELEDGSIEIIEATIERVHTRHESIPITPSIKSPPR
LDTSPNMGEEVPQDPSEQQILMYCNDMNDGRSLGKNADGIDHMPSIETKNIKKPLERKRGKTEKSISLEVLQR
YFAGSLKDAAKSLGVCPTTMKRICRQHGISRWPSRKINKVNRSLSKLKRVIESVQGAEGAFGLNSLSKSPLPIAVG
SFPEPSTPNKFSQSASLSIKPSEPQVKENELNASKALEANRQAGMEDQLLGGRTQNLEKVINDKGGYTREVGRE
PKRTRTRNGSSEDSTNPTSHGSCHDSPPNESSPVKDIFITSNNDQCAGIKRSPESTLQPTINTPSRPTAYPMPDFV
AVELQEPFGGMLIEDAGSSKDLRNLCPLVAEVILEDMIPEACGTNLPGPDLSPKLSMGTPNKAVTPFAAMKEM
KTVTIKATYREDIIRFRVSLTCGIVELKEEIAKRLKLEVGTFDIKYLDDDHEWVLIACDADLQECMDVSRSSGSNIIR
VLVHDITSNLGSSCESSGE
>Glycine_max_Glyma.17G100400.1.p
MESQSLMGWSSFYEIDEKPFPFTCQFSYSGNSEVYPSLDWPYGFPIQDYYLDAVPLMDYYPSDPLYETLTLATTE
PTQSVQDYDFYDIKKGLSGWNEVDAVFDSQKVFLFGNNEESGGIEKQMMEEDGKVNNQGREERINNSSCRTL
SRKTISQYFYMPISQAATELNVGITLLKKRCREVGIRRWPHRKLMSLQTLINNVQELGKEEGGESEEKLRSAIEILE
REKKVLEEMPDTELEDTTKRLRQACFKANYKKRKLTVTGRRKEPRSSSSSSLH
>Glycine_max_Glyma.09G137000.1.p
MSESKDENPDLPPKSKPQEEHGFAMDFDISLESLWPSDHISLVSNPMSPFLFSTISDQPCSPVWAFSDAEDERLI
RIAASAGNTNTTTENLVENYDNKKTVPPLVAIPTSENPDAYCLIKERMTQALRHFKELTEQNVLAQVWAPMRN
GNRYALTTSGQPFVLDPHSNGLHQYRTVSLMYMFSVDGENDEIMGLPGRVFQQKIPEWTPNVQFYSSKEYQR
LNHAQHYNVRGTLALPVFELAGQSCVAVVELIMTSQKINYAPEVDKICKALEAVKLRSSEILEHQYIQICNEDRQY
ALAEILEILTVVCETHSLPLAQTWVPCKHRSVLAHGGGHKKSCSSFDGCCMGQVCMSITEVAFYVIDAHTWGF
HEACVEHHLQQGQGVAGRAFLSHNMCFCGNIAQFCKTEYPLVHYALMFGLTSCFAVCLQSSHTGNDDYVLEFF
LPPGITDFNEQKRLLGSILATMKPHFQSLKIASGIELEENASIEIIEARNERVNLRFESIPITQSSKSPPRHASPNVGE
GLPLEPSEQKIMAYFDGINDGGSLGDNAGGHIDQNTSLKIKTKKKPSERKRGKAEKSISLDVLQHYFTGSLKDAA
KSLGVCPTTMKRICRQHGISRWPSRKIKKVNRSLSKLKCVIESVHGAERAFGLNSLSTVSLPIAAGSFSEPSTSNKF
NRQTSLTIRPSEPKINEKDFDASGASETKRQAAMEDQFLGLEARAQSPEKVINDRGVATQEIGTKGTNKFRTGS
GSSESSGNPTPHGSCHGSPPNEISPPKDIFVTGHSEKCLVLRGSLGSTTLHSTSTPNCTTAYPMPHIVETTEPQELF

GGQLLERAGSSKDLRNLCPSADAVLEDQVPEAYKMNPQCSDLPQMQHMDNLNNTLTPFAVRKEVKSVTIKAT
YKEDIIRFKVSMDCGIVELKEEIAKRLKLEAGTFDIKYLDDDHEWVLIACDADLQECMDISRSSGSNVIRLVVHDIL
PILGSSCESSGDWKGCI
>Glycine_max_Glyma.04G054800.1.p
MDNNNNNNINSSVFVSYACRDPFVSPLLDNFNEPDPALVYLLPEPNLRNDEQQPLQGHVSSTDSTIHDQSHHY
CNDNFTIGGGVFPNYEFVEGSSKNKRKYPFTDTDIEDQSFMHDFEAGPSNLNQPASGNMGPRNWPPMPRPF
FCTCCQVLRQTIHTNGTDFERLEIHGRIGLISHIIIQNQSITPGGRSCDNYQMIDFTNGSPAEVKGFLEDYIARKDK
LGYIIAEESFSEFYEAVCTKLDWVGKRTNNEDDDLTPQNVEEEPEPEPEPPKERRRKRSRKDQSDRINSMTLDDL
SGVFHITMKAAKNVLDMSVTAIKSICRKCRLYKWPHRQLQPLARRLKVLKRALESSQDPVVIQTTSMEVRRIKQ
HMTQLCGGVTPTGIEIPEVEE
>Glycine_max_Glyma.04G234600.1.p
MGFVFVSDHDIVPHTFNSSFSSAQVNHTHKIMEISPLEGFSELDWPYSSNHLCFNEFPDLENLDLDFSLPPLGEY
LEEELDQKPLNIVVSQGVHCNREKGFGEASSFTIENGSATASYVQNIIRQRPQPVSSSSKKKTCVLKFDEIRKHFG
VPITEAAKRLNVGLTMLKRRCRELNVKRWPHRKLKSLQLLIDNVKEMGLEDEVARVEKHKRLLEKLPGMELSEE
AKKLRQACFKANYKKRRCMASLQA
>Glycine_max_Glyma.04G169000.1.p
MENQQLTFWKSNYEIDDYPFAPTCQFSSYDYCSWGNANDGYHWPYEFPLQDSYLDAIPYMKCYYPHDILYEST
LPIEPTSLSIRDYDFSDVKNVLSALNETDGSDHKPAFSSCNDGESVDEMQEDLKGKKCREEGISSSAKMLSRKTVS
QYFYMPISQAARELNVGLTHLKKRCRELGIQRWPHRKLLSLQTLIKNIQEQRETGGHENDEKTRAAIEILEREQR
KVEEMPDLELEDNTRRLRQACFKANYKKRKLMGMRLIEPDSSLSGLFNKY
>Glycine_max_Glyma.04G017400.1.p
MGDSGVTSSAATMMEAPPPDGTTTTATSMDFDYMGEFFLDGCWLEASADVSDFLLQSPSFSNPLFDPSLSW
PALETNHNKSQDAAFGTQQESHNNIVSVVAGGGYSQQFQSETHSVEGVSEGVRRWWFAPSPIPSPGPGPSIM
EKLIRALMWIKDYNRNKDMLIQIWVPIHKEGRPILAADDLLFSLESKSLNLAKYREISVTYEFSAEESDSKELARGL
PGRVFRYKVPEWTPDVRFFRSDEYPRVDHAQEYDVRGTVAVPIFEQGSKTCLGVIEVVMTTQQINYGPELESVC
KALEAVDLRSSKQLSIQNVKQACNRTYEAALPEIYEVLRSACEMHRLPLAQTWVPCVQQGKEGCRHSEDNYLLC
ISPVEHACYVGDPSIRSFHEACTEHHLLKGEGVAGGAFMTNQPCFSDDITSLSKKDYPLSHYARLFGLHAAVAIRL
RSIYNSTDDFVLEFFLPVDCNDSEEQRKMLTALSIIIQRVCRSLRVIRDKELEEANLSVDEVIALADSGFARNAIFSE
PQYKGMVASLDAEEKSSETMGRKFSDLRQQQESPILKGNLDCVKECSTSVEGNLSSLGTNKTGERRRAKAEKTI
TLQVLRQYFAGSLKDAAKNIGVCTTTLKRICRQHGIKRWPSRKIKKVGHSLQKLQLVINSVQGASGAFQIGSFYS
NFPDLASPNLSGTGFFSTLNQSDYPNSTSTQPDHGSLSPEGASKSPSSSCSQSSISSHSCSSMSELQQHRTANGA
GNKVSTTVSEDSAGVVLKRISSEAELKSLSQDRAKLLPRSQSQETLGEHPKTQYQQPLLKTSSSKVDSHRVKVAY
GDEKTRFRMPKSWGYEDLLQEIARRFNVSDMSKFDVKYLDDDCEWVLLTCDADLEECIDVCQSSESGTIKLSLQ
PSSHSMRSSLEFR
>Glycine_max_Glyma.04G000600.1.p
MEYGGLVQNSAYGSFSEIFGPETDFIDELFVEGCWVETRVGSYLNCGGEANSSKPNTMASMETSAQIIFQEESL
SDPEISLMVGKRWWIGPRENPGPSSSVKERLVIAVGYLKEYAKNSNLLIQVWVPERRRSARAQPQDNYPYAALL
NTTSAFQFQEDWVHDQWTPNIRFFRSHEYPRHLRRPGSLALPVFESGSAMCLGVVEILMPNNPDLHALQSVD
FTSSTCHQNFIPPAAVTAKGFDELYQSALNEIVEVLTCVCKAHNLPLALTWAPCIQQGKSGCGHSNDENYVSIVD
PASFVADVEVLGFLEACSECHLLGGQGVVGTAFTTTKPCFANDITAFSKAEYPLAHHANMFGLHAAVAIPLLSDS
ADFVLEFFLPKDCHDTQEQKQMLNSLSMLVQQACRSLHVVMGKEEEEEEEEEELIALPPVGKEMESSSSSSWIA
HMMEAQQKGKGVSVSLEYLQEPKQEFRVTTNCNEQVSVGVESGGGEESYTAAFGGRRGARKSAGDKRRTKA
EKTISLPVLRQYFAGSLKDAAKSIGVCPTTLKRICRQHGITRWPSRKIKKVGHSLRKLQLVIDSVQGAEGAIQIGSF
YTSFPELSSANGVSESSNINNKFFSEYNQGATTFKSPTSPCSQTTILISENQQPLVLGASIQVQEEAKHFAHHLEPL

PPAPLPQSSTSLWNTAGTFRVKATFGDEKIRFSLQPNWGFRDLQMEIARRFNLNEISNIQLKYLDDAREWVLLTC
DADLDECKDINTSSQSRTIRLFLFQASPLNHANAFRGTSPS
>Glycine_max_Glyma.15G027900.1.p
MEYPFSPKESVIGDWQSSGAQLEGSASLDGRMSNSIPEDMPNSFSELMNFDTYAGLCNSPSITDQILANDLPSF
ASLSYPLPDGFNLVQQYSGQYCMSGVGRNNNDMESSPIYGEKVVCQQMDTLLGCLNDTNEANNLNSKLKMN
SSSQHLNNFDTGNYMMSRSPGLSLDERMLRALSFFKESAGGGILAQVWVPIKHGDQFILSTSDQPYLLDQML
AGYREVSRTFTFSTEGKSGCFLGLPGRVFTSKVPEWTSNVGYYSMSEYLRFEHAINHKVRGSIAIPIFDLHSEFPC
CAVLELVTTKEKPDFDRELEIVRHALQLVNLRTVKTLRCLPQSLSNNKKATLTEIVDVLRSVCHAHRLPLALTWIPC
GYTECSRGEASRIRIKGGHSTSSEKSVLCLEESACYITDRAMAGFIRACMEHHLEEGKGIAGKALQSNHPFFYPDV
KTYDISEYPLVHHARKYNLNAAVAIRLRSTYTNDDDYILEFFLPVNMRGSSEQQLLLDNLSGTMQRICSSLRTVSE
TELSGIESSPVGLGKKNAPSFFPLSSRNSDIPLINGDCDSVQKMSLKATTNLKDNEIEPSPNQERNGSKRQVQKN
RSTSEKNVSLSVLQQYFSGSLKDAAKKIGVCPTTLKRICRQHGIPRWPSRKINKVNRSLKKIQTVLDSVQGMEGG
LKFDPSMGAFVAGGSIIQEIDAPKSTIKDPVPVTQDAFSVPPAPCSEGENFSIKLEGKLKKTNVSSVDYSEDSKSM
AINDGSCQMASLCTKVQDCPEQACLGSVLAKEHDKRILNKGGLSVEKFKHNIVGQSSKSLIADEMDIGVDGDD
GVVERNHPTSSSLTDSSNGSGSMMHSSSSGSRSFENQDQSKVKSTIIDCGSKLIVKATYREDTIRFKFDPSEGCFR
LYEEVAARFKLQNGLFQLKYLDDEEEWVMLVNDADLQECIEILDDIGTRSVRFLVRDMPSVLSSSGSSNSYLGGS
S
>Glycine_max_Glyma.13G346300.1.p
MEYPFSPKDRVIGDWQSSGAQLEGSASLDGRMINSISEDMPNSFSELMNFDTYAGLCYSPSITDQILANELPSF
APLSYPLPDGFNPVQLNSGQCCMSGVGRNNNDTENSSLYGEKVVCQQMDTLLGFLNDTNEANNLNSKLKINV
SSQHLNNSDTGNYMMSRPPGLSLDERMLRALSFFKESAGGGILAQVWVPIKDGDQFILSTSEQPYLLDQMLA
GYREVSRTFTFSAEGKSGCSLGLPGRVFTSKVPEWTSNVGYYSMSEYLRFEHAINHKVCGSIAFPIFDLHSELPCC
AVLELVTTNEKPDFDRELEIVCRALQLVNLRTAKPLRCLPQCLSNNKKATLTEIVDVLRSVCHAHRLPLGLTWIPCC
FTECSRGEASSIRIEGGHSTSRGKNILCLEESACYITDRAMGGFVRACMEHRLEEGKGIAGKALQSNHPFFYPDV
KTYDISEYPLVHHARKYNLNAAVAIRLRSTYTNDDDYILEFFLPVNMKGSSEQQLLLDNLSGTMQRICSSLRTVSE
TELSGMESLAVGLGKKNAPSFFPLSSRNSEIPLINGDCDSVQKATNLRDNEIESSPNQERNGSKRQVQKNRSTSE
KNVSLSVLQQYFSGSLKDAAKNIGVCPTTLKRICRQHGIPRWPSRKINKVNRSLKKIQTVLDSVQGVEGGLKVLD
SVQGVEGVLKFDPSMGAFVAGGSIIQEIDAHKSTIKDPVPVAQDAFSVRPAPCSEGENFSIKLEGKLKKTNVSSV
NYSEDSKSMAINDGSCQTASLCTKVQDCPEQACLGSVLAKEHDKRILNKGGLSVEKFKRNIVGQSSKTLIGDEM
DIGVDGDDGVVERNHPTSSSLIDSSNGSGSMMHSSSSGSQSFKNQDQSKVKSTIVDSRSKLIVKATYREDTIRFK
FDPCAGCFSLYEEVAARFKLQNGLFQLKYLDDEEEWVMLVNNADLQECIEILDDIGTRSVRFLVRDMPSVLGSS
GSSNSYLGGSS
>Glycine_max_Glyma.02G311000.1.p
MEYGAFGSLSECEEDLIEELLEKGCWVETNGSSEAQYDIEQELAMPMGKTTWWIGPKGPVSGSSSSVKERLVV
AVGYLREYTKNNNAANVLIQIWVPIRRMTGLLAIGLGPDESVSVAFPFPTNMNMNMKSNVNIRFFRSHEYPRI
PYEYGSLALPVFERGSGTCLGVLQIVMPAARWDTINYLPQLDNNNNNNAFQGMIPGGVVKVFDEDEVYEASV
NEMMEVVRCVCKTQNVPLALTWAPCVQQLCVDCGHSRDENNYVSIVDRACFVGDGQPQLLGFQEACSQHH
LFRGQGIVGTAFTTAKPCFAMDITAFTKAEYPLSHHANIFDLHSAVAIPLRTSSYPHFDFVLEFFLPKHCPDHNLFL
NSLSILLQQACPTFHLSAPIHDDVFTSLPSESQSQQASWIAHMIDAQSQHKDNGKGVCLSLDFLEEEPKEEFKVT
TTHCNWDTTATSTYHSHDHVVFSDFGEESQSQSQSQTHTFGVKRGRKPGEKRRTKAEKTISLPVLRQYFAGSLK
DAAKSIGVCPTTLKRICRQHGITRWPSRKIKKVGHSLKKLQLVIDSVQGAEGAIQIGSFYNSFPELSSRASQSSSPA
AQSPPHVPQQPLRVKATFADEKIRFSLQPLWGFTELQLEISRRFNLSDVSNANNVVLKYLDDDGEWVVLACDG
DLEECKDLHTTSQSRTIRLALFQASPLNNQPNSYTFANTTSTPSSS
>Glycine_max_Glyma.20G160200.1.p

MPESEEENQDYVPRSKPSEEAGGGCTMDFDLDLETSWPLDHMAFGSNPMSPFLFSTSSDQPYSPLWAFSDGE
DPKLPASAFSDCHKIFSCDSNSIAEKPVENDDNKKLLPPLVPISPVENLDGYCVIKERMTQALRYFKELTELNVLAQ
VWAPVKNGNRYVLTTSGQPFVLDPHSNGLYQYRTVSLMYMFSVDGENDGSLGLPGRVFQQKLPEWTPNVLY
YSSKEYPRRDHAQHYNVRGTLALPVFEPSIQSCVGVLELIMTSQKINYAPEVDKICKALETVNLRSSEILDHPHTQI
CNEGRQNALSEILEILTVVCETHNLPLAQTWIPCKHRSVLAQGGGVKKSCSSFDGRCMGKVCMSTTDIAFYIIDA
HLWGFREACVEHHLQQGQGVAGRAFLSHGMCFCSNITQFYKTDYPLVHYALMFGLTSCFAICLRSSHTGNDDY
VLEFFLPPRITHIHEQKTLLGSILAIMKQHFQSLHIASGVEPEDGSIEIIEATIERVHTRLESIPIASSIKSPPRPDTSPN
MGEEVPQDPSEQQILMYCNDINNGGSLGENAARNIDHMPSLETKNINKPLERKRGKTEKSISLEVLQRYFAGSL
KDAAKSLGVCPTTMKRICRQHGISRWPSRKINKVNRSLSKLKRVIESVQGAEGAFGLNSLSKSPLPIAVGSFPEPS
TPNKFSLPASLSINPSEPQIKENELNASKALETNSQAVMEEDRLLGGRTPHLEKVINDKGRHTREVGKEPKRTRT
GSGSSEDSTNPTSHGSCHDSPPNESSPVKNIFITSNNDQCAGLKRSPESTLQLTTNTPNRPAAYPMPDFVAAEL
QEPFGGMLIEDAGSSKDLRNLCPSVAETILEDMVPEACGTNIPGPDLSPKQSMGTPNKAVTPFVAMKEMKTVT
IKATYREDIIRFRVSLTCGIVELKEEVAKRLKLEVGTFEIKYLDDDHEWVLIACDADLQECMDVSRSSGSKIIRVLVH
DITSNLGSSCESSGE
>Glycine_max_Glyma.20G016400.1.p
MDNNNNNYSGFVPNNDTDDDCTFLQTLLELDPQHFYLPTEEFVSLSLQQQQQNDPFGVEKGNNVPFLQGNN
NNDFPIGEGVVPDVVDVEEEGPPRRTGKEPIPEGQGDRFFIHGHDIFFFGEGGVGNNVEPPQQNQTQPPNN
QDVRSLCSWPPPPEPFSCSCCEVLRQINYTNGVRFDKLEIHGAVGVINHAICLVEDKTSNRPPRDTYQIIDFCNK
NTEEIRNFLNVYCKERTEAGFIPIEDPSMSAFYDALCTGMDWTEVSNDVDDDLGPPNGTEQESESEPEAENGR
RASTSRPRRRDMAVQRQRVPRMTLSDLSGVFHLTISAAAVKLDVGDSTVKSICRRGKLKRWPQRKVQSLAKKV
RVLERALNSPDPGVKRRTLDEIRRLQQEIIENCGGITPTGIPMLQFDEE
>Glycine_max_Glyma.20G016500.1.p
MFPIDNPLLRFLFPRPKQQGNNVPFLQGNNNNDFPIGEGVVPDVVDVEEEGPPRRTGKEPIPEGQGDRFFIHG
HDIFFFGEGGVGNNVEPPQQNQTQPPNNQDVRSLCSWPPPPEPFSCSCCEVLRQINYTNGVRFDKLEIHGAV
GVINHAICLVEDKTSNRPPRDTYQIIDFCNKNTEEIRNFLNVYCKERTEAGFIPIEDPSMSAFYDALCTGMDWTE
RQRVPRMTLSDLSGVFHLTISAAAVKLDVGDSTVKSICRRGKLKRWPQRKVQSLAKKVRVLERALNSPDPGVKR
RTLDEIRRLQQEIIENCGGITPTGIPMLQFDEE
>Glycine_max_Glyma.05G026500.1.p
MGWSSFYEIDEEPFPFTCQFSYSGNSEVYPSLDWPYDFPIQDYYIDAVPLMDYYPSDPLYETLTTEPTQSVQDYD
FYDIKKGLPFWKEVDAVFDSQKVFLFGNNEESGTKKEMMEDGKVNDNRGIEERISGSSSSCRMLSRKTISQYFY
MPITQAARELNVGITLLKKRCREVGIRRWPHRKLMSLQTLINNVQELGKEEGRESEEKLRSAIEILEREKKVLEEM
PDTELEDTTKRLRQACFKANYKKRKLTVTGRGREPHSSSFSGFVARSIVNATGEYSNESEEERDIKYLLSQFLE
>Glycine_max_Glyma.11G125500.1.p
MMRLLGCWLTRTKPLVQSLIQRNSNIGQISTSIFLSNLIMEDHVSPEGKEVSCCTPPGAQLEEPTSVDGGMKKS
ASEDMFSNFSELMNFDTYAGWSNSPSMTDQSLANVFSSFSLAPYPVPDVLNLVEHGNGPFFMTEDSEIHND
MESAPSCGERIIFQQMDFQLGFLDEANDSNSLDSKQKPNGTSQEVNTTDMCNYIISSSPGRSLDDRMLRALSF
FMESADGGMLAQVWVPIKHGDEFILSTSEQPYLLDPKLAGYREVSRAFTFSAEGKTQSCPGLPARVFISHVPEW
TSNVGYYNKTEYLRLEHARNHEIRGSIALPISDVHSQVPCAVLELVTTKEKPNFDRELEIVSQALQLVNLRTTMPP
RLHPQCLSSNKRAALTEIIDVLRAVCHAHRLPLALTWIPCCYSEGIRNETDRIRIKEGHTSPNEKCVLCIEESACYVN
DGAVEGFVHACVEHHLEEGQGIAGKALQSNHPFFYTDVKTYDIGEYPLVHHARKYNLNAAVAIRLRSTYTNDD
DYILEFFLPVNMTGSSEQELLLDNLSSTMRRICKSLRTVSDAELTGIEGSQGGFPKEKVSGFFPMSRRNSQIAFISD
DHDSVLKMSLKASNMRNNGIEAVHSQTMNGSRKQVEKKRSTVENNVSLSVLQQYFSGSLKDAAKSIGVCPTTL
KRICRQHGISRWPSRKINKVNRSLKKIQTVLDSVQGVEGGLKFDPYTGGFIAGGSIMQETEAHKYLVFPEKSSVK
DPKPATQKKVSVAPAPASTIENSTIKLNDDEGVCLVGNKLVHSRSIPNSNSGEGELKKDNVSSDDSKSMTMNDG

SCHKACHWKKTKDCPEQTCSMSLVTDEVEVGVDRVEGADEHNHPTSSSTTNSSNGSGSMMHGSSSCSHEN
QKYSKVKSNCVDSGSKMIVKASYRGDTIRFKFDPSSGCFQLYKEVATRFKLQNGSFQLKYLDDEEEWVMLVNDS
DLQECTEILDDIGTRCVKFLVRDVPCVLSSHGSNSCFLSDSS
>Glycine_max_Glyma.11G085500.1.p
MDSSHTNTTLLTNLLVFNNTLQPELMRSVHVYRRGGGEKREVEREFVFSESGSYVEMQATPIPGLVKSRVSEVC
EGHRNGVWLCVFAFHADHTPQFCRIPRLLLVTRNPKLKMIPNLLDDLHMIYKLNRKEEDRDIAQDSTGEERQG
HSNNFQPSRKVFPVLDQDLNFLPYEDYESESPDNETDVESSPGFLVKKKRAPSDLVAKISLSDLVKYFGMPIVEAS
RNLNVGLTVLKRKCREFGIPRWPHRKIKSLDSLIHDLQEEAKNQELEDREAAMAVAKRQRMLESEKENIEKKPF
MDIKIETKRFRQDVFKRRHRARAVEKHNSTVSST
>Glycine_max_Glyma.14G001600.1.p
MEYGGFGSLSECEEELIEELLVQGCWVETSGRSEEMPMGKTWWIGPKANHASVKERLEAAMGYLREYTNNNI
QIWVPLRRSAGQELGTDESDTIAFERNRNVKLRLFRSQEGCVGVPVLERGSGTCLGVLEIVMEDEVVSEMMEV
VRCVCKAQKVPLALAWAPCVQQKQAKTSSGRSLATAKPCFATDITAFSNAEYPLSHHASIFDLHAAVAIPLTTFSS
SFHFVLEFFLPLDCPDHNHFLNSLSLLLHQACRSTFHLSLIHDHHLDFEFLPTESPSQASWIAHMMEAQSQHIKG
VCLSLEEEPKEEFKVTTTHYCNWDSTATSTYQAHDQVVFGEESHTHTFGGKRGRKPGEKRRTKAEKTISLPVLRQ
YFAGSLKDAAKSIGVCPTTLKRICRQHGITRWPSRKIKKVGHSLKKLQLVIDSVQGAEGAIQIGSFYNSFPELSSQS
SKSNNNNNSSSPTAAAAAAQSPQQPLRVKATFADEKIRFSLQPHWGFTELQLEIARRFNLNDVSNGYLVLKYLD
DDGEWVVLACDGDLEECKDLHTTSQSRTIRLALFQASPLNNLPNTYTFAAATPSSSLAVSL
>Glycine_max_Glyma.01G159200.1.p
MESTLLTNLLVFNNTLQPELMRSVHVYRRGDGEKREVEREFVFSESGSYGEMQATPIPGLVKSRVSEVCEGHRN
GVWLCVFAFHADHTPQFCRIPPVLLVTRNPKLKMIPNLLDDLHVIYKLDRKEEDSDIAQDYTGEERQGNSNNC
QPSWKVFPVLDQDLNCLPYEDDESESLDNETDVESSPGLLAKKKRAPSDLVAKISLSDLVKYFGMPIVEASRNLN
VGLTVLKRKCREFGIPRWPHRKIKSLDSLIHDLQEEVKSQELEDREAALAVAKRQRMLESEKENIEKKPFMDIQSE
TKRFRQDVFKRRHRARAVGKHNSTVSST
>Glycine_max_Glyma.12G050100.1.p
MEDHVSPEGKEVSCCAPPGAQLEESTSVDGGKKNSSSEDMFSNFSELMNFDTYAGWSNSPSMTDLSVANVFS
LFSSAPYPPPDGLNLVEQSNGSFFMTEDSEFHNGMESSPSCVERVIFQQMDIHLGFLDEANDSNNLDSKEKLN
GKSQQVNTSDMCNYIISRSPGRSLDDRMLRALSFFMESADEGMLAQVWVPTKHGDEFILSTSQQPYLLDPKL
AGYREVSRAFTFSAEGKTRTCPGLPARVFISHVPEWTSNVGYYNKTEYLRLEHAKNHEIRGSIALPISDVHSQVPC
AVLELVTTKEKANFDRELEIFSHALQLVNLRTSMPPRLLPQCLSSNKRVALTEIIDVLRAMCHAHRLPLALTWIPCC
YSEGIRDETERIRIKEGHTSSNEKCVLCIEESACYVNDGGVGGFVHACIEHHLEEGQGIAGKALQSNHPFFYTDVK
TYDIGEYPLVHHARKYNLNAAVAIRLRSTYTNGDDYILEFFLPVNMTGSSEQQLLLDNLSGTMRRICKSLRTVSD
AELTGIDGSQGGFPKEKVSGFFPMSRRNSEIAFINGDHDSVQKMSMKTSNMRNNGTEAVHSQAMNGSRKQ
VEKKRSTVEKNVSLSVLQQYFSGSLKDAAKSIGVCPTTLKRICRQHGISRWPSRKINKVNRSLKKIQTVLDSVQGV
EGGLKFDPYTGGFIAGGSIMQEIDAHKYLVFPKKSSVKDPKPATQKTVSVAPAPGSTRENSTIKLNDDESVCLVRN
KFVHSRNVLNSNSSKGELKKDNVSSDDCCDDSKSMAMNDGSCQKSCLWTKTQDCPEQTCSISLVTDEVEVGV
DRVEGADEHNHPTSSSTTHSSNGSGSMMHGSSSCSQSFEKQKHSKVKSNCVDSGSKMIVKASYRGDTIRFKF
DPSSGCFQLYEEVATRFKLQNGSFQLKYLDDEEEWVMLVNDSDLQECTEILGDIGTRFVKFLVRDVPCVFRSRGS
NSCFLSDSS
>Glycine_max_Glyma.06G194300.1.p
MENQQLTFWKSNYDIDDYYPFAPTSQFSSYDYCGWGSAADGYHWPHEFPLQDSYLDAIPFMKFYYPHDILYET
TLPIEPTSLSIQDYDFSDWNEIDGSDHKPVFSSCNDGESVSEMKEDLNAKKCREEKISSSAKMLSRTTVSQYFYM
PISKAARELNVGLTHLKKRCRELGIQRWPHRKLMSLQTLIKNIQEQGEAEGHENDEKLRAAIEMLKREKRKVEE
MPDLELEDNTRRLRQACFKANYKKRKLVGMRFIEPESSLSGLSNKY

>Glycine_max_Glyma.06G130000.1.p
MGFVFVSDRDFPNLDNVPHTFTSSFSFAQLNHTHKIMEVSPLEGFSELDWWTPQLPYSSNHLCFNEFPDLENL
DLDFSLPSLGEHLEEVDQKPSNIVVPEGLRCNHEKVFGDALLATEHVSATCVKKEQVLNNIDQSPLPVLLASNSTT
RKKPCALEFEEASFAIQNQNGSATASYLKKEEVQNIIHQRPLPVSSSSKKRTCVLEFDEIKKHFGVPITEAAKRLNV
GLTMLKRRCRELSITRWPHRKLKSLQLLIDNVKEMGLEDEVPRLERQKRLLEKLPGLELSEEAKKLRQACFKANY
KKRRCMALQS
>Glycine_max_Glyma.06G198300.1.p
MDNNNNNSGFVPNNDTDDDCTFLQTLLEMDPQHFYLPTEEFDSLSQQQQQKNDPFGVEKGNKVSFLQGNN
NNDFPIGEGVVPDVVDVEEEEGPPRGAGKELIPEGQGDRFFIHGDDIFFFGEGGVGNNVDPPQENQTQPPNN
QDVRSLCPWPPPPEPFSCSCCEVLRQINYTNGVRFDKLEIHGTVGVINHAICHVEDKTSNRPPRDTYQIIDFCNK
NTEEIRNFLNVYCKERIEAGFIPIEDPSMSAFYDALCTGMDWTEVSNDVDDDLGPPNGTEQESESEPEAGNGR
RASTSRPRRRDMAVQRQRVPRMTLSDLSGVFHLTISAAAVKLDVSNSTVKSICRRGKLKRWPQRKVQSLAKKV
RVLVRALNSPDPGVKRRTQDEIRRLQKEIIENCGGITPTGIPMLQFDEE
>Glycine_max_Glyma.06G017800.1.p
MMGDGGVISSAATMMEAPPPDGTTTTSMDFDYMGELFLDGCWMEASADGSDFLLQSPSFSNTLFDPSFSW
PALETNHNESQVAAFGSQQESHNNNMVSVVAGGDYSQQFQSETHSVEGASEGIRRWWFAPTHTPTPSPGP
GPSIMEKLIRALMWIKDYNRNKDMLIQIWVPVHKEGRPILAADDLLFSLESKSLNLAKYREISVTYKFSAEESDSK
ELAWGLPGRVFRDKVPEWTPDVRFFKIDEYPRVDHAQEYDVRGTLAVPIFEQGSKTCLGVIEVVMTTQQINYG
PELESVCKALEAVDLRSSKQLSIQNVKQACNRSYEAALPEIHEVLRSACEMHKLPLAQTWVPCVQQGKEGCRHS
EDNYLLCISPVEHACYVGDPSIRSFHEACTEHHLLKGEGVAGGAFMTNQPCFSDDITSLSKKDYPMSHHARLFG
LRAAVAIRLRSIYNSTDDFVLEFFLPVDCNDIEEQRKMLTSLSNIIQRVCRSLRVIREKELEEANLSVNEVIALADSG
FTRDEICSEPQQKGMVASLDTEEKSSETMGRKFSEPRQQQESPILKGNLDCVRECSTSVEGNLSSPGTSKTGERR
RVKAEKTITLQVLRQYFAGSLKDAAKNIGVCTTTLKRICRQHGIKRWPSRKIKKVGHSLQKLQLVIDSVQGASGAF
QINSFYSNFPDLASPNLSGTGFFSTLNQSDNPNSTSTQPEHGSLSPEGASKSPSSSSSQSSISSHSCSSMSELQQQ
HTTNIASDKDPATVGEYSADVVLKLIRNEAKLKSLSQDRAKLLPRSLSQETLGEHPKTQYQLPLLKTSSSKVDSHR
VKVTYGDEKTRFRMLKNWVYEDLLQEIGRKFNVSDMSKFDVKYLDDDCEWILLTCDADLEECIDVCQSSESGTI
KLSLQPSSHSVRSSLEFR
>Glycine_max_Glyma.06G000400.1.p
MEYGGLVQNAYGSYGSFSDILGPETDFIDELFVEGCWLETRVGSYLNCGTEANSSKSNTMRSMESTQIIFQQEES
LPDPEISLMVGKRWWIGPRENPGPSSSVKERLVIAVGYLKEYAKNSNLPIQVWVPERRSARAQPQDNIYPYAAA
LLNGDAAAFQIQEDWVHVNDQYWTPNVRFFRSHEYPRHLRTPGSLALPVFERGTAMCLGVVEILMPNNPPD
LQCTVDFTSSTSSHHHNFIPPAVTVKGFDELYQSALNGIVQVLTCVCKAHNLPLALTWAPCIQQAGKSGCGHSN
DEMNYVSTVYAASFVADVELMGFLEACSECHLLGGQGVVGTAFTTTKPCFANDITSFSKAEYPLAHHANMFGL
HAALGIPLRSASADFVLEFFLPKDCHDTQDQKQLLNSLFMLVQQACRSLHLVMEEEEELIALPSVVGKEMESSS
WIAHMMEAQQKGKGVSVSLEYLQEPKQEFKVTTNCSNTNEQVSVGVESTAAFCGRRVGRKSAGDKRRTKAEK
TISLPVLRQYFAGSLKDAAKSIGVCPTTLKRICRQHGITRWPSRKIKKVKELLHSNPQQLRLAVRPLL
>Glycine_max_Glyma.06G054900.1.p
MTLNDLSGVFHITMKAAKDVLEMSVTVIKAICRKYRLYKWPQRQLQPLARRLKVLKRALESSQDPVIIQTTNME
VRRIKQEMTQLCGGVTPTGIEIPEVEENSV
>Glycine_max_Glyma.06G129900.1.p
MDFVFVSDNDFPNLDIIPHTLNNSCFCSPHLNDTHKIMEISPLKGYFSHLDWTPQSPYPSNELPDLESWNLDFIL
PPLDEDLEELDQKQLKSVVPQGFHCNQEKGFGDALLATENVSATCVKKEKVQIIDQSPLASSSSTKKRPCALEFEE
ISKYFGVPINEAAKQMNVGVTMLKKRCRELNIMRWPRRKIKSLQMVIDSVKEMGLEDEVARLEEHKKLLEKLP
GLELSEEAKKLRHACFKANYKKKRCMALQA

>Glycine_max_Glyma.16G182400.1.p
MSESKEENPDLLLKSKAQEEHGFVMDFGIGLESLWPLDHISLVSNPMPPFLFSTISEQPFSPVWAFSDAEDERQI
RIAASGNTNTTTENPIENDDNKKTVPPLVAMTASENPDGYCLIKERMTQALRHFKELTEQNVLAQVWAPVRNG
NRYALTTSGQPFVLDPHSNGLHQYRTASLMYMFSVDGENDEILGLPGRVFQHKIPEWTPNVQFYSSKEYQRLN
HAQHYNVRGTLALPVFELAGQSCVAVVELIMTSQKINYAPEVDKICKALEAVKLRSSEILEHPYNQICNEYRQYAL
AEILEILTAVCETHSLPLAQTWVPCKHRSVLAHGGGLKKICSSFNGCCMGQVCMSITEVAFYVIDAHKWGFHEA
CAEHHLQQGQGVAGRAFLSHNMCFCGNNTQFCKTEYPLVHYALMFHLNSCFAVCLQSSHTGNDDYVLEFFLP
PGITDFNEQKRLLGSILATMKPHFQSLKIASGVELEGNASIEIIDARNERVHLRFESIPITQSSKSPPRHTSPNMGE
GLPLEPSEPKIMAYFDGINDGGSLGDNAGGHIDQNTSLEIKTKKKPSDRKRGKAEISISLNVLQHYFTGSLKDAAK
SLGVCPTTMKRICRQHGISRWPSRKIKKVNHSLSKLKCVIESVHGAEGAFGLNSLSTGSLPIAAGSFSDPFTSNKF
HRQTTLTIRPSEPKINENDFDAFGASETKRQVAMEDQFLGLEARTRSPEKVINDKGVSIQEIGTKGTNKFRTGSG
SSGSGNPTHASCHGSPPNEISPPKDTFVTGNSEQCLVLRGSLGSTTLHSTSTPNRATAYPMLHIVETTEPQEPFG
GQLLEVAGSSKDLRNLCPSADAVLEDQVPEAYKMNPQCSDLPQMQHMDTLNKTMTPFAVRKEVNSVTIKATY
KEDIIRFKVSMDCGIVELKEEIAKRLKLEVGTFHNKYLDDDHEWVLIACDADLQECMDISRSSGGNIIRLVVHDIL
PILGSSCESSRDWKGCI
>Lotus_japonicus_Lj0g3v0169309.1
MIMGVHPFTSWSSNSDMSDENLYPLTCQFSHSGNGSASYPALDWSDEFPIYDSHFDAAPYNVECYPVDPLYET
LSIEPTPLTVQDYDFYEVKEGFSVWNEIDAGFESEKALLYCTNEESGKELMGNVKAKRGREVRSSSTRMLSRTTL
SLYFYMPITEAARELNVGLTLLKKRCRELGIRRWPHRKLMSLQTLIKNVQELGKEGNEGEEKLRNAIEILEKEKKLV
EEMPDKDLESNTKRLRQACFKANYKKRRLMEMEKMDPKYSSFYSGPVSVSITSSYATSECCYDDEKEHDIKGDL
RSKLPF
>Lotus_japonicus_Lj2g3v0381780.2
MPESEEENPDLPLKSKPMEEHGFPMDFDNDLESSWPFDHVSCLSNPMSPFLLSNISEQPFSPLWAFSDVEDDK
HVSITASDTVTENPVENDDSKKHMTPLVPMPPLENRDGFCLIKERMTQALRHFKELTEQNVLAQVWVPVRNG
NKYKLTTSGQPFVLDPHSNGLHQYRTVSLMYMFSVDGENDEILGLPGRVFQQKLPEWTPNVQYYSSKEYSRLN
HAQHYNVRGTLALPVFEPSGQSCVAVMELIMTAQKVNYAPEVDKVCKALEAVNLRSSEILEHPYSQNCNEGRK
NALAEILEILTLVCETHNLPLAQTWVPCRYRSVLAHGGGLKKSCSSFDGYCMGQVCMSITEVAYYIIDSHTWGFH
EACVEHHLQQGQGVAGRAFLSQNMCFCGNITQFCKTDYPLVHYALMFGLTSSFAICLQSSHTGNDDYVLEFFLP
PRITEFNEQKALLGSILATMKQHFQSLKIASGVELEEDGSVEIVETINERVHMRLESVPIIQSDKSPPRHDGSASPN
LRHGVPLDPLEQQTMLDGINDGGSLGDSGGGSMEKISSLEIKNKTSQRKRGKAEKSISLEVLQRYFSGSLKDAAK
SLGVCPTTMKRICRLNGISRWPSRKIKKVNSSLSKLKCVMESVHGAEGAFGLNSPSSSLPIASAEPCTSKKLDHQ
APLRIRPSESRMNWK
>Lotus_japonicus_Lj2g3v1623180.1
MDSSFLTNLLLFNNIIQPELMRSVHVYRQCGDDGEEETEVEREFLFSKYSYYVEMQASPILQLQKSCVSGIREGY
RNGVWDCIFAFHAGHSPQFSRIPPLLLVTRNPKLHCIPNLLNDLHIIYKLDVKEEDIDVTQEESLGNNNDCLPSEI
ALPKRDLDLNCLPYEEDESELQDNEIEVESLTGLVGKKKRAPSNHIANIAYSDLVKYFDMPIVEASRRLNVGLTVLK
KKCREFGIPRWPHRKIKSLDSLIHDLQEMAKHQELEDKAAAMAVMKRQKMLESEKENIEKQPFIEIQSETKKFR
QDIFKRRYRARAIEKQNSTVSSS
>Lotus_japonicus_Lj3g3v0339060.1
MEYPFSPKGGELGYWQSPSAQFGGSISSDNGISNSISDDMPNSFSELLNFDSYAGWFTGPSVTDQIFANELPSF
ASVSYPLSDGFNLAEQNSGQFFTTGVCGNSSNLESSSTHGERVVCQQMDTLLGFLDETNDANTLNSKQKINDT
LQQFSTFEEGNYTISRPPGPSLDERMLRALSFFKESAGGGILAQVWVPLEHGGQVILSTSEQPYLLDQMLAGYR
EVSRTFKFPAEGKPGGFSGLPGRVFVSKVPEWTSNVGYYSKNEYLRVEHARNYKVRGTIAFPIFDTHSELPCCAV
LELVTTKEMSDFDRELEVVCHALQLVNLRTTMPLRIFPECYSNNKRAALAEIVDVLKSVCHAHRLPLALTWIPCCY

TEGPKGEAMRIQIKEGHSSSGEKVLLCIEESACYVTDRLMEGFVRACIEHPLEEGKGIAGKALQSNHPFFYPDVK
EYDISEYPLVHHARKCNLSAAVAIRLRSTHTNDDDYILEFFLPVNMRGSSEQQLLLDNLSGTMQRICRSLRTVSD
VESSRIESTHMGFENKNLPSFSPLSRENSQIPLINANQDSVQKSLKASRLRNKGSKPPYNQVSNGSRRQVEKNR
GTAEKNVSLSVLQQHFSGSLKDAAKSIGVCPTTLKRICRHHGISRWPSRKINKVNSSLKKIQTVLDSVQGVEGSLK
FDPSVGAFVAGGSTIQGIDTHKSLLFPEKSTIRDPRPITQDAVSVPAVPCGESEKSAIKLEGKLKKTNASLVDCCED
SKSMAMDDRSSHSSSLWTKAQGSSEQASLGSDLAKRDKWVLNNDGLRVENLKCNTVGQSSGSFTDDEMGID
VDNDEVVELNHPTCSSLTGSSNGSSPMIHGSSSSSQSFENQSKGKSTTVDRGSKIIVKATYRKDIIRFKFDPSAGC
FKLYEEVAARFKLQNGTFQLKYLDDEEEWVMLVSDSDLQECVDILDDIGTRSVRFLVRDLPCVSEAVSTEAAHSE
L
>Lotus_japonicus_Lj4g3v1635310.1
MIMGVHPFTSWSSNSDMSDENLYPLTCQFSHSGNGSASYPALDWSDEFPIYDSHFDAAPYNVECYPVDPLYET
LSIEPTPLTVQDYDFYEVKEGFSVWNEIDAGFESEKALLYCTNEESGKELMGNVKAKRGREVRSSSTRMLSRTTL
SLYFYMPITEAARELNVGLTLLKKRCRELGIRRWPHRKLMSLQTLIKNVQELGKEGNEGEEKLRNAIEILEKEKKLV
EEMPDKDLESNTKRLRQACFKANYKKRRLMEMEKMDPKYSSFYSGPVSVSITSSYATSECCYDDEKEHDIKGDL
RSKLPF
>Lotus_japonicus_Lj5g3v1999250.2
MSESDEEKPDFPQKARPTEEPGFAMDFDLDLESSWPMDHMSFVSNPMSPFLFSTMSDQPCSPLWAFSDGED
DRHTSLAAPAFSDCHKIFPCDSNSVTEKPMENDDNNKLLPPVVPQPPIENLDRFCVIKERMTQALRHFKELTEL
NVLAQVWAPVRNGNRYVLTTSGQPFVLDPHSNGLHQYRTASLMYMFSADGENDGTLGLPGRVFQHKLPEW
TPNVLYYSNKEYPRRDHAEHYNVRGTLALPVFEPALQSCVGVLELIMTSQKINYGPEVEKICKALEAVNLKSSEILE
PPYTQICNEGRQNALSEILEILTVVCETHNLPLAQTWVPCRHRSVLAHGGGLKKICSSFDGSCMGQVCMSITDV
ASYIIDPHLWGFREACVEHHLQQGQGVAGRAFSSHSMCFCPNITQFCKTDYPLVHYALMFGLTSCFAICLRSSHT
GDDDYVLEFFLPPRITHFSEQKTLVGSILATMKQHFQSLKIAAGVELEDGGSVEIIEATNERVTLRLESIPIAQSVQS
PPGPGGSPNMVEEVPKDPSEQQTMMNCNDMNEGGAHGDNAGGNIDQMSSLETKTMKKPSERKRGKTEK
SISLEVLQRYFAGSLKDAAKSLGVCPTTMKRICRQHGISRWPSRKINKVNRSLSKLKRVIESVQGAEGAFGLNSLS
TSPLPVVGSFPEPSTPNKFSQQASMRPLESQMKENEIDASKILEIEDPLLGGMTQNLEKVFNDKGPRRIRTGSGS
SEDSTNPTSHGSCYGSPPIESSHVKDISITSNNERCVVPRGSPESSPLQPINTLNSPSPIPIPDIVLTELQEPFGGMLI
EDAGSSKDLRNLCPSAAEAILEDQVPEACPTNNPPGCSSLAPKRPVDTLGKTVTPFAAKKEMKTVTIKATYREDII
RFRVSLTCGIVELKEEVSKRLKLEVGTFDIKYLDDDQEWVLIACDADLQECIDVSRSSASHIIRVLVHDITSNLGSSC
ESSGE
>Lupinus_angustifolius_OIV93669
MDSTHATFLTTLLVFKNTIREELIRSVHVYKVRGGKEREVEREFVFSESGSYGEMQASPILRNPKLKMIPGLLNDL
RVIYELPCKSEDRSIPSDSPGECQAKTNDCQPPKKTMPMLRQDLNCLPIDDDMSESLDNKLDVETLPGIMEKKR
RAHSDHIAKIALPDLVKYFDVPIIEASRNLNVGLTVLKRKCREFGIPRWPHRKIKSLDGLIHDLQEEARQQESEGEA
AAEAVAERKRMLESEKENIERKPSVDIKYETKRFRQEIFKKRHRARILQKQISTLSNT
>Lupinus_angustifolius_OIW14789
MGDDATILDDGTISMDFDYMNELFFDGCWLETTAEEFDFPLFDSSLFSWNVLENEHESQEALFSTQDNIVNVD
VSSNKQNQSEIQSLEGASEGFTRWWIAPTPNNPGTSYSVMEKLMKGLKCIHDLNRNKDMLIQIWVPVNRGG
KQILTTNDHQFCLGTRSTNLAKYREISMRYHFSTEEDGSKGLVPGLPGRVFKEKVPEWTPDVRLFRIDEYPRVDH
AQEYEVRGTLALPIFEQGSRTCLGVIEVVMTTSQLNYRPELESVCKALEAVDLRSSKLSSIPDLKASHKSYEAVLPEI
REVFRSACEMHKLPLAQTWIPCIQQGKEGCRHSEDNYLHCISPVEHACYVNDPSIRAFHDACSEHHLLKGQGV
AGGAYMTNQPCFSSDITSLSKTDYPLSHHARMFGLQGAVAICLRSIYNSMDDYVLEFFLPVNCIDSEEHKRMLTS
LSMIIQRVCHSLRVITDKELESETNLLVDEVTALADNRSRRTAICKEVLQDGMVDSLDTEEKSKGAKFSDLRQQQ
DLKGNLDSVGGCPTFGEGNLSSVGISKTEEKKRTKADKTITLQVLQQHFAGSLKDAAKNIGVCTTTLKRICRRHGI

KRWPSRKIKKVGHSLQKLQLVIDSVQGASGACQIDSFYSNFPDLLSPNLSGTDMLSTLKQSDNPNSLSPKDVLKS
PSSSCSQSSISSHLCSTMSEQQYHTSNAVEGNKDSIVVEDSANVASTRIRNEAELRSLVSQDNEEKLLPISLSQETL
GEQQHPKTKGNATKKEESHRVKVTYGDEKSRFRMPTTWSYENLLQEIGMRFHISNMSKFNVKYLDDDCEWVL
LTCDADLEECIDVCQSSENRTIKLALQVSNLGLRSSLEFT
>Lupinus_angustifolius_OIV98021
MDISPWDDLFEFGFTPQLPYNYQSNQLCFNGIDLDNLNFDDIDLPHLDENFGTEQKPLNMILSQPLPNCFSEES
FVSSVKKELEENNEEMLFLSSSNKKRTCSLGACVKEEVIENNGKMLLLPNTNSNNYRSKKRKSCTLEFEEIKKHFG
VPITQAAKEMNVGLTLLKRRCRELNIMRWPHRKLKSLEFLIENVKEMGLSNEVAMLEQHRKMLEKLPDLELTEE
TKKLRQTCFKANYKKRWCLV
>Medicago_truncatula_Medtr3g115400.2
MGDGAAVTSSATNTMMEAPPQDENLMDFDYMSELLLDGCWLEASADGSEFLLQSSPFSNPIFDPSFSWPVLE
NNEQPGNDVRHESQDVPLGTEQESRIIVNNAGSSDLQQCEFKTHLGEGVSRLWIPPTPNPGPGGSSITEKLIRA
LKWIKDFNRNKDMLIQIWVPVNRGDDRPILRANDLPFSLESRSLNLAKYREISVRYQFSAEVEDSKELVAGLPGR
VYRDKIPEWTPDVRFFRSDEYPRVDHAQECDVHGTLAVPVFEQGSRTCLGVIEVVMTTQQSNYGRELETVCKA
LEVVDLSSSGHSSPQNVKACVKSYETALPEIQEVLRSACEMHKLPLAQTWVSCIQQGKDGCRHSDDNYTHCISP
VEYACYVGDSSVRFFHEACMEHHLLIGQGVAGGAFMTNQPCFSTDITSLSKTDYPLSHHARLFGLRAAVAIRLRS
IYSISDDYVLEFFLPVDCNDGEEQKKMLTSLSMIIQRVCRSLRVITDKELEKNNLSSNEVMAVADIGFATDAVQSG
LQHKRMAVSLDGEEKFNETMSSKFSEQRRQQESLTLRGDADCGRECSPSVEGNFLSSLGVNRTGEKRRAKAEK
TITLQVLRQYFAGSLKDAAKNIGVCTTTLKRICRQHGIKRWPSRKIKKVGHSLQKLQLVIDSVQGASGSFQIDSFY
SKFSDLSSPNLSGSTLISTLNQVDNPVSLSIQPDPDSLSPEGASKSPSSSCSQSSYSSHSCSSLSEQQHHTNNVAA
GNKDSLVGEDSVDVVLKRIRSEAELKSLIPDNKSKLMPRSQSQETLGEHPKTEYRKSLLKTTRKASQKEDAHRVK
VTFGDEKTRFRLPKIWCYEDLVQEIARRFNVSEMSKFDIKYLDDDYEWVLLTCDADLEECIDVSQSSETSTIKLCL
QPSSNFIRSSLEFR
>Medicago_truncatula_Medtr3g465110.1
MANRKNNPSIEGATFKFDDDDAIDPNFFNMSNPTLNDIYMNNNNNGPTNLSQPNHHSNLSNPNPTNLSINR
NNNGPHQIYHGLGILQRGQQSSNFNGHFSNPLSQQNCHGLGILLQGYGSNEQFHTTLSQQNHQNVVEHGH
GSNIGNVPFNSNAMPQNDHGIQHGRSGNYDPFNYNLVSQENDHGILQHGGGRNYDPFNYNLISPENDYGIQ
QGQDGNNDLFSHAMSQQNHPGIQHGHGTTNETFSLPMVQNQLGQPNNNVSFGMNNFEAGSSSRTNENQ
QQTEALALDQWPPEQQQNSSSGSQVLRRIIHSNGFQYENLEIHGSLELITHAIHHVTPVNGGPTFDRMIDFSG
QGLGEIKNFLDEYCIARNSAGYFLIQDSMSTYYETLCTGYDWIDDINMEGPIDNFSDEIMEQEQEQVGEIETPEK
RNLSEQRKRCAQFTLADFVDYFHLPMEEAAARMNIAASTLKKISRRTKLRRWPHRKVKCLLRQIAILQNQLDRQ
DPATRKRTEEEIKKIEQEMIADCRGHRPTALNYVANFLPPEQLQQQQ
>Medicago_truncatula_Medtr3g104830.1
MNPSMEDEDLMFDCFENSLDPSFFNMSNPTLADLSIDNNNNNVGPTSLSQQTQHINLSINKNNGPHQNLQG
VGIVSNFNDVFSSPPSQENYYGHGGNSNNGNDPFNSNVLPQQNHSGNNDPFAYNLESPENDHHIQQVHGG
CNDPYMYNLVPPENHHEYIQQGQGGNTDSFSDVMLQQHGNGSTNETFSVPMVQDQYDQNSINFSFGRNSL
EVGDSSSRIYENQQQSIPEIQIPILLNQTELRALNQWPPTVTPYFCSCCQVLREIIHTDGMQFEKLEIHGRLALITH
AIHHQIPVNGAPSVYQMIDFCGKSLGEIKIFLAQYCVERHLAGYILMQDPMSEFYETLCTGLDWFSDINMDGLI
QNNSDEVEQEQEQMGDNGTLRNLSEQRKKSAKLKLSDLCDYFHLPIDEAADHEKVKLCPTVLKKTCRKAGLPR
WPHRKVKSLLKQIKLLAKQWEGQDPEARARTEREIKSLQTEMIKHCGGHIPTAFYNIAAYLPL
>Medicago_truncatula_Medtr3g091700.1
MDFFTQPEITQSFNWFCNVQPQQNDTDKIMGIAPFDDFFEFDWATQFSYQSNHFYMNDFDDLENFNFDFNL
PHLDQNNFEVDKKPLNVVLPELGHYNHKGETVGYVEASVKTESGFVSFVKKENEWENQELSSLVPLALQSSSTS
IIRKRSSSLQFDDIKKHFDVPITMAAKKLNVGVTFLKKRCRELNITRWPHRKLRSLMLLIDNLKDKGLTKEVAMLE

KQKKMLEKLPGMHLNDEIKKLRQACFKANYKNRKLIALCP
>Medicago_truncatula_Medtr3g435550.1
MTDYGYGDAKNVVPMWNELDAGFYSDKHIFSISNIIGENVAKEIVKGKRCREENSARMLTKETISEYFYMPISQ
AARELNVGLTHLKKRCRDLGIQRWPHRKLMSLQTLIKNVQEQGNEYDEKIRNAVEVLQKEMKKVEEKPDLQLA
ENTKRLRQACFKANYKKRRLMVMRLMDQSSISESSNVDDNHKMGY
>Medicago_truncatula_Medtr2g099350.1
MEYPYYSKGGGIGYWQSPGTQFEGPTSLDGGINNLVSEDMPNSFSELMNFDTYAGLCSGPSTTDQIMTNELPS
LASALYQLPDGFNPVEPNSGQFYGGNYNNFESSPIFGEKIVCQQMDTLLGFLDNSNDANNSSSRQKFDDPSQH
VNAFDMGNYVIARPQALISRPPALSLDERMLKALSFFKESAGGGILAQVWVPIKHDGQVLLSTSEQPYLLDQML
AGYREVSRTFRFSTEGKPGCIPGLPGRVFISKVPEWTSNVGYYNPKEYLRAEHARNHEVRGSIAFPIFDLHSGLPC
SAVLELVTTKEKLDFDRELEIICRSLQLVNLRTTASSRLLPECLSTNKRAALTEIVDVLRSVCYAHRLPLALTWIPCFYT
DGTRDETTRIQIKEGNSSSREKNILCIEESACYITDRVMEGFVHACIEHPLEEGNGVAGKALQSNHPFFYSDVKTY
DISEYPLVHHARKFNLGAAVAIRLRSIYTNNDDYILEFFLPINMKGSSEQQLLLDNLSGTMQKICKSLRTVSGAEIS
GMESSVVGFGKKNVPSFPSTSTRNSQVSLINEKDGSVQKLSLDTSNLRNNGNKPSCNQENNGSRRRVEKKRNT
SEKNVSLSVLQQYFSGSLKDAAKSIGVCPTTLKRICRHHGISRWPSRKINKVNRSLKKIQTVLDSVQGVEGGLKFD
PSSGAFVAGGTTTEEINENKSLLFPERSTTQDSEPMSQNAVSVPSEDENLENKWEGKLKETNTCSIDCSEDSKSF
AMDDCPEQAYIGSVLAKCDEWVLNNGGLREEKRKHSTIGQRTSSFAVDEMDTGADGDDDDVVEHNHPTSSS
LTDSSNGSGSMIHGSSSGYQNFKNQKPSKGKSTIVDSGSKIVVKATYGEDTIRFKFDPSTGYYKLYEEVAARFKLQ
DGSFQLKYLDDEEEWVMLVNDSDLQVCLEILNDMGTHNARFLVRDIPCTFGSSGSSSCYLGGSS
>Medicago_truncatula_Medtr5g099060.1
MEYGGGLVADGGVFGPMVGGGGGDQADIIEELLGEGCWIEASENSLMAMQQTTPQSQYMSNNNNIPMG
MGEGDHFNHHHHHHPHPHHQMECTAPAANHDDQQESGFVVGKRWWIGPRANPGPTTSVKERLVVAVGY
LKEYTKNSSNNVLIQIWVPMRRRSALIHTQNHYLQQESSSAPVSVNPNMNVHVRFFRSHDYPRHQQQQQYG
SLLALPVFERGSGTCLGVIEFVISNQTLINYRPQLDHLSNALEAVDFRSSHNMNIPQAVKVFEELYEAAVNEIMEV
LASVCKTHNLPLALTWAPCLQQQQGGGKGSSGASGCGVSTMSCCISTVDSACYVGDMDVLGFQEACSEYHLF
NGQGIVGTAFTTTKPCFAIDITAFSKSEYPLAHHANMFGLHAAVAIPLRSVYTGSAADFVLEFFLPKDCRDTEQQ
KQMLNSLSLVVQQACRSLHLHVVMDDNNNNNMNDNNSSADHDHDQFTFPTTNSYMPSSASEPLSQVDAV
SGCSTKDTSSSCSWIAHMMEAQNKGKGVSVSLEYLQEPKEEFKVTTCNWDREREDNVFSEFGQVLQQQQHD
QSSNSRASVVSVEAGEESPGACGRRSSSSSSGRKSGDKRRTKAEKTISLPVLRQYFAGSLKDAAKSIGVCPTTLKRI
CRQHGITRWPSRKIKKVGHSLKKLQLVIDSVQGAEGAIQIGSFYASFPELSNATANGGDGNDNSNNSFYNNNH
GDGIVTSLKSPPSACSQTHAGNKLPMTTTTAINHHHVVMTENPTGAPLGVDHAFMHASNINIQDYHQLQEDL
DTKQLLLHFNNNNQILPPRPTVAWNNNNSSSSTLLERGAFRVKATFADEKIRFSLQAMWGFRDLQLEIARRFNL
TDMNNLVLKYLDDEGEWVVLSCDADLEECKDLHTSSHTRTIRLSLFQASPLNLPNTFRNSSSSSPSS
>Medicago_truncatula_Medtr5g024070.1
MDSSLITLLLFNNTIQPELMRSVHVYRECDGGEKEVEREFVFSENGSYVEMKATPILRLLKSFVCEEIFEGYKNGV
WLCVFAFHAYNVPQFSHIPTFLLVTRNPKLQAIPNLLNDLHMIYKLEPKKEQKSTPESSASEELKGNNNDFQPPK
KVLPVLNQDLNCLPYEEDEAELLDDETDFGSSAGFLGKKKRAASDHVAKITLSDLAKYFDMPIAEASNNLNVGLT
VLKRKCREFGIPRWPHRKIKSLDSLIHDLQEEAKHQEMEDKDAAMAVIRRQKMLESEKENIEKRPFMDIQIETK
RFRQDIFKRRHRARVIEKQNLQLQAPKPSN
>Medicago_truncatula_Medtr1g069025.1
MGIAPFDDFFEFDWATQFSYRSNHIYKNDFDDLENFNFNFNLPHLDQNNFEVDQKPLNVVLPELGHYNHKGE
TVAESGYVEDSVKTGSGFVSFVKKENEWENQELSLVPLALPSSSTGIIRKRSSSLQYDDIKKHFDVPITMAAEKM
NVGVTLLKKRCRELNITRWSHRKLRSLMLLIENLKEKGLTEEIAMLEKPKKMLEKLPGMDLNDEIKKLRQACFKA
NYKNRKLIALCP

>Medicago_truncatula_Medtr1g100970.1
MSEPEEENQDFPPKTKTSLEEHGCSMDFDLDLETSWPLDHMSFITNPMSPFLFSNISDQPSSPLWTFTDGEDG
KLAASALSDCHKIFSCDSNSITEKLVEKDDNKTLLPPLLPPIENLDGYCVIKEKMTQALRYFKEWTELNVLAQVWA
PVRNGNRYVLTTSGQPFVLDPHSNGLNQYRTVSLMYMFSVDGENDGTLGLPGRVFQQKLPEWSPNVLYYSNK
EYPRRDHAQHYNVRGTLALPVFEPSLQSCIGVIELIMTSLKINYAPEVEKICKALEAVNLRSSEFLDHPFTQICNEG
RQNALSEILEILTVVCETHNLPLAQTWVPCRHRSVLAHGGGFKKSCSSFDGSCMGQVCMSTTEAAAYIVDAHL
WGFREACVEHHLQQGQGVAGRAFLSQTMSFCTNITQFCKTDYPLVHYALMFGLTSCFAICLRSFHTGNDDYVL
EFFLPPGITEFHEQKTLLGSIFSTMKQHFQSLNIAAGVELEENGSIEIIEATDEGIRLRTESIPIAQSIKSPPRPDASPN
MEDEEEGVPQDPSEVRGENVGGSVDPMSTLGNKNIKKPSERKRGKTEKSISLEVLQRYFAGSLKDAAKSLGVCP
TTMKRICRQHGISRWPSRKINKVNRSLSKLKRVIDSVQRAEGAFDLNSLGNNQLPIVSSFPEPSTLNKSSQQGSL
SNRPSEPQMKENEFDASKVSETNLQIVMENQLLGGRKHSLEKEVINGKGVTIQEIGKDRKRNRTRSGSSEDSTN
PTSHGSCHGSPPIEIPTIKDLFIPSNNEQHVVLRRSPEPGMQPTNALNSPTAHRMVDNVIAELQEPFGGLLIEDA
GSSKDLRNLCPSVAEAILEDMAPEACGNNFPGSSHLAPPKQCIDTINNSATPFAARKEMKTVTIKATYREDIIRFR
VSLNCGIVELKEEVSKRLKLEVGTFDIKYMDDDNEWVLIACDADLQECMYLSRSSGGSNIIRVLVHDITSNLGSSC
ESSGE
>Medicago_truncatula_Medtr4g068000.1
MEGQYSSKERGGISLWTPFDSGMKSSLNSDDMFNNISELMNFDSYAGWCNGSSSITDQTLTNDLSSFAYDGLN
LSEHINGPFFMTEIGGNYNVMDSYDEKVLLQEMETQLEFLDNNSNETNNNLDSQQNGSYDMCNYIISKSPSW
SLDERMMSALSFFKESAGGGILAQVWAPIKYGDDFILTTSDQPYLLDQKLAGYREVSRSFTFSAEMKMGSCCAG
LPGRVFNSHVPEWTSNVGYYHKSEYLRLDHAISHEVRGSIALPISDMNSEVSCCAVLELVTTKEKPNFDKELEFVS
HALQRVNLRTIMPPRLLPQCVSSNKRAALTEITDVLRAVCHAHSLPLALTWIPCCYSEGKGEESERIRIKEGHITSS
NEKCVLCIEESACYINDKMVGGFVHACSEHHLEEGQGISGKALQSNHPFFYTDVKAYDVSEYPLVHHARKYNLN
AAVAIRLRSTYTNDDDYVLEFFLPINMIGSSEQQLLLDNLSDTMRRICKSLRTVSEAELRGMEGSQYEFQKENVS
GFFPMSRGNSQIAFSSGDNDLFQMSFNETNLKNSGNQAAYSQATSGSRKQAEKKRSAVEKNVSLSVLQQYFS
GSLKDAAKKIGVCPTTLKRICRQHGISRWPSRKINKVNRSLKKIQTVLDSVQGVEGVLKFDPHTGGFVAGGSIIQ
QIGKHNKTLKFPEKASVPKLEDEDVLVSNSCEVELEKDNAISVDCIEDSTTWLWTKTQDCPKQTTIDYVLEKEQD
QCGLDNSSLHDIGSHSSFSMIELGFEEGKGADEHNNPTSSSMTDSSNASGSMVNGSSSGSQSIENQKHSKVKS
ICVDSESKFAVKANYRGDTIRFKFDPSVGCCQLYEEVAKRFKLQNGSFQLKYLDDEEEWVMLVNDSDLKECVEV
LSDIGTHCVKLLVRNIHGGSDS
>Medicago_truncatula_Medtr0022s0430.1
MQKSEEENHDLPQKSKPLDENVFSMDYDIDFETSWPLDHISNSMHPFFLQNISEQPFSPIWTFSDDDHNIAAS
GNNKNIITENRVENDDKHKRVTPHIGLPPLQNPHGYCLIKERMTQALRNFKELTELNVLAQVWAPVRNGSRYK
LTTSGQPFVLDAHSNGLHQYRTVSLMYMFSVDGENDEVLGLPGRVFQQKLPEWTPNVQYYSSKEYSRLNHAL
HYNVRGTLALPVFEPPGQSCVAVLELIMTSQKINYAPEVDKVCKALEAVNLRSSEILEHPYSQICNEDRQNALSEI
LEILTVVCETHNLPLAQTWVPCRHRSVLAHGGGLKKNCSSFDGYCMGQVCMSITEVAFYIIDSHTWGFHDACV
EHHLQQGQGVAGRAFLSQNMCFCRNITQFCKTDYPLVHYALMFDLTSSFSICLQSSHTGGDDYVLEFFLPPSINK
FCEQKGLLGSILATMKQNFQSLKVASGVELEEDGSVEIVEIINGTVHSRLESVPIIQSTKLSARHDDASPDMEEGV
PLDQSRQKIITNFDDINDIGNLDDYAGGSIDQIHSLKTETKKKPSLRKRGKAEKSISLEVLQDYFSGSLKDAAKSLG
VCPTTMKRICRQHGISRWPSRKIKKVNRSLSKLKCVIESVHGVEGAFGLDSPSTSTIPIAAGSFNEPSTSNKFEHH
TSLSIMPSEPKKNENYLDASKSEPQKPLGGMLIEGADSSKDLKNLCPSTDVVLEDQVLEASRIHPPCSDFVHMQ
MQHMDTLNFAAQKEMKIVTIKATYRDDIIRFRVSWNCGIVELREEIAKRLKLEVGTFDIKYLDDDQEWILVACDA
DLQECMDILTLSGSNIIRLVVHDILSILGSSVESSGE
>Mimosa_pudica_Mimpu11703S14211
MDCFFSDFDFPNLDFPHDFSSYFPTSHQETSTLDVSHMTMGLHGMDLMVPPPLPFESNLFYSNNQFTELENF

NYNFSGFDFNNFQLSCNDMVIDEKPCTTILPNHFQSFNLPISDDLLIKEMEMASSVVLPNESHHQYKNFEIQSC
QSATSLFDGNGFSPLIKQDDDDHETRIFRICYNKKKRSANLELDDIRNYFDVPITTAAKKLNVGLTLLKRRCRELNI
TRWPHRKIKSIKTLIDNVKELGLVEEVAMLEKHKRLMEKMPDVELSEQTKKLRQACFKANYKRRRFLALHAPA
>Mimosa_pudica_Mimpu1984S19289
MEYGAALLHNNSAFETLSDSTTTSVSEVGDFIDDLFANGCWLETTGHGGSSYNLTNPTSLINDPNLHNYWMTS
SDPNHPHHQHQQIYQQDQSEVSNFSLFQSESFVVANTEVGKRWWIGPKANPGPSSSVKERLVLAVGYLREYT
KNTNVILQIWVPTRREDDPSCNNKNLENYYRNVSKVYNHHHQLAGEEESAEDQCRVFLGKLPDHHQHQWAP
PASVRFFKSHEYVAYVNNFAPHVHHHHNQQHYDMLRGSLALPVFERGSGTCLGIVEIVIPNHQNNIDFHPQID
NVCHSLQVSFFFIILLFLLFPHLHSATYVYILICLQTVDFNKGACQNLISPAVKVYDELYLAALNEIVEVLTSVCKTHN
LPLALTWAPCAQQGKAGLCPNDNFVGCLSTVDSACYVADLDLLGFQEACSQYHLLRGQGNIVGTAFTTTKPCF
ATDITAFSKTEYPLSHHAAMFGLHAAVAIPLRSAYTASQADFVLEFFLPKNCHDGEEQKQMLNLLCIVVQQACRS
LHVAMDKQMQEVEEDEDFTFTVGDMFAAAAATKVGGGGGGGAHHHKEETLEFGSCNLEDGSSSKESSWIA
HMMEAQTQHKGKGVSVSLEYLEEPKQEFKVTTNWGFCNNSNSNNGQAFSDYGQVQVNSSSAAASRGMSVE
GGGESYAFRGGAGRRSSSGGRKSGEKRRTKAEKTISLPVLRQYFAGSLKDAAKSIGVCPTTLKRICRQHGITRWP
SRKIKKVGHSLRKLQLVIDSVQGAEGAIQIGSFYSSFPELSSTTNLSPTTTGPSQSSNISEDHSKIQHNNPHHQAAL
YNHHGGTTLKSPPSCCSQTSTIINAAVSGGGDILMAENPEASLMMMRTQHNLHEDMKHPSVVEVLAQPLQE
NNASVGGGGFRVKATFGDEKIRFSLHPSWGFRELQLEIARRFNLEDVSNIGLKYLDEEGEWVLLTCDADLEESKD
TYRSSKSRTMRFALFQTSPLNLANTTFGSNSSS
>Mimosa_pudica_Mimpu40297S10319
MEYPSSHKGKDSEYWSSPQAQVEEPASLDGDAKKPISEDMLTTFSELINLDAYAGWCNSPFPIDRVLANGLSSS
ASVPYASLDEFNLMGESCGSFLASDVNKNYNMTRSFPNSAEKAAFQQMEAQLELSDGGNDAGNLNYKHYPN
ESVEELGSSMISNYIVSRQPGWSLDERMLWALSLFKESAGGGILAQVWVPIKHGDQVILTTSEQPYLLDQMLA
GYREVSRTFTFSAEGRPGSVLGLPGRVFISKVPEWTSNVGYYTKAEYLRIEHAIDHKVRGSIAVPVFDLYSEMPCC
AVLELATTNEKTDFDRELEILCQALQVVNLRTAVTPRLLPQSLSSKKRAALMEIIDVLRAVCHAHSLPLALTWIPCC
YLKGIDNETMSALNKEDHSSSSDKLVLCIEESACYINDRKMEGFVHACVEHHLEEGQGTAGKAIQSNQPFFYPD
VKGYDVGEYPLVQHARKYNLNAAVAIRLRSTFTNDDDYILEFFLPVNMRGSSEQQLLLDNLSGTMQRICKSLRT
VTDAELSGISGSLVNFENGKVREFLPISGRNSQIGLMDVDKDSVSKISLKACNLRNNGIETTCNQEMNRSKRQV
EKKRSTGEKNVSLRVLQQYFSGSLKDAAKSIGVCPTTLKRICRQHGISRWPSRKINKVNRSLKKIQTVLDSVQGVE
GGLKFDPSTGGFVTGGSVIQEFDAHGRLLFSEKSMPMQDPECVANHTVSVPDVPYSERENSDIKLEDNDVYLA
DNQLVCRQSLIVPNSGEGAMKKAKAFVDFSQDSKSMALDDGSCQMVKSQGCPKQAYNLGPVAKKDGNAW
GLNMNGQAAKNSGPDNVSWSSSSLVADEIENIVDGDNGIVEHSHPTSSNVTDSSNGSGSMIHGSSSGAQSSE
NPRRPKVKSVLVDSGSKIIVKATYKDDTVRFRFDPSAGCFQLYEEVATRFKLHNGSFQLKYLDDEEEWVMLVNDS
DLQECVEILEDMSTSCAKFLVRDMPFLSSSGSSSSFLQGS
>Mimosa_pudica_Mimpu12208S14529
MQMQLQTHSKKEKAAFSLLRHPSLFLTHADTPSPYTHDIPATFMASLLTRTPFLVDEERACVAREGWEGEGGGK
RVRVLWECEMRATPILILSVHNNLGVCEEGYQNGVWLCVNPKLQMIPSLLNDLRIIHELNCKSKENGMLQLSPD
ERFQGNNNDSHQPRKILLQDLNCLPYPDDMPELQDREVDEESNVEKKKRAASKDVASIGLADLVKYFDVPIVEA
SKNLKVGLTVLKRKCREFGIPRWPHRKIKSLDNLIHDLQKNIQEVAENEEKENDGAVMAVTKRQKMLEYEKENI
ERKPSLDIRKETKRFRQDVFKRKHRARTMTKQSSSNYNSLHLKVKEEKATI
>Mimosa_pudica_Mimpu36607S22996
MGDPNPMNNVGPYHDDDTNMILNFDPTLLDSSNNIFPEVGDDAAVQALLDDIAPYLDPMFPSDHKFEAGPS
SQFHTHDLPPLPEQLPHPHQQNYNDNNNHNVVATSLLCPNLPVPFSCTFCQTLREIVYTDGVLHFSKLQIHGKL
GLISHAVLYQNNDVGSSAHQPFQIFDFSQKSIPQIKIFLIRHCLNQNSMGYFMMRDPLSLYYESLACGLDWAED
SNNDKLSDHGPDGVGTSGVKFGMEQQVNAENDMERTPTSGMKLSDMRNLFDLPIEKAAKKLKLCPTEMKKI

CRKHGLERWPQRKVKSIGRQISILRRALESGDGASTRSQTEAEIERLEQEMARHCGGFPPTSLNQESK
>Mimosa_pudica_Mimpu6940S04388
MGDPNPMNNVGPYHDDDTNMILNFDPTLLDSSNNIFPEVGDDAAVQALLDDIAPFLDPMFPSDHKFEAGPS
SQFHTHDLPPLPEQLPHPHQQNYNDNNNHNVVATSLLCPNLPVPFSCTFCQTLREIVYTDGVFHFSKLQIHGKL
SLISHAVLYQNNDVGSSAHQPFQIFDFSQKSIPQIKIFLIRHCLNQNSMGYFMMRDPLSLYYESLACGLDWAEDS
NNDKLSDHGPDGVGTSGVKFGMEQQVNAENDMERTPTSGMKLSDMRNLFDLPIEKAAKKLKLCPTEMKKIC
RKHGLERWPQRKVKSIGRQISILRRALESGDGASTRSQTEAEIERLEQEMARHCGGFPPTSLNQEPK
>Mimosa_pudica_Mimpu4886S10857
MGDPWTIVPYNDPYDYPIPENILDFQHHHFSLLPDPLLSNNNRHNNENNNPSPSFPQHSNAIFQSVNNDLNS
GHHNQQVIIHDTPMCEQIPHQSGNFEAGPSQIHGDNHRQEINNNHSNINYNNVHSLNFPEATQITQWPDQP
APFLCSCCQILREVIHTNGIQFVKLEIHGTLGVIFHAILRHNINAGSSGDQQTTMLDFSSFSMEGIKNFMKQYCA
EKNAAGCFTLPDPLSGYYEALCVGMEWNEDLNDELLDTFTDNNGGPSDDTEDPMDKVDKASLAIQRERAGK
MKLCDFSDVFHLPIEDAAKHVNLCPTVVKKICRKQGLPRWPHRKIKSIMRKVNILRGSLNSAEAASRAQTEAEIQ
RLHEEIVEHCGGIVPTSLNFNA
>Mimosa_pudica_Mimpu144438S15740
MGDNVVSPCGAMLDSSLDFDYMEEIFSDGCWLETAADGSDFLLRSPSAASIPLLDSSFSWPAFENNGHVSLEQ
QSKSLLMIGDYQEEAQVNINNNDNSNRSVGKGKDIIEVSGTGNNMSDNSEVVRRLWIGPMVNPSPECSVLER
LIKALVYIRDFNRDKDMLLQLWVPVNREGGRILSTRDLPFSLESSSPNLAKYREISAKYHFPAEEYSRGLVPGLPGR
VFKEKVPEWTPDVRFFRSDEYPRVDHAQEYDVRGSLAIPVFEKCSSICLGVIEAVMTAQKIKYFPELESVCNALQ
AVDLRSKNISSIQNDKECNKSYEAALPEIHEVLRSACEMHKLPLAQAWVPCNKQGKEGCRHTDDNYIHCIFPVE
HACYVGDPRVQGFHEACSEHHLLKGQGVTGEAYMTNQPCFSTDVTSYSKTDYPLSHHARIFGLRSAVALRLRSI
HNSTDDFVLEFFLPVNCIDSEEQKKMLSSLSMIIQRVCHSLRVISNKELENETNLSMNEVTVPAETGSARMEFEQ
CRNITSHGTKENSNETLTRQVSNMSQQEDTVLKGNLDNGWECSSFGEGGLPVAGLSKTGDKRRTKAEKTITLQ
VLRQYFAGSLKDAAKNIGVCTTTLKRICRQHGIKRWPSRKIKKVGHSLQKLQLVIDSVQGGSSAFQIDSFYSKFPE
LASPNLPGSSLFSTSKQSDLPIPSNTRPETGFMSPEAPSTSCNQSSSSSHSSSNMSEFPLHAQNIAGGKVPRVGD
DSVDGVMKRVPSELGLKSLSQDTAELLPTSQSQETPCVHPNTVLKGSNTVHPKEDTHRVKVTFREEKTRFRMP
KSWSYEDLLWEIAWRFNIDDMRKFDIKYLDDDSDWVLLTCDADLEECIDVCQHSQSSTIKLCLQDKKS
>Mimosa_pudica_Mimpu1764S07803
MVGNQNEALVGGWNLESELVPFPLTYQFSSPDLSGYGYGYLGLDWADEAALPWNIMEPDAMYETLSIEPTLSV
QDYGYYDIKKGLGLLNEIYDAFDSDNKAVYVDSGKELKEKDRWVKREELSGSTKLLSRKTISQYFYMPITKAAKEL
NVGLTLLKKRCRELGIRRWPHRKLMSLQTLINNVKELGKEGGQESEEKLRDALEILAKEKKMLEEMPDLQLEDN
TKRLRQACFKANYKKRKLMVSSMESECTRSECDYEDQTQDIKLMLPSTPLHFTA
>Mimosa_pudica_Mimpu5007S24951
MSDSDDEIQNPQITPQSKPLEPASVMDFDLDLEASCSWPLDQIGFSSSSNPMSPFLISATSDHQPSSPLWVFSD
VDDDRQAKLANASALSDVHRLLSCDSNSVAEKPVENEDTKKTPPPFVSLPPIPLETPEWYCLIKEKMTQALRYFK
EWADQNVLAQVWAPVRMGNQYVLTTSGQPFVLDPHSNGLYQYRTVSLMYMFSASGDNDGILGLPGRVFHQ
KLPEWSPNVQYYSSKEYPRRNHAQHYNVRGTVALPVFEPSGQSCIGVLELIMTSQKINYAPEVDKICKALEVGFL
PPLLSISCSCACFIHLLPVFVQAVNLKSSEILEHQFTQICNESRQNALAEILEILTVVCETHKLPLAQTWVPCRHRSV
LANGGGLKKSCSSFDGSCMGQVCMSTTDVAFYVIDAHMWGFHEACAEHHLQQGQGVAGRAYLSGNMSFC
GNITQFCKTEYPLVHYALMFGVTSSFAVCLQSSHTGRDDYILEFFLPPEVTDLNEQKIMLGSIVATMKQHFHSLKI
AAGLELEKECSVEIIEATNESVYSRLESIMITQPTELPHRPDALPNMREISPLDTSEQQMTFLDDMIGGGSLGDRA
GGTSDQRSPLETKNAKKPSERKRGKTEKSISLEVLQNYFAGSLKDAAKSLGVCPTTMKRICRQHGISRWPSRKIN
KVNRSLSKLKRVIESVQGAEGAFGLNSLSTSPLPMAVGSLPDPSNKFCRQASPSAQLAEPPMKDSELDASKVSET
SRQTELEHHLFRGRVQSPDTHEKSGSSQDFSKGPRGHRTGSASSEDGANPGSHGSCHGSPPIESSSPKDIFVTT

HTDQYVAARGSPELRPTSKQNSPPGYAVPNLMPTELQESFGGMLIEDAGSSKDLRNLCPAVADANVEDHLPEA
SAAFPPGSDLGFKQCANNLGDTMAPFAARREMKTVTIKATYREDIIRFRISISSGIMELKEEVSKRLKLEVGTFDIK
YLDDDQEWVLIVCDADLQECMDISRSSDSNIIRLLVHDITSNLGSSCESSGE
>Nissolia_schottii_Nissc3812S22614
MDNSFSPKGMGIGYWTSPQGQWEGLTLFDGGTKNSVSEEMVNKLSELMEFDTYAAGWCNIPSMPDHSLGN
GLSSFASVPCPSPDGFNLLEPGNGPIFMTEDSSNYKAMESSSGYGEKVVLQQMDTQLGMSDDANDTNNLDSK
PKFDGSFQQFNTFDVGNHMIPGPYEWSLDERMLRALSSFKESVGGGILAQLWVPMKHGDEFILTTSEQPYLLD
HTLSGYREVSRTFTFSVDGKSGSSPGLPGRVFKSHVPEWTSHVGYYSKNEYLRMDHAINHKVRGSIAIPITDLHS
ELPCCAVLELVTTKEKPDFDRELEVVSNALQLVDLQTTMPPRLLSQCLSDNKKAALTEINDVLRAVCHAHRLPLAL
TWIPCFYSEGIGDEAARIQIKEGHTGFNEKSVLCTEESACYINDRAVGGFVHACVEHPLEEGQGIAGKARQSNH
PFFYPDVKEYDVTEYPLVQHARKYNLNAAVAVRLRSTYTNDDDYILEFFLPVNMRGSQEQQLLLDNLSGIMQRI
STSLRTVSDAELSGIEGSQVENVSSFFPMSRINSDIALNGNRDSVQMMSLKPSNLRNNGIETAHNKAMNGSRR
QVEKKRSTVEKNVSLSVLQKYFSGSLKDAAKSIGVCPTTLKRICRQHGILRWPSRKINKVNRSLKKIQTVLDSVQG
VEGGLKFDPYTGGFVTGGSINQEIDAHKSFLFPEKHSAKDTVAVPPAPGSKGDNLAVKFEDDDICMDGNQLLH
SSVLISDSKGELKKDQASSVDFSEDSKSVAIWGMSKSSLGAKNSSHDIACHSPSSLIADEIEIHADGADGAVEHN
HLSSSSMTNSSNSSGSIIHRSSSCSSSENDKRSKVKSTCVVDSGSKMIVKATYREDTIRFKFNPSAGCLQLYEEVAT
RFKLHNGSFQLKYLDDEDEWVMMVNDSDLQECVEILDDLGTRNVKFLVRSMPSSLSSRQSNSCFLADSS
>Nissolia_schottii_Nissc119281S13298
MREGFMEEEFYDWEWEPQLTHKSKHLWLNEEEFPQLLTSFNDFNGFDLPSLDDYFAAVDQKPLNTGSNTGTE
SVVCVKQENQVEEEHHILPINIEYKKASSLDFEEIKKHFNFPIAEAAKRMKLGVTLLKRRCRELNIVRWPHRKIKSL
HLLIENLKEMGMENEVAMLEEHKEMLENLPWMELSQETKKLRQAFFKAKFKLRRRNLAPKP
>Nissolia_schottii_Nissc17691S16468
MSESEEENPDLSVPRSKAQQQQQQQQQQQQAGGGGGGVCAMDFDLDLEGPWPLDHLSFVTSNPMSPF
LFSISSSEQPCSPLWAFSDAEDDRHSRVAASAFSDCHKIFSSDSNSITEKPRENEDNKRLLPPLAIPSTLEILDSYCVI
KERMTQALRHFKELTEQNVLAQVWAPVRNGNRYVLTTSGQPFFLDAHTSGLHQYRTVSLMYVFSVDGENDG
SLGLPGRVFQQKLPEWTPDVQYYSSKEYPRRDHAQHYNVHGTLALPVFEPSVQSCVGVLELIMTSQKINYAPEV
DKICKALKAVNLKSSEILDHQYNQICNEGRQNALAEILEILTLVCETHNLPLAQTWVPCRHRSVLAHGGGLKKICS
SFDGSCMGKVCMSTTDIAFYIIDAHLWGFREACVEHHLQQGQGVAGRAFLSHNMCFCGNITQFCKTDYPLVH
YALMFGLTSCFSVCLRSSHTGNDEYVLEFFLPSRITDFHEQKTLLGSILATMKQHFQSLKIAAGVELEEDGLIEIIEVT
NERVHLRLESIPIAQFIQSPPVPDASPDVGEEVPQDPPEQKIMMYCNDINNVGGLGDNARGSLEHVSSLEPKNI
KKPSERKRGKTEKSISLEVLQQHFAGSLKDAAKSLGVCPTTMKRICRQHGISRWPSRKINKVNRSLSKLKRVIESV
QGAEGAFGLNPLSTSPLSIPVGSFPEPSTPNKFSQQASLSMRQSDPQVKENELDAFKVSETNRQAGTEDQLLG
GEGTQSLDKATNDKGGSPHVVGEEPKRTRAGSGSSADSTNPTSHGSCHGSPPIESSPVKDIFITPNSDQCVVIRS
PESSLQPINTLNSPTAYPVPDIVATELQEPFGGMLIEDAGSSKDLRNLCPSVAEAILEDLASEPFGTNPPCPDLAPK
QSMDALKNTVTPFAARKEMKTVTIKATYREDIIRFRVSLDCGIVELKEEVAKRLKLEVGTFDIKYLDDDHEWVLIA
CDADLQECIDVSRSSASNIIRVLVHDITSNLGSSCESSGE
>Nissolia_schottii_Nissc12880S13849
MFFNLAMKTTFLTTLLVFKNTIREELIRSVHVYEVKDGKVKEVEREFVFSESGSYGEMRATPILMSQGNFCVSEV
SEGYKDGVWLCIFAFHSDQMPQFSSIPSLLLVSRNPKLRTIPTLLKDLGMIYKLDCKSEDKDVLEDKDVLSDSTGE
ECQDNSKDFQPSREVIPVFEQDLNCLPYEHDMSELPDIKLDDETLPGSVEKKRRAASGRVAKIELSDLAKHFDM
PIVEAARQLNVGLTVLKKKCRDFGIPRWPHRKIKSLDSLIQDLQTRTLQEEVEHQGPEDQAMDVAEKRRMLESE
KENIEKKPFMDIKSETKRFRQDMFKKRHKARAHGKQNSAAAASTNKKTTT
>Nissolia_schottii_Nissc524265S09652
MLPNPLTSHVLFNNHNSSMDSSKTTLLTNLLVFTNTLQQELIRSVHVYRKCDGEEREVVEREFVFSESGSYGEM

QANPVLTPLQFRVPQVSQGFQNGMWLCIFAFHADQRPQFSHIPDLLLLTRNPKLQEIPNLLNDLKMIYKLDGKE
EEKDDPQDSPQEEQQESSNDSEQPHKELLVFHQDLNCLPYDEDTSVSQDNEINDESSSGLAAKKRRAVRAPSD
HIAKLALSDLVKYFDMPIVEASRNLNVGLTVLKKKCREFGIPRWPHRKIKSLDSLIHDLQEEAKNQESKDKDAVM
AVAKRQKMLECEKESIEKKPFMDIQIETKRFRQDVFKRRHRARAIENKGSTVSST
>Nissolia_schottii_Nissc2388S07083
MPESGGKNSDLPSKSKPQEEHGFAMDFDIYLESSWPLLDHVSNNPMSPFLITTSSEQPFSPLWTFSDVEEDHK
HVRISGSGDSNAVAEIPVENDDKKNLVPPFVPLPPLKNPDGYYLIKERMTQALRYFKELTEQNFLAQVWAPVRN
GNRYALTTSGQPFVLDPRSNRLHQYRTVSLTYMFSVDGGNDEILGLPGRVFLQKLPEWTPDVQYYSSKEYSRLN
HAQHYNVHGTLALPVFESPGQSCVGVLELIMTSKKINYAPEVDKVCKALEAVNLRSSEILEHPYAQICNEGRQNA
LAEILEILTVVCETHKLPLAQTWVPCKHRSVLAHGGGFKKSCCSFDGSCMGQVCMSTSDVAFYIIDAHTWGFHE
ACAEHHLQQGQGVAGRAFLSHNMCFCGNVTQFCKTDYPLVHYALMFGLTSCFAICLQSSHTGNDDYVLEFFLP
PMITDFYEQKTMLVSILTTMKQHFQSLKIAAGVGLEEGCSVEVIEAINGRNHLSLESIPVNQSAKSPPRLDVSPHK
SEGVPQGQGPSEQKIMTYFEDINDGGNLGDTAGGAIDQISSLETKIKKKPSERKRGKAEKTISLEVLQRYFTGSLK
DAAKSLGVCPTTMKRICRQHGISRWPSRKINKVNRSLSKLKCVIESVQGAEGAFGFDSASKSPVPAAADSFTEPS
TSKMFNHKASLSIRPSEPQMNENDFDIFRMSKTNRQAGLEDQLLGLGGKQSPEKAIHEEGGSSEESANPNAPG
SWHGSPPNESSPPKDIFGTLKNEQCLVLRDSMESTMQPKNTLHRATVYTALNVETTEQEEPFGRMLIEGVGSSK
NLRNLCPSADAVLEDQVPEASGINPPCRSLAPNQCLEDTLKNAVTPSVSDRKEMKSMTIKATYREDIIRFRVSLN
CGIVELQEEISKRLKLEVGTFDIKYLDDDSEWVLISCDADLQECIYILRSSGSNMIRLVVHDLASILGSSCESSGGLG
>Nissolia_schottii_Nissc1008S04822
MEYPFSPKGRGTGYWASPRTQWEGSTSLDCGMRNSISDVVPNSFSELMNFDPYASLCNSPSSMADQVLANGI
PSFVSVPYPIPNGFNLGEQHSGQFFMAEVGGSYNNAAGSSPNYGEKAVFQQMDTLLGFFDDADANNLNSRQ
KIDGSFQHLDAFDSGNCIVSRSPSLSLDERMLKALSFFKESAGGGILAQVWVPIKHGDQFILSTSEQPYLLDQML
AGYREVSRTFTFSAEGKSGSSPGLPGRVFISKVPEWTSNVGYYNKTEYLRMDHAISHEVRGSIAFPICDVDSEQS
CCAVLELVTTREKPDFGKELEIVCHALQNVNLTTTVPPRLLPQCVSDNKRAALTEIIDVLRSVCHAHRLPLALTWIP
CCYTEGTGGNDTRIRIKEGHSSSSEKSILCIEESSCYITDRAMEGFVHACIEHHLEEEKGIAGKALQSNHPFFYPDV
KAYNIGEYPLVHHARKYNLNAAVAIRLRSTYTKDDDYILEFFLPVNMKGSSEQQLLLDSLSGTMQKICRSLRTVSE
AELSRIEGSQVGFEKKNAPSSFPMSRRNSQVSLINSNHDSVHKMSINTSNLSNNGIEPSHSQETSGSGKQVEKK
RSEKNVSLSVLQQYFSGSLKDAAKSIGAVCPTTLKRICRQHGISRWPSRKIKKVNHSLKKIQTVLDAVNGVKGRLK
FYPSMGALVDAGGSIVQEIDHKAVPSPDKSTIKEKGKWVMNKGDLRVEKSNGSNSLAAEEIDTSVDGDDDGFV
QHNHPTSSGLTDSSNGSQSLENKIKNSKGKSTSIIDSGSRIHVKATYGEDTVRFKFDPSACCSKLYEEVATRFKLQN
GSFQLKYLDDEKEWVMLFSDSDLQECLEIMDEAGTRSVKFLVRDMTYILSSSSSSSCYLGCSTS
>Nissolia_schottii_Nissc337S21912
MRDGAVSSSTMMEAPPPPPEGGTTTSMEFDYNMDELFFDGCWLAATADGSDLLMQNPPFSNPLYDPSSISF
PWPSPLDTIEPPHNGVHHQQHEPHEAQPQPPLCHDIVKAAAAAADGAATNCSNQLQQLQSEIQSLEGSSEVL
RRWWIAPSPIPGGPGGSYSVVEKLVRALMCIKDVIISKDMLIQIWVPVSRGGRQILTTNGLPFSLETGSANLAKY
REISVAYQFSTEEDSKGLVAGLPGRVFKEKVPEWTPDVRFFRSCEYPRVDHAQEYDVRGSLALPVFEQGSGNCL
GVVEVVMTTQQIKYGHELESVCKALEAVDLRSSKLPTIQNVKACEKSYEAALPEIQEVLRSACEKHKLPLAQTWIS
CIQQGKEGCRHSEDNYFHCISPVEHACYVGDPSIRFFHEACSEHHLLKGQGVAGEAFMTNQPCFSTDITSLCKT
DYPLSHHARMFGLRAAVAIRLRSIYCSTDDFVLEFFLPVNCIDGEEQRKMLTSLSLIIQRVCHSLRVISDEELEEETN
LSVDEVIALADSRIARTEKVWTEPPQSVMVASLGTEEKSSETMGGSFSDPRQQEENHVLKGNLDSAGDCSTFES
VGISKTGVKKRTKAETTITLQVLRQYFAGSLKDAAKNIGVCTTTLKRICRQHGIKRWPSRKIKKVGHSLQKLQLVID
SVQGASGAFQIDSFYSKFPDLASPGTSLFTTLKQSDNNPNSLSTQPDPGGSLSPEGTSKSPSSSCSQSSISSHLCSS
MADQHHTTNGVAGEHSGDHGVLKRIRSEAELKTLIEDNRAKLIMPRSQSQELLGQNPKTEYHHHRRTLSEANS
KKENAHHRVKVTYGDEKARFRMPKNWGYEDLMEEVSRRFNISDLTKFDVKYLDDDSEWVLLTCDADLEECIDV

CQSSESSSTIKLCLQNSNNCMRSSFAEFR
>Nissolia_schottii_Nissc91520S32148
MLMLANKAFFFVVLASSINSNGYRSVDWLPEFPPFMQDGYLDYPWDPLSETLGIEPISTVQEYDIYDESVKGFS
AWEEVYENWEEGGRGDDNMKKKAKRGRGEEMRSGSTKMLSRKAISEYFYMPITKAAKELDVGLTLLKKRCRE
LGIRRWPHRKLMSLQTLINNIQEIGGDSEDKLRNAIEILEKEKKLLEEMPDMQLEDTTKRLRQACFKANYKKRRL
IGSRDSSSCLA
>Nissolia_schottii_Nissc524354S25958
MISFCRKSIEDVKNFLVQYCVGQTSAGFYILQDPLSSYYEALCIGMEWIEELGDDTDDLSPNSDDDNEPEGDKRV
GKGRRRAHYSAQRDRAGKMTLEDLTDYFHLPIEEAAKQVNLCPTVLKKICRKAQLGRWPYRKIRSIAKKISALKR
TLESNNDERSRARIQGEISRLEAKIREHCVGLYPTALVIP
>Parasponia_andersonii_PanWU01x14_068870.1
MEYSAQLIVPKIENPHGFDWLFHENPSENLLPDFPPVQTFFEANYPLPFSCLYDQLPSIDFQNFEDIDGGFFFDN
AFSYCEDPMVDEKPKTSLVTSFSHNNKNSTSDQIGFEVSMLIESEIFGSSEKNEEETRSCGRKKKKVVELELDDIQ
KYFDVPIKEAAKELRVGVTRLKKRCRELNIMRWPHRKLKSLKYLLNNVKKMGLNSNEIMMLEEQQRLLEVAPD
MDLTERAKKLRQACFKANYKSKRSLEAARG
>Parasponia_andersonii_PanWU01x14_071980.1
MADPGAIMSYSDLYDGPFSENLSNILTDYENPSLLDLPEPDHIGGQGPSNTSDNHNPNHVTLEQNSATIWDLPI
DFNVDQCLGSGPNNQPGPSRIRDHQETSNNDPDDQAIEENLELPQVNNSNNVGGNSRTLPTWPSPPVPFNC
TCCQVLREIIHVNEFNNYMKLEIHGRLGMICHVIWESRSSTNNNVTTSTSSGNDPQFEMFDFCRQSIEDVKRFL
VEYCSRRKVEGYVMLHDPLSIFYEALCVGLEWDELSNNDFDFVPTSPPHNSGGAEENYTEQQGQEEQQVTHQ
QEQTVETRPETTQIIDVERNSRISLAEQRQRTGKLTLMDLREYLHLTIEDAAKAMNVCPTVLKKICRRHNLPRWP
YRKIRSIKRQISSLRPNLESNDAAVREQAQAEIDRLRQEATNICAGVDVVEL
>Parasponia_andersonii_PanWU01x14_111140.1
MEYGAMMPSNAYGTSFSDATMEVDFMDQLLLEGCWDPAVAAAATGLNFTQPAAPCSSAPRSLIETSHYMPSF
ESSNTSTAHLITTHHHQIYQDHQETEEGGSDHLQENHSSESNNNNFFMNEANEVGKRLWIGPRPNPGTGPCS
SVKERLTLAIDYLKECTRDRDVLIQIWVPIRRGGRQILTTYDQPYSLDPNCKSLASYRMVSREYQFLCEEDSAESV
GLPSQAFLGKLPEWTPDVRYFRSYEYPRINYAKQYNVRGSLALPIFERGNGTCLGVVEIVMTTQKVNYRPEIEYV
CQALEMPITLQAVDLRSFQNFSPPAVKAVDLRSFQNFSPPAVKVCDELYQAALSEIVEVLVSVCKIHRLPLALTWA
PCHQQGKGGCRHSDENYTRCVSTVDLACFVSNLEILGFHEACSEHHLFRGQGIVGTAFTTNKPCFATDITAFSKT
EYPLSHHARMFGLRAAVAIPLRSVYTSSSDFVLEFFLPKDCQDPEEQKQMLNSLSIVIQQACRSLHVVEKELEEE
QIMLPNGDPAIVASSEGGLKLGTPTTATTTNTTTCPVGDQSSGKESSWVAHMMEAQQQGRGVSVSLDYLTEE
PKEEFRVTTHWDNLNHGDLSNGQVFSDFGQLQQCSGSNGSIEGGIDSYSFGGRRSSGARKAGEKRRTKTEKTI
SLPVLRQYFAGSLKDAAKSIGVCPTTLKRICRQHGITRWPSRKIKKVGHSLRKLQLVIDSVQGAEGAIQIGSFYSSF
PQLNSPKFQGNSNDSFSSISPSDHHHHHHHLNNQSNSQPESGFFIHGSAMKSSPSMSSQASGLQQSTTTFSCF
NGDPLITEDPSVILKRACSSDEELFASNQDKANLLSRSQSYKALSDQNRILDQTLLPLPDNNERINLTDGFAFRVK
ANFGEKKIRFSLPPKCSFRDLQLEIMRRFALDDITMIDIKYLDDDREWVLLTCNADLQECLEIHRQSQNRTMRICL
QQAPSDQLIEESPFGSSSSPC
>Parasponia_andersonii_PanWU01x14_175040.1
MVAKDENPFDPFSTQFLSSPLNFSGDGYQFSTNFEQNWQNELPIQESILLDAVPLMEYYPTDPLYSTVELEPSPSI
IQDDILINNGNGLEVWDDIGVLGFEANYNQKKPLLLLCNDPSNINGDDQKGKEKMKKEKKIVERRLACRISSEE
KSSTSASKTLSRQVLSQYYYMPITQAAKELNVGLTLLKKRCRELGIRRWPHRKLMSLKTLIKNVQDLGKEEGGHQ
ENDSKLKNAIELLEREKKLVEEVPDIQLDDTTKRLRQACFKANYKKRKLRIMDSDHDHQSGLNTKYLEEEDEEIKS
LLSDLFSSTNHLF
>Parasponia_andersonii_PanWU01x14_226510.1

MDSSKTHFLLSLLIFKNTINRELIRSLHVYRLINGMEKEVEREFVFSVNGAYVEMEADPLLRLQKFEISEVFEGFVN
GLWLCIFAFYAHHPPPPLLTCIPSLLTLSRNPKLRAIPSLANDLETIFHMICVADDKEPSRVLSERTCQRNNQNHH
QLRRNVPVLDQDLNCLPYPVTPSELSDDQQIEQSSSGTMEKKKKRATSKDIARIALSDLAKYFDLPIVEASRNLEV
GLTVLKKKCREFGIPRWPHRKIKSLDSLIRDLQEEREWQQQDNKAAAIAVAKRQSMLENERESIEKKPFLEMKTE
TKRFRQDVFKRKHRARVLGTQSLFLPDN
>Parasponia_andersonii_PanWU01x14_262620.1
MSESEEETTPSFPPKSTSSKEVVPDVREVLMDFDLDLDNPWPIMDQIAFVSNSMSPLLFSSPTDQPCSPLWAFA
DAENNDDNNNNNSNNSDKLGRLVSSAIADSSCFSCNSNSVAERPVGNEERKIAPSPFLGLVPVDNPNGYYLIKE
RMTQALRYLKDSTDQHVLTQIWAPVKNGCRYVLTTSGQPFVLDPDSNGLHQYRMASLLYMFSVDGETNEVLG
LPGRVFRQKLPEWTPNVQYYSIREYPRLDYAQHYNVRGSLALPVFEPSGQSCVGVLELIMTSEKINYAPEVDKVC
KALAGSKLEKCRYIRSYNHEICNEGRKNALTEILEILTAACETHKLPMAQTWVPCMHRNVLAYGGGLKKSCTSID
GSCMGQVCMSATELAFYIIDAHMWGFREACLEHHLQKGQGVAGRAFYSRNLCFCGDITQFCKTEYPLVHYAR
MFGLTSCFAVCLQSSHTGNDNYVLEFFLPPSIANASEQSALLGSLLATMKKHFQSLKVASGIGLEEEGIVELVKVS
AIGGLESSLECIQITKSAESPPGPIALPNGGDMVQQDLLKQQLTVDLDTANVGEDAAHDGGSVNHVSVSENKET
KKISERKRGKTEKSISLEVLQQYFAGSLKDAAKSLGVCPTTMKRICRQHGISRWPSRKINKVNRSLSKLKRVIESVQ
GAEGAFGLTPLATSPLPVAVASISRPSTSNGTNHQHSPSNIPSEPPMERKDSPTTSSPRRGQVETEDQLQGGGIL
SQKEFIHENGGFFSPEINRGSNHSKSGSGSREASVGTPTSHGSCQGSPANGSIIVKDQFVYSIHEQCIKVEGSPES
APQPTDELHFSVPDALVTAECQEPFGGMLIEDAGSSKDLRNLCPAVDAVLDEPVPEYCWINSACSDLAPKQTSGI
ITQTQPNVIVRLRQEMKSVTIKATYREDIIRFRISLSCSVVELKDEVAKRLKLELGTFDIKYMDDDQEWVLIACDAD
LQECMDVCRSSGSIMIRLLIHDIMPNLGSSCESTEE
>Parasponia_andersonii_PanWU01x14_340330.1
MEHPFSSKEKENEYWSSSRAQVENLASFDGVPRGMLPEDVFNSFSELLTFDAYAGWSNSPAVTDQVSATYGLS
SLPSTTYVPMDGSNFAEQNVGALPVTEVGGNFNLPRSSFNYGDKIVFQPVNSEMGMVDSTICRPTRYSLDER
MLRALSFVKESSGGGILAQVWVPVKHGDQLFLSTSEQPYLLDHMLAGYREVSRMYTFAAEGKPGCFPGLPGR
VFISKIPEWTSDVGYYKKNEYVRVEHAYNHQVRGSLALPVFESDPGMPCCAVLEIVTTKEKPNFDTEMEIVCHA
LQAVNLRTNPPPRLVHQCLSKNQKDALAEIIDILRAVCHAHRLPLALTWIPCCYGEGAAGELAGVHVREGKTSSS
EKCILCIEKSACYVNDSLMQGFAHVCVEHHLEEGQGLAGKALQSNLPFFFPDVKTYDISEYPLVHHARKFGLNAA
VAIRLRSTYTGDCDYILEFFLPVNMKGASEQQLLLNNLSGTMQRICKSLRTVSDVEVGAIPSDAFQKGSVPNFMS
LPGESSQMVLSDSDLNPTDELPLKVSNRRNEGIEADGVHEQAIRGSRRQTEKKRSTAEKNVSLNVLQQYFSGSL
KDAAKSIGVCPTTLKRICRQHGISRWPSRKINKVNRSLRKIQTVLDSVQGVEGGLKFDPTTGGLVAAGSIAQELD
AKKSLFFIEKTSSLQNSDPMSAVKLEEDECCTGGVDPNSGEIRKPNIVSLTNSTKDSKIAIEAGSRLPICDARSGALS
DKACLGSYHAKEGKTSCQSKCSSKFENSKYYHVSQSSGSLGVCDEMDTAGDGDDELIEHNLPASSSMTDSSNG
CGSMLHVSSSSSHSQSFEERKHPKGKMSCGDSGSKIIVKATYREDTVRFKFDTSAGCSQLYEEVAKRFKLQTGTF
QLKYLDDEEEWVMLVSDMDLQECLEILDDIGTQSVKFQVRDMPCAVGSSGSSNCFLAGGS
>Parasponia_andersonii_PanWU01x14_370700.1
MEDGVLSPATILGAPADYPMDLDFMDELFLEGCWLETRDGSEFLNQNPPSSNPLFDPLFWPTLEPDGESNAN
PSPKSNQEERHRSLFVESQGKSPLHTLPPTRATTDVVKYSGVSEAHITEGSSELSRRWWIGPKANPGPSSSVME
RLWRALMYIKDVIRDKDILVQIWVPVHKEGRRVLTTRDLPFALDDSGSKLARYRDISVKYQFSAEEDSKDLVLGLP
GRVFSGKVPEWTPDVRFFRNDEYPRLIHAQQIDVRGTLALPIFELDSRTCLGVVEIVMTTQKIKYRPELESVCKAL
EAVDLKSSEVLSTQNVYNEYYQAAIPEIQQVLRSACDTHRLPLAQTWVPCIYQGKEGCRHSDENYGQCVSTVD
HACYALDPQVQSFHEACSEHHLFRGQGVVGLAFMTNQPCFSADITSYTKTEYPLSHHARMFRLQAAVAIRLRSI
HASAADFVLEFFLPVDCKDPDEQKKMLTSLSLIIQQCCQSLRVITDKELEEESCSRVDEVVVPSNLRPARNTCFTE
APQNDTDLSLFPEEKKPREISDGRLSKLSDNQRDSSLKPSVECVEECSTVGEGSFSSVGVGKTGERRRAKAEKTIT
LQVLRQYFAGSLKDAAKSIGVCSTTLKRICRQHGIKRWPSRKIKKVGHSLQKLQLVIDSVQGASGAFQIDSFYTNF

PELASPNVSGTSPFSTSKLNDHPLPSNMQPGDGGIFSVQAATAAATSKSSSSCSQSSSSSHCCSSRSQLHPQTW
NNVTSSDDLIAGENSGGGDDVVLKRVRSEAGLNACSEDDRKLLPRSQSHKSLMKHHKTDKWFPPSSAKNNNN
GARIPQQQGEFQRVKVTYGEDKTRFRMQNNWGFIDLQQEVGRRFGIQEMVKFTLKYLDDDSEWVLLTCDAD
LEECFEVYRSSQNATIKLSLQPSRHLYRGCLRGNDPL
>Phaseolus_vulgaris_Phvul.003G189900.1.p
MESHPLMGWSSYYQIDEKSFPFTCQFSYSEISGVYPPLHDLAIQEYHHDAVPLMEYYPSDPLYETLAIEPTPSLRA
DYDFYDINKGLSVWNEVDAVFDTEVGFLFRKNEERGGGLEEMMENKQGREERTNSSSSRMLSRKTISQYFYM
PITQAARELNVGLTLLKKRCRELGIRRWPHRKLMSLQTLINNVQELLKEEGPESEEKLKYAIETLESEKKLLEEMPD
IELEDNTKRLRQACFKANYKKRKLGQREPQCSSWSACASHFSTYIP
>Phaseolus_vulgaris_Phvul.009G080800.1.p
MDNNWNNSSDLFDNSPLISNYIEPDPALVYIFNDQPNVLPQQRNAEQNDPFVVEQDHVSFTGLTIEDVSTHSD
NDITTGGVSKLDQGGWSMRKRKFSFMDSMIEDHPMHDKDNFPNGENMSAHINLNQLRVWPPAPFDIDIDT
SFDPFLHNLIDRDISIFDLSNDELVSPSLQQNALPQHQQQVPQNALSKQQNALPQLLHTLPQSQNVYQFGVEQI
HVPILNPTFQEQSLHVNCNFTTGGEVPNIDVGNQSMAEHPLLRHGILNEEDVIAIDHWPSSSKLLSCSCCQILR
QIIHTDGFKFEKLEIHGSLGVIGHAIFHIQDMTPGGEPPREIYQMIDLCGKSIEQIRSFLAAYCKDQTRLGLVTLDD
PLSSYYDTICTGLDWVEHYNDGDDDLSPPNSTEPESDSEPEPEPEPEPEPETIPESDPVPEPHNGEASTTKPKPK
RNMAVQRKRVPKMTMNDLSPFFHLTIRDAADKLDVSDSVVKKISRLGNLKRWPQRKLQSLAKDVRVLRKALN
SPYEGTSKRVRQEIQRLQREMVAVCGGVAPTGIEMLQLEEE
>Phaseolus_vulgaris_Phvul.009G115800.1.p
MEYGALVQNGACGSFSDIFAPETDFMDELFAEGCWVETRVGCGGSYLNCGTEPSKSNSCMEIIFEEESEAESLM
VGKRWWIGPRANNPGPSSSVKERLVVAVGYLKEYAKNSNMVIQVWVPARRGCAVGIHQDSYTTLDYTNITNN
NNGDATRVFQFQEEWLSDHWTPNIRFLRTHDYSRVQQYDLRAGSLSLPVFQRGSGICLGVVEILLPNNVNPDL
HNLQGVDFRSYSLQNFIPPAMTVKGFDELYQGALNEIVEVLTCVCKAHELPLALTWAPCIQQGKSGCGHSNEEN
YVSTVDPASFVADVDVLGFLDACSEYHLLGGQGVVGTAFTTAKPCFANDITAFTKAEYPLAHYANMFGLHAAVA
IPLRSVSADFVLEFFLPKDCHDTQDQKQMLNSLSMLVQQACRSLHVVMDKEEEEELVVHHHHHPHDSKEMES
SSWIAHMMEAQQKGKGVSVSLEYLQEPKQEFKVTTNCSEQVFSELEGTGSVGVGVGGRRGGRKSGDKRRTKA
EKTISLPVLRQYFAGSLKDAAKSIGVCPTTLKRICRQHGITRWPSRKIKKVGHSLRKLQLVIDSVQGAEGSIQIGSFY
TSFPELSSANGVSESTKINNDNSKFYSENGLLSNQGVTTTTSTSPFKSPTSSCSQTCNPTSNQSTAIINNNNNND
DIILMSENQSLIGAPIQVQGEARHFTHHPPIPISIPIQSLDALPPLPQTNSVWNTGSGSFRVKATFGDEKIRFSLQP
NWGFRDLQMEIARRFNLNEISNSQLKYLDDAQEWVLLTCDADLEECKDINRSSQSRTVRLFLFHASPLSHSTNA
FGGTSPT
>Phaseolus_vulgaris_Phvul.009G186900.1.p
MNCYYPHDILFETFPLEPAPLSFHDYDFSDVKNVLSAWKEVDCSDHKPAFSSSNDGESVNAMREEMKGKHRRE
ERISNSARMLSRKTVSQYFYMPISQAAKELNVGLTHLKKRCRELGIQRWPHRKLMSLQTLIRNMQEQGQGEGP
QNDEKIRTAIEMLEKEKSLVEERPDLELEDKTRRLRQACFKANYKKRKLMGMRFMEPQSSLDGLSDKY
>Phaseolus_vulgaris_Phvul.009G051600.1.p
MDFVSERDFPNLEIIPHTFNSCFFSAQLYPTDKTMEVSLSPLEGFSDHDWTQQLSYSSKQLSLNEFPDLENLDIDF
SLPPLGEDLEEVDQKPLNIIVVPENGRCSREKGFGDALLAIESGSAICVKKEEVQNIEQMLVPRARGRGSGSSGSS
KKKKPCALEFEEIKKHFDVPINEAAKQMNVGLTMLKRRCRELNIMRWPHRKLKSLQLLIDNVKELGLAEEVSML
EKHKRLLEKLPGLEISAKAKKLRQACFKANYKRRRYMGMAVQA
>Phaseolus_vulgaris_Phvul.009G011200.1.p
MMRDGDVTSSSATMMEASPPDGTTTSMDFDYMGELFYDGCWFEASVDGSDFLLQSPSYSNPLFDPSFSWP
ALETNHNESQGAAFGTQEEGHNNNVVAARGGGGGGQQFQPETISIEGASDGVRRWRFAPTPSPAPGPSIME
KLVRALMRIKDYNRNKNMLIQIWVPVHRGGRPILAANDILFSLDSRSMNLAKYREISVRYEFSAEEGEVKELVPA

EEGDSKELVLGLPGRVFRDKVPEWTPDVRFFRSDEYPRLDHAQEYDVSGSLAVPIFEQGSKMCLGVIEVVMTT
QQINYGPELESVCKALEAVDLRSSKQLSIQNVKACNRSYEAVLPEIQQVLRSACEMHKLPLAQTWIPCVQQGKE
GCRHSEDNYLFCISPAEHACYVGDPKIRSFHESCTEHHLLKGEGVAGGAFMTNQPCFSDDITSLSKKDYPLSHHA
RLFGLRAAVAIRLRSIYNSTDDFVLEFFLPVDCNDSEEQRKMLTSLSIIIQRVCRSLRVISDKELVEAKLSIDEGISLAD
SGFDRTAICEELQHKGTVASLDTEEKLSETTGRKFSDPMRQQKSPILKGNLDCVRESSTSVEGNLSSVGMSKMG
DRRRAKAEKTITLQVLRQYFAGSLKDAAKNIGVCTTTLKRICRQHGIKRWPSRKIKKVGHSLQKLQLVIDSVQGA
SGAFQIDSFYSNFPDLASPNLSGTTLFSTFNQTDNPNSISTQPDPGALSPEGTSKSPSSSCGQSSISSHSYSSMSEL
QQLHTTNIAGKKDSTTAGEDSAVVALKRIRSEAELKSLNQDKAKLLPRSLSQETLGEHPKNQYQRPLLKTSSKVD
AHRVKVTYGDEKTRFRMPKNWGYEDLLQEIGGRFNVSDMKKFDVKYLDDDCEWVLLTCDADLEECIDVCQSS
ESGTIKLSLQTSTHSMRSSLEFR
>Phaseolus_vulgaris_Phvul.005G155100.1.p
MEYPFSSKGREIGDWQSSGTQLVGSTSLDARMSNSISEDMSNSFSELMSFDTYAGLCNSPSISDQILANELPSFA
SLSYSLPDGFNIVQQNNGQCYMSEVGRNNNDMESSPIYGEKVACQQMDTLLGFLSDANEANNLNSKLKVNG
SPQHLNNSVAGNYIMSRPPALSLDERMLRALSFFKESAGGGILAQVWVPIKDGDQLILSTSEQPYLLDQMLAGY
REVSRTFTFSAEGKSDCFLGLPGRVFTSKVHEWTSNVGYYSMNEYLRFEHAVNHHVRGSIAFPIFDVHSELSCCA
VLEIVTTKEKPDFSREFEIVCRALQLVNLRTVKPLRCLPQCLSNNKKATLTEIVDVLRSVCHAHRLPLALTWLPCCY
TEGSRGEATRIIKGGHSTNSGKSILCIEESACYITDRALEGFVRACTEHPLEEGKGIAGKALRSNHPFFCPDVKTYD
VSEYPLVHHARKYNLNAAVAIRLRSTYTNNDDYILEFFLPVNMKGGSEQQLLLDNLSGTMQRICSSLRTVSDIELS
RIESSQEGFGKKNAPCFSPLSSRNSEVPLENGDCHSVPMMSLKATNARDNEIEPSTHQGRNGTKRQVQKNRST
SEKSVSLSVLQQYFSGSLKDAAKNIGVCPTTLKRICRQHGISRWPSRKINKVNRSLKKIQTVLDSVQGVEGGLKFD
PSMGAFVAGGSTIQETDAHKSLLFPEKSIVKDATPITQKSVSVPPAPCSEVENFAFKLDEKLKKTNAFSVGCSEDS
KSMAIDDGSCKMERLCIKVQDCPEQACLGSVFPKEQDKWILNKSGLGVENFKSSIRGQSSNSLFGNQMHIGVD
GDAGVIEPNHPSSSSLTDSSNGSGSMMHSISSGSQSFKNQNQSNVKSTIVDSGSKLIVKAIYREDTIRFKFDRSA
GCFSLYEEVAARFKLQTGSFQLKYLDDEEEWVMLVNDADLQECLEILDDIGTCNVRFLVRDLPLILSSSGSSNCYL
GGSS
>Phaseolus_vulgaris_Phvul.011G052100.1.p
MEDHFCPKGNETGYCTSPGAQLEESTSVDGGMKKSASEDMFSNFSELMNFDTYAGWNNSSCMTYQSLANVF
SSFSSASYPPSEGLNLVEHGNGPCFMTEVSEIHSGMESSPSCEERGIFQQMDIQLGFLDEANDSNSFDSKQNRN
GTYQQLNTSDMCNYLISRSSGRPLDDRILRALSFFMESVDGEMLAQVWVPIKHGDEFILSTSEQPYLLDPKLAG
YREVSRAFTFSAEGKKGSCPGLPARVFISHVPEWTSNVGYYNKTEYLRLDHALNHEIRGSIALPISDLHSLVPCAVL
ELVTTKEKPNFDRELEIVTHALQLVNLKTITPPRLLPQSLSSNKRAALTEILDVLRAVCHAHRLPLALTWIPCSYSEG
LGDESERIQIKEGHTSSNEKCVLCIEESACYINDGALGGFVHACVEHHLEEGQGIAGKALQSNHPFFYTDVKTYDI
GEYPLVHHARKYNLNAAVAIRLRSTYTNDDDYILEFFLPVTMTGSSEQQLLLDNLSGTMRRICRSLRTVSDAELTG
IEGSVAEFPKEKATYFFPMSKRNSQIAFINDDHDSVQKMSLKASNNGIEAVHSQVVNGSRKQIEKKRSTVEKNV
SLSVLQQYFSGSLKDAAKSIGVCPTTLKRICRQHGISRWPSRKINKVNRSLKKIQTVLDSVQGVEGGLKFDPYTG
GLIAGGSIMQEIDTHKYILFPEKSAVKDPKHATQKPVSVVPAPGSTSENSTIKLDNDDGVCLVGNKLVHSRSVLIS
DTSEGGLKKDNASSDDCCEDSKSVAMNDGSSQKGSKRAKNQDCPDQTCSISLVTDEVEVGVDGGEGIDEHNH
LNSSSTTHSSNGSGSMMHGSSSRSQSFENQKHSKVKSTCVHSGSKMIVKASYRGDTIRFKFDPSAGCFQLYDE
VATRFKLQNGSFQLKYLDDEEEWVMLVNDSDLQECIEILDDIGTRCVKFLVRDVPCVLSSRGSNNCFLSDSS
>Phaseolus_vulgaris_Phvul.008G291800.1.p
MEYGGGFGSLWECGSAWEEDSIEEVLVEGCWVEASGVGSCEGEYMGSSEAEMEMEVMRKRWWIGPEANS
SVKERLVVAVGYIRECTKNSNVVIQVWVPLRSGLVGGPGNNLRRNHPDEWVSVEMNMNMNMNMNSARD
VSLRFYRSHEYPRVPYYVDVPGSLALPVLERGTHTCLGVILILMPPCDTINYCPQVLNNSLLCNSSFQVFEEVYEA
AVNEMAEVMKCVCRAQNVALALTWAPCCIQQGGKCGYGHSTETHFSTVERACFVGEAQALPFKEACSQHHLL

RGQGIVGTAFTTSKPCFAVDIAAFSRAQYPLAHHANIFGLHAAVAIPLRTLYADFVVEFFLPKDCHDREHQNHFL
NSISLLLQQSCRNLHVVMGDEFTLPPPPPPPPPPPQQYLSNKEEMVVSEKLESQWQAEACGSWIAHMMEAQ
AQEQKGKGVCVSLEYLEEAKEEFKVTGKCRWESGGGGAYEAADLHEQQVFGDESQTQTQSFGGRRGRKSGEK
RRTKAEKTISLPVLRQYFAGSLKDAAKSIGVCPTTLKRICRQHGITRWPSRKIKKVGHSLKKLQLVIDSVQGAEGAI
KIGSFYNSFPELSSATSHSSIPNNSSSFYGHSPPSSSSPSTAPPQHQHFSQTSCTAAPLRVKASFGDEKIRFSLQPK
WGFRELEVEIGRRFNVKDLGKVVVKYLDDEGEWVVLACDGDLEECKDLHTTYESRTIRLALFQPSSSP
>Phaseolus_vulgaris_Phvul.004G114100.1.p
MSESKEENPDLLPKSKQQEEHGFAMDFDIGFESLWSLDHISLVSNPMSPFLLSTISEQPFSPIWAFDVEDERQIRI
ATAGNTKAATENPVESYENKKTVSPPFVAVPPSENPDGYCLIKERMTQALRHFKELTEQNVLAQVWAPVRNGN
RYALTTSGQPFVLDPHSNGLYQYRTASLMYMFSVDGENDEILGLPGRVFQHRIPEWTPNVQYYSSKEYQRLNH
AQHYNVRGTLALPVFEPAGQSCVAVLELIMTSPKINYAPEVDKICKALEAVNLRSSEILEHPYNQICNEDRQYALA
EILEILTVVCETHSLPLAQTWVPCEHRSVLAHGGGHKKSCSSFDGCCMGQVCMSITDVAFYVIEAHTWGFHEA
CVEHHLQQGQGVAGRAFLSHNMCFCGNITQFCKTEYPLVHYALMFGLTSSFAVCLQSSHTGNDEYVLEFFLPPR
ITDFNEQKRLLGSILATMKPHFQSLKIAAGVELEQNCSIEIIEARKEGVQLRFESIPITQSSKSPPRHASPNMGERLP
PAPSEQQIMVDFDDINDGKNLGDTAGGHIDQNTSLEARTKKKPSERKRGKAEKSISLDVLQHYFTGSLKDAAKS
LGVCPTTMKRICRQHGISRWPSRKIKKVNRSLSKLKCVIESVHGAEGAFGLNSLSTGSFPSTSNKFNRQTSLTIRPS
EPKINENDFDASRASESNRQAGVEDHFLSTRTQNPEKVINEKVVAIQETGTKGTTRFRTGSGSSEESANPSPRGS
CQGSPPNEMSPPKDIFVTGISEQCHVLRGSLGSTLYSTSTPNRATAYPMPNNFVEITEPQEPFGGQLLEGVGSSK
DLRNLCPSADAVLEDQVHEHWRMNHQCSELTPVQHMDTLNNNNNNMTPFAVRKEVKSVTIKATYREDIIRFK
ISLNCAIVELKEEIDKRLKLEEGTFDIKYLDDDHEWVLIACDADLQECMDISRSSGSNMIRLVVHDILPILGSSCESS
GDWKGC
>Phaseolus_vulgaris_Phvul.007G071900.1.p
MSESEEENTDYVTRSKPAEEAGCTMDFDLDLETSWPLDHMAFVSNPMSPFLFSSSTSDQPYSPLWAFSDGEDL
KLPASAFSDCHKIFPCDSNSIAEKPEENDDNKKPLPPLAPMPPVENVGGYCVIKERMTQALRYFKELTELNVLAQ
VWAPVRNGNRYVLTTSGQPFVLDPHSNGLHQYRTVSLMYMFDVDGENDGSLGLPGRVFQQKLPEWTPNVL
YYSSKEYPRRDHAQHYNVRGSLALPVFEPALQSCVGVLELIMTSQKINYAPEVDKICKALETVNLRSSEILDHPYT
QICNEGRQNALSEILEILTVVCETHNLPLAQTWIPCKHRIVLAQGGGVKKSCSSFDGSCMGKVCMSTTDIAFYII
DAHLWGFREACLEHHLQQSQGVAGRAFLSQSMCFCSNITQFCKTDYPLVHYALMFGLTSCFAICLRSSHTGTDD
YVLEFFLPPRVTDFHEQKALLASILATMKQHFQSLKVASGVELEDGSIEIIEATIERIHTRHESIPIAPSFRSPPRPDTS
PNMEEEVPRDPSEQQHILMYCNGTNHGANLRDKAGGNIDHMTTLETKNSKKPLERKRGKTEKSISLEVLQRYF
AGSLKDAAKSLGVCPTTMKRICRQHGISRWPSRKINKVNRSLSKLKRVIESVQGAEGAFGLNSLSTSPLPIAVGSL
PEPSTPNKFSQQVSMGIRPSEPQMKVNELNASKELETNRQAGMEDQLIGGRIRNFERVSNDKGGSTQEVGRE
PKRTRTGSGSSEDSTNPTSHSSWHDSPPNESSPVKDIFITSNHDQCAVLRRSSPGSTLQPATDTPNHPTSYPMPE
FVSAELQEPFGGMLLEDAGSSKDLRNLCPSVAEAILEDLVPEACGTNPPALDLSPKQSMGTPNKVVTPFAATKE
MKTVTIKATYREDIIRFRVSLACGIVELKEEVAKRLKLEVGTFDIKYLDDDHEWVLIACDADLQECMDVSRSSGSN
IIRVLVHDITSNLGSSCESSGE
>Phaseolus_vulgaris_Phvul.002G116000.1.p
MDFSQTNKTTLLTNLLVFNNTVQPELMRSLHVYRQGDGEERTVEREFVFSDCGSYVEMQATPVLRLVKSHVSE
VCEGYKNGVWLCILAFHADNTPQFCRIPPLLLASRNPKLQMIPNLLRDLHMIYKLDRKEEDRHTTLGSTGEECQ
GNSNNFQPSEKVFPVLDQDLNYLPYEEDESELLDNETDVESSPGLPGKKKRAPSDLVAKISLSDLVQYFGMPIVE
ASRNLKVGLTVLKRKCREFGIPRWPHRKIKSLDSLIQDLQEEAQNQELENMEAAMAVAKRQKMLESEKENIEKK
PFMDIQSETKRFRQDVFKRRHRARAVGKQNPTVSNT
>Trema_tomentosa_TorRG33x02_007750.1
MEHPFSSKEKENEYWSSSRAQVENLASFDGVPRGMLSEDVFNSFSELLTFDAYAGWSNSPAVTDQVSATYGLS

SLPSTTYVPMDGSNFVEHNVGALPVTEVGGNFNLPRSSFNYGDKIVFQPVDTQFEVSAQSNNANNSIAKHNN
GSFQENSEMGLVDSTICRPTRYSLDEKMLRALSFVKESSGGGILAQVWVPVKRGDQLFLSTSEQPYLLDHMLA
GYREVSRMYTFATEGKPGCFPGLPGRVFISKVPEWTSDVGYYKKNEYVRVEHAYNHQVRGSLALPVFEPDPGV
SCCAVLEIVTTKEKSNFDTEMEIVFHALQAVNLRTNPPPRLVPQCLSKNQKDALAEIIDVLRAVCHAHRLPLALT
WIPCCYNEGAASELAGLHVREGKTSSSEKYILCVEKSACYVNDSLMQGFAHACVEHHLEEGQGLAGKALQSNL
PFFFPDVKTYDISEYPLVHHARKFGLNAAVAIRLRSTYTGDCDYILEFFLPVNMKGASEQQLLLNNLSGTMQRICK
CLRTVSDAEVGAISSDAFQEGSVPNFMSLPGESSQMVLSDSDLNSTDELPLKVSNRRNEGIEADGVHEQAIRGS
RRQTEKKRNTAEKNVSLNVLQQYFSGSLKDAAKSIGVCPTTLKRICRQHGISRWPSRKINKVNRSLRKIQTVLDS
VQGVEGGLKFDPTTGGLVAAGSIAQELDTKKSLFFIEKTSSLQNSDPLSAVKLEEDECCTGGVDPNSGEIRKPNIV
ASTNSTKDSKIAIEAGSRLPICDARSGAFSDKACLGSYHSKEGKTSSQSKCSSKFENSKYYHVSQSSGSLGVGDEM
DTAGDGDDELIEHNLPASSSMTDSSNGCGSMLHVSSSSSLSQSFEERKHPKGKMSSGDNGSKIIVKATYREDTV
RFKFDTSTGCSQLYEEVAKRFKLQTGTFQLKYLDDEEEWVMLVSDMDLQECLEILDDIGTQSVKFQVRDMPCA
VGSSGSSNCFLAGGS
>Trema_tomentosa_TorRG33x02_035230.1
MEDGVLSPATILGAPADYPMDLDFMDELFLEGCWLETRDGSEFLNQNPPSSSPLFDPLFWPTLEPNGESNANP
SPKSNQEERHRSLFIESQGKSPLHTLPPPWATTDVVKYSGVSEAHITEGSSELSRRWWIGPKANPGPSSSVMER
LWRALMYIKDVIRDKDILVQIWVPVHEEGRRVLTTRDLPFALDDNSSKLARYRDISVKYQFSAEEDSKELVLGLPG
RVFSGKVPEWTPDVRFFRNDEYPRLIHAQQNDVRGTLALPIFELDSRTCLGVVEIVMTTQKIKYRPELESVCKAL
EAVDLKSSEVLSTQNVFNKYYQAAIPEIQQVLRSACDTHRLPLAQTWVPCIYQGKEGCRHSDENYGQCVSTVD
HACYALDPQVQSFHEACSEHHLFRGQGVVGLAFMTNQPCFSADITSYTKTEYPLSHHARMFRLQAAVAIRLRSI
HTGAADFVLEFFLPVDCKDPEEQKKMLTSLSLIIQQCCQSLRVITDKELEEESGSRVDEVVVPSNPRPARNTCFTE
APQNDTDLSLFPEEKKPREISDGRLSKLSDNQRDSILKPSVDCVEECSTVGEGSFSSVGVGKTGERRRTKAEKTIT
LQVLRQYFAGSLKDAAKSIGVCSTTLKRICRQHGIKRWPSRKIKKVGHSLQKLQLVIDSVQGASGAFQIDSFYTNF
PELASPNVSGTSPFSTSKPNDHPMPSNMQPGDGGIFSVQAATAAATSKSSSSCSQSSSSSHCCSSRSQLHPQT
WNNVTSSDDLIAGESSGGGDDVVLKRVRSEAGLNACSEDGRKLLPRSQSHKSLMEHHKTEKWFPPSSAKNNN
NGARIPQQQGEFQKVKVTYGEDRTRFRMQNNWGFIDLQQEVGRRFGIQDMVKFTLKYLDDDSEWVLLTCD
ADLEECFEVYRSSQNATIKLSLQPSRHLYRGCLRGNDPL
>Trema_tomentosa_TorRG33x02_082070.1
MADPGAIMSYYDLYDGPFSENLSNILTDYENPSLLDLPEPDHIGGQGPSNTSDNHNPDHVTLEQNSATNWDLP
IDFNVDQCLGSGPNNQPGPSRIRDHQETNNNDPDDQVIEENLEFPQVNNSNNIGGNSRTLPTWPSPPVPFNC
TCCQVLREIIHVNEFNNYMKLEIHGRLGMICHAIWESRNTTNNNVTTSTSSGNDPRFEMFDFCRQSIEDVKRFL
VEYCSRRKVEGYVMLHDPLSIFYEALCVGLEWDELSNNDFDFVPTSPPHNSGGAEENYTEQQGQEEQQVTHQ
QEQTVETRLETTQNDVERNSRISLAEQRQRTGKLTLMDLREYLHLTIEDAAKAMNVCPTVLKKICRRHNLPRWP
YRKIRSIKRQISSLRPNLESNDAAVREQAQAEIDRLRQEATNICAGVDVVGL
>Trema_tomentosa_TorRG33x02_105690.1
MVAKDENPFDPFSTQFLSSPPNFSGDGYQFSTNFEQNWQNELPIQESILLDAVPLMEYYPTDPLYSTVELEPLPS
IIQDDILSNNGNGLEVWDDIGVLGFEANYNQKKPSLLLCNDPSNINGDDQKGKEKMKREKKIVKRRLSCRISSEE
KSSTSASKTLSRQVLSQYYYMPITQAAKELNVGLTLLKKRCRELGIRRWPHRKLMSLQTLIKNVQDLGKEEGDHQ
ENDSKLKNAIELLEREKKLVEEVPDIQLDDTTKRLRQACFKANYKKRKLRIMDSDHYHHSGLNTKYLEEEDEEIKS
LLSDSFSSTNHLF
>Trema_tomentosa_TorRG33x02_114670.1
MSESEEETTPSFPPKSSSSSKEVVPDVREGLMDFDLDLDNPWPMMDQIAFVSNPITGNSNSVAERPAENEERK
IVPSPFLGLVPVDNPNGYYLIKERMTQALRYLKDSTDQHVLAQIWAPVKNGCRYVLTTSGQPFVLDPDSNGLH
QYRMASLMYMFSVDGETNEVLGLPGRVFRQKLPEWTPNVQYYSIREYPRLDYAQHYNVRGSLALPVFEPSGQ

SCVGAVNLRSADILDHTSTQICNEGRQNALTEILEILTAACETYKLPMAQTWVPCMHRNVLAYGGGMKKNCTSI
DGSCMGQVCMSTTELAFYIIDAHMWGFREACLEHHLQKGQGVAGRAFYSRNLCFCGDITQFCKTEYPLVHYA
RMFGLTSCFAVCLQSSHTGNDNYVLEFFLPPSITNASEQSALLGSLLATMKKHFQSLKVASGIGLEEEGIVELVKVS
AIGGLESSLECIQIPKSAESPPGPIALPNGGDMVQQDLLTQQLIVDLDTANVGQDAAHDGGSVNHVSVSENKET
KKTSERKRGKTEKSISLEVLQQYFAGSLKDAAKSLGVCPTTMKRICRQHGISRWPSRKINKVNRSLSKLKRVIESV
QGAEGAFGLTPLATSPLPVAVASISRPSTSNGTNHQHSPSNIPSEPPMERKDSPTTSSPRREGQVETEDQLQGG
GVLSQKEFIHENGGFFSPEINRGSNHSKSGSGSGEASVGTPTSHGSCQGSPANGSVIVKDPFVYSIHEQCIKVEG
SPESAPQPTDELHFSVPDALVTAECQEPFGGMLIEDAGSSKDLRNLCPAADAVLDEPVPEYCWINSTCSDLAPK
QTSGVITQTQPNVIVRLRQEMKNVTIKATYREDIIRFRISLSSNIVELKDEVAKRLKLELGTFDIKYMDDDQEWVLI
ACDADLQECMDVCRSSGSNMIRLLIHDIMPNLGSSCESTEE
>Trema_tomentosa_TorRG33x02_215090.1
MDSSKTHLLLSLLIFKNTINRELIRSLHVYRLINGKEKEVEREFVFSANRAYVEMEADPLLILQKFEVSEVFEGFVN
GSWLCIFAFYAHRPPPPLLTCIPSLLTLSRNPKLRAIPSLANDLETIFHMICVTDDKEPSRVLSERTCQRNNQNHRQ
LRRNVPVLDQDLNCLPYPVTPSELSDDQQIEQSSSGTMEKKKKRATSKDIARIALSDLAKYFDLPIVEASRNLKVG
LTVLKKKCREFGIPRWPHRKIKSLDSLIRDLQEEREWQQQDNKAAAMAVAKRQRMLENERESIEKKPFWEMK
TETKRFRQDVFKRKHRARVLGTQSLFLPDN
>Trema_tomentosa_TorRG33x02_229930.1
MEYSAQLIVPKIENPLGFDWLFHENPSENLLPDFPPVQTFFEPNYPLPFSYLYDQLPSIDFQNFEDIDGGFFFDNA
FSYCEDPMVDEKPKTNLVTSFSHNNKNLTSDQIGFEVSRLIESEIFGSSEKNEEETRSCGRRKKKVVELELDDIQKY
FDVPIKEAAKGLRVGVTRLKKRCRELNIMRWPHRKLKSLKYLLNNVKKMGLNSNEIMMLEEQQRLLEVAPDM
DLTERAKKLRQACFKANYKSKRSLEAARA
>Trema_tomentosa_TorRG33x02_237950.1
MPITLQAVDLRSSQNFSPPAVKVCDELYQAALSEIVEVLVSVCKIHRLPLALTWAPCHQQGKGGCRHSDENYAR
CVSTVDSACLVSDLEILGFHEACSEHHLFRGQGIVGIAFTTNKPCFANNITAFSKTEYPLSHHARMFGLRAAVAIPL
RIVYTGSSDFVLEFFLPKDCQDPEEQKQMLNSLSIVIQQACRSLHVVVEKELEEEQFMLPVGDPTIVASSEGGLK
RGTTTTTATTNTTTCPVGDQSSGKESSWVSHMMEAQQQGRGVSVSLDYLTEEPKEEFRVTTHWDNLNHGDL
SNGQVFSDFGQLQQCSGSKGSMEGGIDSYSFGGRRSSGARKAGEKRRTKTEKTISLPVLRQYFAGSLKDAAKSI
GVCPTTLKRICRQHGITRWPSRKIKKVGHSLRKLQLVIDSVQGAEGAIQIGSFCSSFPQLNSQGNSNDSFSSISPS
DHHHHHHHHHLNNQSNSQPESGFFIHGSAMKSSPSMSSQASGLQQSTTFSGFNGDPLITEDHSVILNRACSS
DGELFASNQDEANLLSRSQSYKALSDQNRILDQTLPPLPDNNERINSRDGFAFRVKANFGEKKIRFSLPPNCSFR
DLQLEIMRRFALDDITMIDIKYLDDDREWVLLTCNADLQECLEIHR
>Trifolium_pratense_Tp57577_TGAC_v2_mRNA3630
MSQQNQHGIFHNTNSDTFNSNSNAMLSQQNHHQQGQGGNNDPFHYNLISPEDHHQQVQGDNNNNDS
MFITNEMSQQNHHGGNNQSFSDPMVQEQYAQNSNNNSNNFSFGGNNFEVGGTSQMHEDQQLIQPSIQTP
DLPNQNEVLAIDQWPPTQVPFFCTCCQVLREIIHANGFQFEKLEIHGRIGLITHAIHHKLPTNGNTPNYQMIDFC
KSSLDEIKKYLAKYCVDRNTSGYFILQDPMSAFYETLCTGLDWIDDINMEGPMDNNQNNSDDMGENATSDKK
DLSAQRKKSASLTLSDLRDYFHLPIEEASDHPNVKLCPTVLKKTCRKAGLQRWPHRKVKSLLKQIAIMGAQLDGQ
DSATRARTEHEINRLKQEMIAHCGGYIPTAMHNIAAFLPQQQQ
>Trifolium_pratense_Tp57577_TGAC_v2_mRNA39375
MGDGAAVTSSATMMEGPPQDENSMDFDYMSELLLDGCWLEASADGSEFLLQSSPFSNPIFDPSFSWPSLENT
EPPRNEDGHVGIEQESQIIVNNAGSSDRQQCEFETRSGEGVSRLWIPPTPNPGPGGSFITEKLIRALKWIKDFNR
NKDMLIQIWVPVNRGDDRPILRANDLPFSLESRSLNLAKYREISVRYQFSVEEEDSKELVAGLPGRVYRDKIPEW
TPDVRFFRSEEYPRVDHAQECDIHGTLAVPVFEQGSRTCLGVIEVVMTTQQSNYGRELETVCKALEVVDLRSSG
HSSLQNVKQACDKSYETALPEIQEVLRSACEMHKLPLAQTWISCIQQGKDGCRHSDDNYANCISPVEYACYVGD

SSVRFFHEACMEHHLLKGQGVAGGAFMTNQPCFSADITSLSKTDYPLSHHARLFGLRAAVAIRLRSIYSTSDDYV
LEFFLPVDCNDSEEQKNMLTSLSMIIQRVCRSLRVITDKELDKDNLPSNEVMAVTDGRFATNAIWSESQHRRM
AASLGGEEKLSETMSRKFSEQRRQQETLTLKGDLDCGRASSPSVEGTFLSSLGINRTGEKRRAKAEKTITLQVLRQ
YFAGSLKDAAKNIGVCTTTLKRICRQHGIKRWPSRKIKKVGHSLQKLQLVIDSVQGASGSFQIDSFYSKFSDLSSP
RTSLISTLNQIDNQVSLSTLPDPDSLSPEGASKSPSSSCSQSSFSSHSCSSMSEQQHRKGNVAIGNKDPSVGEDSV
DVVLKRIRSEAELKSLIQDNKTKLMPRSQSQETLGENPKTEHHKSFLKSAPKASQKEDSHRVKVTFGDEKTRFRL
PKNWGYEDLVQEIARRFNVSEMSKFDIKYLDDDYEWVLLTCDADLEECIDVCQSSETSTIKLCLQPSSNFIRSSLEF
R
>Trifolium_pratense_Tp57577_TGAC_v2_mRNA6838
MEYPFYPKGRGIGYWQSPGTQLEESRSLDGGISNLGSEDMPSSFSELMNFDTYAGLCSGPSMTDQIMANELPS
LASVLYQSPDEFNLVEQNTGQFYMTGVSGNYNNSESSPIFGEKIVRQQMDSLLDLLDNNNDANNLSSKQKIND
SSQHVNTFDMDNCIISKPPDLSLDERMLKALSFFKESAGGGILAQVWVPIKHGGQVYLSTSEQPYLLDQMLAG
YREVSRTFTFSTEGKPGFLPGLPGRVFISKVPEWTSNVGYYSPSEYLRVEHARNHDVRGSIAFPIFDLHSGLPCCA
VLELVTTKEKLDFDRELEIICRSLQLVNLRTTMPFRLLPECLTNNKRAALTEIVDVLRSVCHAHRLPLALTWIPCFYN
EGTRDETTRIQIKEGNSSSREKNILCIEESACYITDRAMEGFVHACIEHPLEEGKGVAGKALQSNHPFFYSDVKTY
DISEYPLVHHARKFNLNAAVAIRLRSTYTNNDDYILEFFLPINMKGSSEQQLLLDNLSGTMQKICKSLRTVSGAEL
SEMESSQEGFEKNSVPSFPPSSKRKSPTPFINESHGSVKKLSSKASNLRNNGNEPSPNQERNAPKRRAEKYRSSS
EKNVSLSVLQQYFSGSLKDAAKSIGVCPTTLKRICRHHGISRWPSRKINKVNRSLKKIQTVLDSVEGVEGGLKFDP
SVGAFVAKGTAIQEIDENRSLPFPEKSTTEDSEPFSQDAASVPLAHCSDGENSANKLDEKLKETNASLIDCSEDSK
SFAMDDCPEQACFGSVLGKGDQWVLNKGGLREEEKCKHNTIGKRTSSFVVYEMDTGVDGDDEAVENNNPAS
SSLTGSSNSSGSIVHDSSSGYQNLKNQKQSKSKSIIVDSGSKIVVKATYGEDTIRFKFDPATGCLKLYEEVASRFKLR
NGTFQLKYLDDEKEWVMLVNDSDLQECLEILNDMGTRNARFLVRDTPCTFGSSGSSVCYLGGSS
>Trifolium_pratense_Tp57577_TGAC_v2_mRNA19272
MEYGGGLVGDGGVFGNMVGGGGEAADMIEELLVDGCWIEASENNMMAMQSQTQTHYMSTNNNNNNNI
PIGMGEGDHFNNHHHNQIECNVPTDHDDDQESGFVVGKRWWIGPRANPGPSTSVKERLVVAVGYLKEYTK
NSSNNVLIQIWVPALRRRSPLLIHNHYLHESSSSAPVSVNPNTNVHVRFFRSHEYPRHQQQQYGSLFALPVFER
GSGTCLGVIEFVITNQTVVNYRPQLDHLSNALEAVDFRSSHNMNIPSAVKVFEELYEAAVNEIVEVLASVCKTHN
LPLALTWAPCIQQQGGGVRGKGTAGAAGCGVSVAVPTDQMNNNQMMMNCVSTVDSACYVGDMEVLGFQ
EVCSEYHLFNGQGIVGTAFTTTKPCFAIDITAFSKAEYPLAHHANMFGLHAAVAIPLRSVYTGSAADFVLEFFLPK
DCRDPEQQKHMLNSLSLVVQQACRSLHLHVVIDDTDNANHHDHDQDQFTFPTTTTTNNYMPSSASDAATAS
LSQVDATSGCSTKDTSSSCSWIAHMMEAQHKGKGVSVSLEYLQEPKEEFKVTTCNWDREREGNSVFSEFGQVL
QHDQSSNSRASVEAGEESGGCGVGGGRRSSSSSSGRKSGDKRRTKAEKTISLPVLRQYFAGSLKDAAKSIGVCP
TTLKRICRQHGITRWPSRKIKKVGHSLKKLQLVIDSVQGAEGAIQIGSFYASFPELSTATANGGGGGGGDHNNV
NNSFYNSHGDGVVNGLKSPPSACSQTHAAGNKLITANIINGDHHHHHHVMMTENPAAPLAADSLMMHGSN
INIQNYQQLQEDQDTKQLLLHFNNNNQTLPPRPTAGAWNNNNNSSSSGLLERGAFRVKATFADEKIRFSLQA
MWGFRDLQLEIARRFNLNDMNNLVLKYLDDEGEWVVLSCDADLEECKDLHTSSHTRTIRLSLFQASPLNIPNN
FVRNSSSSPSS
>Trifolium_pratense_Tp57577_TGAC_v2_mRNA9575
MFDILFDSEKEHLCCTDIEIKKEVVEFKPEVEERKKITISGEDTKSLSRDTISQYFYMPISQAAKELNIGLTLLKKRCR
ELGIGRWPHRKLTSLQTLINNVQELREEEEPLSDEKLMNVIEMLEKEKKLVEEMPDMELDYRTKRLRQACFKAN
YKKRKLMEQVF
>Trifolium_pratense_Tp57577_TGAC_v2_mRNA19433
MDFFAKPETSYWFCNDQSQQNDTDKIMDMTPYDDFVNIDWANHFSYQSNKLFLNEFHDLENFNFDFNLPNL
DENLDVEQKPTNVVFPELVHNTEKVESVCESAFSETSEKVGKGLMSFVKKEEEWENQDSPFGGNDFSFAESGF

VETFAKHGNEFLNFVKNENEEFSIPLTLPSSNVIMKKKSSLLQFDEIKEHFDVPITMAAKKMNIGVTLLKRRCREL
NINRWPHRKLKSLMLLIDNLKEMGLEDEVAMLEKEKKMLEEIPGMELNAEIKKLRQSFFKANYKKRRSNTFHP
>Trifolium_pratense_Tp57577_TGAC_v2_mRNA8649
MEEDSLKIALEVLEELKMGKNEIGRGDSKVVVANSSMVEVMGGVLNDGQSIKKKKNNIEVKDIYPLKYPICPKC
KGEFFTFSAVIEHLLKNPNCASCGTSNPHQLVDLNKGADEVQQMEIEGSSNAAAAPKPAECFVGKKKRATRDH
VAKITVSDLVKYFDMPLLEASKKLNVGTTVLKRRCRELGLPRWPHRKIKSLDTLIHDLHQEEAAHKEMKNKAAA
MAVRRKQKMVESEKERIEKKPFIAIKSETRRLRQYVFKRRYHARVMEKQN
>Trifolium_pratense_Tp57577_TGAC_v2_mRNA8651
MDSSLTTLLLFNNTIQPELMRSVHVYRGECDGEEREVEREVEREFVFSKNGSYVEMQATPILSLLKSFVSKEIFEG
YTNGVWLCIFAFHAYNTPPFSHIPTLLLVTRNPKLQAIPNLLNDLHMIYKLVPKEEPKDTPESSTAEESKGNNNNF
QPPKKLFPVLNQDLNCLPYEDDESELLDDKSGLLDNKSELLGDETDFESSSGSVGKKKRAASDIVAKITLPDLVKY
FDMPIVEASKSLNVGLTVLKKKCREFGIPRWPHRKIKSLDSLIHDLQEEAKHQDMEDKAAAMAVLRRQKMLEC
EKENIEKMPFMDIRSETKRFRQEVFKRRHRSRVMEKQNSTVASN
>Trifolium_pratense_Tp57577_TGAC_v2_mRNA27901
MPESEEENQDFPPKTKTSLEEHVCAMDFDLDLETSWPLDHMSFISNPMSPFLFSTISDQPSSPLWDFTDGEDE
NHNKLAASAFSDCHKIFSCDSNSITEKPVEKDDNKTLLPPLLPPIEHLDRYCGIKEKMTQALRYFKELTEMNVLAQ
VWAPVRNGNRFVLTTSGQPFMLDPHSNGLNQYRTVSLMYKFSVDGENDGTLGLPGRVFQQKLPEWSPNVLY
YSSKEYPRRDHAQHYNVRGTLALPVFEPSLPSCIGVLELIMTSPKINYAPEVEKICKALEAVNLRSSEILDHPFTQIC
NEGRQNALSEILEILTVVCETHNLPLAQTWVPCRHRSVLAHGGGFKKSCSSFDGSCMGQVCMSTTEVASYIIDA
HLWGFREACVEHHLQHGQGVAGRAFLSQNMSFCSNITQFCKTDYPLVHYALMFGLIGCFAICLRSFYTGDDDY
VLEFFLPPGITEFHEQKTLLGSILATLKEHFQSLKIAAGVELEENGPIEIIEATDEGIRLRTESIPIAQSIKSPFIPDASPN
MEEVPQDDPSGIHGEDIGGSINPMSPLGNKNIKKPSERKRGKTEKSISLEVLQRYFAGSLKDAAKSLGVCPTTMK
RICRQHGISRWPSRKINKVNRSLSKLKRVIESVQGAEGAFDLNSLSNNQLPIVSSFPEPSTPNKSNQQGSLNIKPS
EPQMKENEFDASKVSETNIQAVMEDQLLGGRKHSLDKEVINDKGVSIREIGNDRKRTRTRSGSSEDSTNPASH
GSCHGSPPNEISTVKDLFIPSNNEQCIVLRGSPESRVQPTNAFNSPTAHCLPDNVLAELQEPFGGMLIEDAGSSK
DLRNLCPSVAEAILEDMAPEACGTNLPGSYMAPPKQCMDTINNNNSVMPFAVRKEMKTVTIKATYREDIIRFR
VSLNCGIVELKEEVSKRLKLEVGTFDIKYMDDDNEWVLIACDADLQECMFVSRSSGGSNIIRVLVHDITSNLGSS
CESSGE
>Trifolium_pratense_Tp57577_TGAC_v2_mRNA35367
MNMEDQFSSKDNGISFWTPLDNGMKSSITSDDMFNNISELMNFDNYAGWCNGSSSLIDQTLTSDLSSFAYSP
HDGLSLVEQINGPFFMTDIGGNYSTTSYEEKVLLQQMETQFAFVDNANETNNNNLDSQFDMCNYVMSKSPS
WSLDERMMSALSFFKESAGGGILAQFWVPIKYGDEIVLTTSNQPYLLDQKLAGYREVSRSFTFSAEIKMGACCP
GLPGRVFISHVPEWTSNVGYYHKSEYLRLDHAISHEVRGSIALPISDMNSEVSCSAVLELVTTKEKPNFDKELEYV
SHALQRMNLRTITPPRLLPQCVSNNKRAALAEIVDVLRAVCHAHRLPLALTWIPCYYSEGKGEESERLRIKEGRTT
NSDEKCVLCIEESACYINDKMVEGFVHACSEHHLEEGQGISGKALQSNHPFFYTDVKAYDISEYPLVHHARKYNL
NAAVATRLRSTYTNDDDYVLEFFLPIDMIGSSEQQLLLDNLSDTMRRICKSLRTVSEGELRGIEVSQDGFPKENIS
GFFPMSKGNSQIAFSTGDPDLFQMPLMAANNGIQGSHSQVTSESRKQAEKRRNAVEKNVSLSVLQQYFSGSL
KDAAKKIGVCPTTLKRICRQHGISRWPSRKINKVNRSLKKIQTVLDSVQGVEGVLKFDPHTGGFVAGGSIIEQIGT
CNKNLMFPEKSSVENSAIKLEDDGVLISNSCEGDLKKDNNASSDDYIQDSTSWLWTKTHECPKQNSIDSVLEKE
EDQCGLNNSSLHDIASNSSFSLIELGLDEGKGLDEPNHPTSSSMTDSSNASGSMVRGSSSGSQSIENHKNSKIKS
ICVDSESKFAVKATFRGDTIRFKFDPCLGCFQLYEEVATRFKLRNGSFQLKYLDDEEEWVLLVNDSDLKECVEVLS
DSGTYCVKLLVRDIHGDGNS
>Trifolium_pratense_Tp57577_TGAC_v2_mRNA12227
MWYFENCTIADFDLYDAKNLLPMGNEVDGGFYAENNVFSISNNIVENNAKEIMKGKRCREENSARMLTRKTIS

EYFYMPISQAARELNVGLTHLKKRCRDLGIQRWPHRKLMSLQTLIKNVQEQGDESDEKIRNAVEILQKEMKKVE
EKPDLQLEENTKRLRQACFKANYKKRRLMVMRLMDHQHSSISEIESVNDHIRV
>Trifolium_subterraneum_Tsud_chr1.g44340.1.am.mkhc
MPESEEENQDFPPKTKTSLEEHVCAMDFDLDLETSWPLDHMSFISNPMSPFLFSTISDQPSSPLWTFTDGEDE
NHNKLAASAFSDCHKIFSCDSNSITEKPVEKDENKTLLPPLLPPIENLDRYCGIKEKMTQALRYFKELTEMNVLAQ
IWAPVRNGNRFVLTTSGQPFVLDPHSNGLNQYRTVSLMYKFSVDGENDGTLGLPGRVFQQKLPEWSPNVLYY
SSKEYPRRDHAQHYNVRGTLALPVFEPSLPSCIGVLELIMTSPKINYAPEVEKICKALEICNEGRQNALSEILEILTV
VCETHNLPLAQTWVPCRHRSVLAHGGGFKKSCSSFDGSCMGQVCMSTTEVASYIIDAHLWGFREACVEHHLQ
HGQGVAGRAFLSQNMSFCSNITQFCKTDYPLVHYALMFGLTGCFAICLRSLYTGDDDYVLEFFLPPGITEFHEQK
TLLGSILATMKEHFQSLNIASGVELEENGSIEIIEATDEGIRLRTEYIPIAQSIKSPLIPDASPNMEEVPQDNPSGIHG
EDVGGSIDPMSSLANKNIKKPSERKRGKTEKSISLEVLQRYFAGSLKDAAKSLGVCPTTMKRICRQHGISRWPSR
KINKVNRSLSKLKRVIESVQGAEGAFDLNSLGNNQLPIVSSFPEPSTPNKSNQQGSLSIKPSEPQMKENEFDASK
ASETNIHVVMEDQLLGGRKHSLDKEVINDKGVSIQEIGKDRKRTRTRSGSSEDSTNPASHGSCHGSPPNEMSN
VKDLFIPSNNEQCVVLRGSPESRVQPTNAFNSPTAHCLPDNVLAELQEPFGGMLIEDAGSSKDLRNLCPSVAEAI
LEDMAPEACGTNLPGSYMAPPKQCMDTINNNNSVMPFAVRKEMKTVTIKATYREDIIRFRVSLNCGIVELKEE
VSKRLKLEVGTFDIKYMDDDNEWVLIACDADLQECLYVSRSSGGSNIIRVLVHDITSNLGSSCESSGE
>Trifolium_subterraneum_Tsud_chr2.g36860.1.am.mk
MNMEDQFSSKDNGISFWTPLDNGMKSSITSDDMFNNISELMNFDNYAGQGISGKALQSNHPFFYTDVKAYD
ISEYPLVHHARKYNLNAAVATRLRSTYTNDDDYVLEFFLPINMIGSSEQQLLLDNLSDTMRRICKSLRTVSEGELR
GIEGSRDGFLKENISGFFPMSKGNSQIAFSTGDPDLFQMPLMAGSHSQVTSESRKQAEKRRNAVEKNVSLSVL
QQYFSGSLKDAAKKIGVCPTTLKRICRQHGISRWPSRKINKVNRSLKKIQTVLDSVQGVEGVLKFDPHTGGFVA
GGSIIEQIGTHNKNLMFPEKISVENSAIKLEGDGVLISNSCEGDLKKDNNASSVDYIQDSTSWLWTKTRECPKQK
SIDSVLEKEEDQCGLNNSSLHDIASNSSFSLIELGLDEGKGVDEPNHPTSSSMTDSSNASGSMVRGSSSGSQSIE
NHKNSKIKSICVDSESKFAVKATFRGDTIRFKFDPCLGCFQLYEEVATRFKLRNGSFQLKYLDDEEEWVLLVNDSD
LKECVEVLSDSGTHCVKLLVRDIHGNS
>Trifolium_subterraneum_Tsud_chr2.g58920.1.am.mkhc
MEYPFYPKGRGIGYWQSPGTQLEESRSLDGGISNLVSEDMPSSFSELMNFDTYAGLCSGPSMTDQIMANELPS
LASVLYQSPDEFNLVEQNTGQFYMTGVSGNYNNSESSPVFGEKIVRQQMDSLLGLLDNNNDANNLSSKQKFN
DSSQHVNTFDMDNCIISKPPALSLDEKMLKALSFFKESAGGGILAQVWVPIVHGGQVFLSTSEQPYLLDQMLA
GYREVSRTFTFSTEGKPGFLPGLPGRVFISKVPEWTSNVGYYSPSEYLRVEHARNHDVRGSIAFPIFDLQSGLPCC
AVLELVTTKEKLDFDRELEIICRSLQLVNLRTTMPFRLLPECLSNNKRAALTEIVDVLRSVCHAHRLPLALTWIPCFY
TEGTRDETTRIQIKEGNSSSREKNILCIEESACYITDTAMEGFVHACIENPLEEGKGVAGKALQSNHPFFYSDVKTY
DISEYPLVHHARKFNLNAAVAIRLRSTYTNNDDYILEFFLPINMKGSSEQQLLLDNLSGTMQKICKSLRTVSGAEL
SEIESSQAGFEKNSVPSFPPSSKRKSRIPFINENHGSVHKLSSKASNLRNNGNEPSSNQERNGPRRRGEKNRSSS
EKNVSLSVLQQYFSGSLKDAAKSIGVCPTTLKRICRHHGISRWPSRKINKVNRSLKKIQTVLDSVEGVEGGLKFDP
SVGAFVAKGTAIQEIDENRSLLFPEKSTTEDSEPFSQDAALVPPAHCSDSENSANKLNGKLKEINASLIDCSEDSKS
FAMDDCPEQACFGSVLGKGDEWVLNKGGLREEEKCKHNTIGHRTNSFAVYEMDTGVDGDDEAVEHNNPASS
SLTGSSNSSGSMVHDSSSGYQNRENQKQSKSKSIIVDSGSKIVVKATYGEDTIRFKFDPATGCLKLYEEVASRFKLQ
NGTFQLKYLDDEEEWVMLVNDSDLQECLEILNDMGTHNARFLVRDTPCTFGSSGSSGSYLGGSS
>Trifolium_subterraneum_Tsud_chr5.g11950.1.am.mkhc
MDSSLTTLLLFNNTIQPELMRSVHVYRGECDGEEREVEREFVFSENGSYVEMQATPILRNPKLQAIPNLLNDLL
MIYKLEPKEERRDTPESSTAEESKGNNDNVQPPRKVFPVLNQDLNCLPYEDDESELLDDKSELLDTKSELLDDET
DFESSSGSVGKKRRAASDIVAKITLPDLVKYFDMPIVEASKNLNIGLTVLKRKCREFGIPRWPHRKIKSLDSLIHDL
QEEAKHQEMEDKAAAMAMIRRQKMLECEKENIEKMPFMDIQSETKRFRQEVFKRRHRTRVMEKQNSTVAS

N
>Trifolium_subterraneum_Tsud_chr5.g12030.1.au
MEEDTLKTALEVLLELKMGKNEIGRRDPKVAVANSSMVEVMGGAVHDGQSIKKRKNNIEVKDIYPSKYPICPRC
KGEFFTYSAVVEHLLKNPNCASSGTSNPPQLDLNKGADEIEVQPMEIEGSSNAAAAAPEPAERKKKRATRDHVA
NITVSDLVKYFDMPLLEASKNLKIGTTVLKRKCRELGLPRWPHRKIKSLDSLIDNLHQEEAEHKEMENKAAAMA
VRRKQKMVESEKERIEKKPFTSIEGETKRLRQHVFKRRYHARVMEKQNSTVSSN
>Trifolium_subterraneum_Tsud_chr5.g12040.1.am.mk
MKSPNPNSSKNSIACPKETNPPFKSSKGSMEEDTLKTALEVLLELKMGKNEIGRRDPKVAVANSSMVEVMGGA
VHDGQSIKKRKNNIEVKDIYPSKYPICPRCKGEFFTYSAVVEHLLKNPNCASSGTSNPPQLDLNKGKKKRATRDH
VANITVSDLVKYFDMPLLEASKNLKIGTTVLKRKCRELGLPRWPHRKIKSLDSLIDNLHQEEAEHKEMENKAAA
MAVRRKQKMVESEKERIEKKPFTSIEGETKRLRQHVFKRRYHARVMEKQNSTVSSN
>Trifolium_subterraneum_Tsud_chr5.g43000.1.am.mk
MEYGGGLVGDGGVFGNMVGGGGEADMIEELLVDGCWIEASENNMMAMQSQTHYMTNNNIPINGMGEG
DHFNNHHHNQIECNVPTDHDDDQESGFVVGKRWWIGPRANPGPSTSVKERLVVAVGYLKEYTKNSSNNVLI
QIWVPALRRRSPLIHNHYLHESSSSAPVSVNPNTNVHVRFFRSHEYPRHQQQQYGSLFALPVFERGSGTCLGVI
EFVITNQTVVNYRPQLDHLSNALEAVDFRSSHNINIPSAVKVFEELYEAAVNEIVEVLASVCKTHNLPLALTWAPC
IQQQGGGVRGKGTAGAAGCGVSVTAPTDQMNNNNQMMMNCVSTVDSACYVGDMEVLGFQEVCSEYHLF
NGQGIVGTAFTTTKPCFAIDITAFSKAEYPLAHHANMFGLHAAVAIPLRSVYTGSAADFVLEFFLPKDCRDPEQQ
KHMLNSLSLVVQQACRSLHLHVVMDDTDNANHHDHEQDQFTFPTTTNNYMPSSASDAATASLSQVDATSGC
STKDTSSSCSWIAHMMEAQHKGKGVSVSLEYLQEPKEEFKVTTCNWDREREGNSVFSEFGQVLQHDQSSNSR
ASVEAGEESGGCGVGGGRRSSSSSSGRKSGDKRRTKAEKTISLPVLRQYFAGSLKDAAKSIGVCPTTLKRICRQH
GITRWPSRKIKKVGHSLKKLQLVIDSVQGAEGAIQIGSFYASFPELSTATANGGGGDHSNVNNSFYNSHGDGVV
TGLKSPPSSCSQTHAAGNKLITANTINGEHHHHVMMTENPTGPSAADALMMHGSNINIQNYQQLQEDQDTK
QLLLHFNNNNNQTLPPRPTGGAWNNNNSSSSGLLERGAFRVKATFADEKIRFSLQAMWGFRDLQLEIARRFN
LNDMNNLVLKYLDDEGEWVVLSCDADLEECKDLHTSSHTRTIRLSLFQASPLNLPNNFVRNSSSSPSS
>Trifolium_subterraneum_Tsud_chr6.g32900.1.am.mkhc
MQESEEENHDLAKKSKPLEENGFSMDFDIDFENLWPLDHISNTMYPFLLSTTSEQHFSPIWTFSDVEDDSIAAS
GNNTKTETDNRVENDDKKKHVTPHHVEVVPNLQTLHGYCLIKERMTQALRQFKELTEQNVLAQVWAPVRNG
NRYKLTTSGQPFVLDPHSNGLHQYRTVSLMYMFSVDGENDEILGLPGRVFQQKLPEWTPNVQYYSSKEYSRLN
HALHYNVRGTLALPVFEPPGQSCVAVLELIMTSQKINYAPEVDKVCKALEAVNLRSSEILEYPYSQICNKDRQNAL
SEILEILTVVCETHNLPLAQTWVPCRHRSVLAHGGGLKKSCSSFDGYCMGQVCMSITEVAFYVIDSHTWGFHDA
CVEHHLQQGQGVSGRAFLTQNMCFCENITQFCKTDYPLVHYALMFDLTSCFSICLQSSHTGNDEYVLEFFLPPSII
EFCEHKALLGSILAIMKQNFQSLKVASGFELEEDGSIEIVEILNGRVHSRVESVPIIQSEKLSTRHDDASPNMEEGV
LPLDPLQQKVITSFDDINDRRILDVNTGGNIDRISSLETKTKKKTSQRKRGKAEKSISLDVLQNYFSGSLKDAAKSL
GVCPTTMKRICRQHGISRWPSRKIKKVNRSLSKLKCVIESVHGVEGAFGLNSPTTSPLHIASSSFNQPSTSNKFEH
QTALNIIPSEPKMNENYLNASKSQPQEPFIGMLIEGADSCSKDLKNMCPLADTVLEDQILEASRTNPPCFDFAPL
AEKEMKIVTIKATYRDDIIRFRVSLNCGIVELREEISKRLKLEVGTFDIKYMDDDQEWILVACDADLQECMDILTSS
GSNIIRLVVHDIVSILGSSVEISE
>Trifolium_subterraneum_Tsud_chr6.g32910.1.au
MTQALRQFKELTEQNVLAQVWAPVRNGNRYKLTTSGQPFVLDPHSNGLHQYRTVSLMYMFSVDGENDEILG
LPGRVFQQKLPEWTPNVQYYSSKEYSRLNHALHYNVRGTLALPVFEPPGQSCVAVLELIMTSQKINYAPEVDKV
CKALEAVNLRSSEILEYPYSQICNKDRQNALSEILEILTVVCETHNLPLAQTWVPCRHRSVLAHGGGLKKSCSSFD
GYCMGQVCMSITEVAFYVIDSHTWGFHDACVEHHLQQGQGVSGRAFLTQNMCFCENITQFCKTDYPLVHYAL
MFDLTSCFSICLQSSHTGNDEYVLEFFLPPSIIEFCEHKALLGSILAIMKQNFQSLKVASGFELEEDGSIEIVEILNGR

VHSRVESVPIIQSEKLSTRHDDASPNMEEGVLPLDPLQQKVITSFDDINDRRILDVNTGGNIDRISSLETKTKKKTS
QRKRGKAEKSISLDVLQNYFSGSLKDAAKSLGVCPTTMKRICRQHGISRWPSRKIKKVNRSLSKLKCVIESVHGVE
GAFGLNSPTTSPLHIASSSFNQPSTSNKFEHQTALNIIPSEPKMNENYLNASKSQPQEPFIGMLIEGADSCSKDLK
NMCPLADTVLEDQILEASRTNPPCFDFAPLAEKEMKIVTIKATYRDDIIRFRVSLNCGIVELREEISKRLKLEVGTFD
IKYMDDDQEWILVACDADLQECMDILTSSGSNIIRLVVHDIVSILGSSVEISE
>Trifolium_subterraneum_Tsud_chr7.g11400.1.am.mkhc
MGDGAAVTSSATMMEGPSQDENSMDFDYMSELLLDGCWLEASADGSEFLLQSSPFSNPIFDPSFSWPSLENT
EPTRNEDGHVGVEQESQIIVNNAGSSDRQQCEFETRSGEGVSRLWIPPTPNPGLGGSSITEKLIRALKWIKDFN
RNKDMLIQIWVPVNRGDDRPILRANDLPFSLESRSLNLAKYREISVRYQFSAEEKDSKELVAGLPGRVYRDKIPE
WTPDVRFFRSEEYPRVDHAQECDIHGTLAVPVFEQGSRTCLGVIEVVMTTQQSNYGRELETVCKALEVVDLRSS
GHSSLQNVKACDKSYETALPEIQEVLRSACEMHKLPLAQTWISCIQQGKDGCRHSDDNYANCISPVDYACYVG
DSSVRFFHEACMEHHLLKGQGVAGGAFVTNQPCFSADITSLSKTDYPLSHHARLFGLRAAVAIRLRSIYSTSDDY
VLEFFLPVDCNDIEEQKNMLTSLSMIIQRVCRSLRVITDKELDKDHLSSNEVMAVTDGQFATNAVWSESQHRG
MAASLGGEEKFSETMGRKFSEQRRQQETLTLKGDLDCGRVRSPSVEGTFLSSLGINRTGEKRRAKAEKTITLQVL
RQYFAGSLKDAAKNIGVCTTTLKRICRQHGIKRWPSRKIKKVGHSLQKLQLVIDSVQGASGSFQIDSFYSKFSDLS
SPRTSLISTLNQIDNQVSLSTLPDPDSLSPEGASKSPSSSCSQSSFSSHSCSSMSEQQHHKGDVAAGNKDPSVGE
DSVDVVLKRIRSEAELKSLIQDNKTKLMPRSQSQETLGENPKTEHHKSLLKTARKASQKEDSHRVKVTFGDEKTR
FRLPKKWGYEDLVQEIARRFNVSEMSKFDIKYLDDDYEWVLLTCDADLEECIDVSQSSETSTIKLCLQPSSNFIRSS
LEFR
>Trifolium_subterraneum_Tsud_chr7.g15020.1.am.mkhc
MHEDQQLSLPSIQTPALPNQNEVLAIDQWPPTQMPFFCTCCQVLREIIHANGFQFEKLEIHGRVGLITHAIHHQ
LPVNGNNPNYQMIESSLDEIKKYLAKYCVDRNSSGYFILQDPMSAFYETLCTGLDWIDDINMEGLLDNNQTNS
DDMGENGTPDKKDLSAQRKKSASLTLSDLRDYFHLPIEEASDHPNVKLCPTVLKKTCRKAGLQRWPYRKVKSLL
KQIALMGAQLDGQDSTARARTEHEINRLKQEMIAHCGGIIPTAMYNIAAFLPLQYQQQQ
>Trifolium_subterraneum_Tsud_sc00745.00.g00050.1.au
MDISPFDDFFNIDWENQFSYQSNKLYINEFIDFENFNFDFSLPQLDENLEKEPKPINLVLPELVHNNEKDESVCQS
AFSGSSEEVGNGFVKKEKKWEIQDMPFGENGFSFAETYAKSGNEVENQDFPKSLILASSSLIRKKRSCLLQFDEIK
KHFDVPITMAAKKMNVGVTILKRRCRELKINRWPHRKLKSLMVLIDNLKEMGLENEVAKLEKHKKMLENIPG
MELNEEIKKLRQSCFKANYKKRRTSSFHP
>Trifolium_subterraneum_Tsud_sc01256.00.g00010.1.au
MVEEFVHACSEHHLEEGQGISVKALQSNHPFFYTDVKAYDISEYPLVHHARKYNLNAAVATRLRSTYTNDDDYV
LEFFLPINMIGSSEQQLLLDNLSDTMRRICKSLRTVSEGELRGIEGSRDGFLKENISGFFPMSKGNSQIAFSTGDP
DLFQMPLMAGSHSQVTSESRKQAEKGRNAVEKNVSLSVLQQYFSGSLKDAAKKIGGETPSIFVKICPTTLKRICR
QHGISRWPSRKINKVNRSLKKIQTVLDSVQGVEGVLKFDPHTGGFVAGGSIMEQVGTHNKNLMFPEKSSVENS
AIKLEDDGVLISNSCEGDLKKDNNVSSVDYIQDSTSWLWTKTRECPKQNSIDSVLEKEEDQCGLNNSSLHDIASN
SSFSSIELGLDEGKGVDEPNHPTSSSMTDSSNAFGSMVRGSSSGSQSIENHKNSKIKSICVDSESKFAVKATFRGD
TIRFKFDPCLGCFQLYEEVATRFKLRNGSFQLMYLDNEEEWVLLVNDSDLKECVQVLSDSGTHCVKLLVRHIHIV
DSSQICLSI
>Vigna_angularis_vigan.Vang0090s00160.1
MEFASEHDFPNFETIPHTFSSCFFSAQLYPTDNTILFFFLFACLDEWYFNGVCDFRTMEVSLSPLEGFSGHDWTQ
QLPYSSKQLCLNEFPDLENLDLDFSLPPLGEDLEEVEQKPLNIIVVPEEGHCGREKGFGDALLAIENGSAICVKKEE
VQNMEQMAVPSASSGSKKKKPCALEFEEIKKHFDVPINEAAKQMNVGLTMLKRRCRELNIMRWPHRKLKSLQ
LLIENVKVMYPDMLVMSYELGLADEVPMLEKHKRLLEKLPGLELCAKAKKLRQACFKANYKRRRFMGMTLQA
>Vigna_angularis_vigan.Vang02g05230.1

MSESKEENPDFLPKSKPQEEHGFAMDFDIGFESNTKAVIENPVENYDNKKYVSPSLVAVPPSENSDGYCLIKER
MTQALRHFKELTEQNVLAQVWAPVRNGNRYALTTSGQPFVLDPHSNGLHQYRTVSLRYMFSVDGENDEILGL
PGRVFQQKIPEWTPNVQYYSSKEYQRLNHAQHYNVRGTLALPVFEPAGHSCVAVLELIMTAPKINYAPEVDKIC
KALEHALAEILEILTIVCETHSLPLAQTWVPCKHRSVLAHGGGHKKSCSSFDGCCMGQVCMSITEVAFYVIDAHT
WGFHEACVEHHLQQGQGVAGRAFLSHNMCFCGNITQFCKTEYPLVHYALMFGLTSSFAVCLQSSHTGNDEYVI
EFFLPPRITEFNEQKTLLRSILATMKPHFQSLKIAAGVELEQNCSIEIIEPRKERVHLWFESIPITQSSMSPPRHASPS
MGERLPLEPSEQQITTDFDDINDGRNLGDNTDGHIDQNTSLEPRTKKKPSERKRGKAEKSISLDVLQHYFTGSLK
DAAKSLGVCPTTMKRICRQHGISRWPSRKIKKVNRSLSKLKCVIESVHGAEGAFGLNSLSNGSLPIAAGSFSEPST
SNKFNCQTSLTIRPSEPKINEQDFDASRASEKNRQAGLEAHFLSTRTQNPEKAINEKVVAVQEIGTKGTTRFRTGS
GSSEESANPSPHGSCQGSPPNEMSPPKDIFVTGTSEQCLVLRGSLGSTLHSTSTPNRATANPMPNFVETTEPQE
PFGGQLLEGVARGIGNLVSNGYLNEPVCSCPSIATQTYLVLNPCAFTEQLCSLSYQKNSSRLLCQKRKEIHDQPFH
>Vigna_angularis_vigan.Vang05g00050.1
MEYGGFSDIFAPETEFMDELFVEGCWVETRVGCGTEASKWNRSMESNGAHIIFEEESEAESLMVGKRWWIGP
RGNPGPSSSVKERLVVAVGYLKEYAKNSNMVIQVWVPARRGCEVGIHHHQDFPYTLDYTNNGDATGSFQFHE
DWLSDHWPPNIRFLRTHDYTRLHHYDLRPGSLALPVFQRGTGICLVQVLTCVCKAHELPLALTWAPCIQQGKSG
CGHSNDENYVSTVDPACFVADVDVLGFLEACSEYHLLGGQGVVGTAFTTSKPCFANDITAFTKAEYPLAHHAN
MFGLHAAKGKGVSVSLEYLQEPKQEFKVTTNGNEQVFSDLEGTSGAGVGGRRGGRKSGDKRRTKAEKTISLPV
LRQYFAGSLKDAAKSIGVCPTTLKRICRQHGITRWPSRKIKKVGHSLRKLQLVIDSVQGAEGAIQIGSFYTSFPELS
SANGVSESPKINSDNSKFYSENGLFSNQGVTSTSSCSQSCGGTFRVKATFGDEKIRFSLQPNCGFRDLQMEIARR
FNLKEMSKIQVKYLDDAQEWVLLTCDADLEECKDINRSSQSRTVRLFLFHASPLNHSTNAFGSTSPT
>Vigna_angularis_vigan.Vang05g03830.1
MDNNCNNYSDSFHSPLIGNYIEPDPALIYILNDQPNVQQRNAQQNDPFVVEQDHVSFTGLTIQDVSTHSDNDF
TIGGVPNLENGGWSTRKRKFPYMDSMIEGHPVHGDNTFPNELQFWPPGPFDIDIDASFDPFLHNLIDRDISIF
DLSNDELIAPSSQQNALPQHQHAVPGQQNALCKQPNAPPQNQNLYPFGLEQIHVPILNPTFQDQSLHVNYNF
ATGEVPNIDMEGPSRRKVKQPMAEHPLLKHGILNEEDVPVRFSSSFSFSLNFKHSAGFKFEKLEIHGSLGVIGHAI
FHVQDMTPGGEPPREVYQMIKQLLYLSLLTFIFSGITSFGLKSIEQIRSFLVAYCKDQTRLGFVTLDDPLSAYYDTICT
GLDWAEHNDGDDDLSPRWSSSLNSQRKRVPKMTMNDLSPFFHLTIRDAADKLEVSDSVVKKISRLGNLKRWP
QRKLQSLAKDVRVLRKALDSPYEGTRQRVRQEIQRLQLEMVAICGGVTPTGIEIIQFEEE
>Vigna_angularis_vigan.Vang06g08410.1
MSESEEENTDCVPRSKPAEEVGCTMDFDLDLETSWPLDHMSFGSNPMSPFLFSSNSDQPYSPLWAFSDGEDA
KFPASAFSDCHKIFPCDSNSIAEKPEENDDNKKLLPPLAPMTPLENVGGYCVIKERMTQALRYFKELTELNVLAQ
VWAPVRNGNRYVLTTSGQPFVLDPHSNGLHQYRTVSLMYMFAVDGENDGSLGLPGRVFQQKLPEWTPNVLY
YSSKEYPRRDHAQHYNVRGSLALPVFEPALQSCVGVLELIMTSQKINYAPEVDKICKALETVNLRSSEILDHPYTQI
CNEGRQNALSEILEILTVVCETHNLPLAQTWIPCKHRSVLAQGGGVKKICSSFDGSCMGKVCMSTTDVAFYIIDA
HLWGFREACVEHHLQQSQGVAGRAFLSHSMCFCSSITQFCKTDYPLVHYALMFGLTSCFAICLRSSHTGTDDYV
LEFFLPPRVTDFHEQKTLLASILATMKQHFQSLKIASGVELEDGSIEIIEATIERIHTRHESIPIAPSIRSPPRPDTSPN
MEEEVSLDPSEQHILMYCNGTNHGANLSDKAGGKIDHMITLETKTSKKPLERKRGKTEKSISLEVLQRYFAGSLK
DAAKSLGVCPTTMKRICRQHGISRWPSRKINKVNRSLSKLKRVIESVQGAEGAFGLNSLSTSPLPIAVGSLPEPST
RNKFSQPVSMSIRPSEPQMKVNELNASKELETNREAGMEDQLVGGRIHNLERVTNDKGGSTQEIGKEPKRTRT
GSGSSEDSTNPTSHGSWHDSIPNESSPVKDIFITSNHDQCAVLRRSPESTLQPATNTPNQATPFCMPEFVAAEL
QEPFGGMLIEDADKRS
>Vigna_angularis_vigan.Vang09g02510.1
MENQQLTFWNSNYQIDENSFAPTCQFSYDYFRKSYHVSIFNFCRGNASDGFHWPYEFPLQDSYFDVVPFMKC
YYPHDILYETLPLEPTPLSFQGFAGADYDFSDVKNVLSAWNEVDVSDHKPAFSSSNDGGSVNETMEEVKGKQH

REERISSSARILSRKTVSQYFYMPISQAAKELNVGLTHLKKRCRELGIQRWPHRKLMSLQTLIKNMQIWNLKIKQ
EGLDKLVSRLTTRRGNS
>Vigna_angularis_vigan.Vang11g11480.1
MIMDSHPLMSWSSYYQIEENPLPFTCQFSYSENSEVYPPPLQDLPIQEYHHDAVPLTEYYPSDPLYETLAIEPTPT
LRADCDFYDISKGLPVWNEVNAVFDSEVDFLFRKKEESLKEMVDDRKVNEGREEITNPRNSRKLSRKTISEYFY
MPITQAAREMNVGLTLLKKRCRELGIRRWPHRKLMSLQTLINNVQELLKEEGPESEEKLRFAIETLENEKKLLEE
MPDIELEDNTKRLRQACFKANYKKRKLGQREPECSSWSGAMSEYNNESEEELDMKYLLSQLA
>Vigna_radiata_Vradi0553s00010.1
MEYPFSSKEREIGDWQSFGNQLVGSTSLDSRMSNSISEDMPNSFSELMNFDTYAGLCTSPSISDQILANELPSFA
SLPYSLPDGFNLVQQNNGQCYMSGVGRNDNDMESSPIYGEKVACQQVDTLLGFLNDANEANNLNSKLKIIGS
SQHLNNSDAGNYIMSRPPALSLDERMLRALSFFKESAGGGILAQVWVPIKNGDQFILSTSEQPYLLDQMLAGY
REVSRTFTFPAEGKSGCFLGLPGRVFTSKVHEWTSDVGYYSMNEYLRFEHAINHHVRGSIAFPIFEMHSELLCCA
VLEIVTTKEKPDFNREFEIVCRALQLVNLSTAKPLRCLPQCLSNNKKATLTEIVDVLRSVCHAHRLPLALTWLPCCY
TEGSRGEVTRIRTKGCHSTISGKNILCLEESACYITDRALAGFVRACTEHHIEEGKGIAGKALRSNHPFFCPDVKAY
DISEYPLVHHARKYNLNAAVAIRLRSTYTNHDDYILEFFLPVNMRGGLEQQLLLDNLSGTMQRICSSLRTVSDAE
LSRIDSSQQGFEMKNAPYFPPLSCQNSGVPLINGDYHSVPMMPSTPTNAGGNEIEPSANQGRNGAKRQVQK
NRSTSEKNVSLSVLQQYFSGSLKDAAKNIGVCPTTLKRICRQHGISRWPSRKINKVNRSLKKIQTVLDSVRGVEG
GLKFDPSMGAFVARGSIIQETDAHKSLLFPEKNIIKDPAHITQEAVPVPRAPCNEVENFSFKLEEELKKTNSFSVGC
CEDSKSMAIDDGSCEMESLCTKLQDCPEQACLGSVLPKEQEKWTRNKSGLRVENFKCSILGQSSNSLIGKEMDI
GVDGDAEVVEPNHPSSSSLTDSSNDSGSMMHSISSGSESFKNHNQSKVKSTIVDSGSKLIVKAIYREDTIRFKFD
RSAGCFRLYEEVAARFKLQTGSFQLKYLDDEEEWVMLVSDADLQECLDILDDIGTCSVRFLVRDLPSILTSSGSSNS
YLGGSS
>Vigna_radiata_Vradi08g18730.1
MSESEEENTDCVPRSKPAEEGGCTMDFDLDLETSWPLDHMAFGSNPMSPFLFSSNSDQPYSPLWAFSDGEDT
KFPASAFSDCHKIFPCDSNSIAEKPEENDDNKKILPPLAPMPPLENVGGYCVIKERMTQALRYFKELTELNVLAQV
WAPVRNGNRYVLTTSGQPFVLDPHSNGLHQYRTVSLMYTFAVDGENDGSLGLPGRVFQQKLPEWTPNVLYYS
SKEYPRRDHAQHYNVRGSLALPVFEPALQSCIGVLELIMTSQKINYAPEVDKICKALEVGFLLTWLIMPIICSYSKF
EEFGNFGPSIHSGDLTIRHGASPSVILLLFIILTLCSHRYNLAMLLNQICNEGRQNALSEILEILTVVCETHNLPLAQT
WIPCKHRSVLAQGGGVKKICSSFDGSCMGKVCMSTTDVAFYIIDAHLWGFREACVEHHLQQSQGVAGRAFLS
HSMCFCSNITQFCKTDYPLVHYALMFGLTSCFAICLRSSHTGTDDYVLEFFLPPRITDFHEQKTLLASILATMKQHF
QSLKIASGVELEDGSVEIIEATIERIHTRLESIPIAPSIRSPPRPDTSPNMEEEGPLDPSEQHILMYCNGTNHGSNLS
DQAGGKIDHMITLETKTSKKPLERKRGKTEKSISLEVLQRYFAGSLKDAAKSLGVCPTTMKRICRQHGISRWPSR
KINKVNRSLSKLKRVIESVQGAEGAFGLNSLGTSPLPIAVGSLPEPSTPDKFSQPVSMNIRPSEPQMKVNELNAS
KELETNREAGMEDQLVGGRIHNIERVTNDKRGFTQEIGKEPKRTRTGSGSSEDSTNPPSHGSWHDSIPNESSHV
KDLFITSNHDQCAVLRRSPESTLQPATNTPNQSTPFRMPEFVAAELQEPFGGMLIEDAGSSKDLRNLCPSVAEAI
LEDLVPEACGTNPPALDLSPKQSMGAPNKTVTPFAATKEMKTVTIKATYREDIIRFRVSLTCGIVELKEEVAKRLKL
EMGTFDIKYLDDDHEWVLIACDADLQECMDVSRSSGSNIIRVLVHDMTSNLGSSCESSGE
>Vigna_radiata_Vradi10g01040.1
MEFASEHDFPNFEIIPHTFASCFFSAQLYPTDKAMMDVSLSPLEGFSGHDWTQQLPYSSKQLCLNEFPDLENLD
LDFSLPPLGEDLEEVEQKPLNIIVVPEEGHCGREKGFGDALLAIENGSAICVKKEEVQNIEQMAVPSGSSGSKKKK
KPCALEFEEIKKHFDVPINEAAKQMNVGLTMLKRRCRELNIMRWPHRKLKSLQLLIENVKVMYPDMLVMSYEL
GLADEVPMLEKHKRLLEKLPGLELCAKAKKLRQACFKANYKRRRFMGMTLQA
>Vigna_radiata_Vradi01g14220.1
MSESKEENPDFLPKSKPQEEHGFAMDFDIGLESLWPLDHISSVSNPMSPFLLSTIEQPFSPVWAFSDVEDEKQIR

IAIAGNTKAVTENPVENYDNKKYVSPSLVAVPPSENSDGYCLIKERMTQALRHFKELTEQNVLAQVWAPVRNG
NRYALTTSGQPFVLDPHSNGLHQYRTVSLRYMFSVDGENDEILGLPGRVFQQKIPEWTPNVQYYSSKEYQRLN
HAQHYNVRGTLALPVFEPAGQSCVAVLELIMTSPKINYAPEVDKICKALECHNVPLVRSSDPVVFSLQAVNLRSS
EILEHPYNQICNEDRQHALAEILEILTIVCETHSLPLAQTWVPCKHRSVLAHGGGHKKSCSSFDGCCMGQVCMS
ITEVAFYVIDAHTWGFHEACVEHHLQQGQGVAGRAFLSHNMCFCGNITQFCKTEYPLVHYALMFGLTSSFAVC
LQSSHTGNDEYVIEFFLPPRITEFNEQKTLLRSILATMKPHFQSLKIAAGVELEQNCSIEIIEARKERVHLWFESIPIT
QSSMSPPRHASPNMGERLPLEPSEQQTTTDFDDINDGRNLGDNTDGHIDQNTSLEPRTKKKPSERKRGKAEK
SISLDVLQHYFTGSLKDAAKSLGVCPTTMKRICRQHGISRWPSRKIKKVNRSLSKLKCVIESVHGAEGAFGLNSLS
TGSLPIAAGSFSEPSTSNKFNRQTSLTIRPSEPKINEQDFDASRASETNRQAGLEGQFLSTRTQNPEKVINEKVVAI
QEIGTKGTTRFRTGSGSSEESANPSPHGSCQGSPPNEMSPPKDIFVTGISEQCLVLRGSLGSTLHSTSIPNHATAY
PIPNFVETTEPQEPFGGQLLEGVGSSKDLRNLCPSTDAVLEDQVPEPYRMNPQCSDLAPVQHRDTNNMTPFA
VRKEVKNVTIKATYREDIIRFKISLNCVIVELKEEIAKRLKLEEGTFDIKYLDDDHEWVLIACDADLQECMDISRSSG
SNIIRLVVHDILPILGSSCESSGDWKCCI
>Vigna_radiata_Vradi11g07090.1
MRSLHVYRQGDEEERAVEREFVFSECGSYVEMQATPILKFVKSHVSRVFQGYKNGVWLCIFAYHADHTPRFYRI
PPLLLVSRNPKLQMVPNLLHDLHVIYKLDRKEEDKDTTLESKGEECQGNSDNFQPSEKVFPVLDQDLNYLPYEE
DESELLENETDVESSPVLQYHEVQVFTIIDYIVVMRSEFAGLLGKKKRAPSDLVAKISLSDLVQYFGMPIVEASRNL
NVGLTVLKRKCREFGIPRWPHRKIKSLDSLIHDLQVLLALPVEHMQEEAKSQELEDMEAAMAVAKRQRMLECE
KENIEKKPFMDIQTETKRFRQDVFKRRHRARAVGKHNSTVSNT
>Vigna_radiata_Vradi02g03910.1
MEDHFSPKGKETGYCTSPCTQLEESTSVDGGMKKSASEDMFSNFSELMNFDTYAGWSNSSSMTYHSLANVFS
SFSSASYPPSDGLNLVEHGNGPCFMTEVSEIHNGIESSPSCEERGIFQQMDIQLGFLDEANDSNSFDSEQNRNG
SYQQFNTSDMCNYMISRSSGRPLDDRMLRALSFFMESVDEGMLAQVWVPIKHGDEFILCTSEQPYLLDPKLA
GYREVSRAFTFSAEGKKGSCPGLPARVFTSHVPEWTSNVGYYNKTEYLRLDHALNHDIRGSIALPVSDMHSHEP
CAVLELVTTKEKPNFDRELEIVTHALQLVNLKTTMPPRLLPQCLSSNKRAALTEIVDVLRAVCHAHRLPLALTWIP
CCYSEGIGDEAERIRIKEGHTSSNEKCVLCIEESACYINDGAIGGFVHACVEHHLEEGQGIAGKALQSNHPFFYTD
VKTYDISEYPLVHHARKYNLNAAVAIRLRSTYTNDDDYILEFFLPVNMTGSSEQQLLLDNLSGTMRRICRSLRTVS
DAEITGIEGSVAGLPKEKLTYFFPMSRRNSQIAFINDDNDSNQKMSLKASNNGIEAAHGQVMNGPRKQIEKKR
STVEKNVSLSVLQQYFSGSLKDAAKSIGVCPTTLKRICRQHGISRWPSRKINKVNRSLKKIQTVLDSVQGVEGGLK
FDPYTGGFIAGGSLMQEIDTHKYILFPERSAVKDPKHATQKPVSVVSAPGSTSENSTIKLDDDDGVCLVENKIVDS
RSALLSNTSVGGFKKDNASSDDCCEDSKSVAMNDGSSQKAKNQDCPEQTCSISLVTDEAEVGVDGGEGIEEHN
NLVNSSSTTNSSNGSGSGSMMHGSSSCSQSFENQKHSKVKPTSVDSESKMIVKASYRGDTIRFKFDPSAGCFQ
LYEEVATRFKLQNGLFKLKYLDDEEEWVMLENDSDLQECVEILHDIGTRCVKFLVRDVPCVLSSCDSNNCFLSDS
S
>Vigna_radiata_Vradi05g14430.1
MMRDGGVTSSSASMTEAPPPDGTITTKTMDFDYMGELFLDGCWLEASVDGSDFLPQSPSFSNPLFDPSFSW
PALETNHNESQGAAFGTQQEGHNNNMVNVVVGCGDGGGQENKNMLIQIWVPVHRGGRPILAANDILFSLD
SRSVNLAKYREISVRYEFSAEEGEVKELVPAEKGGSKELLLGLPGRVFRDKVPEWTPDVRFFRSDEYPRVDHAQE
YDVRGSLAVPIFEQGSKMCLGVIEVVMTTQQINYTPELESVCKALEAVDLRSSKQLNIHNVKACNRSYEAVLPEI
QLVLRSACEMHRLPLAQTWIPCVQQGKEGCRHSEDNYLLCISPVEHACYVGDPKIRPFHESCTEHHLLKGEGVA
GGAFITNQPCFSDDITSLSKKDYPLSHHARLFGLRAAVAIRLRSILNDTDDFVLEFFLPVDCSDSEEQRKMLTSLSIII
QRLCRSLRVISDKELEEANSSIDEMIFLADGGFDRTVICEELQHKGTVALWNTEEKLSETTGRKFSDPRQQQQES
LNFKGNLDCVRECSTSVQGNLSSVGMSKMGERRRAKGEKTITLQVLRQYFAGSLKDAAKNIGVCTTTLKRICRQ
HGIKRWPSRKIKKVGHSLQKLQLVIDSVQGASGAFQIDSFYSNFPELASPNLSGTSMFSTPNQTDNPNSISTQPD

PGSLSPEGTSKSPSSSCGQSSISSHSCFSMSELRQQHHTTDISGNKDSTMAGEDSLDVALKRIRSEAELKSLNQD
KAELLPRSLSQETLGEHPKNQYERPLLKTSSKVDAHRVKVTYGDEKTRFRMPKNWGYEDLMQEIGRRFNVSEM
SKFDVKYLDDDSEWVLLTCDADLEECIDVCRSSENATIKLSLQISSHSLRSSLEFR
>Vigna_radiata_Vradi07g17040.1
MIMDSHPLMSWSSYYQIEEKPLPFTCQFSYSENSEVYPPPLHDLPIQDCYQGAVPLTEYYPSDPLYETLAIELTPTL
RADCDFYDISKELSVWNEVNAVFDSEVDLLFRNKEESLKEMVDDSKVNEGREEITNPRNSRKLSRETISEYFYMP
ITQAARELNVGLTLLKKRCRELGIRRWPHRKLMSLQTLINNVQELLKEEGPESEEKLRFAIETLEKEKKLLEEMPDI
ELEDNTKRLRQACFKANYKKRKLGQREPDCSSWSGVMSEYNNESEEELDMKYLISQLV
>Vigna_radiata_Vradi03g09040.1
MENHQLTFWNSNYQIDENSFAPTCQFSSYDYFRFFPLLYFSTLLSFHVRKSYHVSIFNFCRGNASDGFHWPYEFP
LQDSYFDVVPFMKCYYPHDILYETLPLEPTPLSFQGYYDFSDVKNVLSAWNEVDVSDHKPAFSSSNDGGSVNDT
MEEVKGKHREERISSSARNLSRETVSQYFYMPISQAAKELNVGLTHLKKRCRELGIQRWPHRKLMSLQTLIKNM
QEQVESEGPENDEKIRTAIEMLEKEKSSVEQKPDLELEDKTRRLRQACFKANYKKRKLMTMRIMEPNLPLLPYPT
SIDKGGE
>Vigna_radiata_Vradi05g15890.1
MVIQVWVPARRGCEVGIHHHQDFPYTLDYISNGDATRSFQFHEEWLSDHWPPNIRFLRTHDYPRLHHYDLRP
GSLAIPVFQRGTGICLASFVADVDVLGFLEACSEYHLLGGQGVVGTAFTTSKPCFANDITAFTKAEYPLAHHANM
FGLHAAVAIPLRSVSSDFVLEFFLPKDCHDTQDQKQILNSLSMLVQQACRSLHVVLDKEEEEELVSHHHHHHSKE
MESSSWIAHMMEAQQKGKGVSVSLEYLQEPKQEFKVTTNCNEQVFSDLEGTGGAGVGGRRGGRKSGDKRRT
KAEKTISLPVLRQYFAGSLKDAAKSIGVCPTTLKRICRQHGITRWPSRKIKKVGHSLRKLQLVIDSVQGAEGAIQIG
SFYTSFPELSANGVSESPKINSDNSKFYSENGVKATFGDEKIRFSLQPNCGFRDLQMEIARRFNLKEMSKIQVKYL
DDAQEWVLLTCDADLEECKDINRSSQSRTVRLFLFHASPLNHSTNAFGSTSPT
