# Supplementary material for: Genome-Wide Identification, Characterization, and Regulation of RWP-RK Gene Family in the Nitrogen-Fixing Clade
Source: Plants (Basel). 2020 Sep 11;9(9):1178. doi: 10.3390/plants9091178 (PMC7569760; doi:10.3390/plants9091178)
Supplement: Supplementary file 1 [file plants-09-01178-s001.zip › Supplementary_data/FigureS4.pdf]

Motif	Consensus sequence	Found in subfamily
1	VLQQYFAGSJKDAAKSJGVCPTTLKRICRZH	ALL
2	GQGLPGRAFQSKVPEFTPBVTYYSKTEYPLVHHAR	NLP(NLP-1,NLP-2,NLP-3)
3	GISRWPSRKIKKVNRSJKKLQ	ALL
4	NYQAALAEILEVLRSVCETHRLPLAQTWIPC	NLP(NLP-1,NLP-2,NLP-3)
5	GTFDJKYLDDDEEWVLLTCDADLZECIDI	NLP(NLP-1,NLP-2,NLP-3)
6	CLSTVESACYVNDAHVWGFHEACSEHHLQ	NLP(NLP-1,NLP-2,NLP-3)
7	MFGLNAAVAIRLRSTYTGDDDYVL	NLP(NLP-1,NLP-2,NLP-3)
8	EGSKVRVKATYGEDKIRFRFDPSWGCVELYEEVAKRFKLZD	NLP(NLP-1,NLP-2,NLP-3)
9	PVFEPSSQSCLGVLELVMTSZKINYAPELEKVC	NLP(NLP-1,NLP-2,NLP-3)
10	EEMPDLZLEDETKRLRQACFKANYKKRRL	RKD(RKD-1,RKD-2)
11	VIDSVQGAEGAFQIDSFYGSF	NLP(NLP-1,NLP-2,NLP-3)
12	EEQKLLLNSLSATMQRHCRSL	NLP(NLP-1,NLP-2,NLP-3)
13	GQGVLAQVWVPVKNGNRYVLTTSEQPF	NLP(NLP-1,NLP-2,NLP-3)
14	KGSRKQGEKKRTKAEKTISLS	NLP(NLP-1,NLP-2,NLP-3)
15	SNGLAQYREVSRMYTFSAEGE	NLP(NLP-1,NLP-2,NLP-3)
16	PGPYSSVKERMLRALRYFKES	NLP(NLP-1,NLP-2,NLP-3)
17	KALZAVBLRSSEILR	NLP(NLP-1,NLP-2,NLP-3)
18	TELQEPFGGMLIEDAGSSKDLRNLCPSVA	NLP(NLP-2)
19	EFFLPVBCTDS	NLP(NLP-1,NLP-2,NLP-3)
20	SSGSNIIRLLVHDIPSNLGSS	NLP(NLP-2,NLP-3)
21	VLAHGGGLKKSCSSFDGSCMG	NLP(NLP-2)
22	PGLPGRVFISKVPEWTSNVGYYSKTEYLR	NLP(NLP-1,NLP-2,NLP-3)
23	HNHPTSSSMTDSSNGSGSMMHGSSSGSQS	NLP(NLP-3)
24	MDFDYMDELLLDGCWLETT	NLP(NLP-1)
25	SGSSEDSTNPTSHGSCHGSPPNESSPVKD	NLP(NLP-2)
26	GEESEEKLRBAIEILEKEKKL	RKD(RKD-2)
27	IQQGKEGCRHSDDNY	NLP(NLP-1)
28	YNVRGSJA	NLP(NLP-1,NLP-2,NLP-3)
29	PKGKGIGYWTSPGAQLEGSTSLDGGIKNS	NLP(NLP-3)
30	RTVSDAELEGIEGSQ	NLP(NLP-1, NLP-3)
31	NNVEPPQQNQTQPPNNQDVRSLCS	RKD(RKD-3) (2)
32	RSSZSRTIKLSLQQS	NLP(NLP-1)
33	LFSTSSDQPFSPLWAFSDGED	NLP(NLP-2)
34	FILQDPLSAFYEALCTGLDWDE	RKD(RKD-3)
35	GRRWWIGPRAN	NLP(NLP-1)
36	YKNGVWLCIFAFHADHTPQFSRIPSLLLVTR	RKD(RKD-1)
37	WPPPPVPFSCSCCQVLREI	RKD(RKD-3)
38	DMFNNFSELMNFDTYAG	NLP(NLP-3)
39	GASKSPSSSCSQSSSSSHSCS	NLP(NLP-1)
40	KAAAMAVAKRQKMLESEKENI	RKD(RKD-1)
41	NGFQFEKLEIHGRLGLISHAI	RKD(RKD-3)
42	IAAGVELEESSSVEJIEASNER	ALL
43	NKBMLIQIWVPVRRG	NLP(NLP-1)

44	EAILEDMVPEACGTNPPGPDLAPKQSMGTLNKTVTPFAARK	NLP(NLP-2)
45	LASPNLSGTSLFSTLKQSDNP	NLP(NLP-1)
46	HDWTQQFSYSSNQLCFNEFPDLENFBFDF	RKD(RKD-1)
47	FENQKHSKVKSTCVD	NLP(NLP-3)
48	KLLPRSQSQETLGEHPKTEYH	NLP(NLP-1)
49	GLTBEVAMLEKHKRL	RKD(RKD-1)
50	VHSRLESIPITQSAKSPPRPD	NLP(NLP-2)
