# Supplementary material for: Genome-Wide Identification, Characterization, and Regulation of RWP-RK Gene Family in the Nitrogen-Fixing Clade
Source: Plants (Basel). 2020 Sep 11;9(9):1178. doi: 10.3390/plants9091178 (PMC7569760; doi:10.3390/plants9091178)
Supplement: Supplementary file 1 [file plants-09-01178-s001.zip › Supplementary_data/FigureS8.pdf]

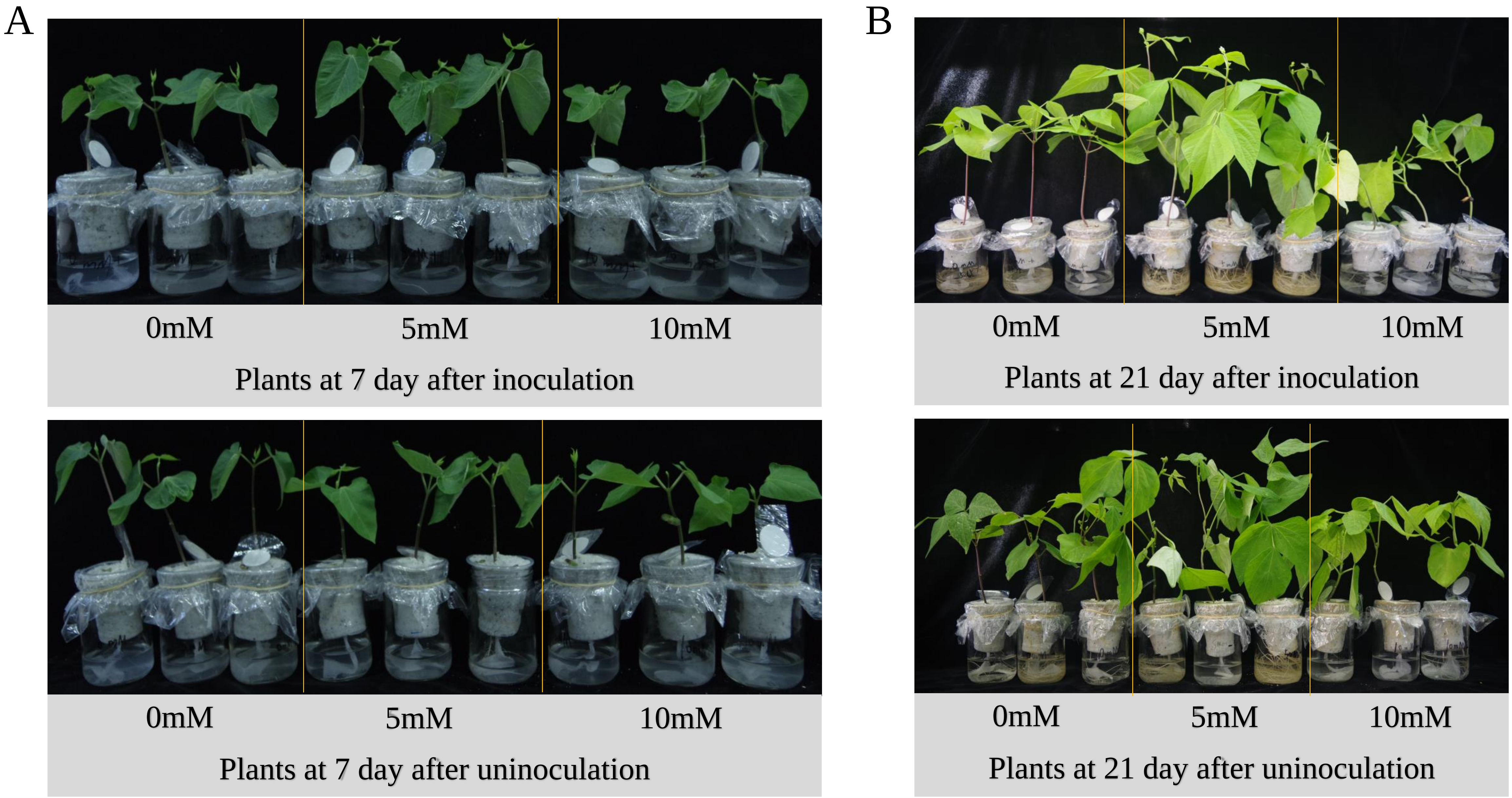
A
0mM
5mM
10mM
Plants at 7 day after inoculation
0mM
5mM
10mM
Plants at 7 day after uninoculation
B
0mM
5mM
10mM
Plants at 21 day after inoculation
0mM
5mM
10mM
Plants at 21 day after uninoculation
