# Supplementary material for: Genome-Wide Identification, Characterization, and Regulation of RWP-RK Gene Family in the Nitrogen-Fixing Clade
Source: Plants (Basel). 2020 Sep 11;9(9):1178. doi: 10.3390/plants9091178 (PMC7569760; doi:10.3390/plants9091178)
Supplement: Supplementary file 1 [file plants-09-01178-s001.zip › Supplementary_data/TableS8.docx]

| **Gene name** | **Gene identifier** | **Primer (Forward/Reverse)** | **Length（bp）** | **Tm** | **GC%** | **Product size（bp）** |
| --- | --- | --- | --- | --- | --- | --- |
| *PvNLP1* | *Phvul.004G114100* | AATGATGGAAAGAATCTTGGCG | 22 | 57.36 | 40.91 | 230 |
|  |  | GATATCCCATGTTGCCTACAGA | 22 | 57.38 | 45.45 |  |
|  |  | GTGTGAACTTGAAATCCTCGAG | 22 | 57.35 | 45.45 |  |
| *PvNLP2* | *Phvul.007G071900* | TACGAAGCTCTCATACAGGAAC | 22 | 57.37 | 45.45 | 115 |
|  |  | TTCATTGTTGCCAATATGGACG | 22 | 57.63 | 40.91 |  |
| *PvNLP3* | *Phvul.008G291800* | GAGACTCACTTCTCAACGGTG | 21 | 58.33 | 52.38 | 81 |
|  |  | CTGAGAGCATGCTTCCTTGAAG | 22 | 59.32 | 50 |  |
| *PvNLP4* | *Phvul.009G011200* | CTACGAAGCTGTACTACCTGAG | 22 | 57.46 | 50 | 208 |
|  |  | AGACTCGTGAAAAGACCTGATT | 22 | 57.39 | 40.91 |  |
| *PvNLP5* | *Phvul.009G115800* | CTTTGTGGCAGATGTAGATGTG | 22 | 57.39 | 45.45 | 129 |
|  |  | GCAGTTATGTCATTTGCAAAGC | 22 | 57.43 | 40.91 |  |
| *PvNLP6* | *Phvul.011G052100* | GATACTTGATGTGTTACGTGCC | 22 | 57.48 | 45.45 | 101 |
|  |  | CAGATTCATCCCCTAATCCCTC | 22 | 57.43 | 50 |  |
| *Actin* | *Phvul.008G011000* | TGCATACGTTGGTGATGAGG | 20 | 57.98 | 50 | 190 |
|  |  | AGCCTTGGGGTTAAGAGGAG | 20 | 58.71 | 55 |  |
