# Supplementary figures and images for: Genome-Wide Identification, Characterization, and Regulation of RWP-RK Gene Family in the Nitrogen-Fixing Clade
Source: Plants (Basel). 2020 Sep 11;9(9):1178. doi: 10.3390/plants9091178 (PMC7569760; doi:10.3390/plants9091178)

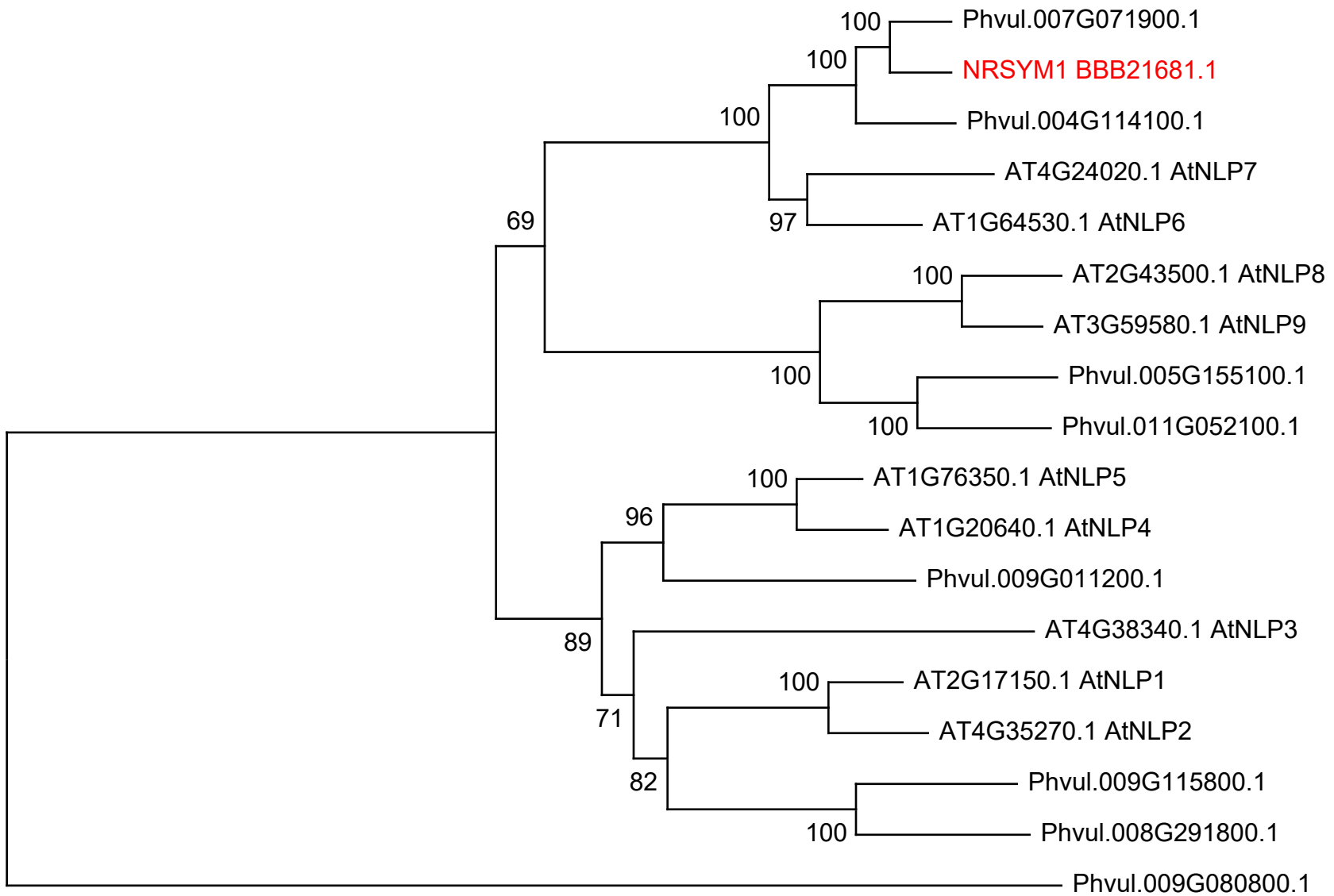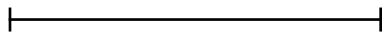

0.50

Supplement: Supplementary file 1 [file plants-09-01178-s001.zip › Supplementary_data/FigureS10.pdf]

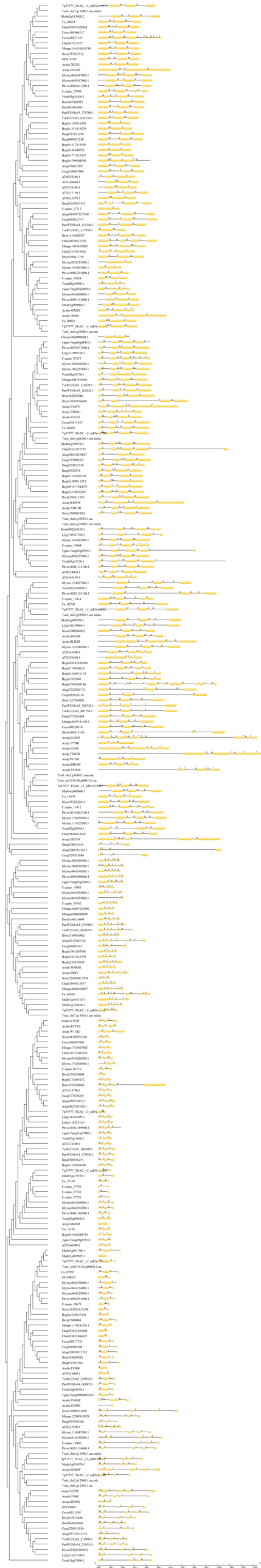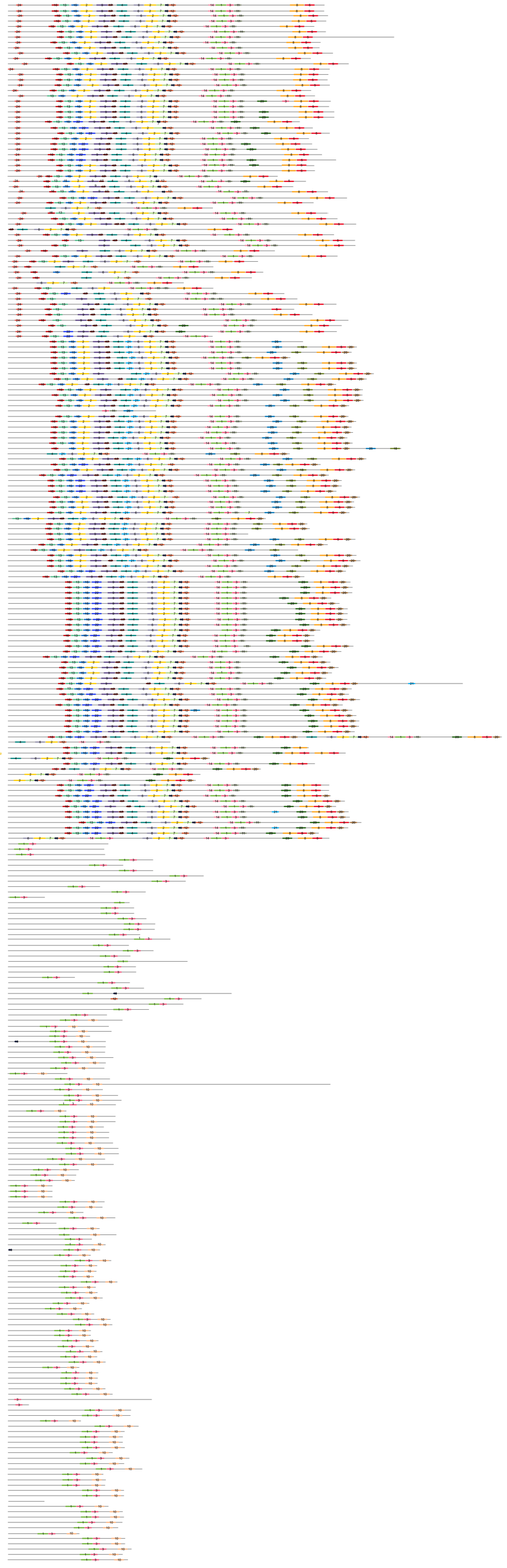

Supplement: Supplementary file 1 [file plants-09-01178-s001.zip › Supplementary_data/FigureS5.pdf]

**A**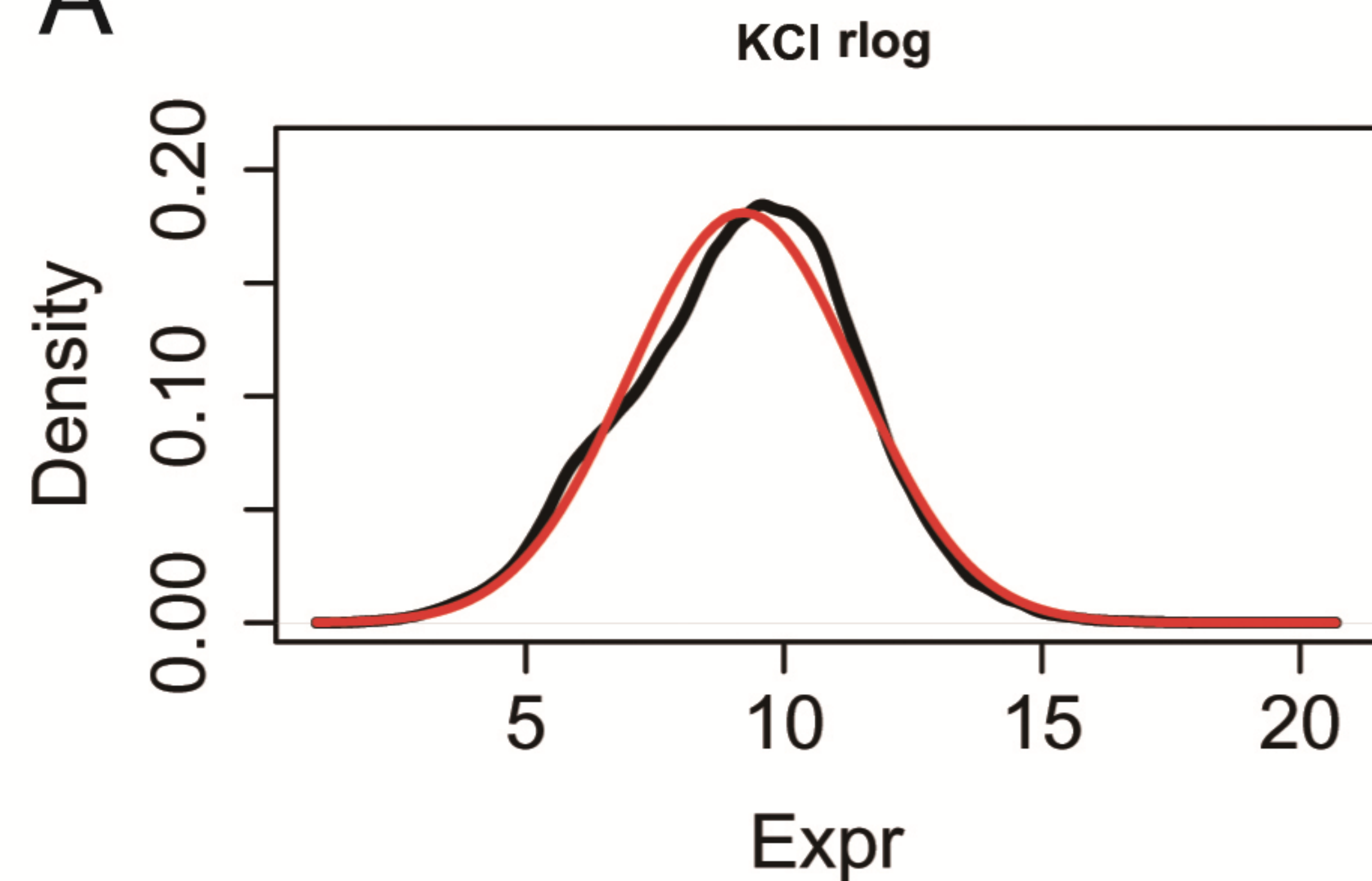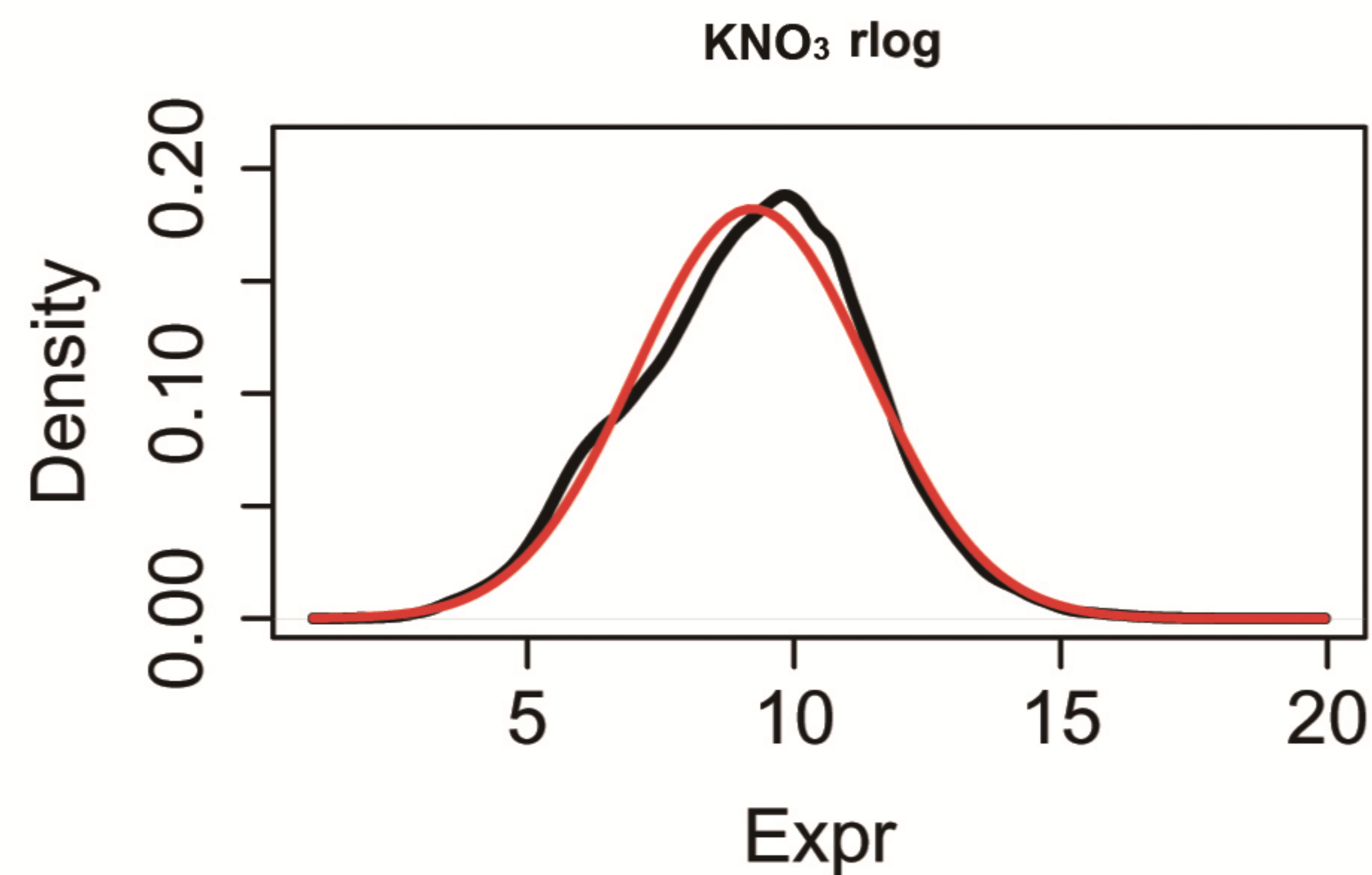**B**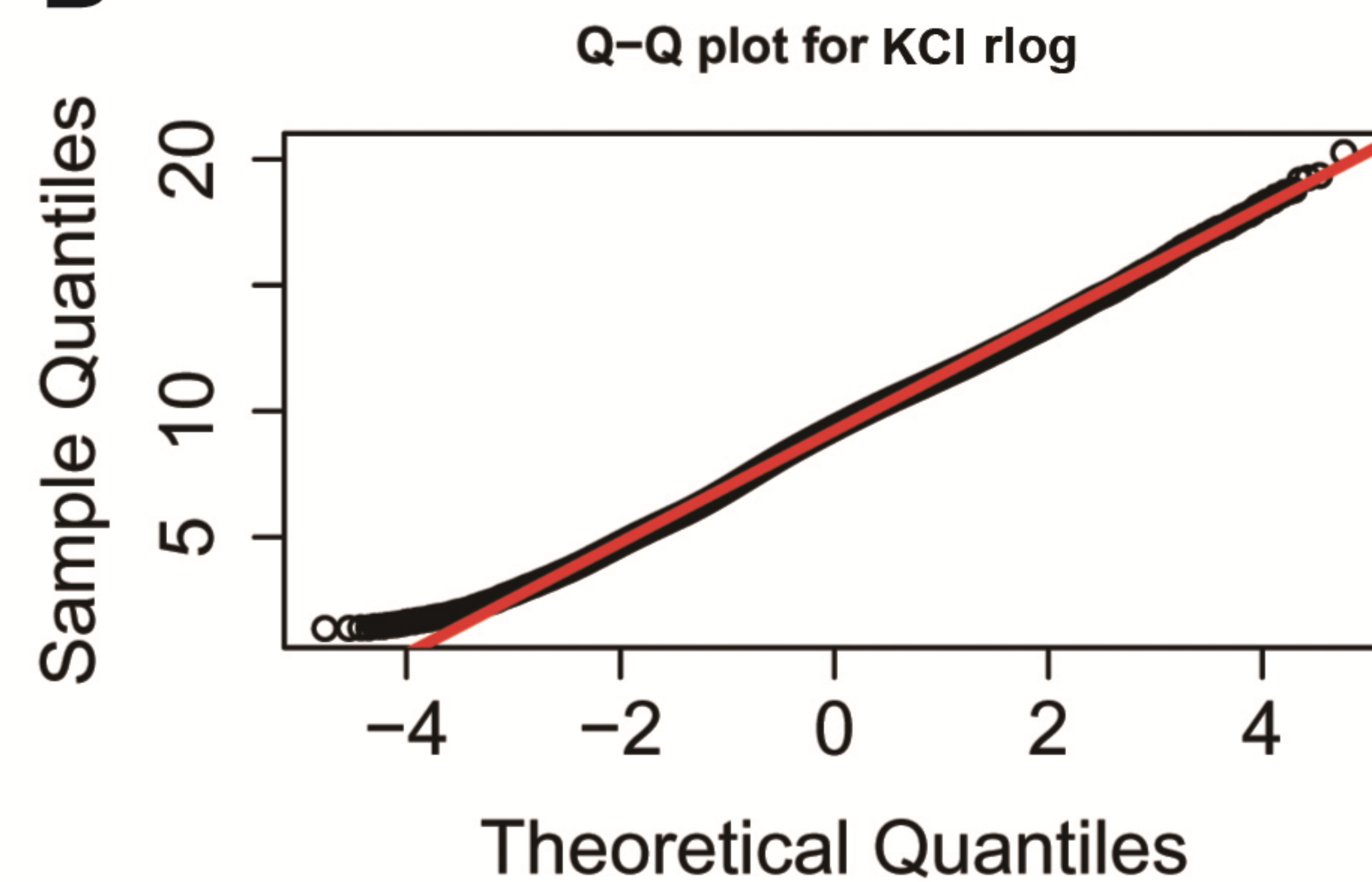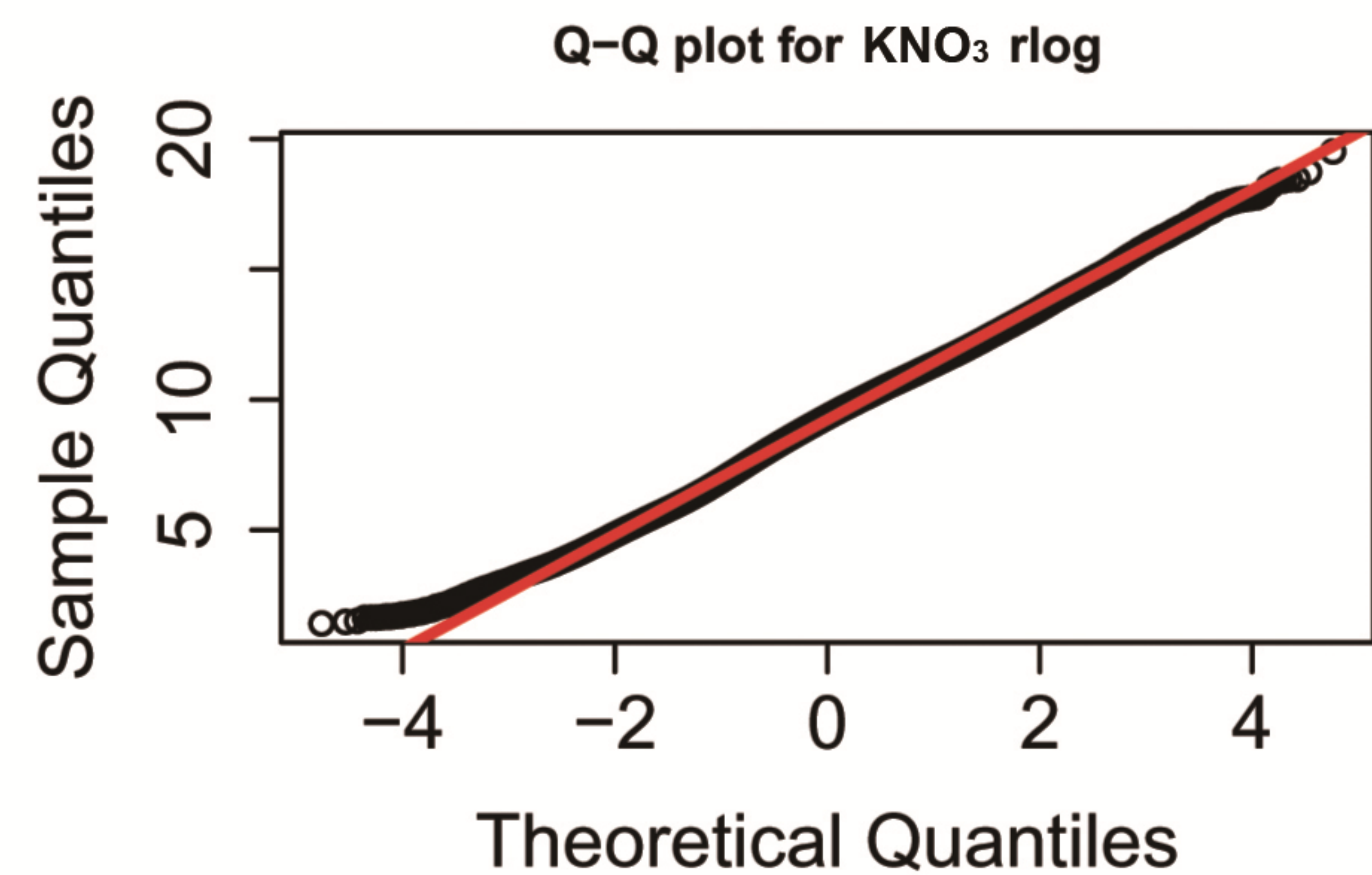

Supplement: Supplementary file 1 [file plants-09-01178-s001.zip › Supplementary_data/FigureS6.pdf]

A

KCl vs KNO<sub>3</sub>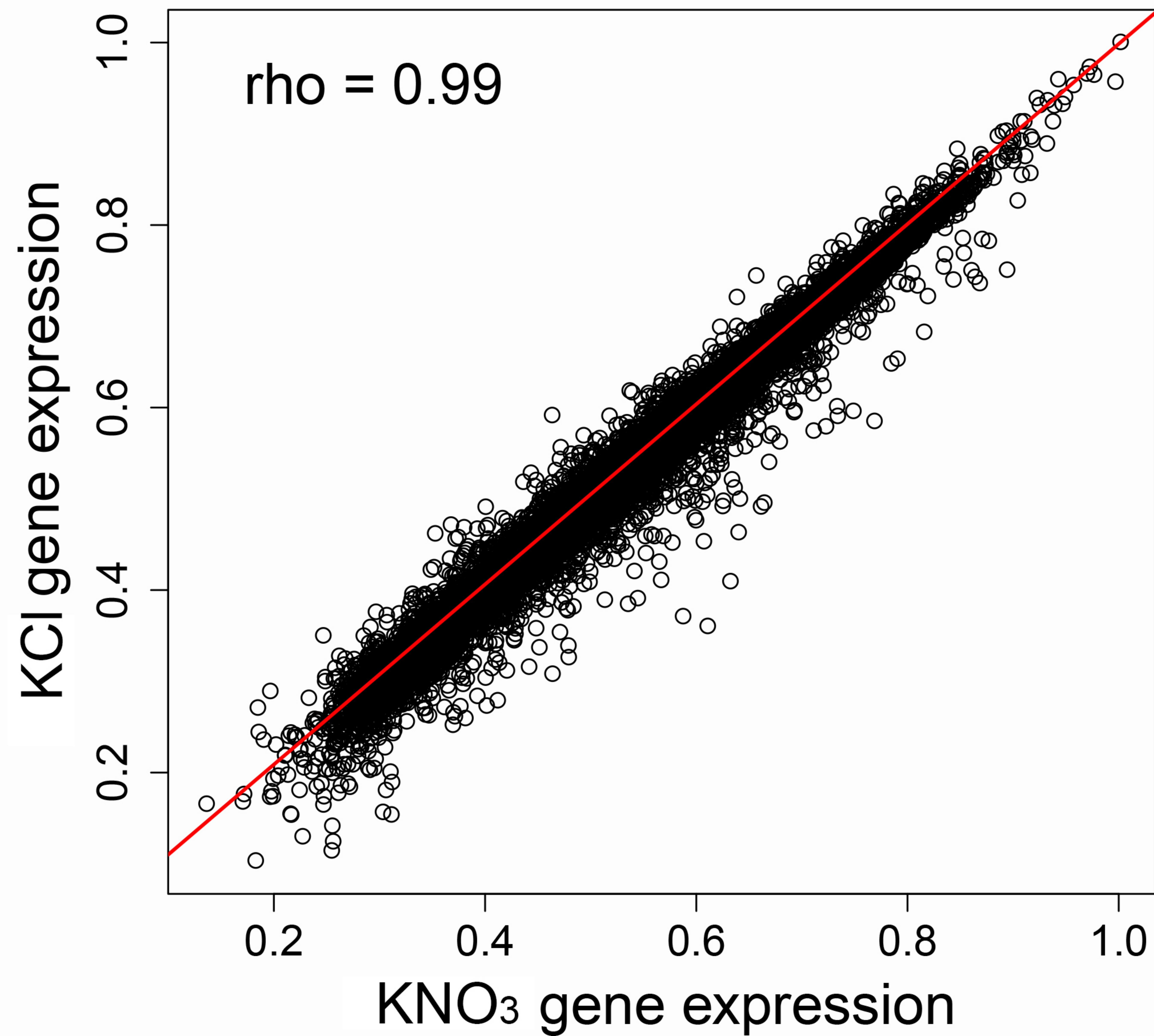

B

KCl vs KNO<sub>3</sub>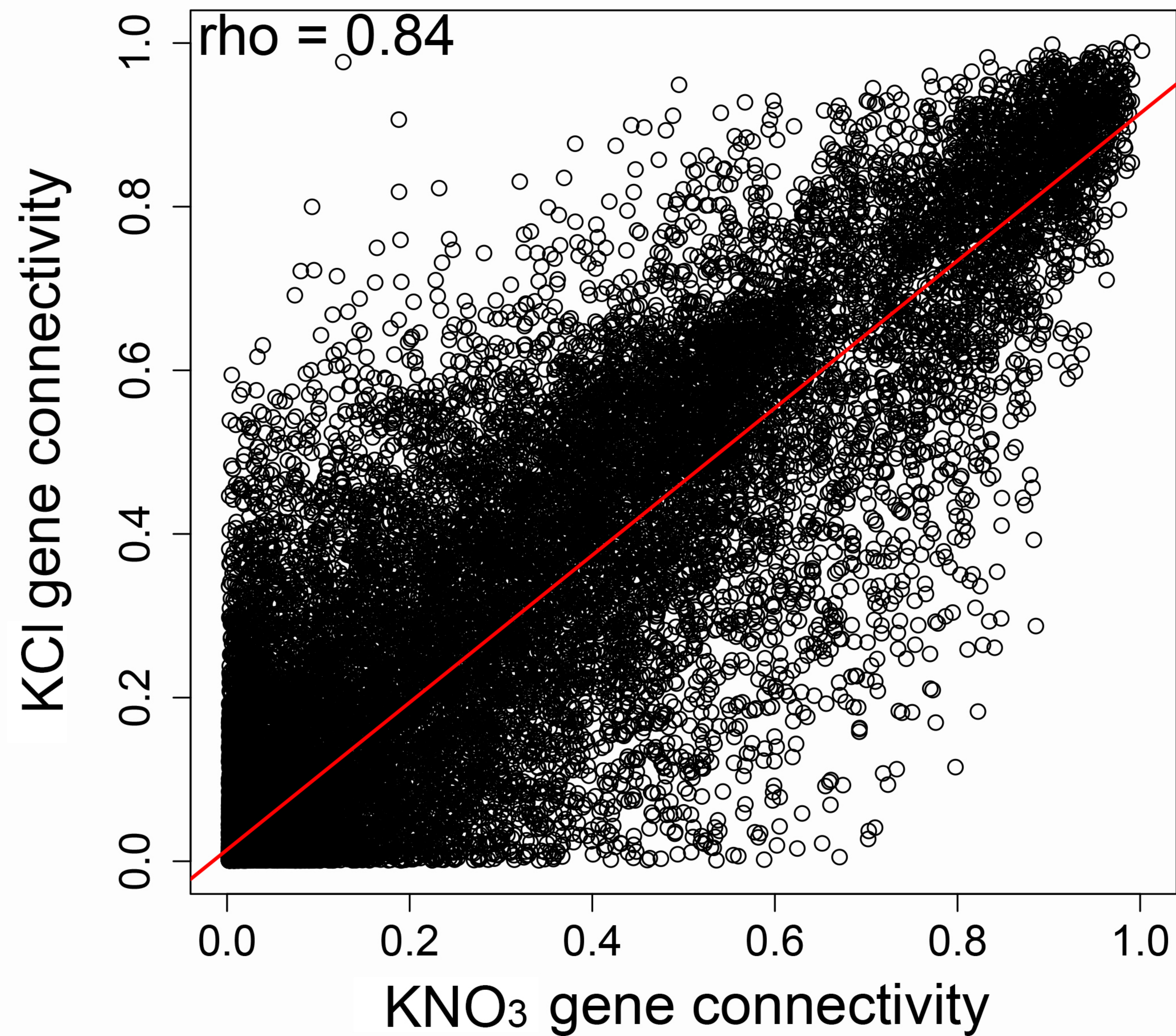

Supplement: Supplementary file 1 [file plants-09-01178-s001.zip › Supplementary_data/FigureS7.pdf]

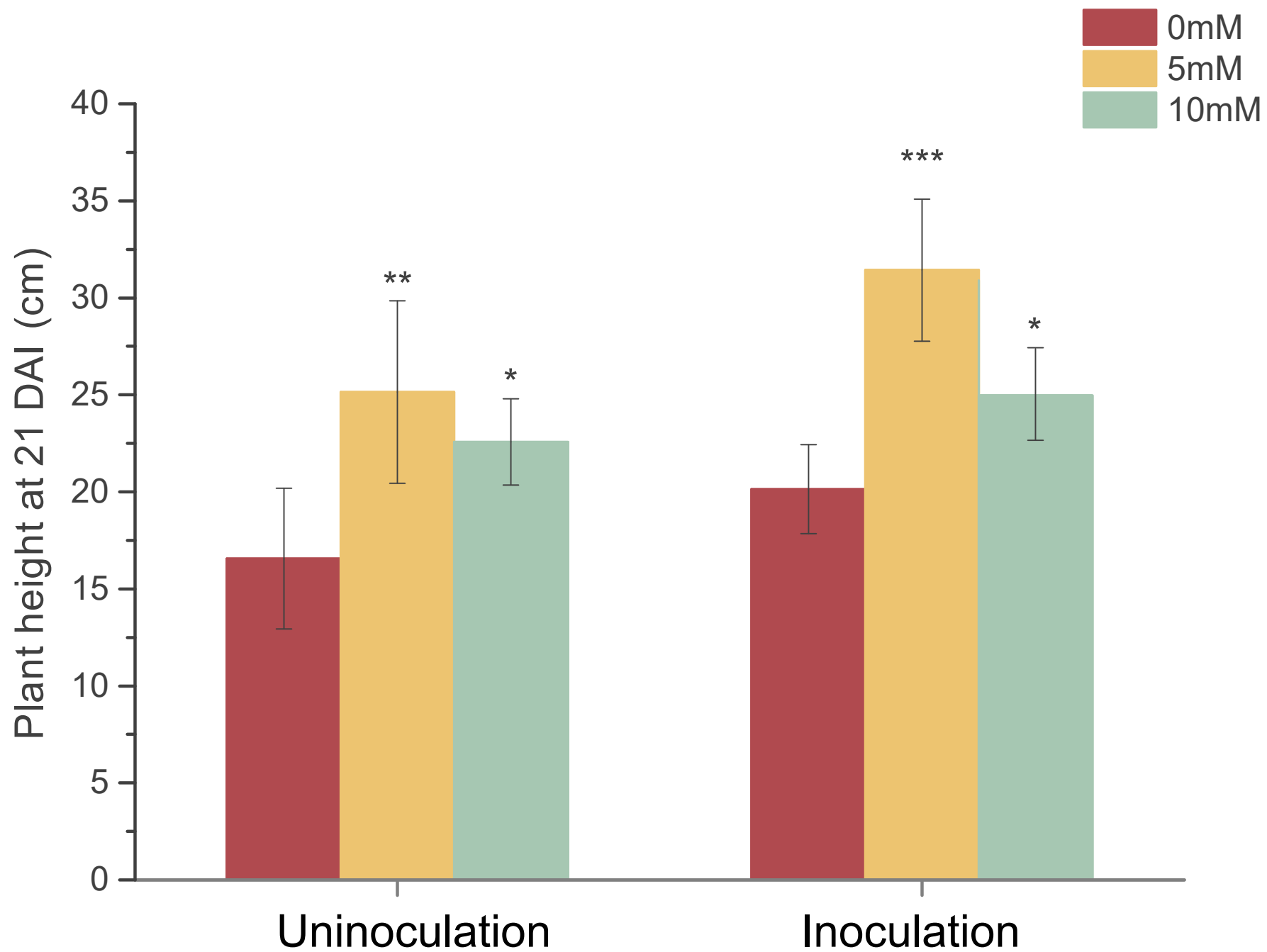

Supplement: Supplementary file 1 [file plants-09-01178-s001.zip › Supplementary_data/FigureS9.pdf]
